# Z-form DNA-RNA hybrid blocks DNA replication

**DOI:** 10.1093/nar/gkaf135

**Published:** 2025-03-04

**Authors:** Shiyu Wang, Yan Xu

**Affiliations:** Division of Chemistry, Department of Medical Sciences, Faculty of Medicine, University of Miyazaki, 5200 Kihara, Kiyotake, Miyazaki 889-1692, Japan; Division of Chemistry, Department of Medical Sciences, Faculty of Medicine, University of Miyazaki, 5200 Kihara, Kiyotake, Miyazaki 889-1692, Japan

## Abstract

We discovered that the Z-form DNA-RNA hybrid stabilized by methylated CpG repeats impacts on the initiation and elongation of Okazaki fragments, contributing to blocking DNA replication at first time. We further present the first Z-form DNA-RNA hybrid structure by using NMR spectroscopy and dynamic computation, revealing the molecular mechanism of inhibition, indicating that a distinctive zig-zag strand pattern of the Z-form hybrid with a smaller helical diameter (15 Å) and a very narrow minor groove (8.3 Å) plays the key role in the repression toward DNA replication.

## Graphical Abstract

**Graphical Abstract:**
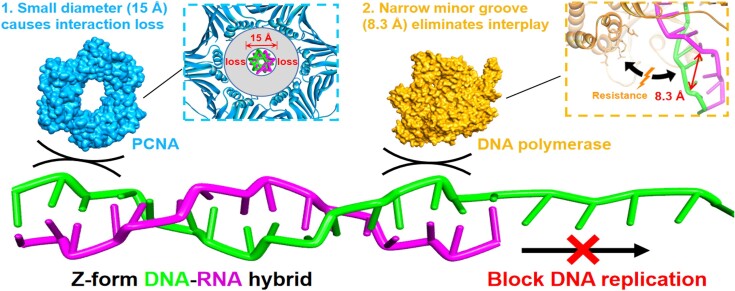

## Introduction

The left-handed Z-form double helix structure, including Z-RNA and Z-DNA, has been shown to have an important role in multiple physiological events [1–5], including the regulation of gene expression [6], nucleosome positioning [7, 8], and genetic instability associated with nucleic acid damage and repair [9]. We recently suggested the relationship of Z-form structure and several diseases, such as cancer and inflammation [10, 11]. In addition, we demonstrated that Zα domain proteins can induce phase separation and facilitate the conversion from A-form to Z-form [12]. Previous research has demonstrated that epigenetic modifications, such as the methylation of cytosine residues in CG-rich sequences, can facilitate and stabilize the formation of Z-form DNA structures [13–15]. Therefore, such prevalent Z-DNA and Z-RNA structures with a widespread of biological functionalities prompts us to explore new Z-form nucleic acids and these associated biofunction.

DNA-RNA hybrids serve pivotal functions in the intricate process of DNA replication. Their involvement is particularly pronounced in the initiation and elongation of Okazaki fragments, segments of newly synthesized DNA on the lagging strand [16–18]. These fragments are kickstarted by an DNA template/RNA primer hybrid, which provides the necessary starting point for DNA polymerase and the replication clamp proliferating cell nuclear antigen (PCNA) to commence elongation [19, 20].

In the canonical model of DNA replication, DNA-RNA hybrids emerge as crucial regulators, exerting precise control over the onset of DNA synthesis [21, 22]. These hybrids not only facilitate primer formation but also contribute to the coordination of enzymatic activities involved in strand elongation and maturation [23, 17]. Emerging evidence suggests that DNA-RNA hybrids act as signaling hubs, modulating the cellular response to replication stress and DNA damage, thereby safeguarding genomic integrity [24–26]. Their functions emphasize their indispensability in ensuring the faithful transmission of genetic information and the preservation of cellular homeostasis [27–29].

However, our understanding of how the DNA-RNA hybrid structure influences these functions, particularly in the initiation and elongation of Okazaki fragments, remains limited. For the first time, we have demonstrated that DNA methylation can stabilize Z-form DNA-RNA hybrids and block DNA replication. Further investigation reveals that these hybrids adopt a classical left-handed helical conformation, characterized by a smaller helical diameter (15 Å) and a much narrower minor groove (8.3 Å), which markedly differs from the canonical A-form DNA-RNA hybrid. These unique structural features enable Z-form DNA-RNA hybrids to act as formidable obstacles to DNA polymerase and PCNA recognition and binding, thus impeding their ability to serve as substrates for primer extension reactions. By elucidating the mechanistic basis underlying the inhibitory effect of Z-form DNA-RNA hybrids on DNA synthesis, we provide novel insights into the regulatory mechanisms governing DNA replication fidelity and efficiency. These findings not only expand our fundamental understanding of nucleic acid biology but also have potential implications for therapeutic interventions targeting DNA replication-related disorders.

## Materials and methods

### Synthesis of oligonucleotides

In this study, all oligonucleotides were synthesized (at trityl-off) at the condition of 1.0 μmol using 5-benzylthio-1H-tetrazole (0.25 M in MeCN) as the activator and 0.02 M iodine in tetrahydrofuran/pyridine/water (7:2:1, v/v) for oxidation. The standard synthesis cycle was used for assembly of the reagents and native nucleosides, except that the coupling time was extended to 20 min for artificial nucleosides. Cleavage from the solid support and deprotection were accomplished with the NH_4_OH aqueous (28%, w/w) and the methylamine aqueous solution (40%, w/w) at 50:50 ratio (v/v) at room temperature for 20 min and 65 °C for 10 min. For the deprotection of TBDMS moiety from 2′-OH of RNAs, the HF in TEA with DMSO was used and kept for 2.5 h at 65 °C. The crude oligonucleotides were purified by RP-HPLC on an inertsustainswift C18 column, 5 μm, 10 × 250 mm (GL Sciences) in a linear gradient of 50 mM triethylammonium acetate buffer (pH 7.4) in 1:1 acetonitrile*/*H_2_O and 50 mM triethylammonium acetate buffer (pH 7.4) in H_2_O. The purified oligonucleotides were quantified by nanodrop microvolume spectrophotometers (Thermo Scientific™) and confirmed by MALDI-TOF-MS on an Autoflex III smart beam mass spectrometer (negative mode) (Supplementary Data S34-S89).

### Protein and enzyme

E. coli DNA polymerase I (pol I) and 3′-5′ exonuclease were commercially obtained from TaKaRa Bio. They were used according to the manufacturer's instructions without any additional treatment. Human polymerase δ catalytic subunit p125 (pol δ) was ordered from Funakoshi and prepared as 1 μM in stock solution after dilution at -20 °C before use. The full-length ZBP1 was purchased from ORIGENE Technologies, Inc. (US). It's use follows manufacturing suggestion without further treatment. It's conservation by using Milli-Q with 10 mM EDTA under -70 °C until next use. Full-length human PCNA protein was ordered from NeoBiotechnologies and used following manufacturing suggestion.

### CD measurements

Solution (10 μM in duplex) was annealing at 85 °C for 3 min and gradually cooling to room temperature and incubating at 4 °C overnight. For the ZBP1 binding assay, DNA-RNA hybrid solution (1 μM in duplex) was used. Annealing and renature processes of DNA-RNA at 85 °C for 3 min and gradually cooling to room temperature, followed by incubating at 4 °C overnight. Remaining additional 2 h at room temperature before mixing with 1 μM ZBP1 and directly using for measurement. The experiments were performed at 10 or 37 °C and repeated twice in each sample, the average result was recorded as final presentation. Experimental condition: 150 mM KCl with indicated or 25 mM MgCl_2_ in 10 mM Tris-HCl, pH 7.0 and 1 mM EDTA for protein binding assay especially.

### UV-thermal melting assay

The DNA-RNA hybrid samples were prepared in 50 mM Tris–HCl (pH 7.0), 2 mM DTT, 1 × E. coli DNA poly I buffer, 150 mM KCl and 25 mM MgCl_2_ at 15 °C. The concentration of duplex was 10 μM. All samples were annealed by heatingat 90 °C for 10 min followed by a gradual cooling to r.t. Thermal denaturation was monitored at 260 nm at a rate of 1°C/min. The Tm values were determined from the first derivatives of the UV-melting curves.

### 1H NMR experiments

For 1D NMR measurement, DNA-RNA hybrid samples of 5 mM concentration were dissolved in 150 μL of designed solution containing 10% (exchangeable proton mode) or 100% (non-exchangeable proton mode) D_2_O, 100 mM KCl and 10 mM Tris-HCl buffer (pH 7.0). In the exchangeable proton mode, the NMR spectra recorded in 90% H_2_O/10% D_2_O water signal was suppressed using the 3–9–19 WATERGATE pulse sequence or excitation sculpting with gradient pulse. The data were processed with TopSpin 3.0 (Bruker BioSpin Gmbh) software and analyzed with MestReNova software. The 2D NOESY spectrum in 90% H_2_O/10% D_2_O (exchangeable proton mode) or 100% D_2_O (non-exchangeable proton mode) were collected from 360 scans with 150 ms mixing time at 20 °C. On average, 2048 complex points and 512 FIDs were collected within the spectral width of 14097 Hz. The sample solutions were as follows: 5 mM DNA-RNA hybrid duplex was dissolved in 150 μL of designed solution containing 10% or 100% D_2_O, 100 mM KCl and 10 mM Tris-HCl buffer (pH 7.0). Samples were prepared by heating the oligonucleotides at 85 °C for 3 min and gradually cooling to room temperature and incubating at 4 °C overnight.

### Structural determination

All assigned NOESY cross peaks were classified to strong (1.8–3.0 Å), medium (1.8–3.7 Å), weak (1.8–5.5 Å) and very weak (1.8–7.5 Å) interproton distance restraints based on the intensity of NOESY. The NOE peaks of H5-H6 from cytosine bases were used as calibration for the distance measurements. Distance restraints for the hydrogen bonding in each watson-crick base pair were 1.8–3.7 Å. The force constant of hydrogen bonds and NOE restraints were kept between 5 to 50 kcal mol^−1^ Å^−2^ throughout the computation. Then molecular dynamics simulations were performed by the standard dynamics cascade in BIOVIA Discovery Studio 4.5 with modifications. Generally, the structure was heating from 50 K to 300 K over 4ps and equilibration at 300 K with 100 ps simulation time. The save results interval in the production step was 2ps during 100 ps simulation time at 300 K. 10 best conformations generated by simulation were further energy minimized until the gradient of energy was less than 0.1 kcal mol^−1^. Next, NMR assigned coordinate was imported and the entire structure was run by energy minimization until the gradient of energy was less than 0.1 kcal mol^−1^. Total 10 conformers were obtained and the conformation with lowest energy was selected as the final presentation. In the case of molecular dynamics simulation using AMBER 18, the NMR assigned molecular coordinate was introduced into a standard dynamics cascade, which is heating from 50 K to 300 K and equilibration at 300 K with constant pressure of 1 bar. Next, the obtained results interval in the production step was 100ns during 250ns simulation time at 300 K. Total 50 conformations were produced and subjected to energy minimization to give the best 10 conformations. Helical parameters for the set of 10 final structures were evaluated using the program CURVES + v.2.6. All conformation parameters were subjected to statistical analysis. For construction of a longer Z-form DNA-RNA hybrid with 20 base pairs shown in Fig. 5C left, use NOE refined standard structure in 8 base pairs as a independent unit to repeatedly extend the helix, following the orientation from 5′ terminus to 3′ end in DNA strand as well as from 3′ terminus to 5′ end in RNA strand, until the duplex getting to 20 base pairs. This generated hybrid preserve the consistent structural features with the initial Z-form hybrid. For studying of absolute CG-repeated DRH_19_ model in Z-form hybrid, at first, the 4 mer fragment d(CGCG)/r(CGCG) from NMR restrained DRH_6_ model was used as a independent subunit to conduct a helix extension and get the 8 mer hybrid with sequence as d(CGCGCGCG)/r(CGCGCGCG). Next, the model was subjected to molecular dynamic simulation experiments by using BIOVIA Discovery Studio 4.5 or package Amber 18 following identical experimental flows in above and provide representative modeling structure of DRH_19_.

### Molecular modeling

We manually produced the model of Z-form DNA-RNA hybrid structures based on the reported Z-form structure (PDB code 1TNE and 1T4X) using the BIOVIA Discovery Studio 4.5 and package AMBER 18.

### ^19^F NMR measurement

0.1 mM DNA-RNA hybrid duplexes were dissolved in 150 μL of a designed solution containing 10 mM Tris-HCl buffer (pH 7.0) and 10% D_2_O in various concentrations of NaCl. The ^19^F NMR spectrum was measured on a Bruker AVANCE 400 MHz spectrometer at a frequency of 376.05 MHz and referenced to the internal standard CF_3_COOH (–75.66 ppm). The experimental parameters are recorded as follows: spectral width 89.3 kHz, ^19^F excitation pulse 15.0 μs, relaxation delay 1.5 s, acquisition time 0.73 s, scan numbers 1024–4096, and line width 3. Mixing time is 2 s. For in-cell ^19^F NMR measurement, the activity SLO (streptolysin O) was added for getting a final concentration of 0.1 μg mL^−1^. Then, the HeLa cells were incubated at 4 °C for 15 min with gentle rotation. The cells were washed three times with ice cold HBSS buffer, followed by incubation with 5 μM RNAs in 400 μL HBSS buffer at 37 °C for 30 min. For resealing of the HeLa cells membranes, CaCl_2_ was added to a final concentration of 1 mM and the HeLa cells were incubated at 37 °C for 30 min. The HeLa cells were washed three times with HBSS buffer containing 1 mM CaCl_2_. The resealed HeLa cells were treated by the 5 mM DNA-RNA hybrid duplex and then suspended in 200 μL of DMEM with 10% D_2_O and transferred to a Shigemi tube (Shigemi 5 mm Symmetrical NMR microtube). The experiment was performed at 296 K with a scan numbers value in 4096. After the intracellular NMR measurement, 100 μL of DMEM was added to the cell suspension, and the supernatant was collected by centrifugation at 4000 g for 30 min. The ^19^F NMR spectrum of the supernatant was measured with the same number of scans as the in-cell ^19^F NMR measurement.

### DNA replication assay in electrophoresis gel

For detection of DNA replication reaction product in gel image, 5′-Cy3-labeled RNA was used as primer. Condition as following, Cy3-labeled primer–template hybrid (2 μM) was incubated in 50 mM Tris–HCl (pH 7.0), 2 mM DTT, 1 × E. coli DNA poly I buffer, 150 mM KCl (total volume, 20 μL) and 25 mM MgCl_2_ at 15 or 37 °C. The polymerization was initiated by the addition of all four dNTPs at 1 mM final concentration and E. coli DNA poly I or DNA poly δ (5 U). Aliquots of the reaction mixtures were taken at pre-selected time points and quenched by heating at 70 °C for 10 min. The extension products were loaded onto 20% denaturing PAGE containing 7 M urea, running at 150 W for 1.5 h in 1 × TBE buffer, and visualized at Cy3 channel with ex. 540 nm and em. 570 nm using a phosphorimager (LAS-3000, Fujifilm).

### DNA replication assay in HPLC profile and MALDI-TOF MS

The total 20 μL reaction mixture including Cy3-labeled primer–template hybrid (2 μM), 50 mM Tris–HCl (pH 7.0), 2 mM DTT, 1 × E. coli DNA poly I buffer, 150 mM KCl, indicated concentrations of MgCl_2_, 1 mM dNTPs and E. coli DNA poly I or poly δ (5 U) were taken at pre-selected time points at 15 °C and quenched by heating at 70 °C for 10 min. The remained solution in a 10-fold dilution by mixing 10 μL reaction mixture with 90 μL deionized water and analysis by RP-HPLC on an inertsustainswift C18 column, 5 μm, 10 × 250 mm (GL Sciences) in a linear gradient of 50 mM triethylammonium acetate buffer (pH 7.4) in 1:1 acetonitrile*/*H_2_O and 50 mM triethylammonium acetate buffer (pH 7.4) in H_2_O, monitoring at 550 nm. The collected fractions were lyophilized and used for MALDI-TOF-MS on an Autoflex III smart beam mass spectrometer (negative mode).

### Z-form hybrid stability assay *in vitro*

For the study of DNA-RNA hybrid stability with various nucleases, DNase I (25 U mL^−1^), RNase A (1 mg mL^−1^) RNase H (50 U mL^−1^) in 1 μL volume were respectively added into hybrids solution (2 μM in 10 μL), the resulted sample was incubated at 37 °C for 10 min and used to analysis using the non-denaturing PAGE by phosphorimager (LAS-3000, Fujifilm).

For the study of RNA primer stability with 3′-5′ exonuclease, the DNA-RNA hybrid (2 μM in 10 μL) treated with 3′-5′ exonuclease in 180 U, the resulted mixture was incubated for 10 min at 37 °C and followed by the inactivation of enzyme in 5 min at 65 °C. The sample was loaded onto 10% denaturing PAGE and analyzed by using a phosphorimager (LAS-3000, Fujifilm).

For the study of stability of DNA-RNA hybrid double helix during DNA replication, Cy3-labeled primer–template hybrid (2 μM) was incubated in 50 mM Tris–HCl (pH 7.0), 2 mM DTT, 1 × E. coli DNA poly buffer, 150 mM KCl (total volume, 20 μL) and 25 mM MgCl_2_ at 15 °C, the resulted mixing samples at pre-selected time points was collected respectively and used to analysis using the non-denaturing PAGE by phosphorimager (LAS-3000, Fujifilm).

### Cell culture

HeLa cells (CCL2) and HT29 (HTB-38) cells grown in Dulbecco's modified Eagle's medium (DMEM) medium containing 10% FBS under a 5% CO_2_ atmosphere were harvested and washed twice by using Hanks’ Balanced Salt Solution (HBSS) buffer. Streptolysin O (Bioacademia) was activated with 10 mM DTT and 0.05% bovine serum albumin at 37 °C for 2 h.

### DNA replication assay in cells

These Cy3-labeled primer–template hybrids or RNA primer were transfected into live HeLa (CCL2) or HT29 (HTB-38) cells (1 × 10^7^) by using Lipofectamine 3000 (Thermo Fisher Scientific) according to the manufacturer's protocol in a final concentration of 5 μM. In details, Cy3-labeled hybrids or RNA primer (1 μM) was mixed with Lipofectamine 3000 solution for 20 min and added into cells in DMEM medium with FBS free. This mixture was incubated for 16 h at 37 °C under a 5% CO_2_ atmosphere condition and the cells washed by PBS buffer. Thereafter, these cells were lysed using 1 mL cell lysis buffer at room temperature for 30 min and subjected to 200 μL of 5 M NaCl following 12 h at 4 °C. The supernatant was collected after centrifugation at 12 000 × g for 30 min at 4 °C and got phase extracted by phenol-chloroform (1:3) for once. The aqueous layer was collected and centrifugation at 12 000 × g for 30 min at 4 °C, followed by lyophilization overnight. The dried samples were resolved in 100 μL nuclease-free water and mixed with 100 μL isopropanol for precipitation overnight at -20 °C. RNA was pelleted by centrifugation at 12 000 × g, washed by ice isopropanol. The extracted RNA were loaded onto 20% denaturing PAGE containing 7 M urea, running at 150 W for 1.5 h in 1 × TBE buffer, and visualized at Cy3 channel with ex. 540 nm and em. 570 nm using a phosphorimager (LAS-3000, Fujifilm). In order to study DNA replication using hybrids in different phases of cell cycles, phase arresting assay was conducted. For the G1 phase arrest, cells were incubated with 2 mM thymidine for 12 h, and then washed with PBS buffer. Subsequently, these cells were incubated in DMEM medium for 12 h and followed by treated with second round of thymidine to a final concentration of 2 mM in another 9 h. These cells were washed and kept in DMEM medium for 9 h before following experiments. For the S phase arrest, cells were incubated with 2 μM Antimycin A for 24 h and washed with PBS buffer. Next, these cells were allowed to incubated in DMEM medium for 48 h until subsequent experiment start.

### Immunofluorescence microscopy

These Cy3-labeled primer–template hybrids were transfected into live HeLa cells (5 × 10^4^) by transportation with Lipofectamine 3000 (Thermo Fisher Scientific) according to the manufacturer's protocol in a final concentration of 2 μM. The treated cells were washed by PBS buffer and fixed with 4% paraformaldehyde. Thereafter, these cells were permeabilized with 0.2% Triton X-100 and subjected to primary antibody, 5 μL Clone Z22 antibody (Sigma-Aldrich, primary antibody) at 37 °C overnight. After three washes in PBS, slides were incubated with 10 μL Alexa fluor 488 conjugated secondary antibody (2 mg/mL) (Santa Cruz, sc-516606, AF488) for 1 h at 37 °C. Following an additional three washes in PBS, slides were treated with 5 μg/mL Hoechst for 20 min and imaged by confocal microscopy on a TCS SP8 confocal microscopy (Leicamicrosystems). The data were recorded using Leica software. The laser of TCS SP8 for 63 × was HC PL APO CS2 63x/1.40 OIL. The red fluorescence for Cy3 dye was an excitation wavelength of 550 nm. The excitation/emission filter of 520 − 550 nm/550 − 600 nm was used. The green fluorescence for Alexa fluor 488 dye was an excitation wavelength of 488 nm. The excitation/emission filter of 480 − 490nm/495 − 520 nm was used. The blue fluorescence for Hoechst 33 258 was an excitation wavelength of 350 nm. The excitation/emission filter of 330 − 360 nm/430 − 450 nm was used. Max intensity z-projection and a single 0.5-μm optical slice was used for all images.When required, 5 μL DNase I (25 U mL^−1^), 100 μL RNase A (1 mg mL^−1^) and 0.83 μL RNase H (50 U mL^−1^) were added respectively and incubating at 37 °C for 1 h before additional primary antibody. For the detection of localized G1 and S phase in arresting cell cycle experiment, after the treatment using thymidine double block and Antimycin A to arrest cells, cdt1 antibody (Santa Cruz, sc-365305, AF488) and PCNA antibody (Santa Cruz, sc-25280, AF647) were used to label cdt1 and PCNA in nucleus after dilution with 1:100 in PBS buffer solution. Next, these cells were nuclear stained with 5 μg mL^−1^ Hoechst and imaged using TCS SP8 confocal microscopy (Leica microsystems).

### Measure K_d_ value between T/P hybrid and DNA polymerase

Fluorescence titrations were performed to determine the equilibrium dissociation constant (K_d_) of T/P hybrid and pol. The Cy3 labeled T/P hybrid was excited at 540 nm and the emission was observed at 565 nm. Fluorimetric titration experiments were performed on a fluorescence spectrophotometer (FP-8200, Jasco,Japan). A constant amount of T/P (100 nM) was titrated against increasing concentration of DNA polymerase I (0 − 500 nM) or DNA polymerase δ (0 − 200 nM) in the buffer (50 mM Tris-HCl, pH 7.0, 50 mM NaCl, 10 mM MgCl_2_, 5 mM DTT and 1 mM EDTA) at 25 °C. A control experiment was carried out in identical conditions with the presence of the unlabeled T/P (100 nM) and increasing amounts of the pols. The fluorescence changes from the control experiments were subtracted from the data obtained with the Cy3 labeled T/P, and the corrected values were plotted against the corresponding pol concentration. The dissociation constant K_d_ was calculated using the following equation, F = F_max_ × [pol]/K_d_ + [pol], where F is the relative fluorescence intensity, and F_max_ is its maximum value.

### Binding affinity assay between T/P hybrid and DNA polymerase or PCNA

The incubation mixture contained, in a final volume of 20 μL, 50 mM Tris-HCl, pH 7.0, 1 mM EDTA, 0.1mg/ml BSA, 10 mM MgCl_2_, 1.0 nM of Cy3 labeled T/P hybrid duplex with 0, 10 or 20 nM polymerase δ. After incubation for 5 min at 37 °C, the samples were subjected to electrophoresis in precooled 4% (w/v) non-denaturing PAGE, running at 150 W for 4.5 h in 1 × TBE buffer, and visualized at Cy3 channel with ex. 540 nm and em. 570 nm using a phosphorimager (LAS-3000, Fujifilm). For the study in use of PCNA, 1.0 nM T/P hybrid was in in a final volume of 20 μL, 50 mM Tris-HCl, pH 7.0, 1 mM EDTA, 0.1mg/ml BSA, 10 mM MgCl_2_, followed by additional PCNA at 0, 1 or 10 nM. This mixture was incubation for 1 h at 37 °C before running on non-denaturing PAGE and image. Nucleic acid staining reagent, gelstar (Lonza) was used by dilution with 1:1000 in running buffer to label DNA ladder (TaKaRa Bio.), which 10 μL in each well was used.

### Molecular dynamics simulations

Our initial models of human polymerase δ in complex with native DNA-RNA hybrid was based on ternary crystal structure PDB code 6NTY, 6P1H and 5M1S. In this complex structure, three residues in the thumb domain (residues 959, 960 and 968) interacted A-form DNA-RNA hybrid with incoming dTTP nucleotide as well as A-form DNA-RNA hybrid in the PCNA localization were resolved respectively through the standard dynamics cascade and energy minimization processes. To construct the Z-form DNA-RNA hybrid in complex with polymerase δ and PCNA, we modified and extended our NOE restraints refined Z-form structure, generating a 20 mer DNA/10 mer RNA hybrid that conserve consistent structural parameters. Next, the Z-form DNA-RNA hybrid was subjected to pol δ and PCNA respectively following standard dynamics cascade and energy minimization. Total 10 conformers were provided and the best one was selected as final presentation. The complex of Z-form hybrid-pol δ indicates multiple intermolecular pumps, proving it's quite unstable and cause structural crash. In the case of using Z-DNA to study it's binding with pol δ and PCNA, reported Z-DNA model (PDB: 1TNE) and protein model (PDB: 6NTY, 6P1H and 5M1S) were used. The whole flows adopt standard dynamics cascade and energy minimization. Total 10 conformers were provided and the best one was selected as final presentation.

### Quantification and statistical analyses

For the quantitative study of DNA replication reaction using electrophoresis gel imaging, three independent experiments were conducted. The primer extension yields were generated by the following equation: Yield (%) = reaction produced fluorescence intensity/reaction produced fluorescence intensity + starting fluorescence intensity × 100%, in which the fluorescence intensity was determined by ImageJ software. The reaction yields were normalized as well as calculation of standard deviation (σ^2^) among 3 results. For the quantitative assay of Z-form hybrid in live cells, in each group experiments, 3 independent experiments were completed and > 10 images obtained in each result. At least 5 images that were randomly selected from the totally obtained confocal images > 10 pieces containing intact cells, in which the representative images was divided into at least 16 subunits with equal size and the most representative one finally defined as presentation. The extent of Z-form hybrid formation in living cells was quantified by the mean green fluorescence intensity from total > 30 pixels (per pixel size 5 μm × 5 μm), in which the fluorescence intensity of each pixel was determined by ImageJ software (Wayne Rasband, NIH, USA). These pixels were afforded by equally dividing at least 5 independent imaging areas (∼ 400 μm^2^ each) that were randomly selected in the all obtained confocal images.

## Results

### Z-form DNA-RNA hybrid blocks DNA replication *in vitro*

**Figure 1.**
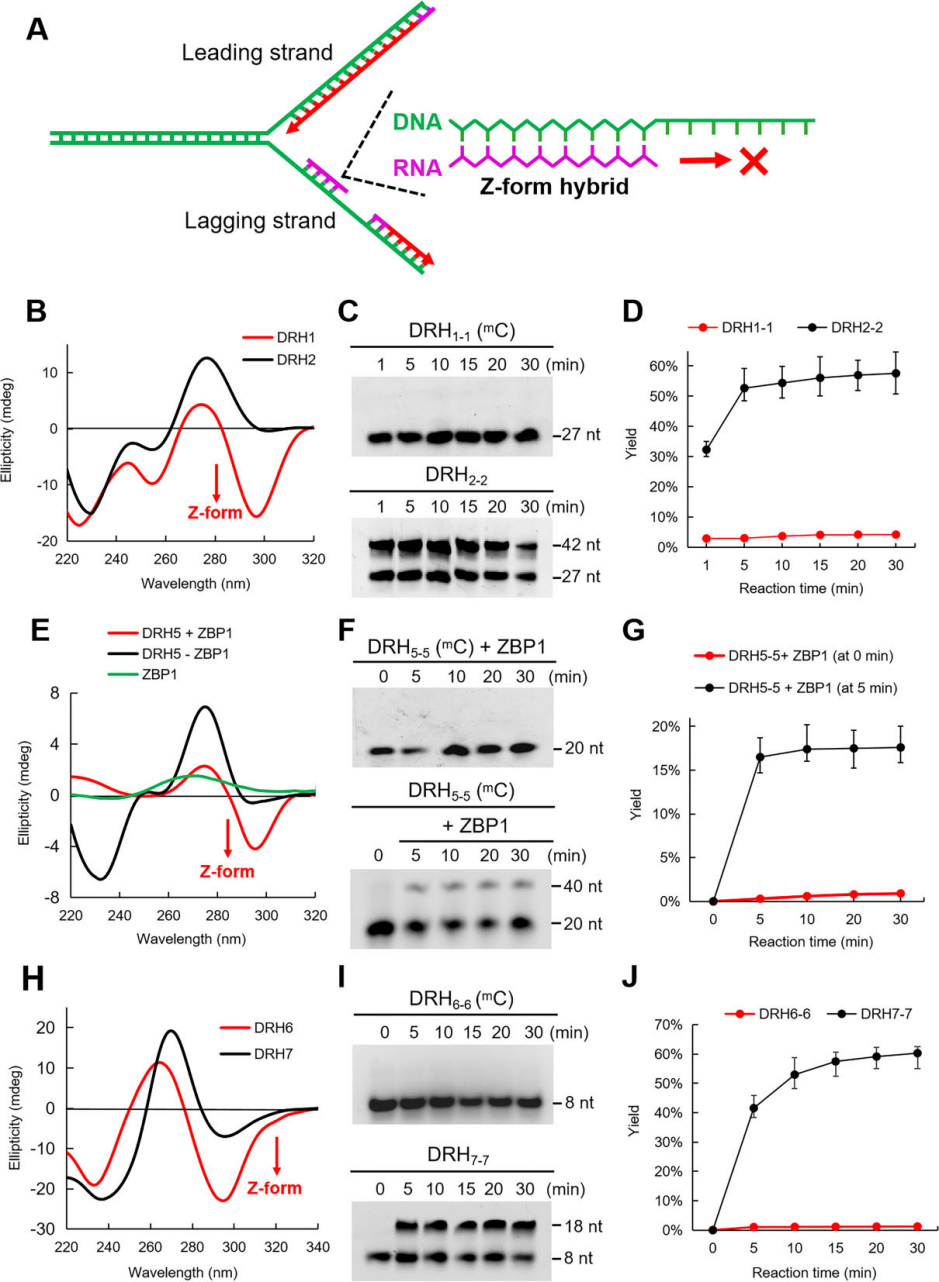
Z-form DNA-RNA hybrid blocks DNA replication *in vitro*. (**A**) Schematic representation of inhibitory effect of Z-form DNA-RNA hybrids on primer extension for Okazaki fragment synthesis in lagging strand. (**B**) CD spectra of DRH_1_ and DRH_2_, in which a clear negative band appeared at 295 nm, representing Z-form hybrid formation in DRH_1_ at 100 mM NaCl. (**C**) Pol δ catalyzed primer extension assay in time dependence using DRH_1-1_ and DRH_2-2_. (**D**) The DNA replication yields were plotted overtime derived from (**C**). (**E**) CD study of DRH_5_ with or without ZBP1 protein, in which additional ZBP1 in DRH_5_ induce a strong negative at 295 nm, representing Z-form hybrid formation. (**F**) Pol δ catalyzed primer extension assay in time dependence using DRH_5-5_ with additional ZBP1 at 0 or 5 min. (**G**) The DNA replication yields were plotted overtime derived from (**F**). (**H**) CD spectra of DRH_6_ and DRH_7_, in which a clear negative band appeared at 295 nm, representing Z-form hybrid formation in DRH_6_ at 100 mM NaCl. (**I**) Pol δ catalyzed primer extension assay in time dependence using DRH_6-6_ and DRH_7-7_. (**J**) The DNA replication yields were plotted overtime derived from (**I**). Error bars represent mean ± standard deviation. *n* = 3. ^m^C represent the hybrid including ^m^C residue on DNA strand..

Highly methylated CpG repeats are preferentially found in genomic locations and are enriched in genes associated with transcription, cell differentiation and development [30–32]. For example, CpG repeats with a high methylation level (up to 80%) can be observed in genes like Hdac9, REL, and Ano1 [4]. To assess the impact of the Z-form DNA-RNA hybrid bearing 5-methyl-2′-deoxycytidine (^m^C) on DNA replication (Fig. 1A), we prepared modified DNA oligonucleotides as templates whose sequences comprise CpG repeats bearing each of the known modified cytosines from Hdac9 and Ano1 genes (Supplementary Fig. S1A, B and Table 1). RNA oligonucleotides were labeled by Cy3 fluorophore at 5′-terminus to visualize the DNA extension product using electrophoresis gel assay. We used CD spectroscopy to monitor the conformational state of methylated DNA-RNA hybrid DRH_1_ from Hdac9 gene, showing a more intense negative band at 295 nm, presenting the Z-form CD spectra at physiological salt concentrations (Fig. 1B). In contrast, the non-methylated DNA-RNA hybrid DRH_2_ did not undergo a transition to the Z-form, instead displaying a positive intense band near 280 nm, indicative of the A-form (Fig. 1B). Using DNA polymerase δ (pol δ), an enzyme responsible for the extension of RNA/DNA primer to produce Okazaki fragments, almost no extended product was detected in the Z-form hybrid DRH_1-1,_ while extension products were observed from the A-form DRH_2-2_ (Fig. 1C and D, Supplementary Figs S31, S32, S36–S45). The strong inhibition effect by the Z-form hybrid was observed when the analyzing time was extended to 120 min, with results indicating no recovery of DNA replication (Supplementary Fig. S2). Similarly, for DRH_3_ from the human REL gene (Table 1), methylation can transfer the structure of the hybrid from the A-form to the Z-form based on CD characterization, but not for the non-methylated DRH_4_ (Supplementary Fig. S3A, B). No extended product was detected from DRH_3-3_, but extension result still was observed when using DRH_4-4_ (Supplementary Fig. S3C and D, S33, S34, S46–S54).

**Table 1.** DNA-RNA hybrid sequences used in this study

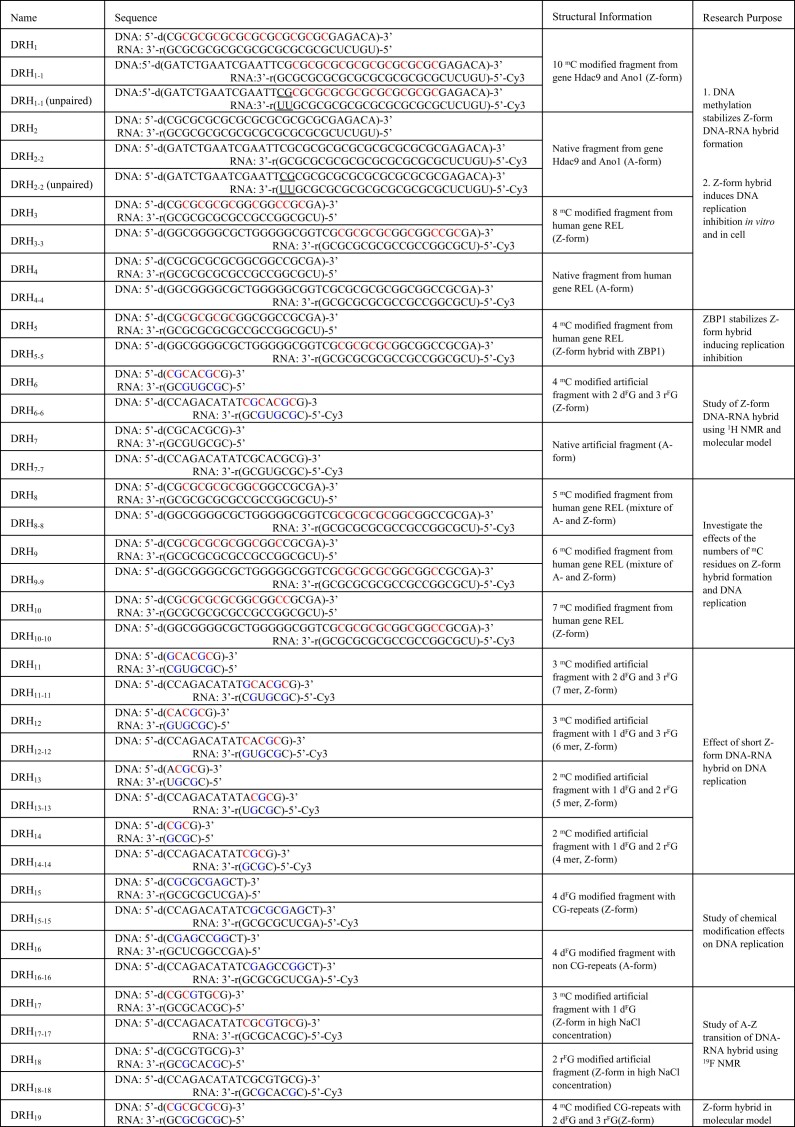

Red residues represent 5-methyl-2′-deoxycytidine (^m^C) on the DNA strands. Blue residues indicate 8-trifluoromethyl-2′-deoxyguanosine (d^F^G) on the DNA strands as well as 8-trifluoromethyl-guanosine (r^F^G) on the RNA strands. The nucleotides with underlines show 3′-terminus of RNA is unpaired to template DNA.

A Z-DNA-binding protein (ZBP1) has been demonstrated to bind and stabilize Z-helical structures [33, 34]. Next, we examined the inhibition of DNA replication by the Z-form DNA-RNA hybrid stabilized by full-length ZBP1, which includes intact structural and functional regions such as the Zα domain, thereby reflecting true physiological conditions. The hybrid DRH_5_, with a sequence similar to that of the human REL gene but containing a few ^m^C residues, primarily adopts an A-form structure while retaining the potential to transition into a Z-form. This design allows us to study whether it can bind to ZBP1 and subsequently form a Z-form structure that inhibits DNA replication. This approach is based on previous reports demonstrating that ZBP1 can bind to B-form DNA, which is prone to transition into the Z-form, and stabilize it as a Z-form structure [11, 35]. We observed that with the addition of ZBP1, the DRH_5_ duplex underwent with a dramatic A − Z transition even at the less methylation sequence, as evidenced by the appearance of a strong negative band at 295 nm in the CD spectrum (Fig. 1E). Consistently, we found that the extension reaction of DRH_5-5_ immediately terminated at different time points when ZBP1 was added (Fig. 1F and G, Supplementary Figs S35, S55–S57). These results indicate that Z-form hybrid formation can be triggered by ZBP1, thereby blocking DNA replication.

To further explore whether the Z-form conformation can block DNA replication when using different sequences in hybrid, we designed and synthesized shorter hybrid sequences DRH_6_ and DRH_7_ with CpG repeats, in which trifluoromethyl (CF_3_) group was incorporated into the C8 position of 2′-deoxyguanosine or guanosine (Supplementary Schemes S1 and S2, Supplementary Data S1–S33). As reported in our previous research, a CF_3_ group at the C8 position stabilizes Z-form nucleic acids and acts as a ^19^F sensor, which can be used to study the DNA and RNA structures using ^19^F NMR [36]. We observed that CF_3_ modified DNA-RNA hybrid, DRH_6-6_, did not produce observable extension products when using pol δ as catalyst (Fig. 1I and J). Conversely, the native DNA-RNA hybrid DRH_7-7_ sharing the identical sequence with DRH_6-6_, can properly drive DNA replication (Fig. 1I and J). These findings are consistent with CD results showing that DRH_6_ adopting a Z-form structure, while DRH_7_ remains in an A-form (Fig. 1H). Using a more common enzyme, E. coli DNA polymerase I (pol I), we observed similar results: the modified DRH_6-6_ blocked primer extension, while non-modified DRH_7-7_ produced extended fragments (Supplementary Fig. S4A–C).

To investigate the effect of ^m^C residue number on Z-form stability and DNA replication blockage, we synthesized hybrids with varying numbers of ^m^C residues (e.g. DRH_5_ with 4 ^m^C, DRH_8_ with 5 ^m^C, DRH_9_ with 6 ^m^C, and DRH_10_ with 7 ^m^C residues). CD spectra revealed that increasing ^m^C residue numbers enhanced Z-form stability. For example: DRH_5_ showed a near-A-form structure. DRH_8_ (5 ^m^C), DRH_9_ (6 ^m^C), and DRH_10_ (7 ^m^C) exhibited progressively stronger Z-form characteristics, as evidenced by an increasingly negative band at 295 nm (Supplementary Fig. S5B). DNA replication assays demonstrated that hybrids with stronger Z-form characteristics inhibited DNA replication more effectively. Specifically, DRH_10-10_ (7 ^m^C) caused near-complete replication inhibition, DRH_8-8_ and DRH_9-9_ showed moderate inhibition (Supplementary Fig. S5A and C). DRH_5-5_ (4 ^m^C) exhibited no significant replication inhibition compared to DRH_4-4_ (no ^m^C residues) (Supplementary Fig. S3C). These results indicate that hybrids with at least 5 ^m^C residues begin to stabilize Z-form and block replication, with 7 ^m^C residues showing maximum inhibitory effects.

To determine the minimum length of Z-form hybrid sequences required to effectively block replication [37], we synthesized and tested a shorter hybrid, 7-mer DRH_11-11_. CD spectroscopy confirmed that DRH_11-11_ adopts the Z-form structure, as evidenced by the strong negative band observed at 295 nm (Supplementary Fig. S6A). UV-melting curve analysis revealed a melting temperature (Tm) of 37 °C, consistent with physiological conditions, indicating that DRH_11-11_ is stable in its duplex form and capable of blocking DNA replication (Supplementary Fig. S6B). DNA replication assays with Z-form DRH_11-11_ demonstrated no primer extension, confirming its ability to block DNA replication (Supplementary Fig. S6C and D). Additionally, we synthesized and analyzed even shorter hybrids, 6-mer DRH_12-12_, 5-mer DRH_13-13_, and 4-mer DRH_14-14_, to examine their structures and effects on DNA replication. CD spectroscopy showed that the characteristic negative band at 295 nm weakened progressively as the sequence shortened, indicating the loss of Z-form characteristics in shorter hybrids (Supplementary Fig. S6A) [38]. UV-melting assays revealed that these shorter hybrids were structurally unstable, with Tm values of 30 °C for DRH_12-12_ and 18 °C for DRH_13-13_. DRH_14-14_ was too unstable to yield a measurable Tm, suggesting that these hybrids transitioned to single-stranded forms under physiological conditions (37 °C) and could not block DNA replication through Z-form stabilization (Supplementary Fig. S6B). Consequently, no DNA replication inhibition was observed with these shorter hybrids, as they failed to form the template/primer hybrid necessary for initiating replication and lacked Z-form structure-induced suppression (Supplementary Fig. S6C and D). Collectively, these experiments concluded that the 7-mer DRH_11-11_ represents the minimum length of Z-form hybrid capable of blocking DNA replication, as shorter hybrids are unstable and unable to adopt the Z-form under physiological conditions.

Moreover, we further confirmed that the DNA replication inhibition was induced by the typical Z-form structure rather than other effects, such as modification-triggered hybrid unwinding during DNA replication. Therefore, a control experiment was carried out under pol δ synthesis buffer conditions to assess whether the Z-form hybrid could stably exist. The time-dependent electrophoresis gel assay revealed that the bands of Z-form DRH_3-3_ displayed lower mobility than RNA primer alone, consistent with the results observed when A-form DRH_4-4_ was used (Supplementary Fig. S7A and B), indicating the stable hybrids. Additionally, a UV-melting assay demonstrated consistent melting temperatures (Tm) for the Z-form (72 °C) and A-form (71.5 °C) at both 0 and 30 min of incubation in the pol δ buffer solution, confirming that these structures remain stable during the DNA synthesis reaction (Supplementary Fig. S7C and D).

To assess whether DNA replication inhibition arises from chemical modifications rather than structural effects, we synthesized DRH_15-15_ and DRH_16-16_, which contain identical numbers of guanine, cytidine, and ^F^G residues but adopt different secondary structures (Z-form for DRH_15_ and A-form for DRH_16_, as confirmed by CD spectroscopy; Table 1, Supplementary Fig. S8A). DNA replication results indicated that the Z-form hybrid caused a similar inhibitory effect, whereas the A-form hybrid allowed proper primer extension (Supplementary Figs. S8B–D).

To evaluate whether the blunt-end formation at the 3′ terminus of RNA in the Z-form hybrid contributes to replication inhibition, we utilized 3′-5′ exonuclease, which specifically cleaves unpaired 3′-terminal nucleotides. Results showed no cleaved RNA when A-form (DRH_2-2_) and Z-form (DRH_1-1_) hybrids were subjected to 3′-5′ exonuclease. However, a control experiment performed using an RNA primer with an unpaired 3′ terminus demonstrated that the unpaired residues on the 3′ terminus of the RNA primers (DRH_1-1_ and DRH_2-2_) could indeed be cleaved (Supplementary Figs. S9A and B). To further confirm duplex stability, UV melting assays were performed to determine the melting temperature (Tm) of the hybrids. The Z-form hybrid (DRH_1-1_) exhibited greater stability (Tm = 79 °C) compared to the A-form hybrid (DRH_2-2_, Tm = 77 °C). However, lower Tm values were indicated from DRH_1-1_ (unpaired) (72°C) and DRH_2-2_ (unpaired) (71.5 °C) (Supplementary Fig. S9C). These results collectively demonstrate that the terminal base pairs of the Z-form hybrid remain intact under the experimental conditions. These findings support the conclusion that the observed DNA replication blockage is caused by the specific secondary structure of the Z-form hybrid rather than by terminal structure dissociation or other factors.

### Z-form hybrid inhibits DNA replication in cells

**Figure 2.**
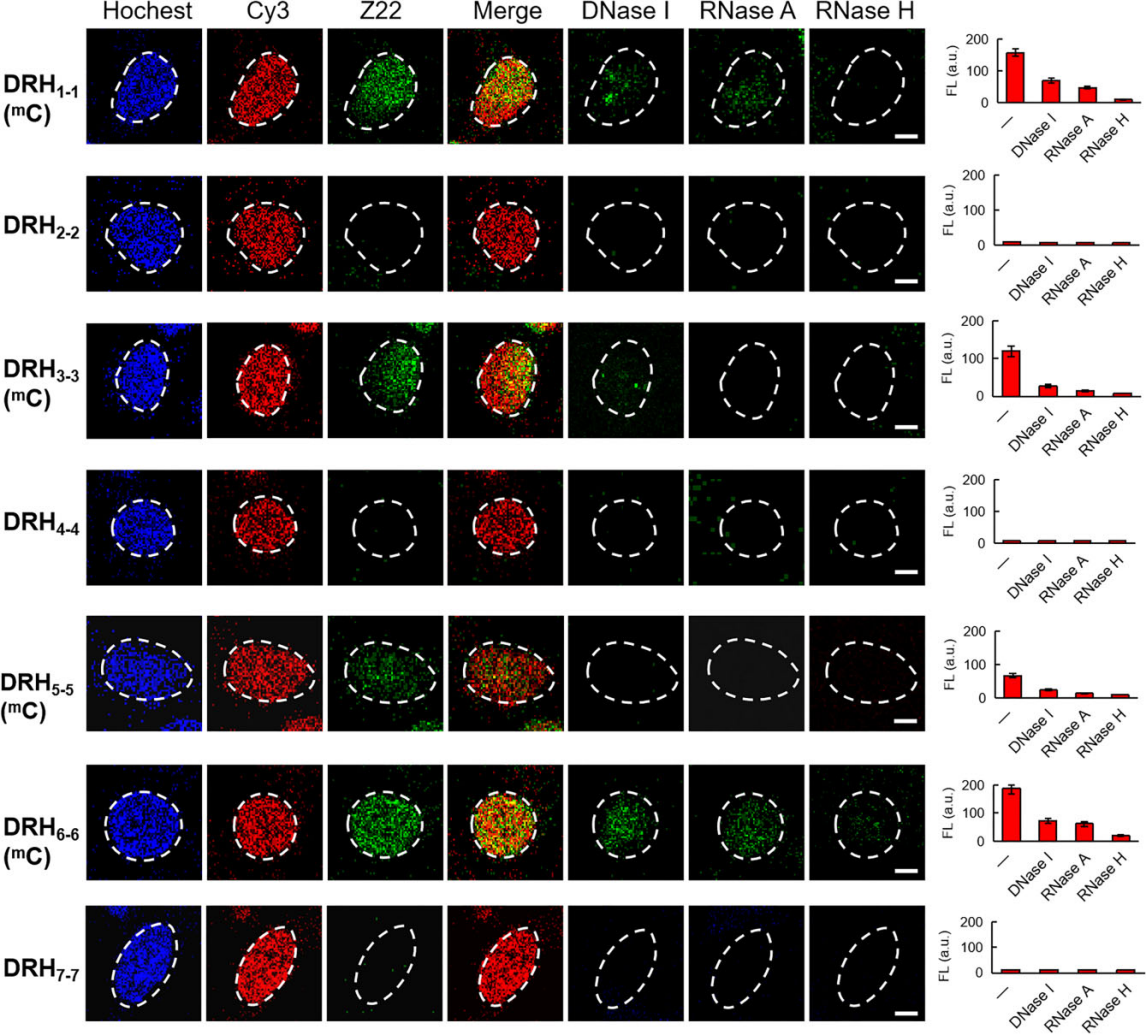
Determination of Z-form DNA-RNA hybrids in HeLa cells. In immunofluorescence assay, DRH_1-1_, DRH_3-3_, DRH_5-5_, and DRH_6-6_ show Z-form structure as observation of green fluorescence in use of Z22 antibody as well as fluorescence decay after treatment with DNase I, RNase A and RNase H respectively. The green fluorescence intensities representing Z-form structure contents were quantitatively analyzed with different treatments as plotted histogram (right column). Nuclei are stained by Hochest 33342 as blue color and outlined with dashed white lines. The red fluorescence from Cy3-labeled RNA within the DNA-RNA hybrid. The merge image was the overlay result between red fluorescence image (Cy3 labeled RNA) and green fluorescence image (Z22). Scale bars, 10 μm. Error bars represent mean ± standard deviation. *n* = 3. ^m^C represents the ^m^C residue on DNA strand.

Encouraged by the ability of Z-form hybrid blocking primer extension *in vitro*, we sought to assessed that whether these hybrids could adapt in Z-form hybrid and similarly inhibit DNA replication in cells. The Z-conformation of hybrids were determined by immunofluorescence assay using a Z22 antibody, which typically recognizes Z-form nucleic acid duplexes. The results show consistent structural characteristics of these hybrids in cells and *in vitro* based on their binding with the Z22 antibody, indicated by clear green fluorescence (Fig. 2). In detail, green fluorescence were observed in use of Z22 antibody when DRH_1-1_, DRH_3-3_, DRH_5-5_, and DRH_6-6_ were used, and mainly located in the nucleus, entirely overlapped with the Cy3-labeled hybrid in red, resulting in a yellow signal. Moreover, the signals were sensitive to DNase I, RNase A, and especially RNase H. These results were further supported by *in vitro* studies, where Z-form hybrids were subjected to digestion using various nuclease treatments (Supplementary Fig. S10). These findings collectively demonstrated that these hybrids predominantly adopt Z-form structure in cells (Fig. 2). In contrast, DRH_2-2_, DRH_4-4_ and DRH_7-7_ did not show fluorescence when treated by Z22 antibody, indicating these hybrids cannot form Z-form structure in cells, which is consistent with *in vitro* (Fig. 2).

**Figure 3.**
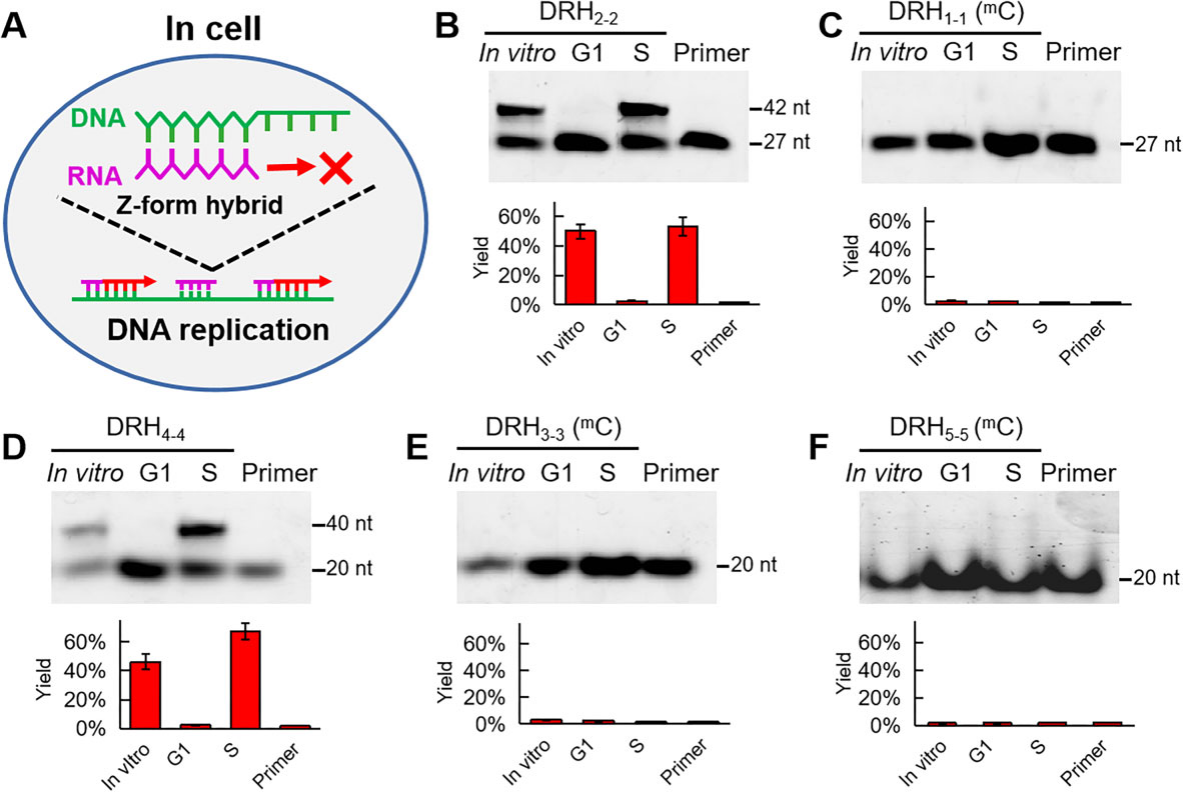
Z-form DNA-RNA hybrid blocks DNA replication in HeLa cells. (**A**) Concept diagram of studying Z-form DNA-RNA hybrid block DNA replication in cells. (**B**) Study DNA replication using DRH_2-2_ in cells, the yields in plotted histogram. (**C**) Study DNA replication using DRH_1-1_ in cells, the yields in plotted histogram. (**D**) Study DNA replication using DRH_4-4_ in cells, the yields in plotted histogram. (**E**) Study DNA replication using DRH_3-3_ in cells, the yields in plotted histogram. (**F**) Study DNA replication using DRH_5-5_ in cells, the yields in plotted histogram, *in vitro* represent ZBP1 was used. Error bars represent mean ± standard deviation. *n* = 3. Primer shows the independent Cy3-RNA used in each hybrid. ^m^C represents the ^m^C residue on DNA strand. To arrest the G1 phase in cells, in brief, 2 mM thymidine was added and incubated in cells for 12 h. The cells was washed and then incubated in DMEM medium for 12 h and followed by treated with 2 mM thymidine in another 9 h. For arresting S phase, cells was incubated with the Antimycin A (2 μM) for 24 h, and then the cells were washed and readily used for next experiments (details in “Materials and methods”).

Next, we used DNA template/RNA primer hybrids to investigate temporal course of Z-form hybrid blocking DNA replication in cell cycles, these allows us to reveal its natural physiological functionalities in maintaining epigenetic stability during cell division. To get cells in the G1 and S phases of the cell cycle, thymidine and Antimycin A were used, according to previous reports [39–43]. Immunofluorescence assays demonstrated strong nuclear enrichment of the G1-phase marker Cdt1 in thymidine-treated cells and bright clusters of the S-phase marker PCNA in Antimycin A-treated cells [44–46] (Supplementary Figs S11 and S12). These findings confirmed that cells in the G1 and S phases were effectively arrested. Subsequently, the effects of Z-hybrid on DNA replication were evaluated in the different cell cycle phases (i.e. G1 and S phases) (Fig. 3A). We used the A-form DRH_2-2_ and methylated Z-form DRH_1-1_ to treat living human cells and found that the DRH_2-2_ in the A-form could initiate primer extension during the S phase but not during the G1 phase, which is consistent with the well-known fact that DNA replication only occurs in the S phase (Fig. 3B). Notably, we observed no extension product for DRH_1-1_, even during the S phase (Fig. 3C). Similarly, A-form DRH_4-4_ could initiate DNA replication (Fig. 3D), while no DNA extension was observed when Z-form DRH_3-3_ and DRH_5-5_ were used (Fig. 3E and F).

Additionally, we observed the extended products from the A-form DNA-RNA hybrid DRH_7-7_ in HeLa and HT29 cells (Supplementary Fig. S13B and D). Importantly, no product was observed in the Z-form hybrid DRH_6-6_, these results are identical to *in vitro* (Supplementary Fig. S13A and C), indicating that the Z-form hybrid can block DNA replication under physiological conditions.

### NMR reveals Z-form DNA-RNA hybrid structure

To clarify the mechanism by which the Z-form hybrid can block DNA replication, the detailed structure of the Z-form hybrid was investigated using ^F^G-modified DRH_6_. ^F^G is an excellent Z-form stabilizer that strongly induces Z-form structure formation under physiological salt conditions, effectively minimizing interference from other nucleic acid structures, such as single strands or A-form hybrids, in NOESY spectra. Importantly, the use of ^F^G does not alter the intrinsic Z-form structure, as confirmed by previous studies employing various chemical modifications as Z-form stabilizers to study Z-form nucleic acids [47–49]. This approach ensures accurate representation of the Z-form hybrid's structural characteristics.

**Figure 4.**
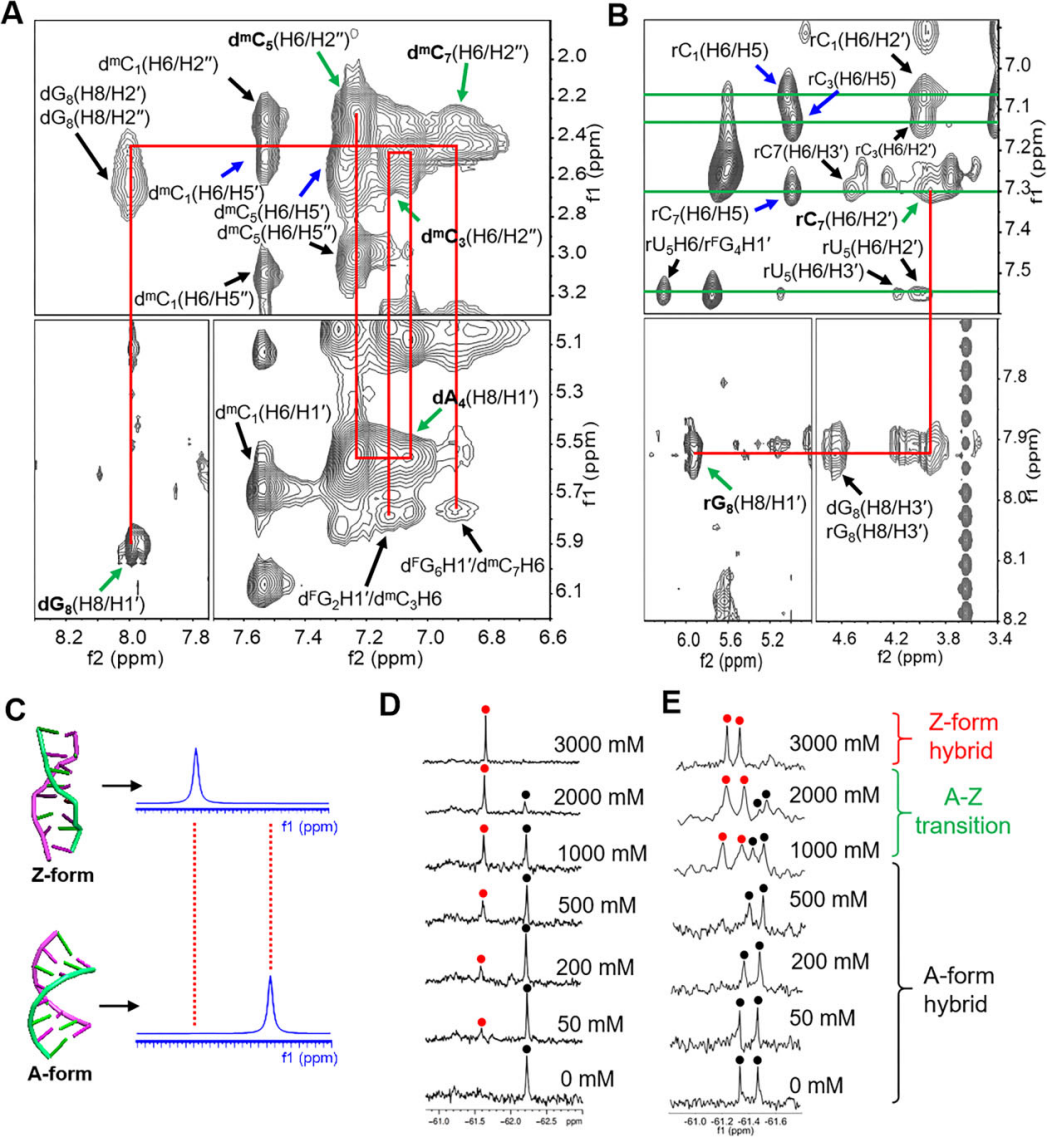
NMR study reveals distinctive Z-form DNA-RNA hybrid. (**A**) 2D-NOESY of DRH_6_ shows the connectivity path (red lines) in DNA strand and labeled by green arrows. d^m^C_1_(H6/H5′) and d^m^C_5_(H6/H5′) were found to upshift 2.49 and 2.31 ppm (blue arrows). Black arrows indicated the intra-residue and inter-residue cross peaks (mixing time: 400 ms). (**B**) 2D-NOESY of DRH_6_ indicates connectivity path (red lines) in RNA strand and labeled by green arrows. The signal upshift is indicated in blue arrows. Black arrows indicated the intra-residue and inter-residue cross peaks. All NOEs linked to H6 of rC_1_, rC_3_, rU_5_ and rC_7_ were connected as green lines (mixing time: 250 ms). (**C**) Concept for the detection of A–Z transition by ^19^F NMR. ^19^F NMR signals are strongly dependent on the structural environment of the ^19^F label. (**D**) ^19^F NMR spectra of DRH_17_ with increasing NaCl concentrations. (**E**) ^19^F NMR spectra of DRH_18_ with increasing NaCl concentrations.

A complete list of ^1^H chemical shifts is showed in Supplementary Table S1. In the non-exchangeable proton spectrum, we successfully assigned the proton resonances based on the ‘walk along helix’ using NOE connectivity path: d^m^C_3_(H6/H2″)-dA_4_(H8/H1′)-d^m^C_5_(H6/H2″), d^m^C_7_(H6/H2″)-dG_8_(H8/H1′) in the DNA strand (Fig. 4A) and rC_7_(H6/H2′)-rG_8_(H8/H1′) in the RNA strand (Fig. 4B), indicating sequence-specific connectivity for left-handed helix. We observed an upshift of the H5 protons of rC_1_, rC_3_, and rC_7_ around 5.04–5.08 ppm (Fig. 4B), as well as H5′ of d^m^C_1_, d^m^C_3_, and d^m^C_5,_ and H2′ of cytosine residues in the DNA strand with a similar upfield shift (Fig. 4A, Supplementary Fig. S14A and B and S16A). These alterations are only observeable in Z-form nucleic acid duplexes. Strong signals of H8/H1′ cross peaks derived from dA_4_, dG_8_ and rG_8_ indicate the *syn* conformation of these residues (Fig. 4A and B), while intra-residue cross peaks of H6/H5″ of all cytosine residues, which indicate the *anti* conformation (Fig. 4A, Supplementary Fig. S14C), are characteristic of the Z-form duplex.

In the exchangeable proton NMR spectrum, similar to previous studies, the cross-peak of d^m^C_5_NH2_1_ and rC_3_H5 was observed, which is predominantly induced by the typical base pairs stacking patterns of the Z-form structure (Supplementary Fig. S14D). Moreover, stable Watson–Crick base pair formation was confirmed by a series of clear cross-peaks from d^m^C_5_NH2_1_/r^F^G_4_H1, d^m^C_5_H6/r^F^G_4_H1, d^m^C_7_NH2_2_/r^F^G_2_H1, and d^F^G_6_H1/rC_3_H1′ in the Z-form hybrid duplex (Supplementary Fig. S15). Additionally, NOEs between H1′ and H2′ from all cytosine and uracil residues were detected, demonstrating that these nucleosides adopt a C2′-*endo* sugar pucker. Conversely, H3′/H4′ cross peaks of guanine and adenine residues appeared, indicating a C3′-*endo* conformation (Supplementary Fig. S16).

Exceptionally, ^19^F NMR was employed to observe Z-form structure formation, as ^19^F signals are highly sensitive to the surrounding chemical environment and produce distinct NMR resonances with high relative abundance and no background interference [50–52]. We have recently shown that the ^19^F NMR can be used to study the Z-form structure [11, 36]. Here, a Z-form hybrid, DRH_17_ that is able to inhibit DNA replication, was used because it possessing only one CF_3_ group, could produce clear ^19^F NMR spectroscopy (Supplementary Fig. S17A–C). The results with gradient NaCl concentrations indicated that the single peak at -62.22 ppm, representing A-form structure progressively decreased and a new signal enhanced around -61.61 ppm, demonstrating Z-form conformation (Fig. 4D). These findings are consistent with the CD spectroscopy results (Supplementary Fig. S17A). Moreover, a hybrid DRH_18_, modified with two CF_3_ groups, was employed to examine its Z-form characteristics. Results demonstrated that this hybrid also adopts a Z-form structure and effectively blocks the DNA extension reaction (Supplementary Figs. S17D–F). Results revealed that two new strong-intensity peaks appeared as the Z-form hybrid (−61.28 and − 61.38 ppm) with increasing NaCl concentration, compared to the two peaks in the A-form hybrid that decreased and completely disappeared, and the four peaks observed during the A-Z transition state, which are completely consistent with CD spectra (Fig. 4E and Supplementary Fig. S17D).

### Structural insights into Z-form DNA-RNA hybrid and A-form hybrid

**Figure 5.**
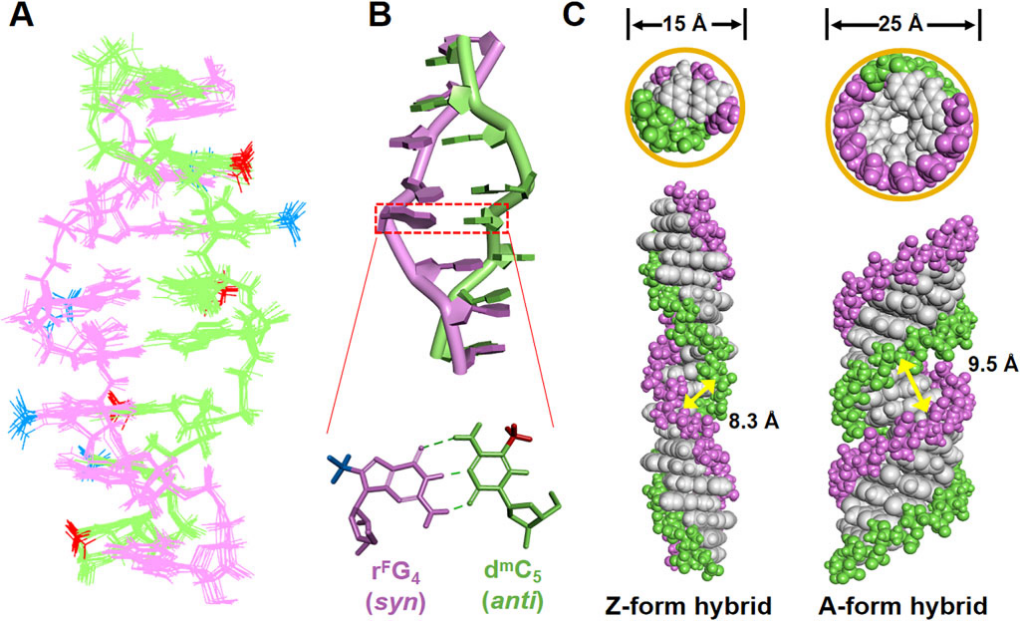
Molecular model of Z-form DNA-RNA hybrid and comparison with A-form hybrid. (**A**) Superposition of 10 conformers representing the refined structure of DRH_6_ in the Z-form with lowest total energy as viewed from the minor groove. (**B**) Molecular model of Z-form hybrid DRH_6_, DNA strand (green) and RNA strand (pink). An enlarged view showed the Watson–Crick base pair of r^F^G_4_ and d^m^C_5_. (**C**) A structural comparison of hybrid at Z-form and A-form, Z-hybrid with much smaller diameter 15 Å and a narrower and rigid minor groove at 8.3 Å, while A-hybrid with a larger diameter at 25 Å and wide and flexible minor groove around 9.5 Å.

The molecular dynamic simulation was carried out in BIOVIA Discovery Studio 4.5 through a standard dynamic cascade with some modifications based on the reported Z-form structure using NOE constrains refined model. Consequently, a intact structural model of Z-form DRH_6_ was constructed (Supplementary Fig. S18A and B). 10 Conformers with lowest energy in superposition was shown in Fig. 5A, in which the most appropriate one that can represent the mean dynamic evolution was selected as well as viewed at major (Fig. 5B, Supplementary Fig. S18A) and minor groove (Supplementary Fig. S18B), respectively, in which representative Watson-Crick base pair was shown as r^F^G_4_:d^m^C_5_ showing typical *syn* conformation for guanosine and *anti* conformation in cytidine that is the hallmark for Z-form structure formation (Fig. 5B). Addtionally, all Watson-Crick base pairs of the Z-form hybrid were performed in Supplementary Fig. S18C. It should be noted that the Z-form hybrid matched with zig-zag shape as classical Z-helix conformation according to observation of coordinated variability of ϵ and ζ angles of cytidine residues and α, β angles of guanosine residues for both CpG and GpC steps. (Supplementary Tables S2 and S3).

More importantly, two unusual structural features were observed in the Z-hybrid. First, the minor groove is notably narrow (width, 8.3 Å) compared to the A-hybrid (9.5 Å). Second, the Z-hybrid structure has a diameter of only 15 Å, making it the smallest among all discovered duplex conformations (Fig. 5C, Supplementary Table S4) [53]. To further substantiate the defined Z-form hybrid structure, molecular modeling was conducted using Amber 18 and compared the models generated through BIOVIA Discovery Studio 4.5. Results demonstrated a Z-form hybrid with highly consistent helical parameters and structural features, particularly the narrow minor groove (8.3 Å) and a small diameter (15 Å), as detailed in Supplementary Tables S5, S6, S11, and S12, and Supplementary Fig. S19. To confirm the robustness of the Z-form hybrid's structural characteristics across different sequences, we analyzed an alternative sequence, DRH_19,_ derived from the human REL gene, containing an absolute CG-repeat fragment. The model was built by extending the 4-mer fragment d(CGCG)/r(CGCG) (from the NMR-restrained DRH_6_ model) to generate the 8-mer sequence d(CGCGCGCG)/r(CGCGCGCG). Molecular dynamics simulations using BIOVIA Discovery Studio 4.5 and Amber 18 were carried out to refine the DRH_19_ structure. The structural data for DRH_19_, summarized in Supplementary Tables S7–S10 and shown in Supplementary Figs S20 and S21, revealed close resemblance to the DRH_6_ model. Specifically, DRH_19_ exhibited a diameter of 16 Å, slightly larger than DRH_6_’s 15 Å but significantly smaller than the 25 Å diameter of the A-form hybrid. The minor groove of DRH_19_ measured 8.4 Å, slightly broader than the 8.3 Å of DRH_6_, yet still markedly narrower than the 9.5 Å minor groove of the A-form hybrid (Supplementary Tables S4, S11, and S12). These consistent structural features, observed across different sequences, strongly confirm the Z-form hybrid's conformation and its distinct characteristics relative to the A-form hybrid. Furthermore, the biofunctional properties linked to this Z-form conformation align with these structural observations, underscoring its unique role in biological processes.

Above results allow us to demonstrate the left-handed Z-form DNA-RNA hybrid is new double helix structure that is absolutely different from reported nuclei acid duplexes. So unique Z-hybrid structure could play significant biological function.

### Molecular dynamic models of Z-form and A-form hybrids with pol δ and PCNA

**Figure 6.**
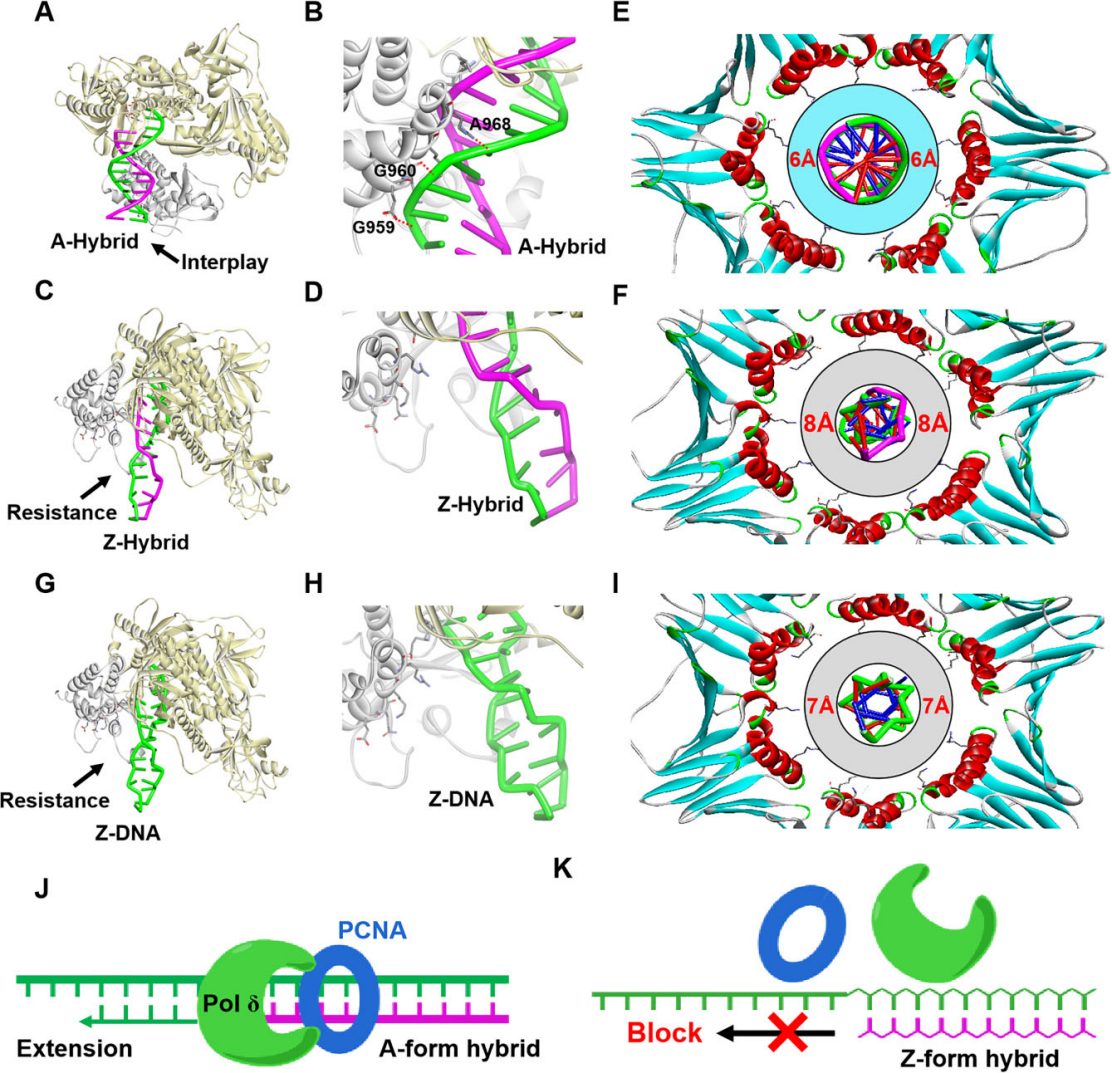
Comparison between the molecular models of A-form and Z-form hybrid-pol δ-PCNA complex. (**A**) A-form hybrid interacted with thumb of pol δ for launching DNA replication. (**B**) An amplification view from (**A**), showing thumb domain residues, G959, G960, and A968, stabilized DNA strand at minor groove of A-form hybrid by polar interaction, indicated by dashed red lines. (**C**) Z-form hybrid shows resistant effect to Pol δ due to a narrower and rigid minor groove. (**D**) An amplification view from (**C**), indicating the thumb cannot interact with Z-form hybrid. (**E**) Molecular dynamic simulation of PCNA and A-form hybrid interaction, the 6 Å circular cyan band marks the space between A-form hybrid and the inner surface of the PCNA ring, filling with a layer of water molecules. (**F**) Molecular dynamic simulation of PCNA and Z-form hybrid interaction, the 8 Å circular grey band marks the space between Z-form hybrid and the inner surface of the PCNA ring. (**G**) Z-form DNA shows resistant effect to Pol δ due to rigid minor groove. (**H**) An amplification view from (**G**), indicating the thumb cannot interact with Z-form DNA. (**I**) Molecular dynamic simulation of PCNA and Z-DNA interaction, the average 7 Å circular grey band marks the space between Z-DNA and the inner surface of the PCNA ring. (**J**) A-form hybrid allow a DNA replication initiation by associated with Pol δ and PCNA. (**K**) Z-form hybrid cannot interacts with Pol δ and PCNA, therefore blocking DNA replication.

Based on the Z-form hybrid structure, we built molecular dynamic models of the pol δ/Z-form hybrid complex and compared them with the pol δ/A-form hybrid complex. We observed that amino acid residues G959, G960, and A968 could contact the DNA strand in the minor groove of the A-form hybrid, stabilizing the pol δ/A-form hybrid complex (Fig. 6A and B). On the other hand, in the complex using Z-form hybrid, these residues are away from the DNA strand and lack interaction, preventing pol δ from localizing closely to the Z-form hybrid (Fig. 6C and D). This result is likely due to the structural features of the Z-form hybrid, which has a rigid backbone with a much narrower minor groove (8.3 Å) compared to the A-form hybrid's wide (9.5 Å) and flexible minor groove.

Another remarkable feature of the Z-form hybrid is its small duplex diameter (15 Å) compared to the A-form hybrid (25 Å) (Fig. 5C). This smaller hybrid diameter directly abolishes the Coulombic interaction between the PCNA clamp and the hybrid. The PCNA clamp plays a pivotal role in DNA replication initiation by activating pol δ during DNA polymerization. The PCNA clamp can encircle and slide on the hybrid via multiple polar residues by approaching the nucleic acid duplex phosphates within a Coulombic interaction distance (<6 Å) [20]. We found that the atomic distances between residues K80, N84, and R149 of PCNA and the phosphates in the A-form hybrid are smaller than 6 Å, allowing the formation of Coulombic interactions between the hybrid and the PCNA clamp (Supplementary Fig. S22A), as observed by the inner space filling with water molecules required for Coulombic force (Fig. 6E), therefore initiating and extending DNA sequence from RNA primer (Fig. 6J). Conversely, in the Z-form structure, all atomic distances exceed 8 Å, which beyond the Coulombic interaction range (Fig. 6F, Supplementary Fig. S22B), indicating that the Z-form hybrid cannot be encircled by the PCNA clamp, leading to the loss of DNA replication function (Fig. 6K).

To further validate that Z-form hybrid-induced DNA replication inhibition is attributable to its unique structural features, we compared the Z-form hybrid with the well-characterized Z-DNA structure. Z-DNA, known for its rigid minor groove (8.2 Å) and relatively small diameter (18 Å), was introduced into molecular dynamics studies to examine potential interactions with pol δ and PCNA. The results showed no interactions between the minor groove of Z-DNA and the thumb domain of pol δ, suggesting that Z-DNA cannot be recognized or used by pol δ as an effective substrate during DNA replication (Fig. 6G and H). This finding is consistent with earlier studies indicating that DNA polymerases lose the capacity to bind Z-DNA due to its rigid structure [54]. Furthermore, the results for Z-DNA–PCNA complexes demonstrated that the distance (>7 Å) between the phosphate backbone of Z-DNA and the inner surface of PCNA exceeds the threshold for effective coulombic interactions (<6 Å) (Fig. 6I and Supplementary Fig. S22C). This lack of interaction suggests that Z-DNA cannot be properly held by PCNA for efficient replication initiation [19]. These observations align with previous reports indicating that Z-DNA strongly blocks DNA replication due to its unique secondary structure [54]. They also support the conclusion that the structural rigidity and specific features of Z-form hybrids, similar to Z-DNA, render them ineffective in facilitating DNA replication reactions.

Consistently, an electrophoretic mobility shift assay shows that pol δ bind to the A-form hybrids DRH_2-2_ and DRH_4-4_, but do not bind to the Z-form hybrids DRH_1-1_ and DRH_3-3_ (Supplementary Fig. S23A and B). Identical results were also observed as A-form hybrids DRH_7-7_ allows to bind with pol δ and pol I, while it's not for Z-form hybrid DRH_6-6_ (Supplementary Fig. S23C and S23D). Additionally, a titration assay using molecular weight markers was conducted. The results showed that pol δ binds specifically to the A-form hybrids DRH_2-2_ and DRH_4-4_, as evidenced by the appearance of new bands with lower mobility at increasing ratios of pol δ to hybrid (1:0, 1:10, and 1:20) (Supplementary Figs. S24A and C). In contrast, no new bands were observed when pol δ was added to solutions containing Z-form hybrids DRH_1-1_ and DRH_3-3_, indicating no intermolecular interaction between them (Supplementary Figs S24B and D). Moreover, the dissociation constants (K_d_) also suggest a preferential binding of pol δ to the A-form hybrids, with nanomolar levels of measured K_d_ values as 28.9 nM to DRH_2-2_ and 25.8 nM to DRH_4-4_ respectively, whereas no K_d_ values could be detected for the Z-form DRH_1-1_ and DRH_3-3_ (Supplementary Table S13, Supplementary Fig. S25 and S26). Similarly, the K_d_ values were determined from using pol δ and pol I with A-form DRH_7-7_ as 38.2 and 22.6 nM, but no results when Z-form DRH_6-6_ was used (Supplementary Table S14, Supplementary Fig. S27 and S28). Exceptionally, the study of DNA-RNA hybrid binding with PCNA also was carried out. Results indicated that Z-form hybrids (e.g. DRH_1-1_ and DRH_3-3_) exhibited no detectable interaction with PCNA, whereas A-form hybrids (e.g. DRH_2-2_ and DRH_4-4_) demonstrated clear binding capacity with PCNA (Supplementary Fig. S29). Similarly, the titration assay using molecular weight markers showed that PCNA binds to the A-form hybrids (DRH_2-2_ and DRH_4-4_), but not to the Z-form hybrids (DRH_1-1_ and DRH_3-3_) (Supplementary Fig. S30). These findings are consistent with observations from dynamic simulation models.

## Discussion

This work demonstrated, for the first time, that the Z-form DNA-RNA hybrid stabilized by methylated CpG repeats blocks DNA replication. We discovered that the smaller helical diameter (15 Å) and much narrower minor groove (8.3 Å) of the Z-form hybrid limit its ability to interact with and localize to polymerase and PCNA, thereby affecting the initiation and elongation of Okazaki fragments. The results presented here provide new insights at the molecular level into how Z-form nucleic acids impact biological functions.

This study highlights the potential biological roles of the Z-hybrid, suggesting its possible influence on the transmission and evolution of complex genetic information, as well as its implications for pathogenesis and inheritance stability. Previous research has demonstrated that epigenetic modifications, such as the methylation of DNA sequences, can promote the formation of Z-form structures. Furthermore, extensive DNA methylation has been prominently observed in tumor suppressor genes, where it can induce gene silencing and the loss of cancer-inhibitory functions, ultimately contributing to oncogenesis [55–57].

DNA methylation has been extensively studied for its role in regulating genetic inheritance stability by precisely managing the temporal order of DNA replication [58–61]. These studies suggest that methylated DNA can result in replication timing heterogeneity and loss of allelic replication, possibly through the deregulation of DNA replication [62, 63]. The conclusions of this study provide further support for these findings, suggesting that methylated DNA can promote the formation of adaptable Z-form structures with RNA primers, regulating DNA replication patterns in a spatiotemporal manner, with significant implications for physiological function and disease pathogenesis.

Previous studies have shown that CG-rich DNA sequences are primarily distributed in origin regions during DNA replication [64–66]. Although only a limited number of studies explicitly demonstrate that CG-rich DNA templates can be directly recognized and utilized by DNA primase during replication [67], several lines of evidence suggest that these sequences play critical roles in DNA replication and related physiological processes. For instance, CG-rich regions have been implicated in regulating the timing of DNA replication [59], influencing DNA flexibility and fidelity [68], and contributing to genomic stability under specific conditions [69].

Currently, no direct evidence, based on our literature review, confirms the presence of protein-free RNA-DNA hybrids in Okazaki fragments within living tissues (e.g. in animals). However, multiple cellular-level studies highlight the existence and biological significance of RNA-DNA hybrids, particularly those associated with Okazaki fragments. For example, the DNA repair protein Ku has been shown to bind RNA-DNA hybrids, facilitating replication fork degradation in cells [70]. Additionally, RNase HIII promotes Okazaki fragment maturation by targeting RNA-DNA hybrids [71]. These findings suggest that RNA-DNA hybrids associated with Okazaki fragments serve as valid cellular models for exploring their functional significance, consistent with the approach of this study.

Under physiological conditions, Z-form hybrids can form and inhibit DNA replication through various mechanisms, including epigenetic modifications (e.g. DNA methylation), interactions with functional proteins (e.g. ZBP1), and environmental factors such as stress, salt concentration, and conformational torsion during chromosomal recombination [72–74]. To counteract these inhibitory effects and restore normal replication, Z-form hybrids can revert to the A-form through enzymatic processes like demethylation or by alleviating environmental stressors. This dynamic regulation ensures tight control of DNA replication timing in response to functional stimuli. Furthermore, the reversible transition between A- and Z-forms may influence genetic information, promoting inherited differentiation during development or tissue repair following injury. These findings highlight the biological significance of Z-form hybrids, not only as regulators of replication timing but also as contributors to genetic and epigenetic diversity.

Our studies have also highlighted the critical roles of Z-form nucleic acids, such as Z-DNA and Z-RNA, in regulating cellular processes, including the induction of necroptosis. For instance, Z-DNA has been shown to activate necroptosis pathways, leading to cell death [11], while viral Z-RNAs can trigger ZBP1-mediated necroptosis [10]. These findings suggest that Z-form nucleic acids, including Z-form hybrids with their characteristic left-handed helical conformation and zig-zag pattern, might activate similar pathways, contributing to cell cycle arrest, necroptosis, or other phenotypic outcomes.

In this study, we demonstrated that DNA methylation-induced Z-form hybrids could block DNA replication. During eukaryotic DNA replication on the lagging strand, multiple RNA primers synthesized by primase form Watson-Crick base pairs with template DNA, producing DNA-RNA hybrids [75–77]. It is plausible that these hybrids remain bound to primase in a complex state until the initiation of DNA synthesis by DNA polymerase. This raises the question of whether the DNA-RNA hybrid can still adopt the Z-form and block DNA replication while in complex with primase. Furthermore, various biological components involved in the regulation of DNA replication, such as signaling factors and active enzymes, may interact with the hybrid and influence the B-Z transition. Future studies focusing on these aspects will be essential to fully elucidate the physiological relevance of Z-form hybrids and their regulatory roles in DNA replication.

In summary, this work presents a new opportunity to understand how the Z-form DNA-RNA hybrid suppresses DNA replication and may be linked to a range of biological processes and diseases.

## Supplementary Material

gkaf135_Supplemental_File

## Data Availability

All NMR data were deposited in the online supplementary material as well as biological magnetic resonance bank under BMRB ID 52637. NMR coordinate of Z-form hybrid was deposited in PDB bank (code: 9JVN). Dynamic models of DRH_6_ are available in PDB bank (code: 9KZP, Amber) and ModelArchive (code: ma-waffc, Discovery Studio). The 3D model of hybrid with pol δ or PCNA are available in ModelArchive (accession code: ma-vwj9b).

## Supplementary data

Supplementary Data ia available at NAR online.

## References

[1] Rich A, Nordheim A, Wang A The chemistry and biology of left-handed Z-DNA. Annu Rev Biochem. 1984; 53:791–846. 10.1146/annurev.bi.53.070184.004043. 6383204

[2] Hall K, Cruz P, Tinoco Jr I et al. ‘Z-RNA’—a left-handed RNA double helix. Nature. 1984; 311:584–6. 10.1038/311584a0. 6482970

[3] McGinty R, Sunyaev S Revisiting mutagenesis at non-B DNA motifs in the human genome. Nat Struct Mol Biol. 2023; 30:417–24. 10.1038/s41594-023-00936-6. 36914796 PMC10225297

[4] Raiber E-A, Murat P, Chirgadze DY et al. 5-Formylcytosine alters the structure of the DNA double helix. Nat Struct Mol Biol. 2015; 22:44–9. 10.1038/nsmb.2936. 25504322 PMC4287393

[5] Wang G, Vasquez KM Dynamic alternative DNA structures in biology and disease. Nat Rev Genet. 2023; 24:211–34. 10.1038/s41576-022-00539-9. 36316397 PMC11634456

[6] Fang Y, Bansal K, Mostafavi S et al. AIRE relies on Z-DNA to flag gene targets for thymic T cell tolerization. Nature. 2024; 628:400–7. 10.1038/s41586-024-07169-7. 38480882 PMC11091860

[7] Herbert A Z-DNA and Z-RNA in human disease. Commun Biol. 2019; 2:7 10.1038/s42003-018-0237-x. 30729177 PMC6323056

[8] Wang SR, Wang JQ, Xu GH et al. The Cucurbit[7]Uril-based supramolecular chemistry for reversible B/Z-DNA transition. Adv Sci. 2018; 5:1800231 10.1002/advs.201800231. PMC6051393 30027051

[9] McKinney JA, Wang G, Mukherjee A et al. Distinct DNA repair pathways cause genomic instability at alternative DNA structures. Nat Commun. 2020; 11:236 10.1038/s41467-019-13878-9. 31932649 PMC6957503

[10] Zhang T, Yin C, Boyd DF et al. Influenza virus Z-RNAs induce ZBP1-mediated necroptosis. Cell. 2020; 180:1115–29. 10.1016/j.cell.2020.02.050. 32200799 PMC7153753

[11] Zhang T, Yin C, Fedorov A et al. ADAR1 masks the cancer immunotherapeutic promise of ZBP1-driven necroptosis. Nature. 2022; 606:594–602. 10.1038/s41586-022-04753-7. 35614224 PMC9373927

[12] Diallo MA, Pirotte S, Hu Y et al. A fish herpesvirus highlights functional diversities among Zα domains related to phase separation induction and A-to-Z conversion. Nucleic Acid Res. 2023; 51:806–30. 10.1093/nar/gkac761. 36130731 PMC9881149

[13] Vongsutilers V, Sawaspaiboontawee K, Tuesuwan B et al. 5-Methylcytosine containing CG decamer as Z-DNA embedded sequence for a potential Z-DNA binding protein probe. Nucleosides Nucleotides Nucleic Acids. 2018; 37:485–97. 10.1080/15257770.2018.1498512. 30188765

[14] Fujii S, Wang AH-J, van der Marel G et al. Molecular structure of (m^5^dC-dG)_3_: the role of the methyl group on 5-methyl cytosine in stabilizing Z-DNA. Nucleic Acids Res. 1982; 10:7879–92. 10.1093/nar/10.23.7879. 7155900 PMC327053

[15] Feigon J, Wang AH-J, van der Marel GA et al. A one-and two-dimensional NMR study of the B to Z transition of (m^5^dC-dG)_3_ in methanolic solution. Nucleic Acids Res. 1984; 12:1243–63. 10.1093/nar/12.2.1243. 6694910 PMC318570

[16] Ogawa T, Okazaki T Discontinuous DNA replication. Annu Rev Biochem. 1980; 49:421–57. 10.1146/annurev.bi.49.070180.002225. 6250445

[17] Xu B, Clayton DA RNA-DNA hybrid formation at the human mitochondrial heavy-strand origin ceases at replication start sites: an implication for RNA-DNA hybrids serving as primers. EMBO J. 1996; 15:3135–43. 10.1002/j.1460-2075.1996.tb00676.x. 8670814 PMC450256

[18] Itoh T, Tomizawa J Initiation of replication of plasmid ColE1 DNA by RNA polymerase, ribonuclease H, and DNA polymerase I. Cold Spring Harbor Symp Quant Biol. 1979; 43:409–17. 10.1101/SQB.1979.043.01.047. 225109

[19] Zheng F, Georgescu RE, Li H et al. Structure of eukaryotic DNA polymerase δ bound to the PCNA clamp while encircling DNA. Proc Natl Acad Sci USA. 2020; 117:30344–53. 10.1073/pnas.2017637117. 33203675 PMC7720213

[20] Lancey C, Tehseen M, Raducanu V-S et al. Structure of the processive human Pol δ holoenzyme. Nat Commun. 2020; 11:1109 10.1038/s41467-020-14898-6. 32111820 PMC7048817

[21] Niehrs C, Luke B Regulatory R-loops as facilitators of gene expression and genome stability. Nat Rev Mol Cell Biol. 2020; 21:167–78. 10.1038/s41580-019-0206-3. 32005969 PMC7116639

[22] Lu W-T, Hawley BR, Skalka GL et al. Drosha drives the formation of DNA: RNA hybrids around DNA break sites to facilitate DNA repair. Nat Commun. 2018; 9:532 10.1038/s41467-018-02893-x. 29416038 PMC5803274

[23] Noy A, Pérez A, Márquez M et al. Structure, recognition properties, and flexibility of the DNA.RNA hybrid. J Am Chem Soc. 2005; 127:4910–20. 10.1021/ja043293v. 15796556

[24] Cristini A, Groh M, Kristiansen MS et al. RNA/DNA hybrid interactome identifies DXH9 as a molecular player in transcriptional termination and R-loop-associated DNA damage. Cell Rep. 2018; 23:1891–905. 10.1016/j.celrep.2018.04.025. 29742442 PMC5976580

[25] Hamperl S, Cimprich KA The contribution of co-transcriptional RNA: DNA hybrid structures to DNA damage and genome instability. DNA Repair (Amst). 2014; 19:84–94. 10.1016/j.dnarep.2014.03.023. 24746923 PMC4051866

[26] Barroso S, Herrera-Moyano E, Muñoz S et al. The DNA damage response acts as a safeguard against harmful DNA–RNA hybrids of different origins. EMBO Rep. 2019; 20:e47250 10.15252/embr.201847250. 31338941 PMC6726908

[27] Sokol DL, Zhang X, Lu P et al. Real time detection of DNA RNA hybridization in living cells. Proc Natl Acad Sci USA. 1998; 95:11538–43. 10.1073/pnas.95.20.11538. 9751701 PMC21676

[28] Shaw NN, Arya DP Recognition of the unique structure of DNA: RNA hybrids. Biochimie. 2008; 90:1026–39. 10.1016/j.biochi.2008.04.011. 18486626

[29] Zhang C, Chen L, Peng D et al. METTL3 and N6-methyladenosine promote homologous recombination-mediated repair of DSBs by modulating DNA-RNA hybrid accumulation. Mol Cell. 2020; 79:425–42. 10.1016/j.molcel.2020.06.017. 32615088

[30] Singal R, Ginder GD DNA methylation. American Society of Hematology Washington. Am J Hematol. 1999; 93:4059–70. 10361102

[31] Stefansson OA, Sigurpalsdottir BD, Rognvaldsson S et al. The correlation between CpG methylation and gene expression is driven by sequence variants. Nat Genet. 2024; 56:1624–31. 10.1038/s41588-024-01851-2. 39048797 PMC11319203

[32] Noë M, Mathios D, Annapragada AV et al. DNA methylation and gene expression as determinants of genome-wide cell-free DNA fragmentation. Nat Commun. 2024; 15:6690 10.1038/s41467-024-50850-8. 39107309 PMC11303779

[33] Hubbard NW, Ames JM, Maurano M et al. ADAR1 mutation causes ZBP1-dependent immunopathology. Nature. 2022; 607:769–75. 10.1038/s41586-022-04896-7. 35859177 PMC9339495

[34] Jiao H, Wachsmuth L, Kumari S et al. Z-nucleic-acid sensing triggers ZBP1-dependent necroptosis and inflammation. Nature. 2020; 580:391–5. 10.1038/s41586-020-2129-8. 32296175 PMC7279955

[35] Berger I, Winston W, Manoharan R et al. Spectroscopic characterization of a DNA-binding domain, Zα, from the editing enzyme, dsRNA adenosine deaminase: evidence for left-handed Z-DNA in the Zα− DNA complex. Biochemistry. 1998; 37:13313–21. 10.1021/bi9813126. 9748339

[36] Bao H-L, Masuzawa T, Oyoshi T et al. Oligonucleotides DNA containing 8-trifluoromethyl-2′-deoxyguanosine for observing Z-DNA structure. Nucleic Acids Res. 2020; 48:7041–51. 32678885 10.1093/nar/gkaa505 PMC7367190

[37] Dai H, Liu J, Malkas LH et al. Characterization of RNA primers synthesized by the human breast cancer cell DNA synthesome. J Cell Biochem. 2009; 106:798–811. 10.1002/jcb.22015. 19204933 PMC2804863

[38] Popenda M, Milecki J, Adamiak RW High salt solution structure of a left-handed RNA double helix. Nucleic Acids Res. 2004; 32:4044–54. 10.1093/nar/gkh736. 15292450 PMC506817

[39] Darzynkiewicz Z, Sharpless T, Staiano-Coico L et al. Subcompartments of the G1 phase of cell cycle detected by flow cytometry. Proc Natl Acad Sci USA. 1980; 77:6696–9. 10.1073/pnas.77.11.6696. 6161370 PMC350355

[40] Chen G, Deng X Cell synchronization by double thymidine block. Bio-protocol. 2018; 8:e2994 10.21769/BioProtoc.2994. 30263905 PMC6156087

[41] Surani AA, Colombo SL, Barlow G et al. Optimizing cell synchronization using nocodazole or double thymidine block. Methods Mol Biol. 2021; 2329:111–21. 10.1007/978-1-0716-1538-6_9. 34085219

[42] Stöckl P, Hütter E, Zwerschke W et al. Sustained inhibition of oxidative phosphorylation impairs cell proliferation and induces premature senescence in human fibroblasts. Exp Gerontol. 2006; 41:674–82. 10.1016/j.exger.2006.04.009. 16713693

[43] Han YH, Moon HJ, You BR et al. p38 inhibitor intensified cell death in antimycin A-treated As4. 1 juxtaglomerular cells via the enhancement of GSH depletion. Anticancer Res. 2009; 29:4423–31. 20032388

[44] Han YH, Kim SH, Kim SZ et al. Antimycin A as a mitochondria damage agent induces an S phase arrest of the cell cycle in HeLa cells. Life Sci. 2008; 83:346–55. 10.1016/j.lfs.2008.06.023. 18655793

[45] Celis JE, Celis A Cell cycle-dependent variations in the distribution of the nuclear protein cyclin proliferating cell nuclear antigen in cultured cells: subdivision of S phase. Proc Natl Acad Sci USA. 1985; 82:3262–6. 10.1073/pnas.82.10.3262. 2860667 PMC397755

[46] Aizer A, Kafri P, Kalo A et al. The P body protein Dcp1a is hyper-phosphorylated during mitosis. PLoS One. 2013; 8:e49783 10.1371/journal.pone.0049783. 23300942 PMC3534667

[47] Sugiyama H, Kawai K, Matsunaga A et al. Synthesis, structure and thermodynamic properties of 8-methylguanine-containing oligonucleotides: Z-DNA under physiological salt conditions. Nucleic Acids Res. 1996; 24:1272–8. 10.1093/nar/24.7.1272. 8614630 PMC145791

[48] Balasubramaniyam T, Ishizuka T, Xiao C-D et al. 2’-*O*-Methyl-8-methylguanosine as a Z-Form RNA stabilizer for structural and functional study of Z-RNA. Molecules. 2018; 23:2572 10.3390/molecules23102572. 30304782 PMC6222775

[49] Song Y, Wang S, Xu Y Mirror-image RNA: a right-handed Z-Form RNA and its ligand complex. Molecules. 2024; 29:4900 10.3390/molecules29204900. 39459268 PMC11510240

[50] Bao H-L, Xu Y Investigation of higher-order RNA G-quadruplex structures in vitro and in living cells by ^19^F NMR spectroscopy. Nat Protoc. 2018; 13:652–65. 10.1038/nprot.2017.156. 29517770

[51] Wang S, Xu Y RNA structure promotes liquid-to-solid phase transition of short RNAs in neuronal dysfunction. Commun Biol. 2024; 7:137 10.1038/s42003-024-05828-z. 38287096 PMC10824717

[52] Wang S, Song Y, He Z et al. Unusual topological RNA G-quadruplex formed by an RNA duplex: implications for the dimerization of SARS-CoV-2 RNA. Chem Commun. 2023; 59:12703–6. 10.1039/D3CC03192F. 37819218

[53] Krall JB, Nichols PJ, Henen MA et al. Structure and formation of Z-DNA and Z-RNA. Molecules. 2023; 28:843 10.3390/molecules28020843. 36677900 PMC9867160

[54] Ramesh N, Shouche YS, Brahmachari SK Recognition of B and Z forms of DNA by Escherichia coli DNA polymerase I. J Mol Biol. 1986; 190:635–8. 10.1016/0022-2836(86)90248-2. 3537317

[55] Berman BP, Weisenberger DJ, Aman JF et al. Regions of focal DNA hypermethylation and long-range hypomethylation in colorectal cancer coincide with nuclear lamina–associated domains. Nat Genet. 2012; 44:40–6. 10.1038/ng.969. PMC4309644 22120008

[56] Fahrner JA, Eguchi S, Herman JG et al. Dependence of histone modifications and gene expression on DNA hypermethylation in cancer. Cancer Res. 2002; 62:7213–8. 12499261

[57] Klusmann I, Wohlberedt K, Magerhans A et al. Chromatin modifiers Mdm2 and RNF2 prevent RNA: DNA hybrids that impair DNA replication. Proc Natl Acad Sci USA. 2018; 115:E11311–20. 10.1073/pnas.1809592115. 30413623 PMC6275510

[58] Takebayashi S-i, Ryba T, Wimbish K et al. The temporal order of DNA replication shaped by mammalian DNA methyltransferases. Cells. 2021; 10:266 10.3390/cells10020266. 33572832 PMC7911666

[59] Selig S, Ariel M, Goitein R et al. Regulation of mouse satellite DNA replication time. EMBO J. 1988; 7:419–26. 10.1002/j.1460-2075.1988.tb02829.x. 3366119 PMC454336

[60] Donley N, Thayer MJ DNA replication timing, genome stability and cancer: late and/or delayed DNA replication timing is associated with increased genomic instability. Semin Cancer Biol. 2013; 23:80–9. 10.1016/j.semcancer.2013.01.001. 23327985 PMC3615080

[61] Du Q, Smith GC, Luu PL et al. DNA methylation is required to maintain both DNA replication timing precision and 3D genome organization integrity. Cell Rep. 2021; 36:109722 10.1016/j.celrep.2021.109722. 34551299

[62] Endicott JL, Nolte PA, Shen H et al. Cell division drives DNA methylation loss in late-replicating domains in primary human cells. Nat Commun. 2022; 13:6659 10.1038/s41467-022-34268-8. 36347867 PMC9643452

[63] Charlton J, Downing TL, Smith ZD et al. Global delay in nascent strand DNA methylation. Nat Struct Mol Biol. 2018; 25:327–32. 10.1038/s41594-018-0046-4. 29531288 PMC5889353

[64] Job D, Marmillot P, Job C et al. Transcription of left-handed Z-DNA templates: increased rate of single-step addition reactions catalyzed by wheat germ RNA polymerase II. Biochemistry. 1988; 27:6371–8. 10.1021/bi00417a027. 3219341

[65] Rein T, Kobayashi T, Malott M et al. DNA methylation at mammalian replication origins. J Biol Chem. 1999; 274:25792–800. 10464318 10.1074/jbc.274.36.25792

[66] Araujo FD, Knox JD, Szyf M et al. Concurrent replication and methylation at mammalian origins of replication. Mol Cell Biol. 1998; 18:3475–82. 10.1128/MCB.18.6.3475. 9584187 PMC108928

[67] Larson MA, Bressani R, Sayood K et al. Hyperthermophilic Aquifex aeolicus initiates primer synthesis on a limited set of trinucleotides comprised of cytosines and guanines. Nucleic Acids Res. 2008; 36:5260–9. 10.1093/nar/gkn461. 18684998 PMC2532735

[68] Ushijima T, Watanabe N, Shimizu K et al. Decreased fidelity in replicating CpG methylation patterns in cancer cells. Cancer Res. 2005; 65:11–7. 10.1158/0008-5472.11.65.1. 15665274

[69] Nathan D, Crothers DM Bending and flexibility of methylated and unmethylated EcoRI DNA. J Mol Biol. 2002; 316:7–17. 10.1006/jmbi.2001.5247. 11829499

[70] Audoynaud C, Schirmeisen K, Saada AA et al. RNA: DNA hybrids from Okazaki fragments contribute to establish the Ku-mediated barrier to replication-fork degradation. Mol Cell. 2023; 83:1061–74. 10.1016/j.molcel.2023.02.008. 36868227

[71] Randall JR, Nye TM, Wozniak KJ et al. RNase HIII is important for Okazaki fragment processing in Bacillus subtilis. J Bacteriol. 2019; 201:e00686-18 10.1128/JB.00686-18. 30670546 PMC6416905

[72] Preisler RS, Chen HH, Colombo MF et al. The B form to Z form transition of poly (dG-m5dC) is sensitive to neutral solutes through an osmotic stress. Biochemistry. 1995; 34:14400–7. 10.1021/bi00044a017. 7578044

[73] Azorin F, Nordheim A, Rich A Formation of Z-DNA in negatively supercoiled plasmids is sensitive to small changes in salt concentration within the physiological range. EMBO J. 1983; 2:649–55. 10.1002/j.1460-2075.1983.tb01479.x. 6315414 PMC555164

[74] Kim SH, Jung HJ, Lee I-B et al. Sequence-dependent cost for Z-form shapes the torsion-driven B-Z transition via close interplay of Z-DNA and DNA bubble. Nucleic Acids Res. 2021; 49:3651–60. 10.1093/nar/gkab153. 33744929 PMC8053131

[75] Masai H, Matsumoto S, You Z et al. Eukaryotic chromosome DNA replication: where, when, and how?. Annu Rev Biochem. 2010; 79:89–130. 10.1146/annurev.biochem.052308.103205. 20373915

[76] Masai H, Arai K Frpo: A novel single-stranded DNA promoter for transcription and for primer RNA synthesis of DNA replication. Cell. 1997; 89:897–907. 10.1016/S0092-8674(00)80275-5. 9200608

[77] Masai H, Arai K Mechanisms of primer RNA synthesis and D-loop/R-loop-dependent DNA replication in *Escherichia co**li*. Biochimie. 1996; 78:1109–17. 10.1016/S0300-9084(97)86737-5. 9150892

