# Supplementary material for: Z-form DNA-RNA hybrid blocks DNA replication
Source: Nucleic Acids Res. 2025 Mar 4;53(5):gkaf135. doi: 10.1093/nar/gkaf135 (PMC11879439; doi:10.1093/nar/gkaf135)
Supplement: gkaf135_Supplemental_File [file gkaf135_supplemental_file.pdf]

Z-form DNA-RNA hybrid blocks DNA replication

Shiyu Wang and Yan Xu*

Table of contents

Supplemental Tables 2

Supplemental Figures 10

Supplemental Schemes 45

Supplemental texts 46

Supplemental Data 51

Table S1. ^{1}H NMR chemical shifts δ_H (p.p.m.) of DRH_6 in the Z-form hybrid at 293 K in D_2O^a.

Residue	H5/Me	H8/6/2	H1′	H2′	H2″	H3′	H4′	H5′	H5″	H1	NH_2
$d^{m}C_1$	2.46	7.53	5.72	1.27	2.32	4.63	3.41	2.49	3.09	—	N/A
$d^{F}G_2$	—	—	5.79	4.08	N/A	4.74	4.22	4.00	4.05	12.94	N/A
$d^{m}C_3$	2.46	7.13	5.54	1.46	2.48	4.69	3.81	2.52	3.41	—	8.13<br>6.39
dA_4	—	7.06 (H8)<br>7.23 (H2)	5.56	5.08	3.10	4.66	4.00	3.62	3.72	—	N/A
$d^{m}C_5$	2.53	7.23	5.80	1.35	2.28	4.49	3.97	2.31	2.99	—	8.24<br>6.43
$d^{F}G_6$	—	—	5.76	4.03	N/A	4.74	3.88	3.79	3.96	12.98	N/A
$d^{m}C_7$	2.45	6.91	5.55	1.59	2.44	4.94	3.97	3.01	3.41	—	8.12<br>6.47
dG_8	—	7.99	5.92	2.65	2.65	4.68	4.05	3.65	3.96	12.76	6.21<br>($NH2_2$)
rC_1	5.08	7.06	5.78	3.98	—	4.69	3.84	3.71	3.39	—	8.44<br>6.51
$r^{F}G_2$	—	—	5.73	4.62	—	5.44	N/A	3.84	3.35	12.97	8.36<br>6.41
rC_3	5.04	7.13	5.56	4.04	—	4.70	4.29	3.77	3.42	—	8.22<br>6.71
$r^{F}G_4$	—	—	6.20	4.06	—	5.06	4.60	3.79	3.69	12.96	N/A
rU_5	5.53	7.53	5.68	4.00	—	4.20	4.02	3.82	3.41	12.94	—
$r^{F}G_6$	—	—	5.68	4.57	—	5.13	4.56	3.69	3.42	12.98	N/A
rC_7	5.04	7.29	5.84	3.93	—	4.56	3.63	3.82	3.60	—	8.24<br>6.20
rG_8	—	7.92	5.90	4.22	—	4.72	4.12	3.89	3.85	12.90	8.30<br>6.43

[a]N/A is "not applicable", indicating these proton signals could not be confirmed.

Table S2. Backbone angles[a] for 10 conformers representing the refined structure of DRH_6 in the Z-form and reference X-ray Z-DNA structures[b] of $d(CGCGCG)_2$ (right column in DNA strand) and Z-RNA structures[c] of $r(CGCGCG)_2$ (right column in RNA strand). These molecular models were generated by dynamic simulation using BIOVIA Discovery Studio 4.5.

Residue	α	β	γ	δ	ε	ζ	χ
d^mC_1	— —	— —	52(2) 39(38)	-119(2) 143(6)	58(4) -93(4)	-52(2) 75(5)	-168(6) -165(4)
d^FG_2	105(3) 65(5)	161(2) -172(3)	-163(3) 177(3)	-132(6) 95(7)	-129(6) -117(7)[d] -161(5)[e]	-49(2) -64(17)[d] 59(8)[e]	66(3) 63(2)
d^mC_3	-145(5) -150(9)[d] 165(5)[e]	-127(5) -132(5)[d] 155(6)[e]	47(2) 54(4)	-95(2) 147(4)	-74(6) -95(5)	85(2) 75(4)	-168(4) -163(2)
dA_4	69(3) 66(5)	170(2) -174(6)	161(5) 179(3)	-136(4) 95(5)	-185(3) -123(9)[d] -176(4)[e]	-9(1) -55(16)[d] 67(5)[e]	66(1) 65(4)
d^mC_5	-154(11) -155(8)[d] 169(3)[e]	-155(6) -133(2)[d] 163(5)[e]	51(1) 49(7)	-110(4) 141(5)	-124(3) -95(4)	104(5) 70(7)	-154(7) -152(3)
d^FG_6	52(3) 79(7)	-141(7) -179(5)	180(4) 176(3)	-141(6) 148(5)	-149(3) —	-12(1) —	70(3) 64(5)
d^mC_7	-158(7) —	-186(6) —	51(3) —	-99(3) —	-97(5) —	89(2) —	-168(4) —
dG_8	62(0) —	-164(4) —	-179(6) —	-112(5) —	— —	— —	61(4) —
rC_1	— —	— —	74(2) 46(2)	-85(3) 153(1)	8(0) -68(1)	-39(3) 76(2)	-162(6) -151(2)
r^FG_2	105(4) 49(4)	111(4) -163(2)	-147(7) -175(3)	-115(3) 81(1)	-152(6) -104(1)	-108(5) -55(2)	101(2) 60(2)
rC_3	-155(4) -145(2)	-112(3) 175(4)	79(4) 71(1)	-114(5) 127(2)	21(1) -72(1)	-48(5) 76(2)	-159(4) -159(2)
r^FG_4	-139(6) 69(3)	160(5) -159(1)	161(4) 172(1)	-121(5) 86(2)	-118(3) -105(1)	-77(2) -65(3)	79(3) 61(2)
rU_5	-169(5) -134(4)	-150(7) -158(3)	102(4) 54(4)	-85(3) 146(1)	147(3) -83(4)	-135(8) 71(1)	180(5) -141(2)
r^FG_6	96(3) 62(2)	-131(5) -165(1)	-138(5) -173(1)	-141(5) 84(1)	-168(6) —	-28(2) —	68(4) 76(2)
rC_7	-168(11) —	-140(8) —	65(2) —	-123(7) —	127(5) —	-96(4) —	-149(9) —
rG_8	-21(0) —	-92(2) —	-111(5) —	-94(4) —	— —	— —	72(2) —

[a]P-α-O5′-β-C5′-γ-C4′-δ-C3′-ε-O3′-ζ-P; average values with standard deviations in parentheses are given.

[b]PDB ID: 131D, 1D39, 1D48, 1DCG, 1DJ6, 1I0T, 1ICK, 292D, 293D, 2DCG, 336D.

[c]PDB ID: 1T4X.

[d]GpC step typical of the Z_I-DNA form.

[e]GpC step typical of the Z_{II}-DNA form.

Table S3. Selected helical parameters for 10 conformers representing the refined structure of DRH_6 in the Z-form. These molecular models were generated by dynamic simulation using BIOVIA Discovery Studio 4.5.

Base steps	Shift [Å]	Slide [Å]	Rise [Å]	Tilt [°]	Roll [°]	Twist [°]
d^mC_1:rG_8—d^FG_2:rC_7	-1.1 (0.1)	4.8 (0.2)	3.5 (0.2)	4.1 (2.1)	10.7 (0.4)	16.6 (0.6)
d^FG_2:rC_7—d^mC_3:r^FG_6	-0.6 (0.1)	-1.1 (0.1)	3.0 (0.1)	4.2 (1.0)	-14.6 (0.2)	45.7 (0.7)
d^mC_3:r^FG_6—dA_4:rU_5	0.43 (0.1)	6.2 (0.3)	3.9 (0.2)	7.3 (1.6)	7.7 (0.1)	32.2 (1.9)
dA_4:rU_5—d^mC_5:r^FG_4	-0.1 (0.1)	-0.9 (0.1)	3.0 (0.3)	-7.0 (1.2)	-21.0 (0.3)	52.0 (2.4)
d^mC_5:r^FG_4—d^FG_6:rC_3	-0.6 (0.1)	6.1 (0.2)	3.3 (0.1)	1.2 (0.2)	11.5 (0.4)	27.9 (1.7)
d^FG_6:rC_3—d^mC_7:r^FG_2	0.3 (0.1)	-1.3 (0.3)	2.9 (0.2)	-5.9 (1.1)	-15.6 (0.0)	43.6 (2.6)
d^mC_7:r^FG_2—dG_8:rC_1	1.4 (0.3)	5.3 (0.3)	3.4 (0.3)	0.4 (0.3)	7.5 (0.1)	20.6 (1.2)

Table S4. Comparison of Z-form DRH_6 structure in this study and authentic A-form hybrid.

Duplex	Z-form hybrid	A-form hybrid
Helical sense	Left-handed	Left-handed
Residues per turn	11.2	11.8
Major groove width (Å)	—	3.9
Minor groove width (Å)	8.3	9.5
Rise (Å)	3.3	2.9
Twist (°)	34.1	33.7
Helical tilt (°)	16.8	13.9
Diameter (Å)	15.0	> 25.0
Inclination (°)	13.6	10.5
Glycosidic bond	*Syn*-purine<br>*Anti*-pyrimidine	*Anti-*
Sugar Pucker	C3′-*endo* purine<br>C2′-*endo* pyrimidine	Major C3′-*endo*

Table S5. Backbone angles[a] for 10 conformers representing the refined structure of DRH_6 in the Z-form. These molecular models were generated by dynamic simulation using Amber 18.

Residue	α	β	γ	δ	ε	ζ	χ
$d^{m}C_1$	—	—	56(2)	-124(2)	55(4)	-51(2)	-164(6)
$d^{F}G_2$	107(2)	160(3)	-162(5)	-135(4)	-130(5)	-52(2)	65(3)
$d^{m}C_3$	-148(3)	-129(4)	48(2)	-99(1)	-75(5)	84(4)	-170(5)
dA_4	71(4)	172(3)	162(5)	-138(4)	-182(3)	-11(1)	62(1)
$d^{m}C_5$	-155(11)	-153(5)	54(3)	-112(5)	-126(1)	105(5)	-154(7)
$d^{F}G_6$	50(2)	-140(8)	182(6)	-142(4)	-150(3)	-16(1)	72(3)
$d^{m}C_7$	-159(7)	-184(6)	53(5)	-101(2)	-99(5)	86(2)	-163(4)
dG_8	60(0)	-162(6)	-177(6)	-115(5)	—	—	63(4)
rC_1	—	—	75(2)	-86(3)	10(0)	-39(3)	-163(6)
$r^{F}G_2$	106(5)	113(2)	-148(9)	-112(3)	-154(6)	-110(4)	103(3)
rC_3	-156(4)	-115(3)	78(4)	-112(3)	22(0)	-49(7)	-160(5)
$r^{F}G_4$	-140(7)	162(3)	163(5)	-120(3)	-119(3)	-75(2)	77(3)
rU_5	-166(5)	-152(7)	104(6)	-86(3)	148(3)	-133(8)	172(7)
$r^{F}G_6$	97(3)	-133(5)	-135(7)	-140(4)	-169(8)	-27(1)	65(5)
rC_7	-164(8)	-142(7)	68(3)	-125(6)	126(3)	-98(5)	-147(6)
rG_8	-20(2)	-94(3)	-113(6)	-95(7)	—	—	71(2)

[a]P-α-O5′-β-C5′-γ-C4′-δ-C3′-ε-O3′-ζ-P; average values with standard deviations in parentheses are given.

Table S6. Selected helical parameters for 10 conformers representing the refined structure of DRH_6 in the Z-form. These molecular models were generated by dynamic simulation using Amber 18.

Base steps	Shift [Å]	Slide [Å]	Rise [Å]	Tilt [°]	Roll [°]	Twist [°]
$d^{m}C_1$:rG_8—$d^{F}G_2$:rC_7	-1.0 (0.1)	4.9 (0.2)	3.4 (0.2)	4.1 (2.1)	10.5 (0.4)	16.6 (0.6)
$d^{F}G_2$:rC_7—$d^{m}C_3$:$r^{F}G_6$	-0.7 (0.1)	-1.2 (0.1)	3.0 (0.1)	4.3 (1.0)	-14.4 (0.2)	45.2 (0.7)
$d^{m}C_3$:$r^{F}G_6$—dA_4:rU_5	0.5 (0.1)	6.3 (0.3)	3.6 (0.2)	7.4 (1.6)	7.8 (0.1)	31.6 (1.4)
dA_4:rU_5—$d^{m}C_5$:$r^{F}G_4$	-0.2 (0.1)	-1.0 (0.2)	3.3 (0.2)	-7.0 (1.2)	-21.2 (0.3)	52.1 (2.3)
$d^{m}C_5$:$r^{F}G_4$—$d^{F}G_6$:rC_3	-0.6 (0.1)	6.2 (0.2)	3.3 (0.3)	1.2 (0.2)	11.3 (0.6)	27.9 (1.9)
$d^{F}G_6$:rC_3—$d^{m}C_7$:$r^{F}G_2$	0.3 (0.1)	-1.4 (0.3)	2.9 (0.2)	-6.0 (1.1)	-15.8 (0.0)	43.4 (2.2)
$d^{m}C_7$:$r^{F}G_2$—dG_8:rC_1	1.3 (0.2)	5.5 (0.3)	3.5 (0.3)	0.4 (0.3)	7.3 (0.1)	20.1 (1.5)

Table S7. Backbone angles[a] for 10 conformers representing the refined structure of DRH$_{19}$ in the Z-form. These molecular models were generated by dynamic simulation using BIOVIA Discovery Studio 4.5.

Residue	α	β	γ	δ	ε	ζ	χ
d^{m}C$_1$	—	—	64(1)	-109(3)	50(5)	-58(4)	-166(3)
d^{F}G$_2$	102(6)	165(7)	-160(3)	-121(7)	-137(2)	-64(2)	67(2)
d^{m}C$_3$	-140(2)	-139(5)	55(4)	-105(3)	-59(4)	67(5)	-165(4)
dG$_4$	79(3)	162(4)	151(3)	-147(5)	-165(4)	-19(3)	60(2)
d^{m}C$_5$	-168(5)	-136(7)	59(6)	-101(4)	-118(2)	98(4)	-168(6)
d^{F}G$_6$	42(3)	-156(9)	174(6)	-135(6)	-132(5)	-28(2)	57(6)
d^{m}C$_7$	-140(3)	-199(8)	68(5)	-114(6)	-82(7)	97(3)	-154(8)
dG$_8$	48(3)	-180(3)	-162(5)	-124(3)	—	—	60(5)
rC$_1$	—	—	87(4)	-75(5)	21(2)	-48(3)	-175(7)
r^{F}G$_2$	112(3)	119(4)	-135(7)	-129(6)	-141(3)	-128(3)	89(6)
rC$_3$	-173(4)	-101(5)	91(8)	-127(5)	30(2)	-57(3)	-133(6)
r^{F}G$_4$	-127(4)	143(4)	138(4)	-147(4)	-108(3)	-57(1)	92(5)
rC$_5$	-172(4)	-144(6)	118(5)	-81(5)	160(6)	-128(4)	177(4)
r^{F}G$_6$	105(6)	-126(8)	-140(6)	-122(6)	-137(5)	-41(2)	48(3)
rC$_7$	-155(5)	-130(7)	75(6)	-131(8)	110(6)	-79(4)	-158(8)
rG$_8$	-21(4)	-77(6)	-119(5)	-118(8)	—	—	65(3)

[a]P-α-O5′-β-C5′-γ-C4′-δ-C3′-ε-O3′-ζ-P; average values with standard deviations in parentheses are given.

Table S8. Selected helical parameters for 10 conformers representing the refined structure of DRH$_{19}$ in the Z-form. These molecular models were generated by dynamic simulation using BIOVIA Discovery Studio 4.5.

Base steps	Shift [Å]	Slide [Å]	Rise [Å]	Tilt [°]	Roll [°]	Twist [°]
d^{m}C$_1$:rG$_8$—d^{F}G$_2$:rC$_7$	-0.5 (0.2)	5.6 (0.2)	3.6 (0.1)	4.8 (1.2)	11.7 (0.3)	17.6 (1.9)
d^{F}G$_2$:rC$_7$—d^{m}C$_3$:r^{F}G$_6$	-1.2 (0.1)	-2.8 (0.1)	3.3 (0.1)	3.9 (0.8)	-12.5 (0.3)	42.4 (1.1)
d^{m}C$_3$:r^{F}G$_6$—dG$_4$:rC$_5$	0.8 (0.1)	4.8 (0.3)	3.5 (0.1)	8.7 (1.3)	9.2 (0.2)	28.8 (1.3)
dG$_4$:rC$_5$—d^{m}C$_5$:r^{F}G$_4$	-0.6 (0.1)	-1.9 (0.2)	3.2 (0.1)	-4.7 (0.9)	-11.4 (0.2)	48.7 (2.9)
d^{m}C$_5$:r^{F}G$_4$—d^{F}G$_6$:rC$_3$	-0.1 (0.1)	4.5 (0.2)	3.3 (0.2)	2.7 (0.2)	15.7 (0.3)	26.8 (1.4)
d^{F}G$_6$:rC$_3$—d^{m}C$_7$:r^{F}G$_2$	0.7 (0.1)	-2.6 (0.3)	3.1 (0.1)	-4.4 (1.8)	-14.0 (0.1)	35.9 (1.6)
d^{m}C$_7$:r^{F}G$_2$—dG$_8$:rC$_1$	1.6 (0.2)	7.0 (0.3)	3.3 (0.2)	1.9 (0.6)	9.2 (0.1)	23.7 (3.1)

Table S9. Backbone angles[a] for 10 conformers representing the refined structure of DRH19 in the Z-form. These molecular models were generated by dynamic simulation using Amber 18.

Residue	α	β	γ	δ	ε	ζ	χ
d^mC_1	—	—	68(2)	-105(4)	58(3)	-62(2)	-162(4)
d^FG_2	100(3)	169(6)	-157(4)	-126(9)	-134(4)	-62(4)	69(3)
d^mC_3	-133(3)	-130(4)	58(2)	-108(6)	-61(5)	62(3)	-161(5)
dG_4	80(6)	160(6)	162(4)	-142(3)	-167(3)	-26(4)	57(3)
d^mC_5	-176(4)	-129(4)	70(4)	-110(3)	-127(4)	103(3)	-161(2)
d^FG_6	49(5)	-147(7)	162(4)	-140(8)	-141(2)	-39(6)	59(5)
d^mC_7	-137(6)	-204(11)	59(8)	-121(8)	-86(5)	110(4)	-136(9)
dG_8	39(4)	-162(6)	-154(6)	-118(5)	—	—	57(4)
rC_1	—	—	79(3)	-66(4)	27(4)	-60(5)	-169(6)
r^FG_2	109(4)	125(6)	-124(6)	-138(5)	-136(5)	-122(4)	96(4)
rC_3	-171(2)	-99(4)	82(6)	-119(6)	26(3)	-51(4)	-140(7)
r^FG_4	-134(5)	156(6)	145(5)	-152(7)	-103(7)	-63(2)	98(8)
rC_5	-182(5)	-157(8)	130(6)	-90(8)	142(7)	-137(6)	162(3)
r^FG_6	115(8)	-134(5)	-163(7)	-127(4)	-143(6)	-53(3)	62(4)
rC_7	-140(7)	-125(6)	69(4)	-122(6)	99(4)	-86(3)	-151(6)
rG_8	-26(3)	-69(5)	-117(3)	-127(6)	—	—	60(5)

[a]P-α-O5′-β-C5′-γ-C4′-δ-C3′-ε-O3′-ζ-P; average values with standard deviations in parentheses are given.

Table S10. Selected helical parameters for 10 conformers representing the refined structure of DRH19 in the Z-form. These molecular models were generated by dynamic simulation using Amber 18.

Base steps	Shift [Å]	Slide [Å]	Rise [Å]	Tilt [°]	Roll [°]	Twist [°]
d^mC_1:rG_8—d^FG_2:rC_7	-0.3 (0.1)	5.5 (0.3)	3.7 (0.1)	4.6 (1.5)	12.5 (0.5)	14.2 (1.8)
d^FG_2:rC_7—d^mC_3:r^FG_6	-1.6 (0.2)	-2.9 (0.2)	3.2 (0.1)	3.6 (0.6)	-10.8 (0.6)	38.5 (1.0)
d^mC_3:r^FG_6—dG_4:rC_5	0.7 (0.1)	4.5 (0.2)	3.5 (0.1)	7.5 (1.1)	9.4 (0.3)	26.4 (0.9)
dG_4:rC_5—d^mC_5:r^FG_4	-0.6 (0.1)	-2.2 (0.1)	3.2 (0.1)	-5.2 (0.8)	-13.0 (0.3)	47.2 (3.1)
d^mC_5:r^FG_4—d^FG_6:rC_3	-0.1 (0.1)	4.3 (0.3)	3.5 (0.2)	2.9 (0.1)	14.6 (0.2)	33.2 (2.0)
d^FG_6:rC_3—d^mC_7:r^FG_2	0.6 (0.1)	-2.8 (0.5)	3.0 (0.1)	-5.1 (1.4)	-11.9 (0.2)	41.5 (1.4)
d^mC_7:r^FG_2—dG_8:rC_1	1.5 (0.3)	6.6 (0.4)	3.2 (0.2)	1.6 (0.7)	9.7 (0.2)	23.1 (2.6)

Table S11. Comparison of diameter and minor groove between Z-form hybrid of DRH_6 and DRH_{19} using different molecular dynamic simulation tools as BIOVIA Discovery Studio 4.5 and Amber 18. Ribbon view of Z-form hybrid shows green color in DNA strand as well as pink color from RNA strand, in which diameters were labeled by red circles and minor groove were marked by blue arrows.

Name	DRH_6	DRH_6	DRH_{19}	DRH_{19}
**Sequence**	5'-d(CGCACGCG)-3'<br>3'-r(GCGUGCGC)-5'	5'-d(CGCACGCG)-3'<br>3'-r(GCGUGCGC)-5'	5'-d(CGCGCGCG)-3'<br>3'-r(GCGCGCGC)-5'	5'-d(CGCGCGCG)-3'<br>3'-r(GCGCGCGC)-5'
**Molecular dynamic simulation tool**	BIOVIA Discovery Studio 4.5	Amber 18	BIOVIA Discovery Studio 4.5	Amber 18
**Diameter in Z-form hybrid model**	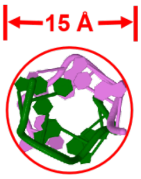	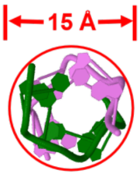	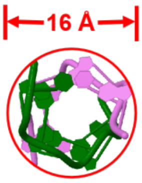	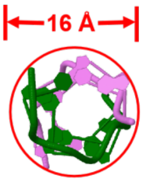
**Minor groove in Z-form hybrid model**	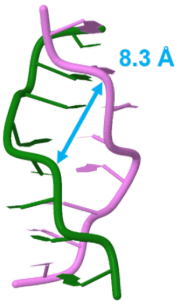	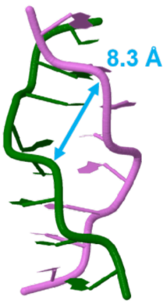	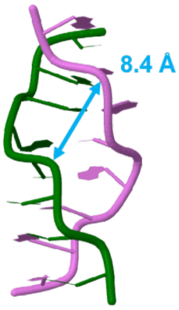	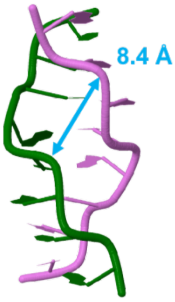

Table S12. Comparison of major structural parameters between Z-form hybrid of DRH_6 and DRH_{19} using different molecular dynamic simulation tools as BIOVIA Discovery Studio 4.5 and Amber 18.

Duplex name	DRH_6	DRH_6	DRH_{19}	DRH_{19}
Sequence	5'-d(CGCACGCG)-3'<br>3'-r(GCGUGCGC)-5'	5'-d(CGCACGCG)-3'<br>3'-r(GCGUGCGC)-5'	5'-d(CGCGCGCG)-3'<br>3'-r(GCGCGCGC)-5'	5'-d(CGCGCGCG)-3'<br>3'-r(GCGCGCGC)-5'
Molecular dynamic simulation tool	BIOVIA Discovery Studio 4.5	Amber 18	BIOVIA Discovery Studio 4.5	Amber 18
Helical sense	Left-handed	Left-handed	Left-handed	Left-handed
Residues per turn	11.2	11.2	11.5	11.5
Major groove width (Å)	—	—	—	—
Minor groove width (Å)	8.3	8.3	8.4	8.4
Rise (Å)	3.3	3.3	3.3	3.3
Twist (°)	34.1	33.9	32.0	31.9
Helical tilt (°)	16.8	16.8	16.5	16.5
Diameter (Å)	15.0	15.0	16.0	16.0
Inclination (°)	13.6	13.6	13.3	13.3
Glycosidic bond	*Syn*-purine<br>*Anti*-pyrimidine	*Syn*-purine<br>*Anti*-pyrimidine	*Syn*-purine<br>*Anti*-pyrimidine	*Syn*-purine<br>*Anti*-pyrimidine
Sugar Pucker	C3'-*endo* purine<br>C2'-*endo* pyrimidine	C3'-*endo* purine<br>C2'-*endo* pyrimidine	C3'-*endo* purine<br>C2'-*endo* pyrimidine	C3'-*endo* purine<br>C2'-*endo* pyrimidine

Table S13. Equilibrium dissociation constant (K_d) of pol δ to DNA-RNA hybrids.

DNA-RNA hybrids	DRH_{1-1}	DRH_{2-2}	DRH_{3-3}	DRH_{4-4}
K_d (nM)	N/A	28.9	N/A	25.8

Table S14. Equilibrium dissociation constant (K_d) of pol δ and pol I to DNA-RNA hybrids.

DNA-RNA hybrids	DRH_{6-6} + pol δ	DRH_{6-6} + pol I	DRH_{7-7} + pol δ	DRH_{7-7} + pol I
K_d (nM)	N/A	N/A	38.2	22.6

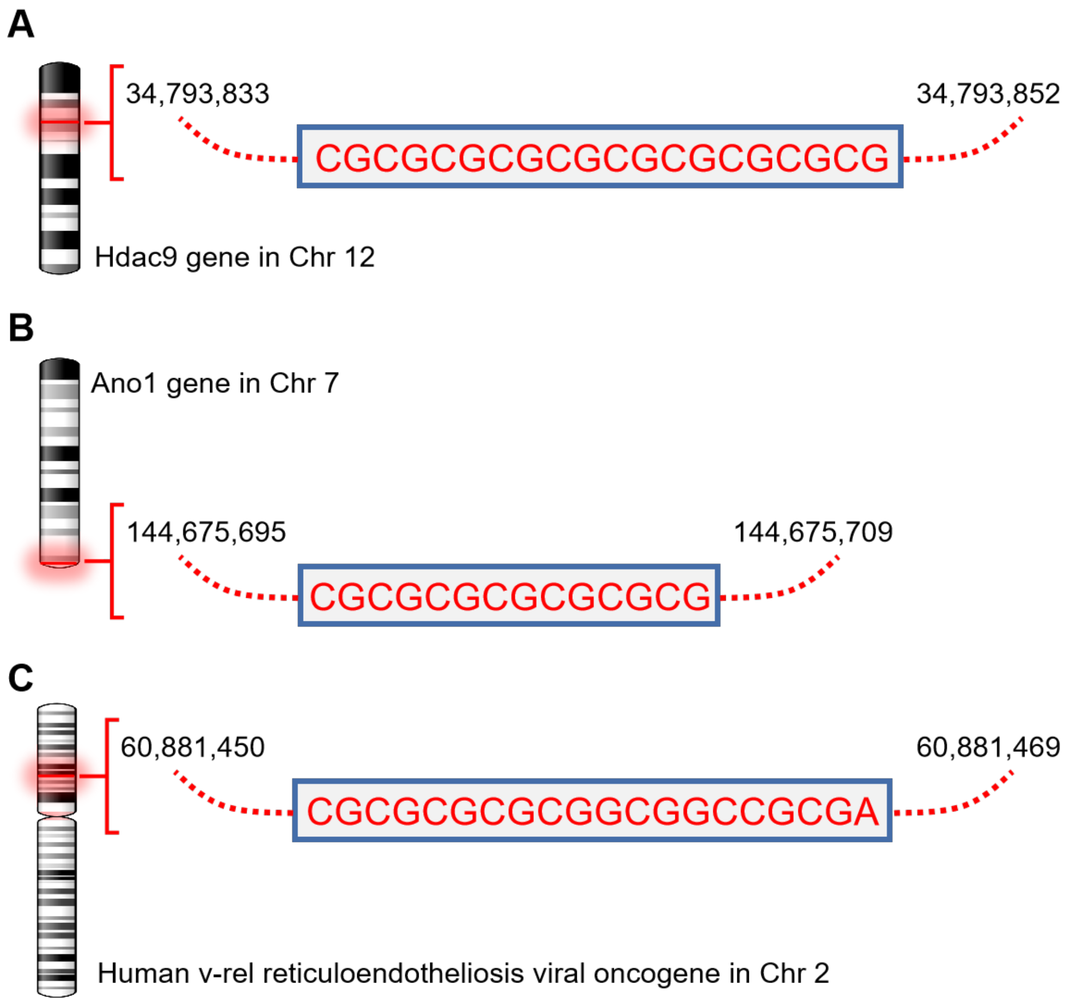

Figure S1. Sequences with enriched CpG repeats in genomic DNA. A series of CpG repeats-rich DNA gene sequences were identified in Hdac9 chromatin 12 (**A**), Ano1 chromatin 7 (**B**) and human v-rel reticuloendotheliosis viral chromatin 2 (REL) (**C**) respectively.

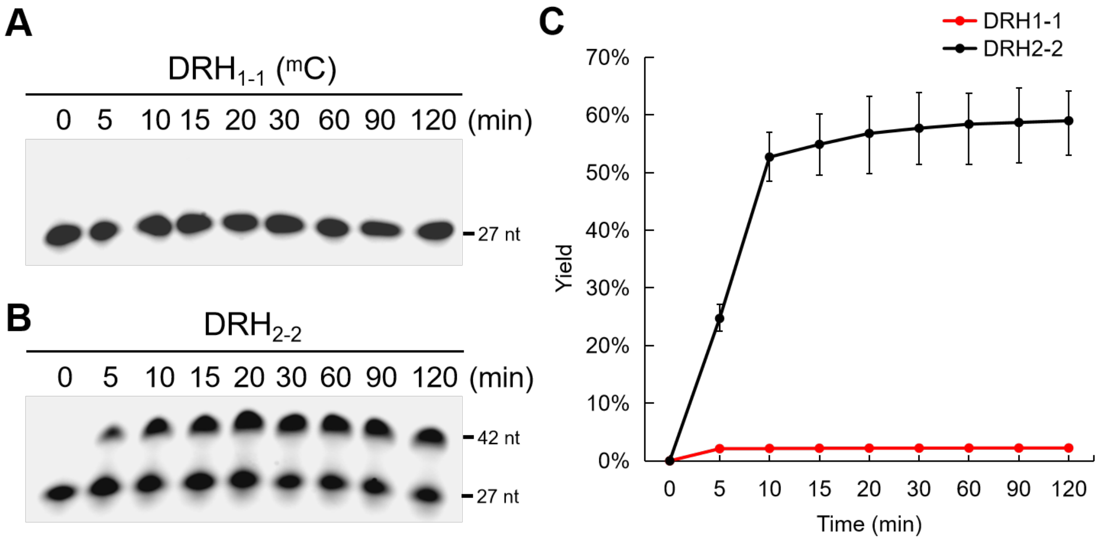

Figure S2. Z-form DNA-RNA hybrid blocks DNA replication *in vitro*. **(A)** Pol δ catalyzed primer extension assay in time dependence using DRH$_{1-1}$. **(B)** Pol δ catalyzed primer extension assay in time dependence using DRH$_{2-2}$. **(C)** The DNA replication yields were plotted overtime derived from **(A)** and **(B)**. Error bars represent mean ± standard deviation. n = 3. ^{m}C represents the hybrid including ^{m}C residue on DNA strand.

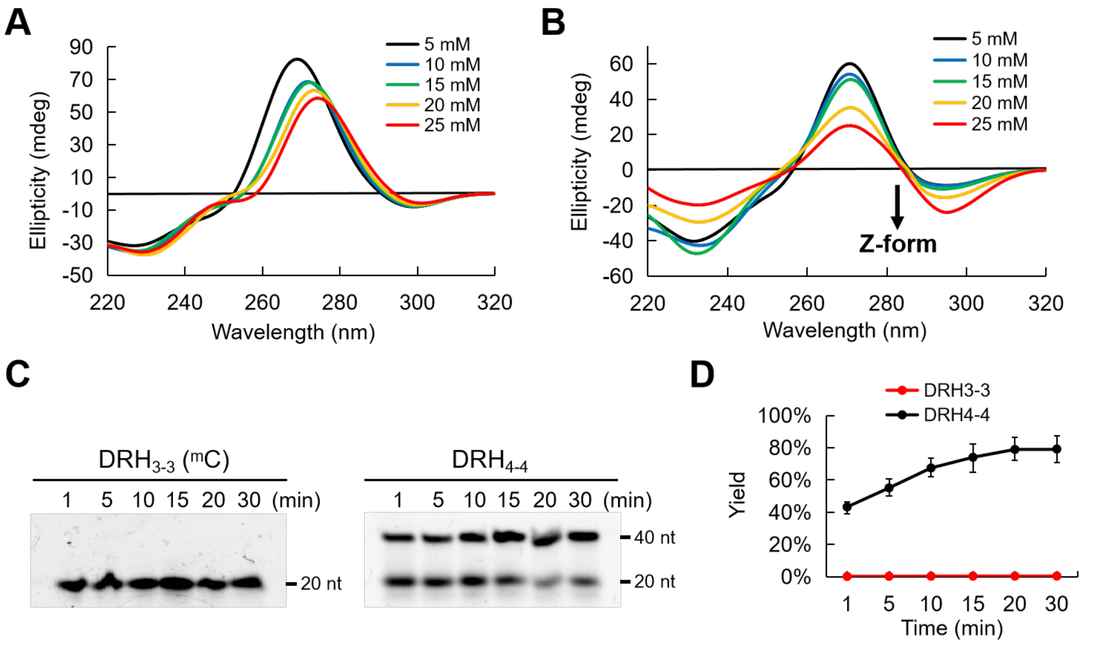

Figure S3. Study of DNA replication using DRH$_{3-3}$ and DRH$_{4-4}$ *in vitro*. **(A)** CD study of DRH$_4$ with different $MgCl_2$ concentrations (5-25 mM). **(B)** CD study of DRH$_3$ with different $MgCl_2$ concentrations (5-25 mM). **(C)** Pol δ catalyzed primer extension assays in denaturing electrophoresis gel using DRH$_{3-3}$ (left) and DRH$_{4-4}$ (right) in the time dependence (1-30 min). ^{m}C represents the hybrid including ^{m}C residue on DNA strand. **(D)** The DNA replication yields were quantitatively analyzed as plotted overtime. Error bars represent mean ± standard

deviation. n = 3.

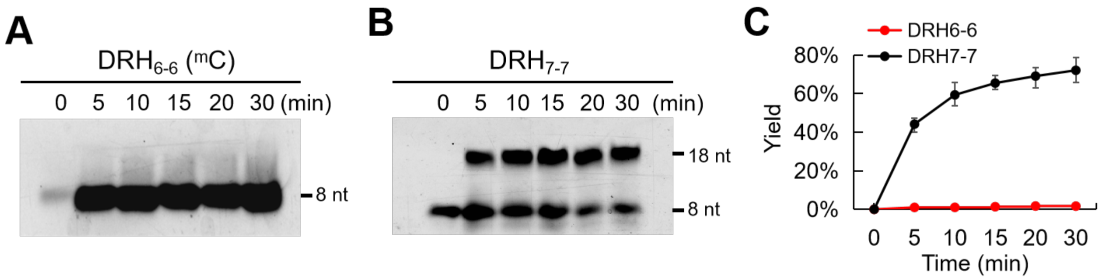

Figure S4. Study of hybrid duplex in Z-form structure blocking DNA replication *in vitro*. Pol I catalyzed primer extension assays in denaturing electrophoresis gel using DRH_{6-6} **(A)** and DRH_{7-7}. ^{m}C represents the hybrid including ^{m}C residue on DNA strand. **(B)** in time-dependence. **(C)** The DNA replication yields were quantitatively analyzed as plotted overtime. Error bars represent mean ± standard deviation. n = 3.

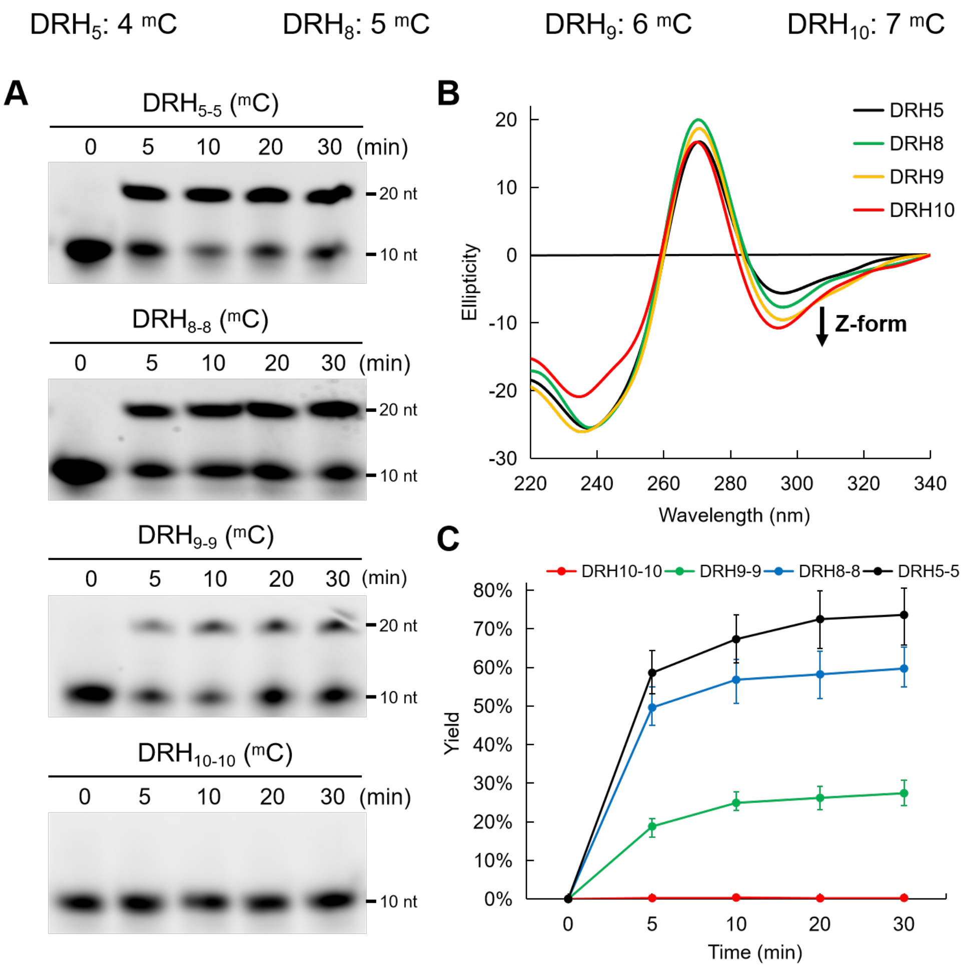

Figure S5. Study the effects of hybrid with different amount of ^{m}C residues upon Z-form structure formation as well as it's capacity towards inhibition of DNA replication. **(A)** Primer extension assays in denaturing electrophoresis gel using DRH_{5-5}, DRH_{8-8}, DRH_{9-9} and DRH_{10-10}. ^{m}C represents the hybrid including ^{m}C residue on DNA strand. **(B)** CD study of DRH_{5-5}, DRH_{8-8}, DRH_{9-9} and DRH_{10-10}. **(C)** The DNA replication yields from **(B)** were quantitatively analyzed

as plotted overtime. Error bars represent mean ± standard deviation. n = 3.

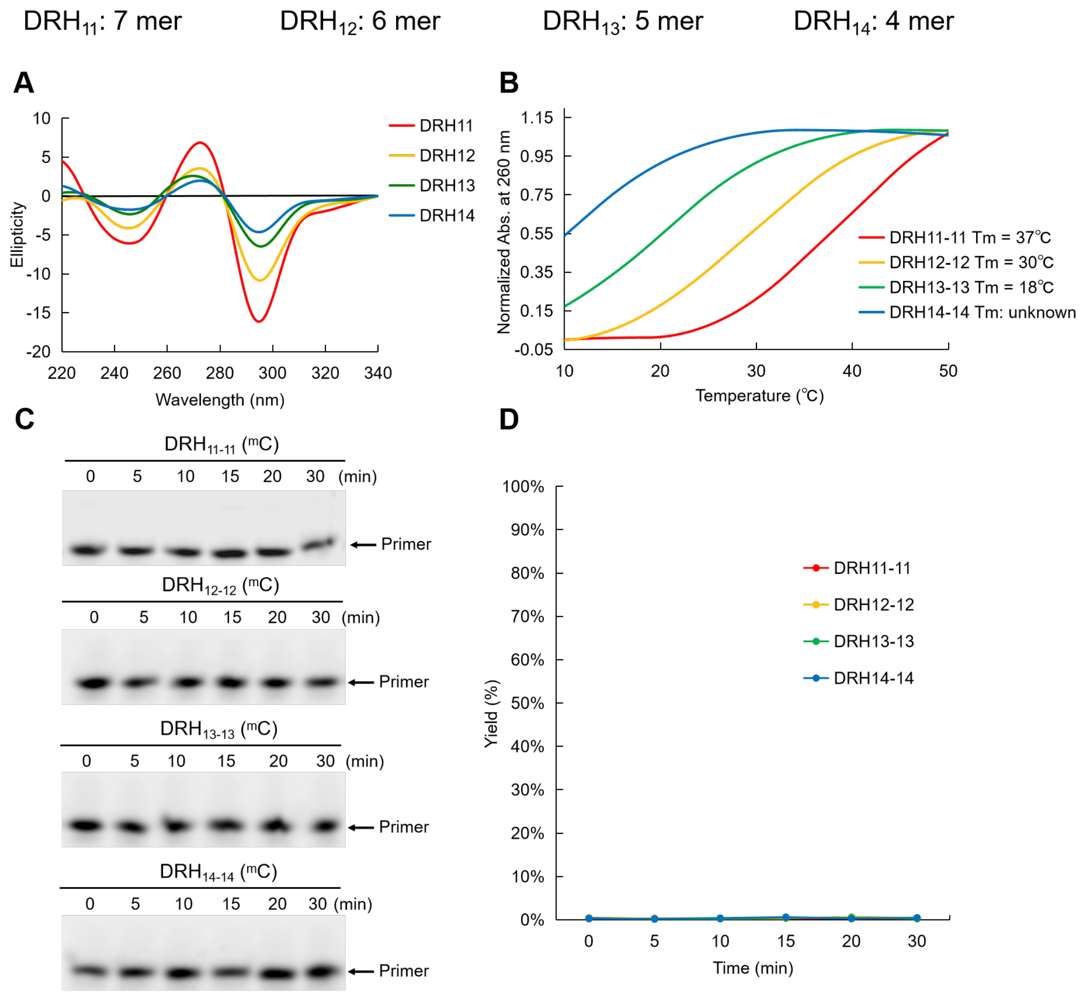

Figure S6. Study the effects of minimal length of Z-form hybrid upon Z-form structure formation as well as it's capacity towards inhibition of DNA replication. **(A)** CD study of DRH_{11}, DRH_{12}, DRH_{13} and DRH_{14} at 37 °C. **(B)** UV-thermal melting curves of DRH_{11}, DRH_{12}, DRH_{13} and DRH_{14}, showing the Tm of $DRH_{11\text{-}11}$ is 37 °C, $DRH_{12\text{-}12}$ is 30 °C, Tm of $DRH_{13\text{-}13}$ is 18 °C and Tm of $DRH_{14\text{-}14}$ is not applicable. **(C)** Primer extension assays in denaturing electrophoresis gel using DRH_{11}, DRH_{12}, DRH_{13} and DRH_{14}. ^{m}C represents the hybrid including ^{m}C residue on DNA strand. **(D)** The DNA replication yields from **(C)** were quantitatively analyzed as plotted overtime. Error bars represent mean ± standard deviation. n = 3.

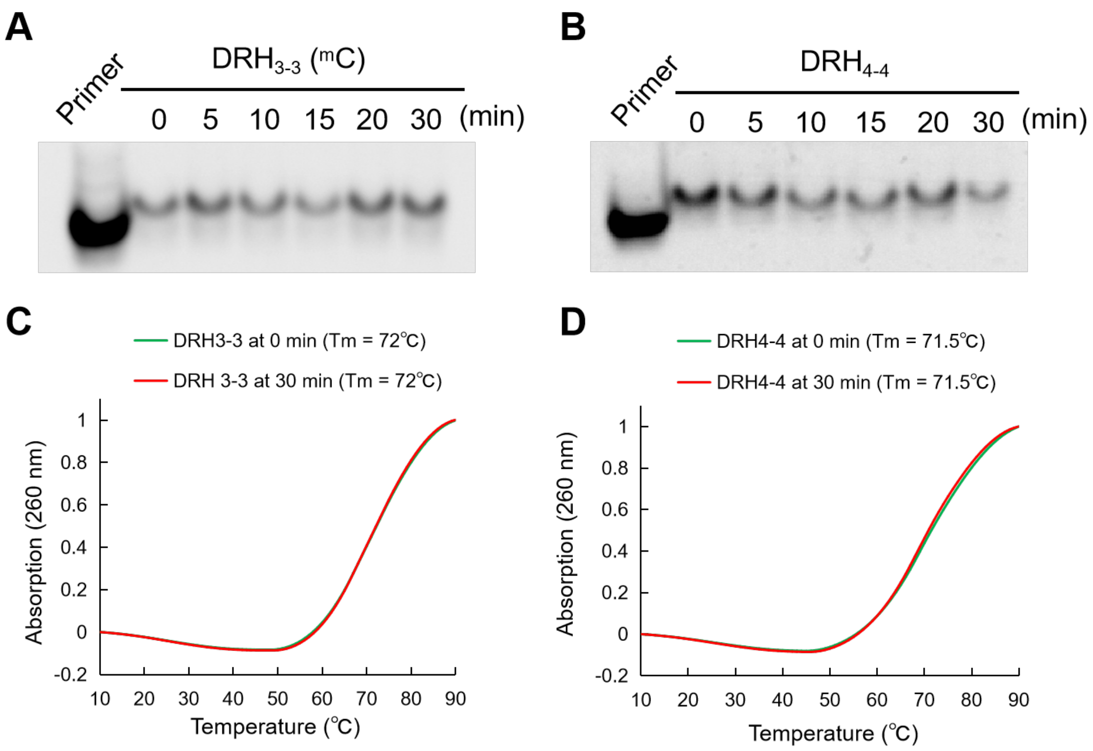

Figure S7. Study of A- and Z-form DNA-RNA hybrid consistent formation in DNA pol δ buffer solution. Non-denaturing electrophoresis gel shows DRH$_{3-3}$ with Z-form **(A)** as well as DRH$_{4-4}$ with A-form **(B)** both consistently remain in hybrid duplex structure in DNA pol buffer solution with time dependence. Primer shows independent Cy3-RNA used in each hybrid. ^{m}C represents the hybrid including ^{m}C residue on DNA strand. UV-thermal melting curves of DRH$_{3-3}$ **(C)** and DRH$_{4-4}$ **(D)** in DNA pol δ buffer solution at 0 and 30 min, showing the consistent Tm of DRH$_{3-3}$ is 72℃ as well as the same Tm of DRH$_{4-4}$ is 71.5 ℃.

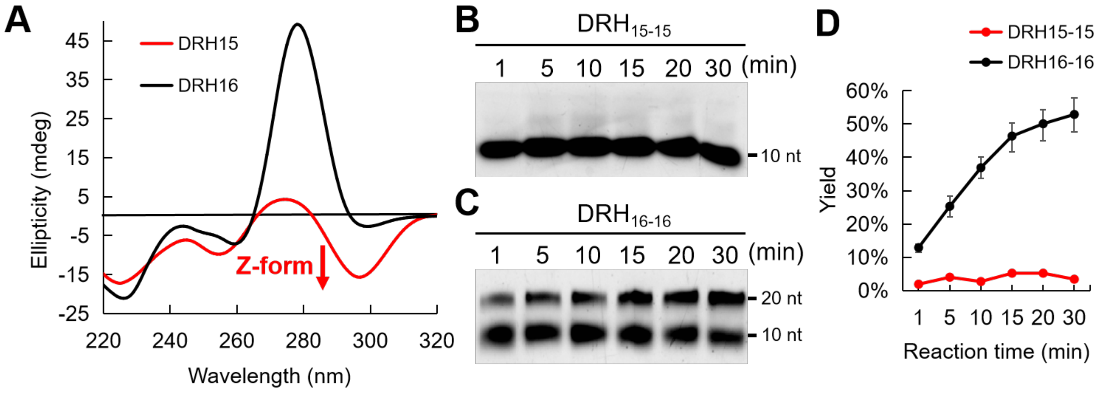

Figure S8. Study of A- and Z-form DNA-RNA hybrid with consistent amounts of ^{F}G residues with DNA replication *in vitro*. **(A)** CD study of DRH$_{15}$ and DRH$_{16}$. **(B)** Pol δ catalyzed primer extension assays in denaturing electrophoresis gel using DRH$_{15-15}$. **(C)** Pol δ catalyzed primer extension assays in denaturing electrophoresis gel using DRH$_{16-16}$. **(D)** The DNA replication yields were quantitatively analyzed as plotted overtime. Error bars represent mean ± standard deviation. n = 3.

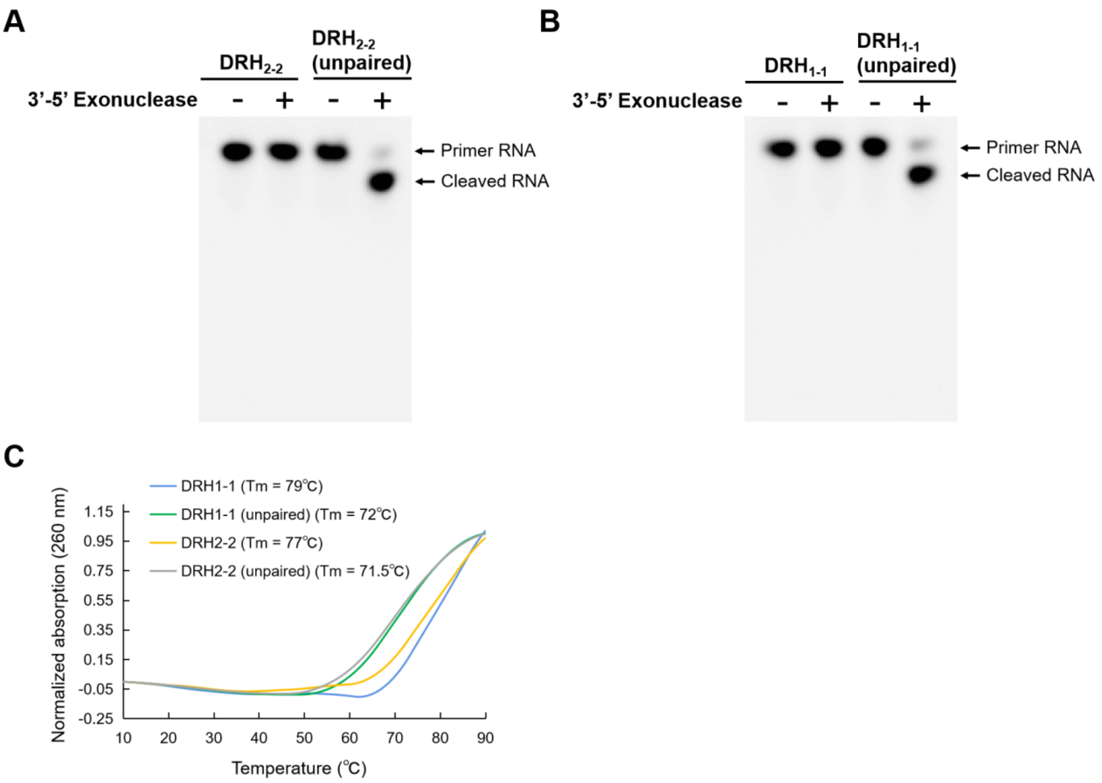

Figure S9. Study primer RNA stability of Z-form hybrid against 3'-5' exonuclease. **(A)** DRH$_{2-2}$ and DRH$_{2-2}$ (unpaired) in absence or presence of 3'-5' exonuclease. **(B)** DRH$_{1-1}$ and DRH$_{1-1}$ (unpaired) in absence or presence of 3'-5' exonuclease. **(C)** UV-thermal melting curves of DRH$_{1-1}$, DRH$_{1-1}$ (unpaired), DRH$_{2-2}$ and DRH$_{2-2}$ (unpaired), showing the Tm of DRH$_{1-1}$ (79°C), DRH$_{1-1}$ (unpaired) (72°C), DRH$_{2-2}$ (77°C) and DRH$_{2-2}$ (unpaired) (71.5°C) respectively. ^{m}C represents the hybrid including ^{m}C residue on DNA strand.

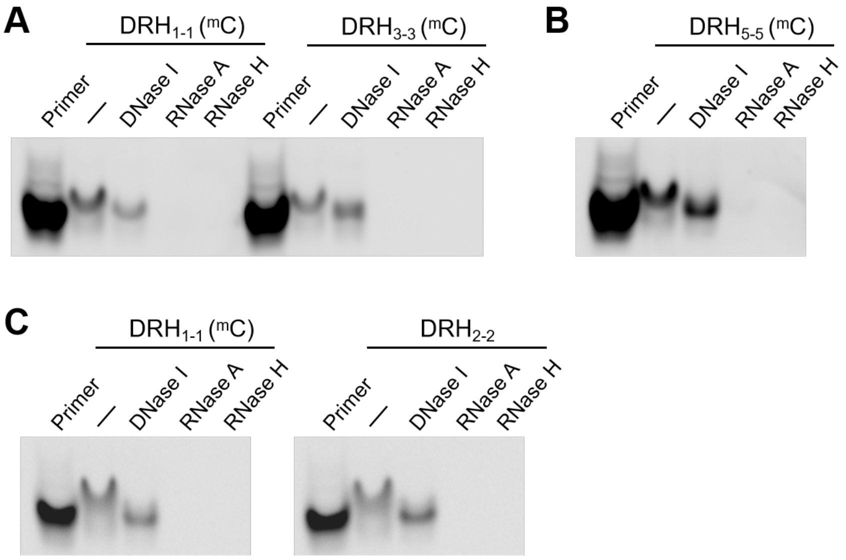

Figure S10. Study stability of A- and Z-form hybrids in DNase I, RNase A and RNase H respectively using denaturing electrophoresis gel. (**A**) Z-form DRH$_{1-1}$ and DRH$_{3-3}$ were

subjected to nuclease. (**B**) Z-form $DRH_{5\text{-}5}$ was subjected to nuclease. (**C**) Comparison of stability of Z-form $DRH_{1\text{-}1}$ and A-form $DRH_{2\text{-}2}$ with nuclease. Primer show independent Cy3-RNA used in each hybrid. ^{m}C represents the hybrid including ^{m}C residue on DNA strand.

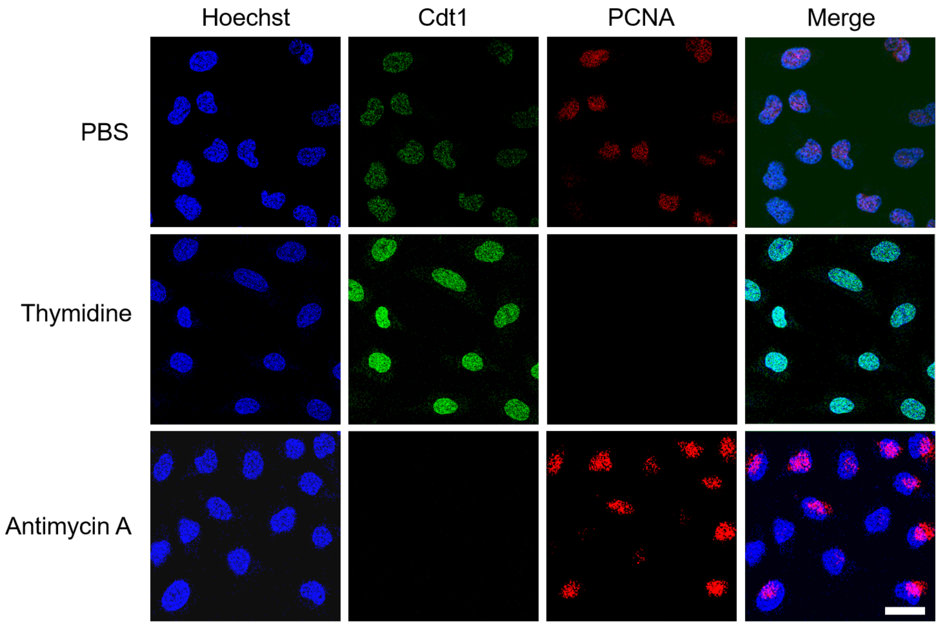

Figure S11. Imunnofluorescence assay of HeLa cells arrested at G1 and S phases using cdt1 and PCNA antibody, respectively. Cdt1 and PCNA were immunostained using anti Cdt1 antibody (AF488) and Cdt1 antibody (AF647), respectively. Blue fluorescence indicated nuclei with Hoechst. Green color is from cdt1 as well as red color is from PCNA. The merge panel shows a colocalization event. Scale bars, 15 µm.

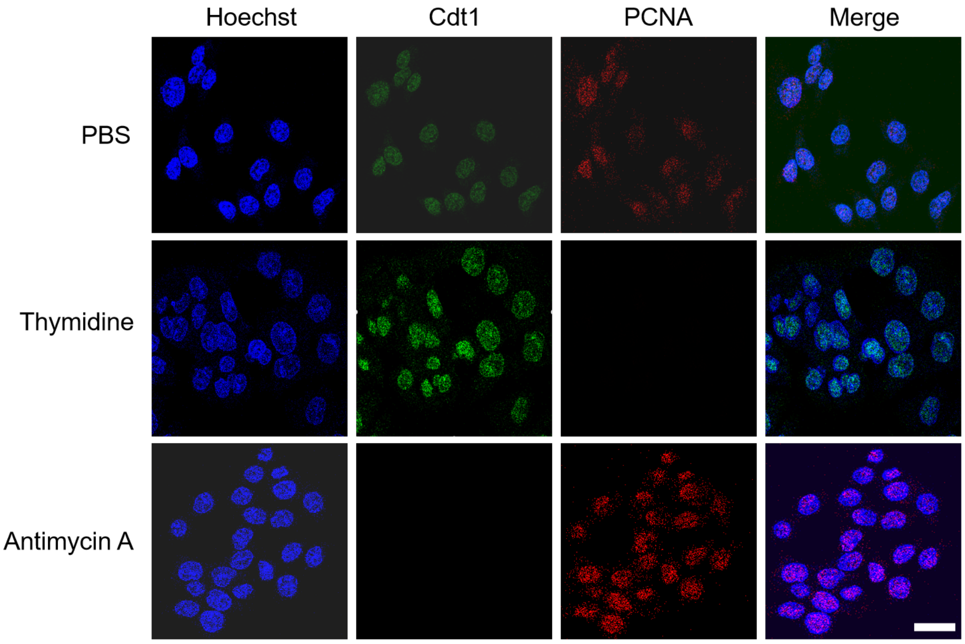

Figure S12. Imunnofluorescence assay of HT29 cells arrested at G1 and S phases using cdt1 and PCNA antibody, respectively. Cdt1 and PCNA were immunostained using anti Cdt1

antibody (AF488) and Cdt1 antibody (AF647), respectively. Blue fluorescence indicated nuclei with Hoechst. Green color is from cdt1 as well as red color is from PCNA. The merge panel shows a colocalization event. Scale bars, 20 μm.

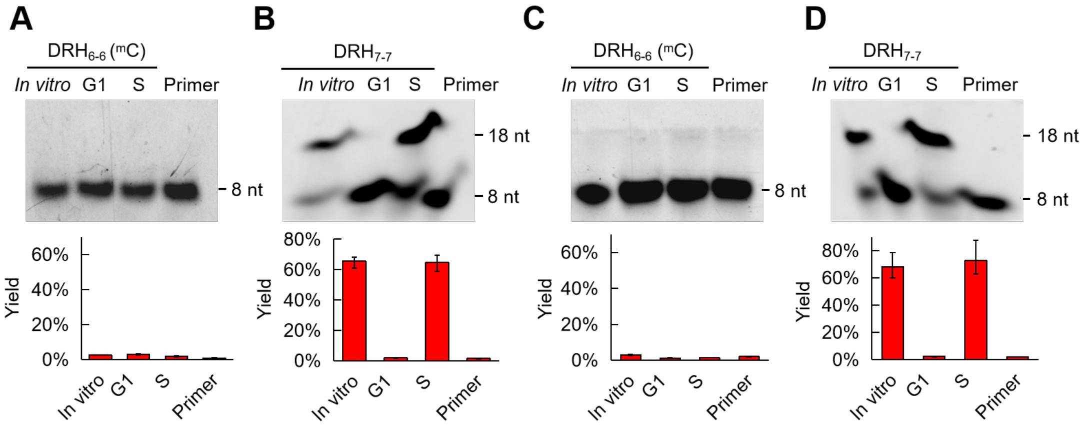

Figure S13. Study of hybrid duplex in Z-form structure blocking DNA replication in cells. **(A)** Study DNA replication using $DRH_{6\text{-}6}$ in HeLa cells, the yields in plotted histogram. **(B)** Study DNA replication using $DRH_{7\text{-}7}$ in HeLa cells, the yields in plotted histogram. **(C)** Study DNA replication using $DRH_{6\text{-}6}$ in HT29 cells, the yields in plotted histogram. **(D)** Study DNA replication using $DRH_{7\text{-}7}$ in HT29 cells, the yields in plotted histogram. Error bars represent mean ± standard deviation. n = 3. Primer show independent Cy3-RNA used in each hybrid. ^{m}C represent the hybrid including ^{m}C residue on DNA strand.

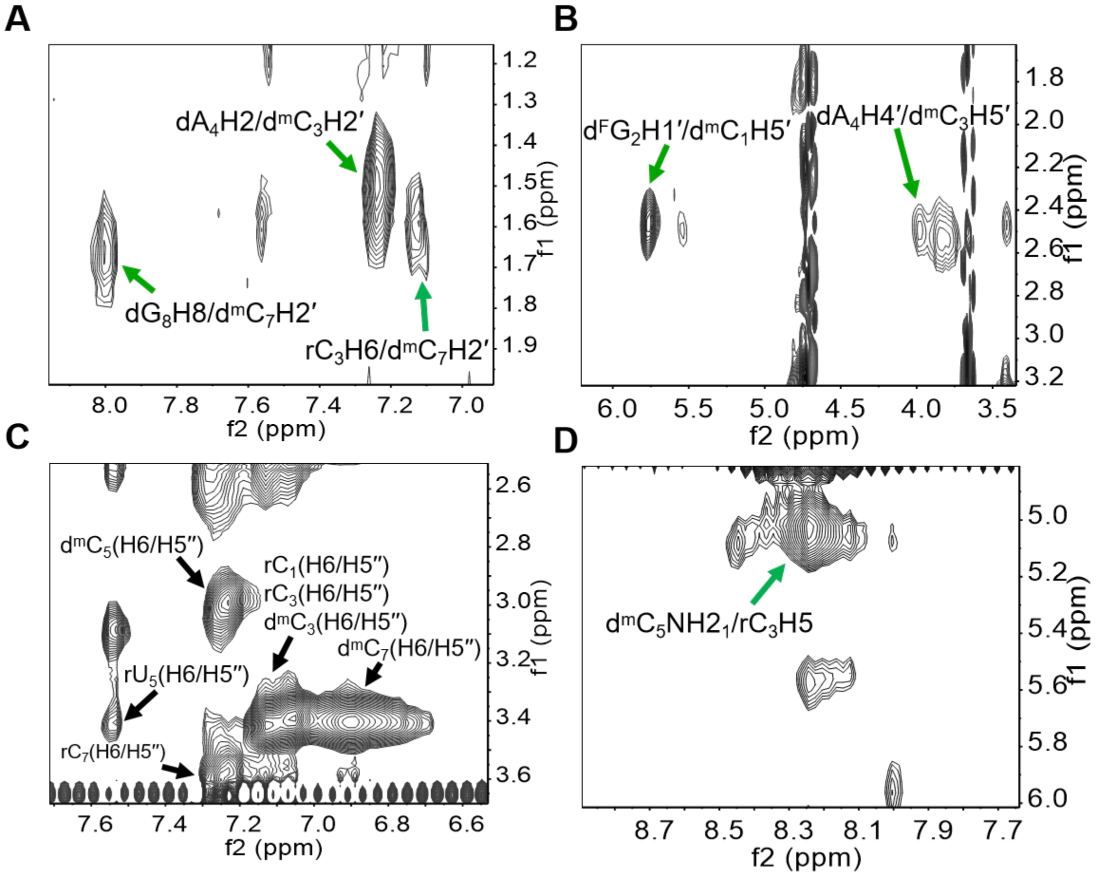

Figure S14. The unique Z-form structure of DRH$_6$ studied by 2D NOESY spectrum. **(A)** These cross peaks as dG$_8$H8/d^{m}C$_7$H2′, dA$_4$H2/d^{m}C$_3$H2′ and rC$_3$H6/d^{m}C$_7$H2′ showed that H2′ of d^{m}C$_3$ and d^{m}C$_7$ in upshift field (green arrows). **(B)** The cross peaks as d^{F}G$_2$H1′/d^{m}C$_1$H5′ and dA$_4$H4′/d^{m}C$_3$H5′ showed that H5′ of d^{m}C$_1$ and d^{m}C$_3$ in upshift field (green arrows). **(C)** Intra-nucleotide cross peaks as all H6/H5″ of cytosine residues in RNA strand associated with signals as d^{m}C$_3$(H6/H5″), d^{m}C$_5$(H6/H5″) and d^{m}C$_7$(H6/H5″) collectively showed these nucleotide in *anti* conformation (black arrows). **(D)** The cross peaks of amino proton of d^{m}C$_5$ with H5 of rC$_3$ was observed (green arrows).

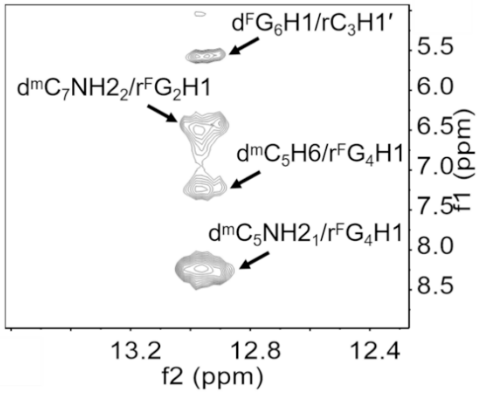

Figure S15. Watson-Crick base pairs formation in DRH$_6$ studied by 2D NOESY spectrum. The cross peaks of imino proton of r^{F}G$_4$ with amino proton and H6 of d^{m}C$_5$ associated with d^{m}C$_7$NH2$_2$/r^{F}G$_2$H1 and d^{F}G$_6$H1/rC$_3$H1′ were observed (black arrows).

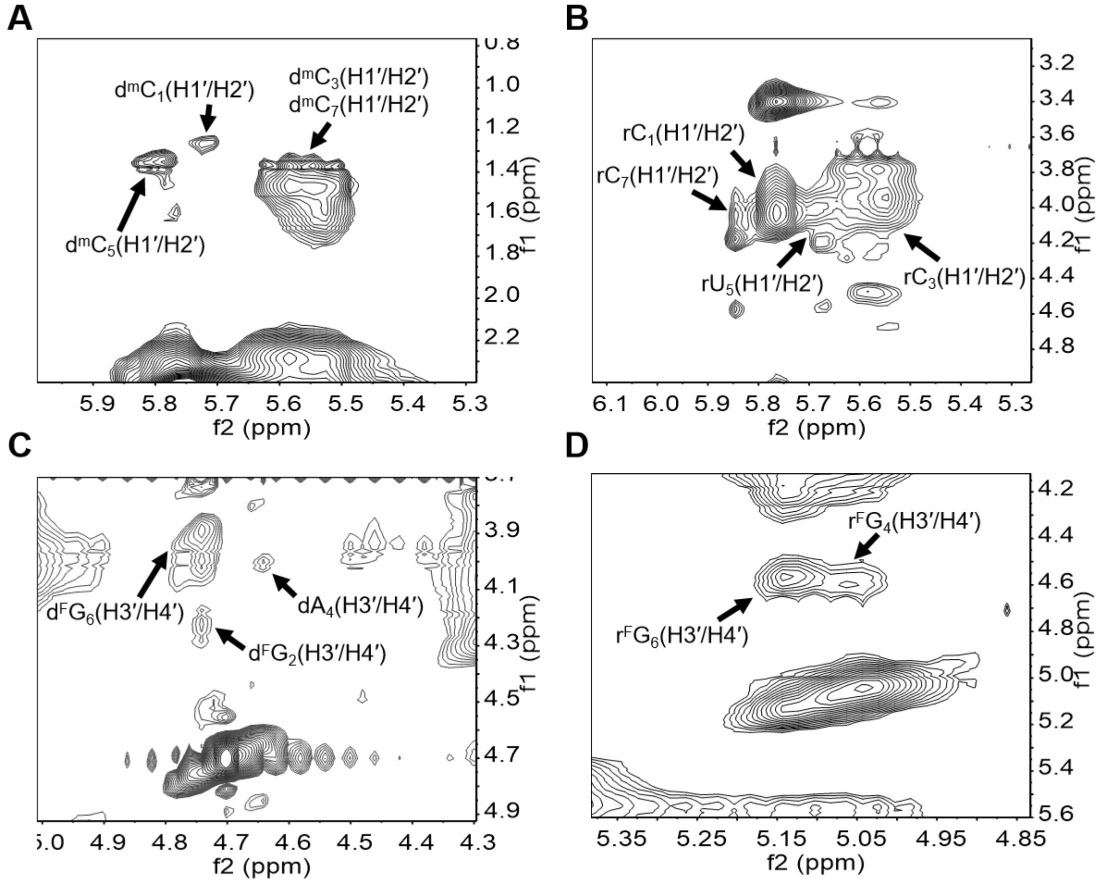

Figure S16. Anomeric regions of Z-form DRH_6 in 2D NOESY. **(A)** All cross peaks, H1′/H2′ of $d^{m}C_1$, $d^{m}C_3$, $d^{m}C_5$ and $d^{m}C_7$ indicated that all cytosine residues in DNA strand with C2′-*endo* sugar pucker, marking by black arrows. **(B)** The cross peaks, H1′/H2′ of rC_1, rC_3, rU_5 and rC_7 indicated that all cytosine residues in RNA strand with C2′-*endo* sugar pucker, marking by black arrow. **(C)** All cross peaks, H3′/H4′ from $d^{F}G_2$, dA_4 and $d^{F}G_6$ indicated that these guanine residues in DNA strand with C3′-*endo* sugar pucker, marking by black arrows. **(D)** The cross peaks, $r^{F}G_4$(H3′/H4′) and $r^{F}G_6$(H3′/H4′) indicated that these guanine residues with C3′-*endo* sugar pucker, marking by black arrows.

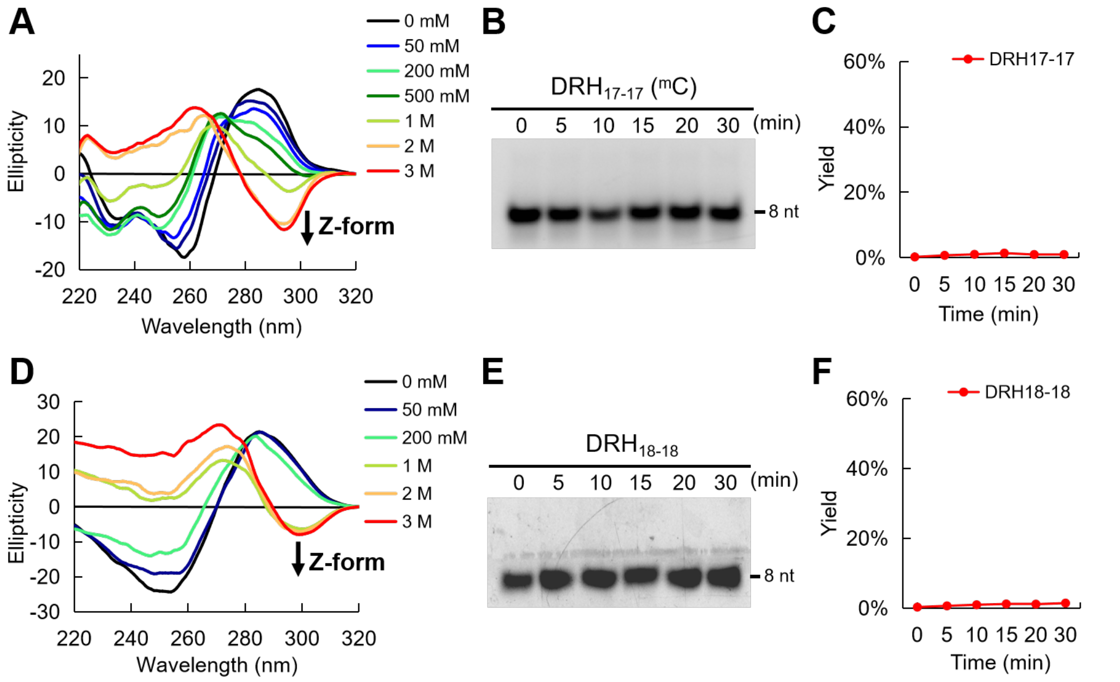

Figure S17. CD spectra of DNA-RNA hybrids in A–Z transition at various NaCl concentrations and blocking DNA replication by denaturing gel electrophoresis. **(A)** CD spectra of DRH_{17} with increasing NaCl concentrations. **(B)** Pol δ catalyzed primer extension assay in time dependence using $DRH_{17\text{-}17}$ in 2 M NaCl. ^{m}C represent the hybrid including ^{m}C residue on DNA strand. **(C)** The DNA replication yields were plotted overtime derived from **(B)**. **(D)** CD study of DRH_{18} with increasing NaCl concentrations. **(E)** Pol δ catalyzed primer extension assay in time dependence using $DRH_{18\text{-}18}$ in 2 M NaCl. **(F)** The DNA replication yields were plotted overtime derived from **(E)**. Error bars represent mean ± standard deviation. n = 3.

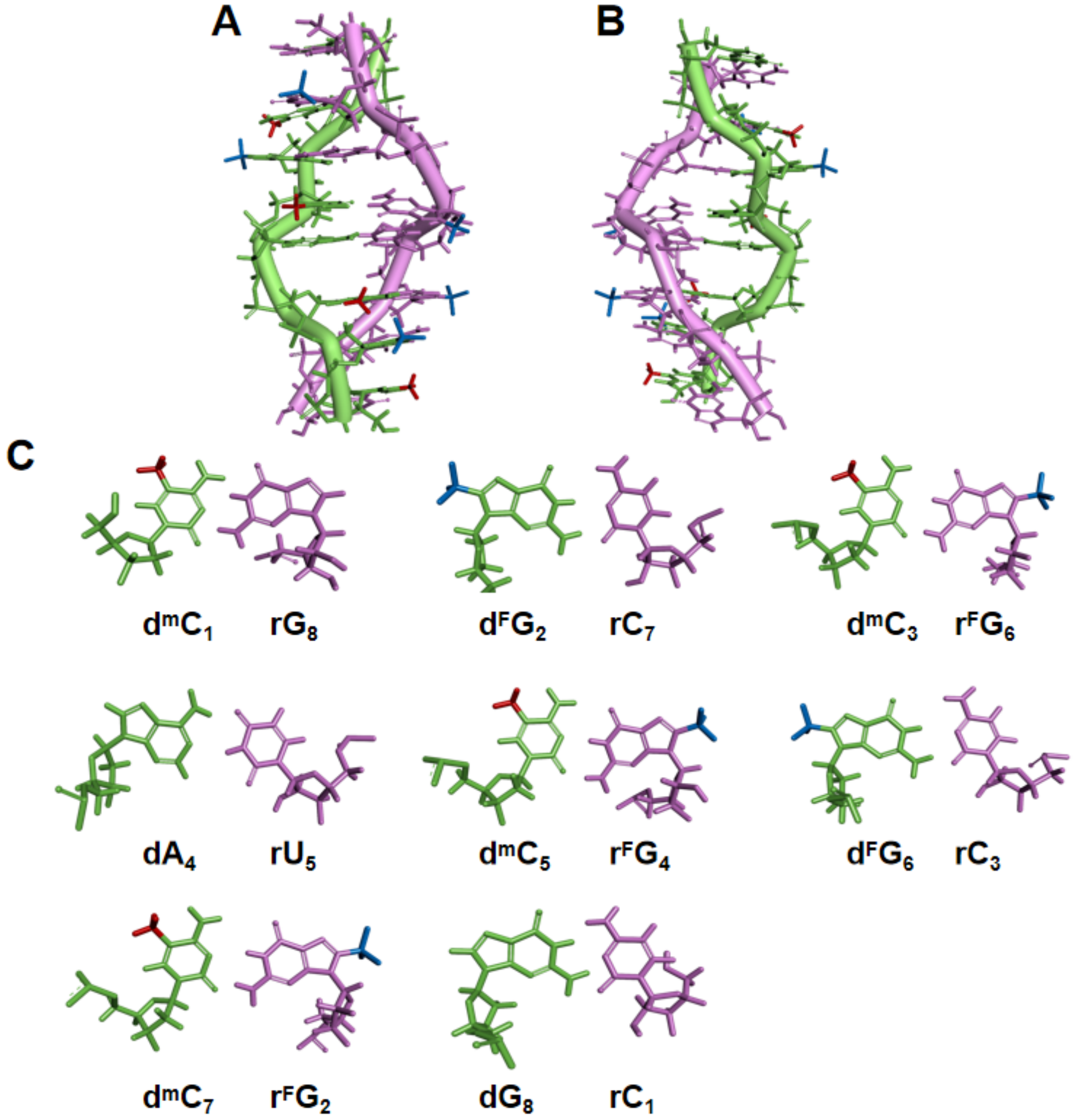

Figure S18. The dynamic simulation model for Z-form DRH_6 using BIOVIA Discovery Studio 4.5. **(A)** Ribbon view of Z-form DRH_6 from major groove. **(B)** Ribbon view of Z-form DRH_6 from minor groove. All above, DNA strand at green color and RNA strand at pink color. Red color labeled 5-CH_3 groups and blue color stained 8-CF_3 group. **(C)** All inter-strand watson-crick base pairs involved in the Z-form DRH_6 generated by molecular simulation, where all nucleosides with numbers were indicated.

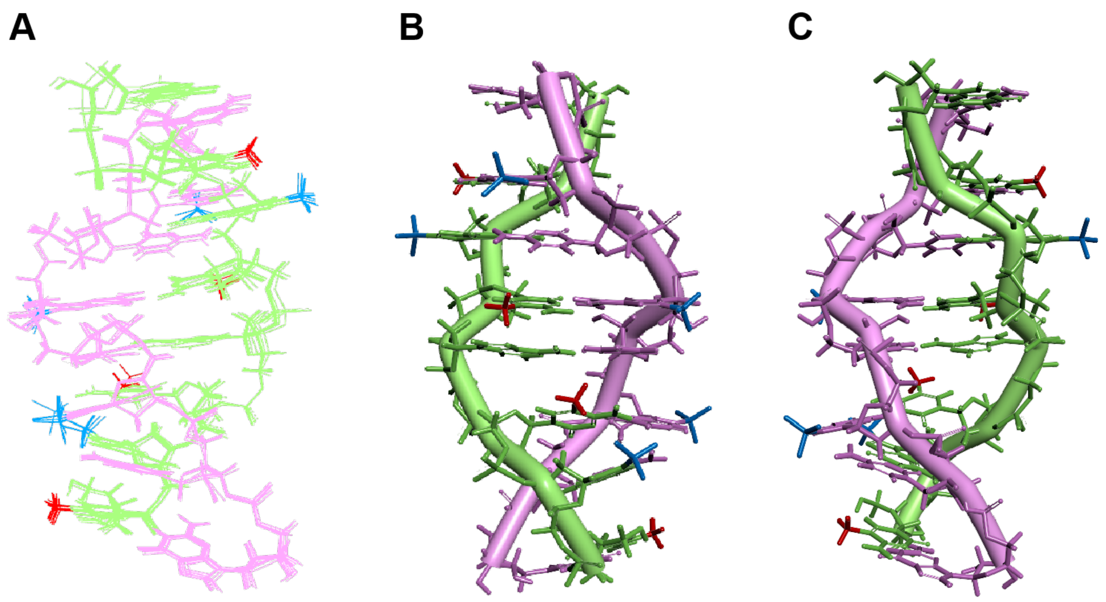

Figure S19. The molecular model of Z-form DRH_6 by using dynamic simulation Amber 18 package. **(A)** Superposition of 10 conformers representing the refined structure of DRH_6 in the Z-form with lowest total energy as viewed from the minor groove. **(B)** Ribbon view of Z-form DRH_6 from major groove. **(C)** Ribbon view of Z-form DRH_6 from minor groove. All above, DNA strand at green color and RNA strand at pink color. Red color labeled 5-CH_3 groups and blue color stained 8-CF_3 group.

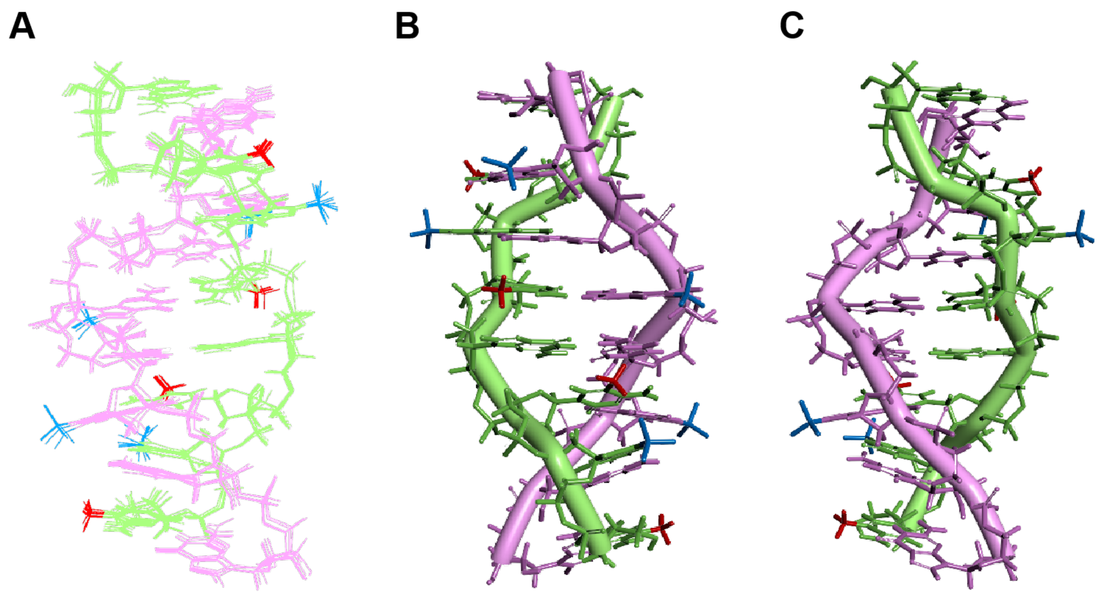

Figure S20. The molecular model of Z-form DRH_{19} by using BIOVIA Discovery Studio 4.5 with dynamic simulation. **(A)** Superposition of 10 conformers representing the refined structure of DRH_{19} in the Z-form with lowest total energy as viewed from the minor groove. **(B)** Ribbon view of Z-form DRH_{19} from major groove. **(C)** Ribbon view of Z-form DRH_{19} from minor groove. All above, DNA strand at green color and RNA strand at pink color. Red color labeled 5-CH_3 groups and blue color stained 8-CF_3 group.

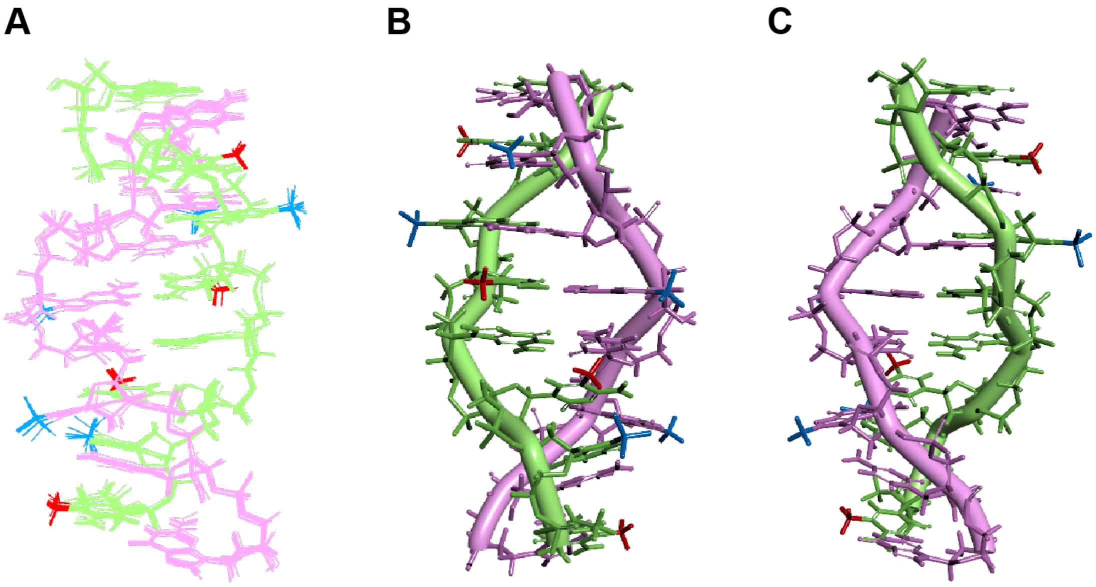

Figure S21. The molecular model of Z-form DRH_{19} by using dynamic simulation Amber 18 package. **(A)** Superposition of 10 conformers representing the refined structure of DRH_{19} in the Z-form with lowest total energy as viewed from the minor groove. **(B)** Ribbon view of Z-form DRH_{19} from major groove. **(C)** Ribbon view of Z-form DRH_{19} from minor groove. All above, DNA strand at green color and RNA strand at pink color. Red color labeled 5-CH_3 groups and blue color stained 8-CF_3 group.

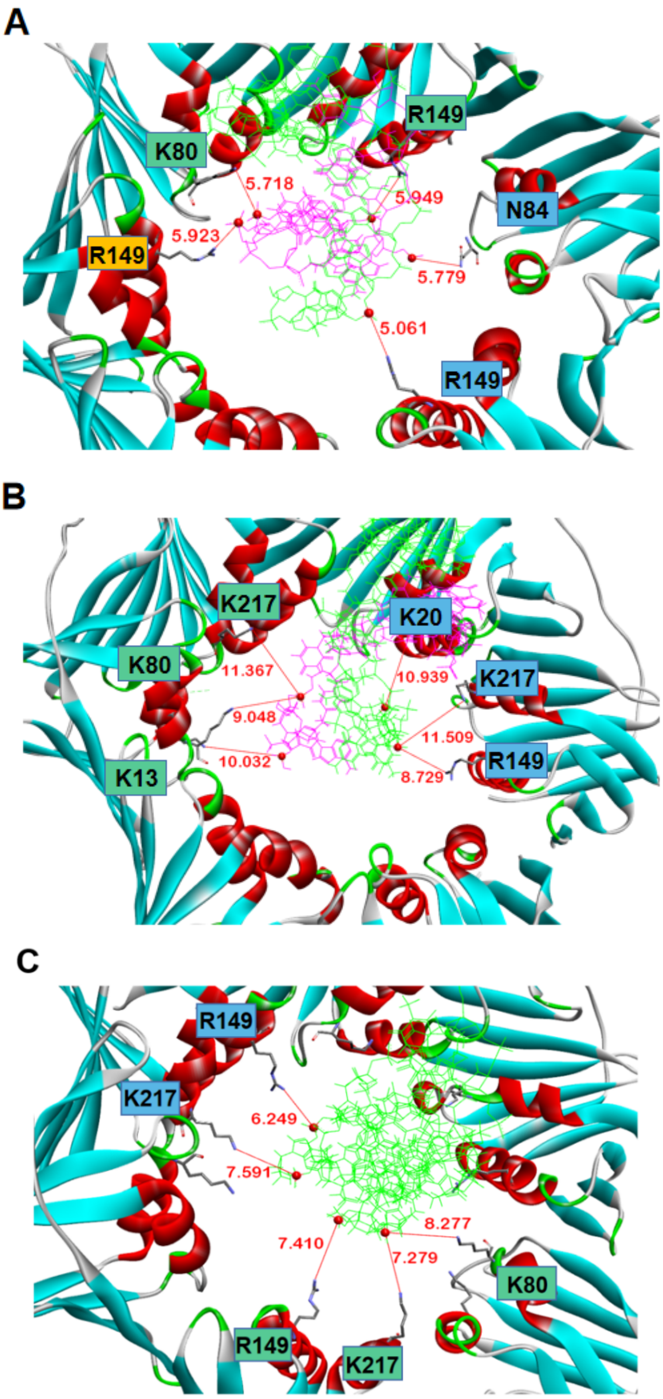

Figure S22. Study interaction of PCNA with DNA-RNA hybrid or Z-DNA. PCNA subunits are shown as cartoons and colored by secondary type. Nucleic acid duplex are shown as line type, in which green represents DNA strand and pink represents RNA strand. **(A)** PCNA interacts and stabilizes A-form DNA-RNA hybrid. Phosphates show a coulombic interaction distance (<6 Å) to PCNA residues. **(B)** PCNA cannot interact with Z-form DNA-RNA hybrid. Phosphates show distance (>8 Å) to PCNA residues. **(C)** PCNA cannot interact with Z-form DNA. Phosphates show distance (>7 Å in average) to PCNA residues. Inter-atomic distance are shown by red solid line. Indicated phosphates were labeled by red ball and PCNA interacting residues are shown as sticks and labeled.

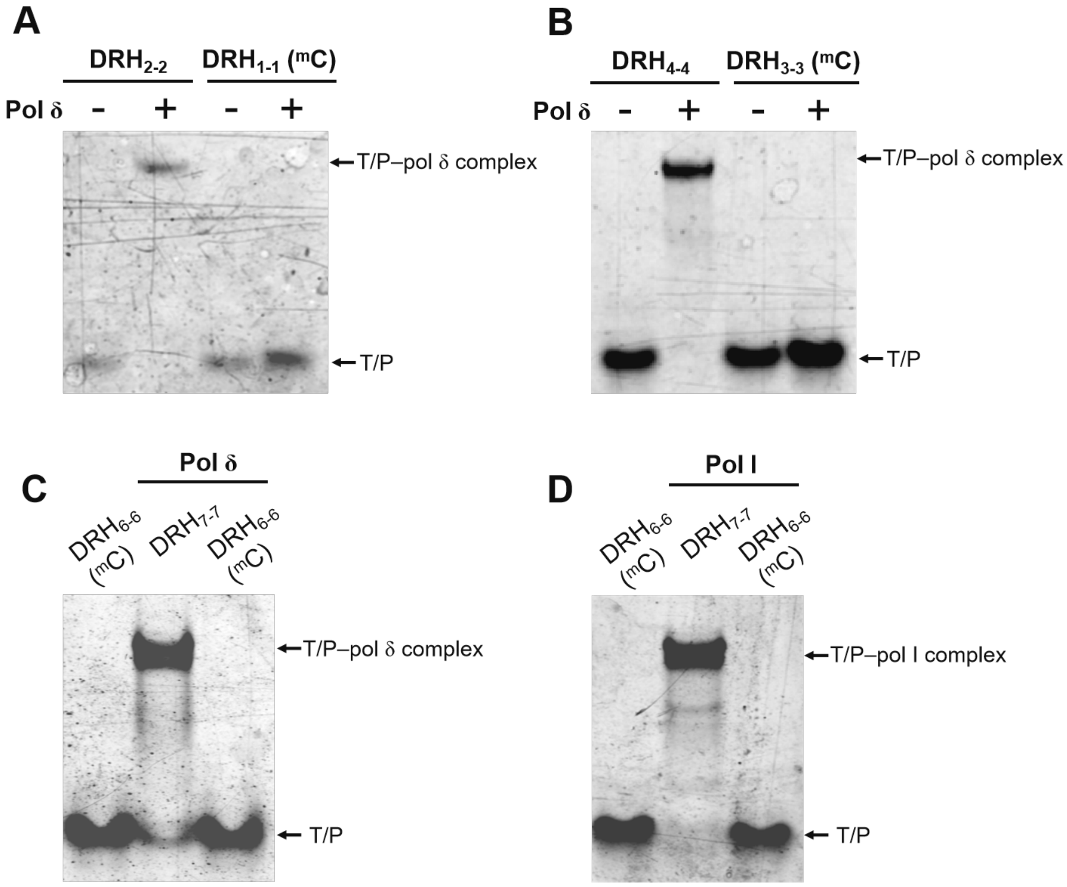

Figure S23. Pol binding assay with DNA-RNA hybrids in non-denaturing polyacrylamide gel electrophoresis. **(A)** DRH$_{1-1}$ and DRH$_{2-2}$ in absence or presence of pol δ. **(B)** DRH$_{3-3}$ and DRH$_{4-4}$ in absence or presence of Pol δ. **(C)** DRH$_{6-6}$ and DRH$_{7-7}$ were subjected to pol δ. **(D)** DRH$_{6-6}$ and DRH$_{7-7}$ were subjected to pol I. T/P represents the DNA-template/RNA-primer used for DNA replication study. ^{m}C represent the hybrid including ^{m}C residue on DNA strand. All hybrids concentration is 1.0 nM, pol δ concentrations are 0, or 20 nM respectively.

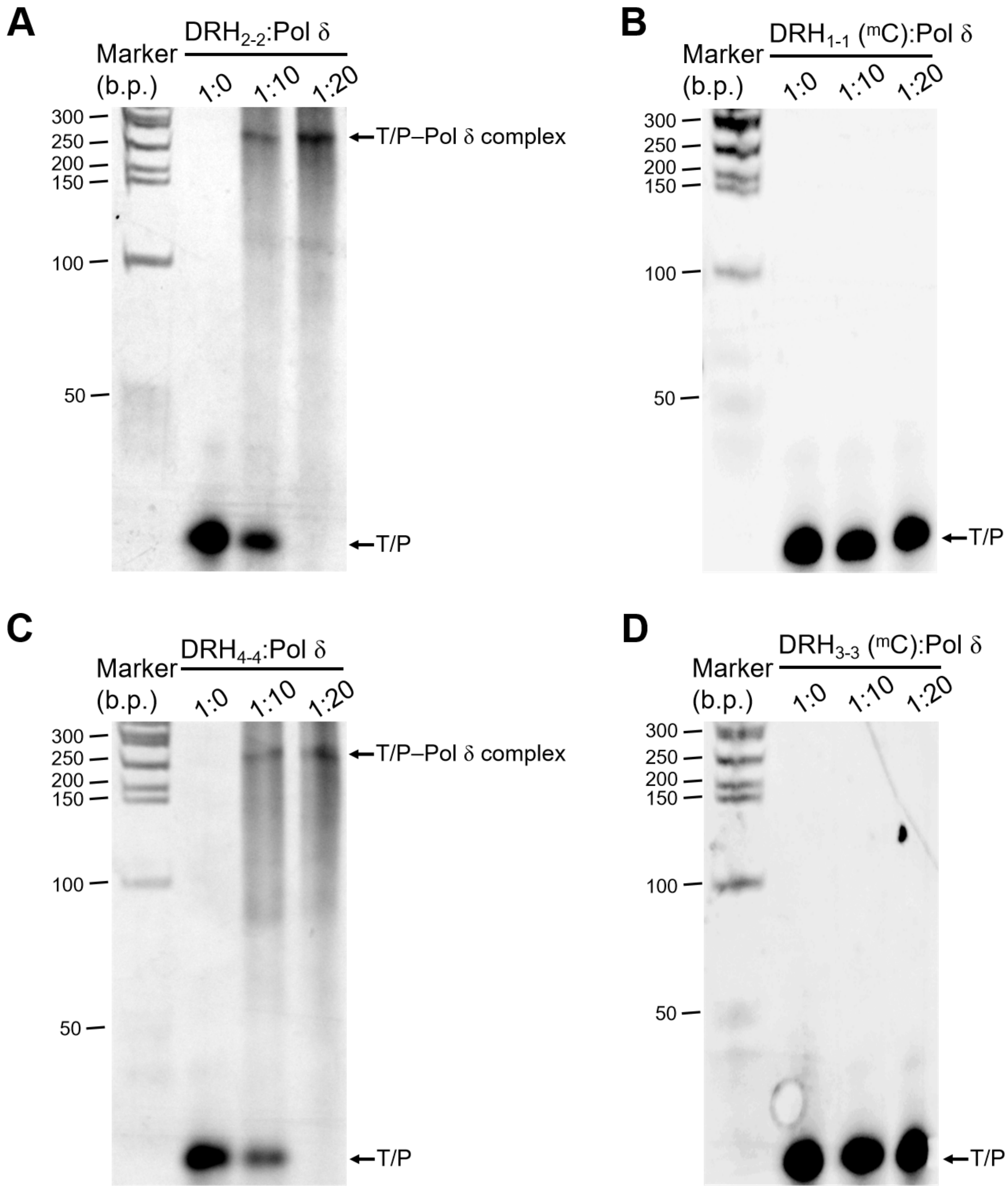

Figure S24. Titration assay of DNA-RNA hybrids with Pol δ at different equivalences (1:0, 1:10 and 1:20) in non-denaturing polyacrylamide gel electrophoresis. **(A)** DRH_{2-2} in different equivalences of pol δ. **(B)** DRH_{1-1} in different equivalences of pol δ. **(C)** DRH_{4-4} in different equivalences of pol δ. **(D)** DRH_{3-3} in different equivalences of pol δ. T/P represents the DNA-template/RNA-primer used for DNA replication study. ^{m}C represent the hybrid including ^{m}C residue on DNA strand. Markers showing base pairs (b.p.) were used on the left side of gel. All hybrids concentration is 1.0 nM, pol δ concentrations are 0, 10 and 20 nM respectively.

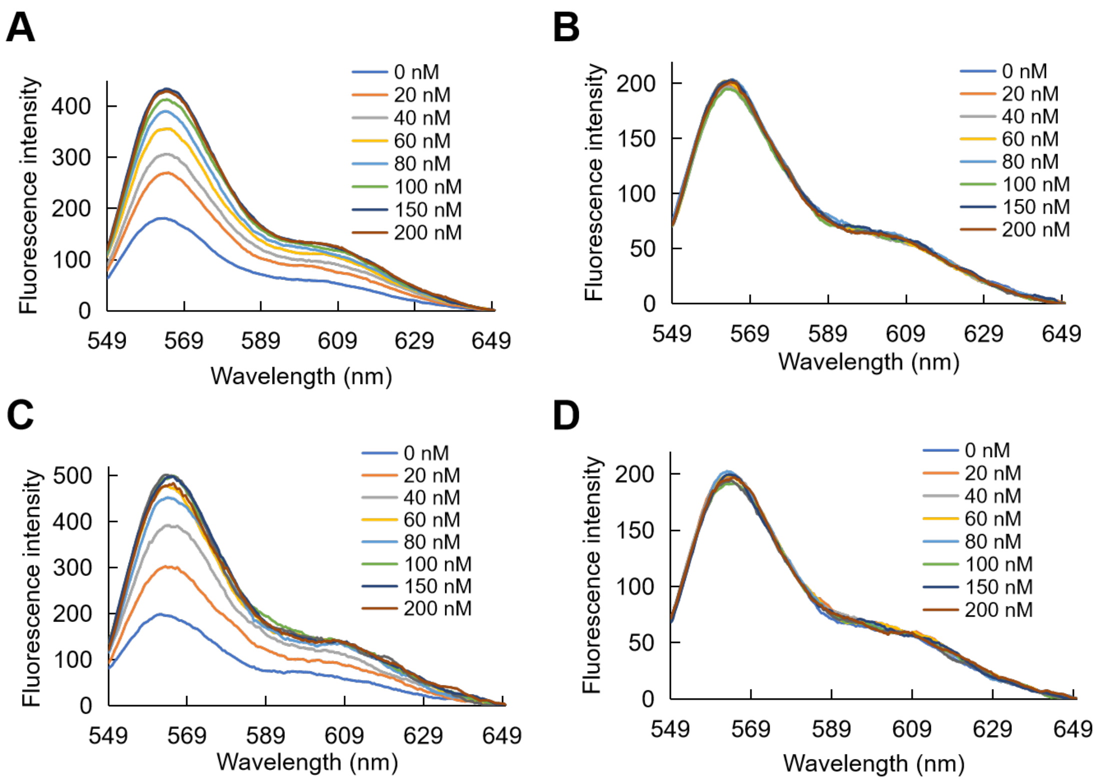

Figure S25. Direct comparison of the steady-state fluorescence properties of pol δ bound to various template-primer with Cy3 at the 5'-terminus of primer. (**A**) 100 nM DRH$_{2\text{-}2}$ was subjected by 0-200 nM pol δ. (**B**) 100 nM DRH$_{1\text{-}1}$ was subjected by 0-200 nM pol δ. (**C**) 100 nM DRH$_{4\text{-}4}$ was subjected by 0-200 nM pol δ. (**D**) 100 nM DRH$_{3\text{-}3}$ was subjected by 0-200 nM pol δ. The excitation wavelength was 540 nm and emitted at 548-648 nm in fluorescence spectroscopy.

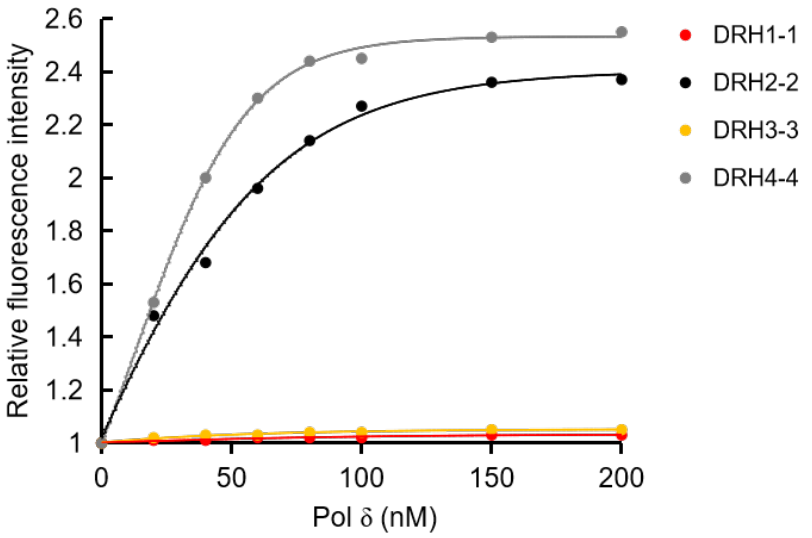

Figure S26. Equilibrium titrations of DRH$_{1\text{-}1}$, DRH$_{2\text{-}2}$, DRH$_{3\text{-}3}$ and DRH$_{4\text{-}4}$ with pol δ. A constant amount of template-primer (100 nM) with Cy3 at 5'-terminus of primer was titrated with increasing concentration of pol δ. The fluorescence was excited at 540 nm and observed at 565 nm. Each measurement was repeated three times, and the average value of the fluorescence intensity was recorded. The analysis of the data yielded the dissociation constant K_d.

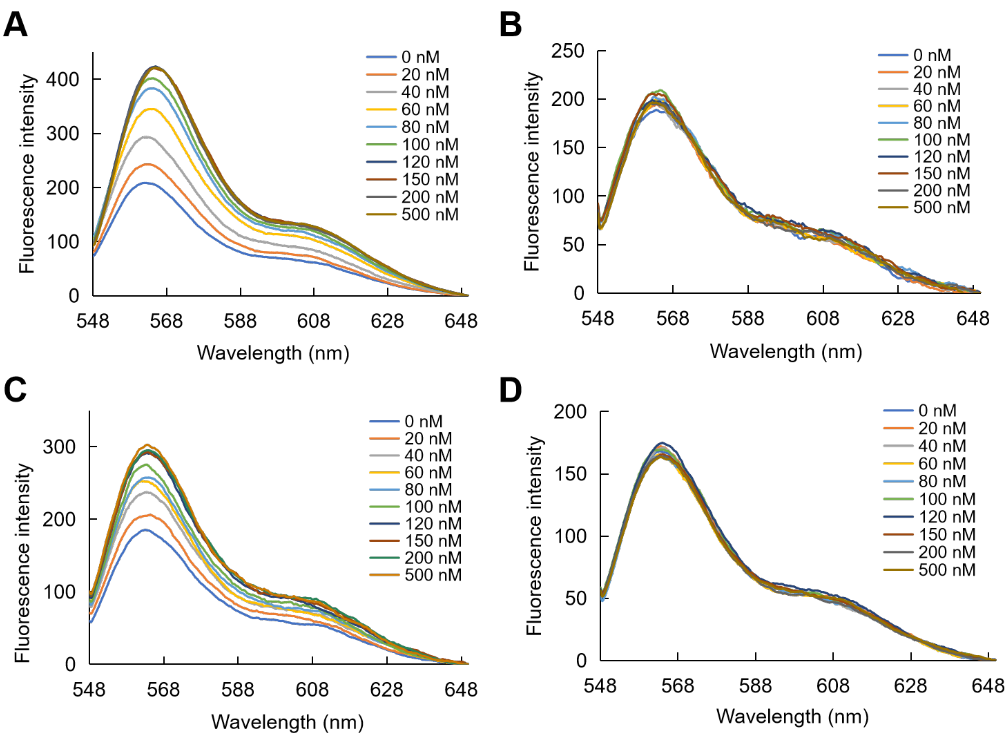

Figure S27. Direct comparison of the steady-state fluorescence properties of pol δ and pol I bound to various template-primer with Cy3 at the 5'-terminus of primer. (**A**) 100 nM DRH_{7-7} was subjected by 0-500 nM pol δ. (**B**) 100 nM DRH_{6-6} was subjected by 0-500 nM pol δ. (**C**) 100 nM DRH_{7-7} was subjected by 0-500 nM pol I. (**D**) 100 nM DRH_{6-6} was subjected by 0-500 nM pol I. The excitation wavelength was 540 nm and emitted at 548-648 nm in fluorescence spectroscopy.

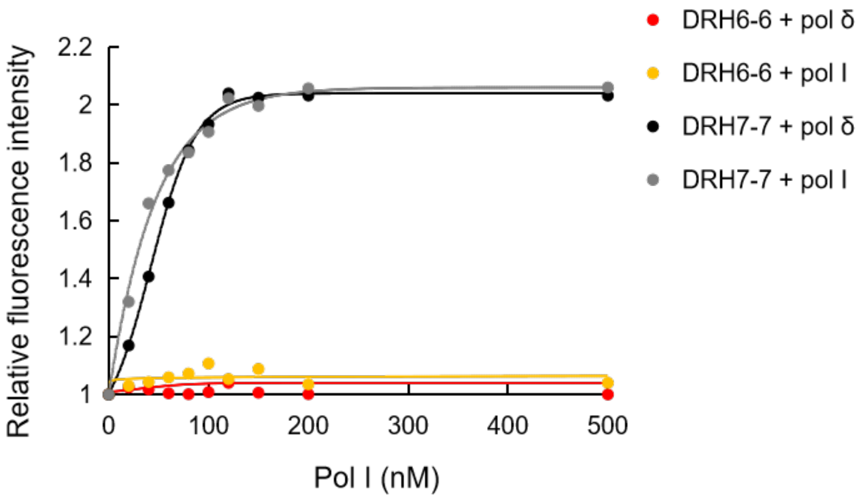

Figure S28. Equilibrium titrations of DRH_{6-6} and DRH_{7-7} with pol δ and pol I. A constant amount of template-primer (100 nM) with Cy3 at 5'-terminus of primer was titrated with increasing concentration of pol I. The fluorescence was excited at 540 nm and observed at 565 nm. Each measurement was repeated three times, and the average value of the fluorescence intensity was recorded. The analysis of the data yielded the dissociation constant K_d.

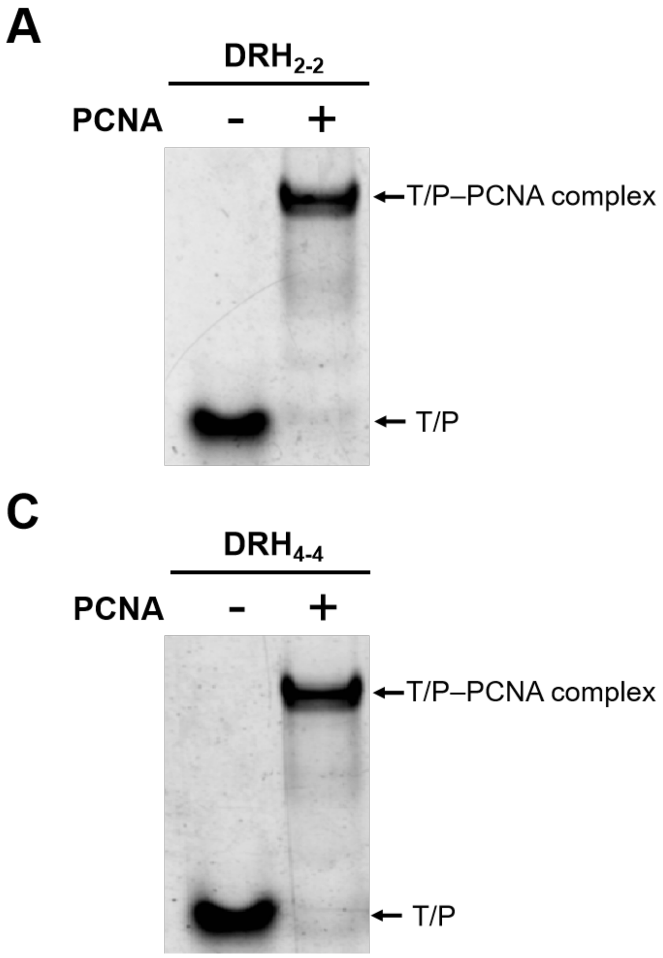

Figure S29. PCNA binding assay with DNA-RNA hybrids in non-denaturing polyacrylamide gel electrophoresis. **(A)** $DRH_{2\text{-}2}$ in absence or presence of PCNA. **(B)** $DRH_{1\text{-}1}$ in absence or presence of PCNA. **(C)** $DRH_{4\text{-}4}$ in absence or presence of PCNA. **(D)** $DRH_{3\text{-}3}$ in absence or presence of PCNA. T/P represents the DNA-template/RNA-primer used for DNA replication study. ^{m}C represent the hybrid including ^{m}C residue on DNA strand. All hybrids concentration is 1.0 nM, PCNA concentrations are 0, or 10 nM respectively.

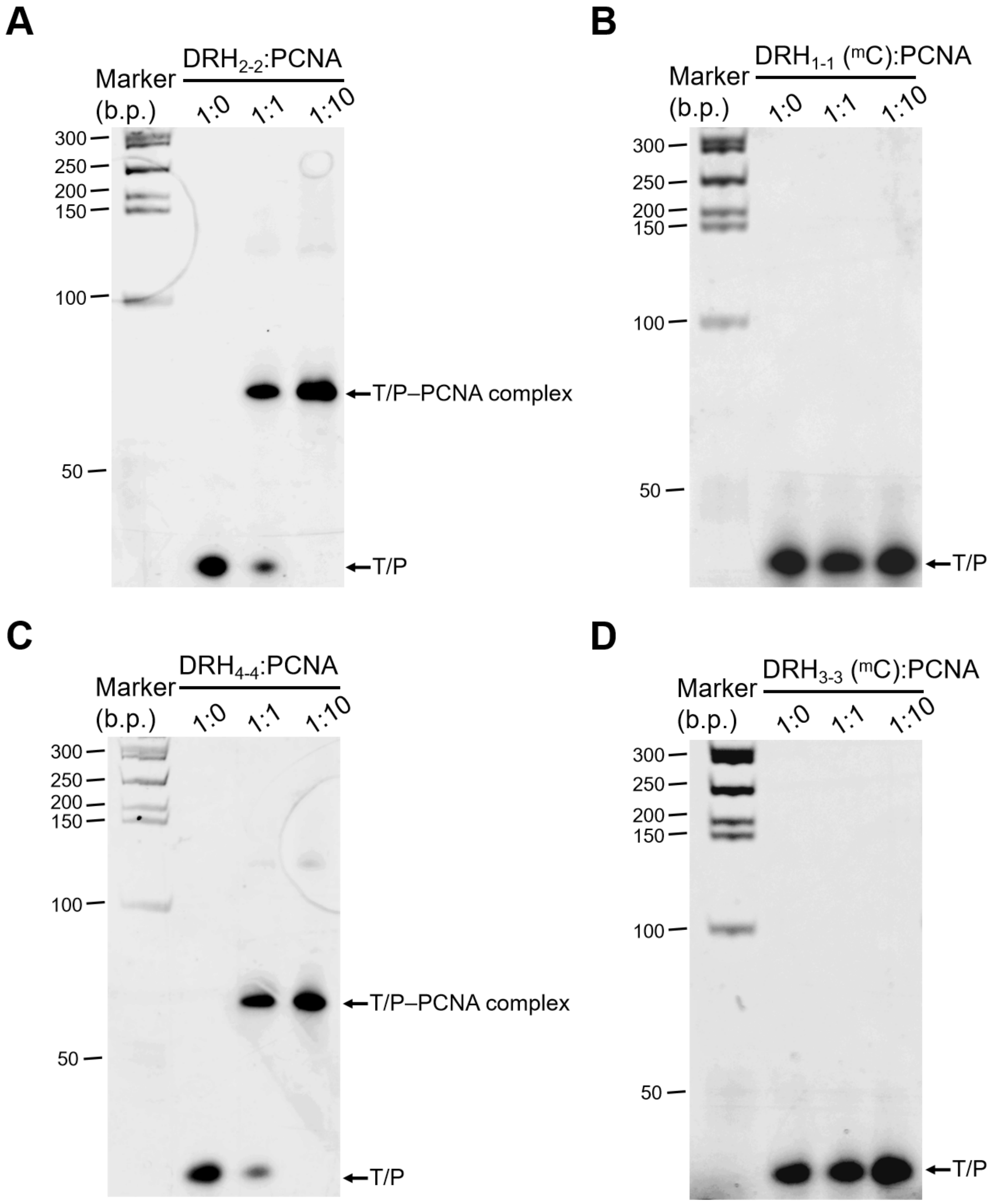

Figure S30. Titration assay of DNA-RNA hybrids with PCNA at different equivalences (1:0, 1:1 and 1:10) in non-denaturing polyacrylamide gel electrophoresis. **(A)** DRH$_{2-2}$ in different equivalences of pol δ. **(B)** DRH$_{1-1}$ in different equivalences of pol δ. **(C)** DRH$_{4-4}$ in different equivalences of pol δ. **(D)** DRH$_{3-3}$ in different equivalences of pol δ. T/P represents the DNA-template/RNA-primer used for DNA replication study. ^{m}C represent the hybrid including ^{m}C residue on DNA strand. Markers showing base pairs (b.p.) were used on the left side of gel. All hybrids concentration is 1.0 nM, PCNA concentrations are 0, 1 and 10 nM respectively.

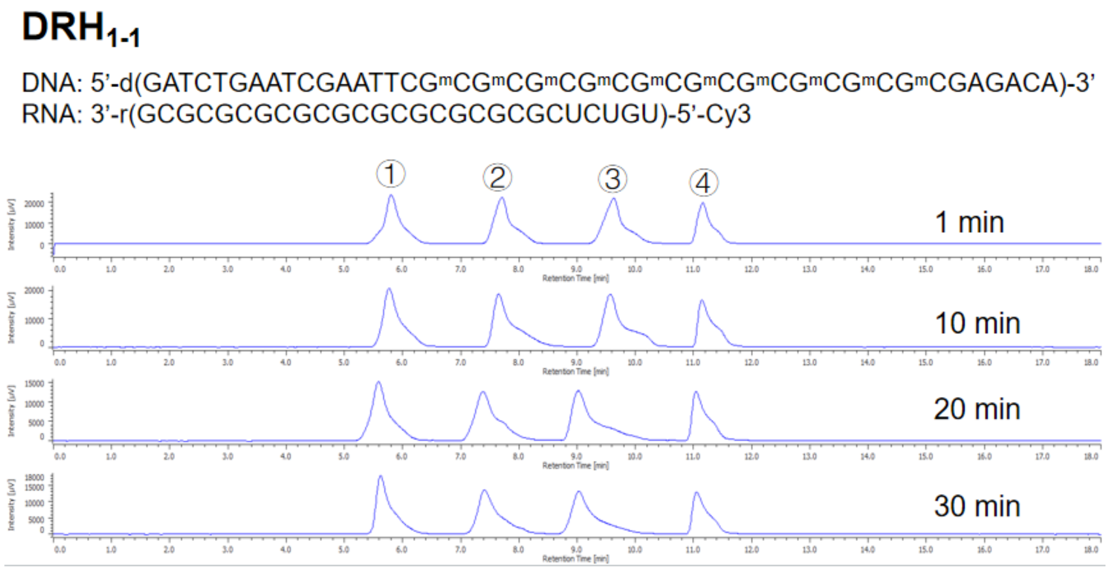

Figure S31. HPLC assay monitors primer extension products in DNA replication process in use of DRH$_{1\text{-}1}$ and pol δ in 25 mM MgCl$_2$. Reactiontimes were presented at right column. Starting material and production were labeling as numbers and characterized by MALDI-TOF MS. Peak ①: Cy3-5'-r(UGUCUCGCGCGCGCGCGCGCGCGCG)-3'/3'-d(ACAGAG^{m}CG^{m}CG^{m}CG^{m}CG^{m}CG^{m}CG^{m}CG^{m}CG^{m}CG^{m}CGCTTAAGCTAAGTCTAG)-5'; Peak ②: Cy3-5'-r(UGUCUCGCGCGCGCGCGCGCGCGCG)-3'; Peak ③: Cy3-5'-r(UGUCUCGCGCGCGCGCGCGCGCGCG)-3'/3'-r(GCGCGCGCGCGCGCGCGCGCGCUCUGU)-5';Peak ④: Cy3-5'-r(UGUCUCGCGCGCGCGCGCGCGCGCG)-3'/3'-r(GCGCGCGCGCGCGCGCGCGCGCUCUGU)-5'.

DRH$_{2\text{-}2}$

DNA: 5'-d(GATCTGAATCGAATTCGCGCGCGCGCGCGCGCGCGCGAGACA)-3'
RNA: 3'-r(GCGCGCGCGCGCGCGCGCGCGCUCUGU)-5'-Cy3

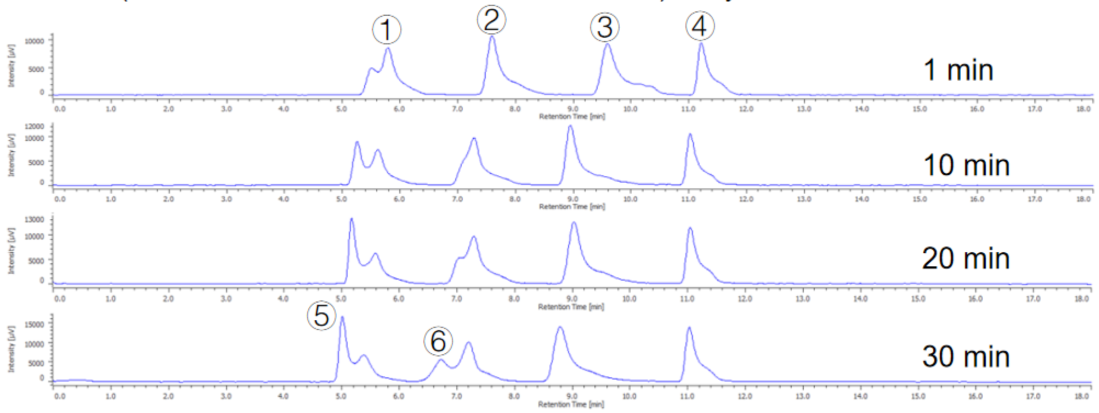

Figure S32. HPLC assay monitors primer extension products in DNA replication process i nuse of DRH$_{2\text{-}2}$ and pol δ in 25 mM $MgCl_2$. Reaction times were presented at right column. Starting material and production were labeling as numbers and characterized by MALDI-TOF MS. Peak ①: Cy3-5'-r(UGUCUCGCGCGCGCGCGCGCGCGCGCG)-3'/3'-d (ACAGAGCGCGCGCGCGCGCGCGCGCGCTTAAGCTAAGTCTAG)-5'; Peak ②: Cy3-5'-r (UGUCUCGCGCGCGCGCGCGCGCGCGCG)-3'; Peak ③: Cy3-5'-r(UGUCUCGCGCGCGC GCGCGCGCGCGCG)-3'/3'-r(GCGCGCGCGCGCGCGCGCGCGCUCUGU)-5'; Peak ④: Cy 3-5'-r(UGUCUCGCGCGCGCGCGCGCGCGCGCG)-3'/3'-r(GCGCGCGCGCGCGCGCGCGC GCUCUGU)-5'. Peak ⑤: Cy3-5'-r(UGUCUCGCGCGCGCGCGCGCGCGCGCG)d(AATTCG ATTCAGATC)-3'/3'-d(ACAGAGCGCGCGCGCGCGCGCGCGCGCTTAAGCTAAGTCTAG)-5'; Peak ⑥: Cy3-5'-r(UGUCUCGCGCGCGCGCGCGCGCGCGCG)d(AATTCGATTCAGATC) -3'. Red represent extended sequences from RNA primer in DNA replication reaction.

DRH$_{3-3}$

DNA: 5'-d(GGCGGGGCGCTGGGGGCGGTCG^{m}CG^{m}CG^{m}CG^{m}CGG^{m}CGG^{m}C^{m}CG^{m}CGA)-3'
RNA: 3'-r(GCGCGCGCGCCGCCGGCGCU)-5'-Cy3

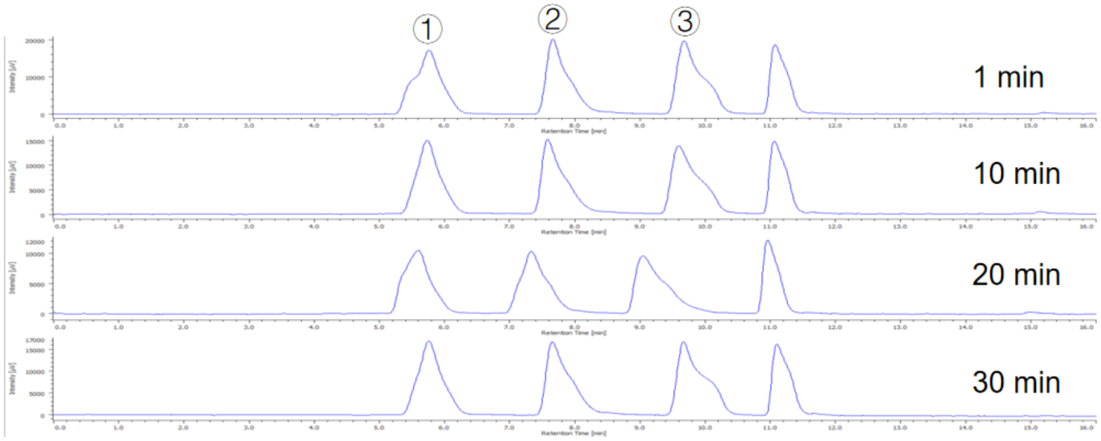

Figure S33. HPLC assay monitors primer extension products in DNA replication process in use of DRH$_{3-3}$ and pol δ in 25 mM $MgCl_2$. Reactiontimes were presented at right column. Starting material and production were labeling as numbers and characterized by MALDI-TOF MS. Peak ①: Cy3-5'-r(UCGCGGCCGCCGCGCGCGCG)-3'/3'-d(AG^{m}CG^{m}C^{m}CGG^{m}CGG^{m}CG^{m}CG^{m}CG^{m}CGCTGGCGGGGGTCGCGGGGCGG)-5'; Peak ②: Cy3-5'-r(UCGCGGCCGCCGCGCGCGCG)-3'; Peak ③: Cy3-5'-r(UCGCGGCCGCCGCGCGCGCG)-3'/3'-r(GCGCGCGCGCCGCCGGCGCU)-5'.

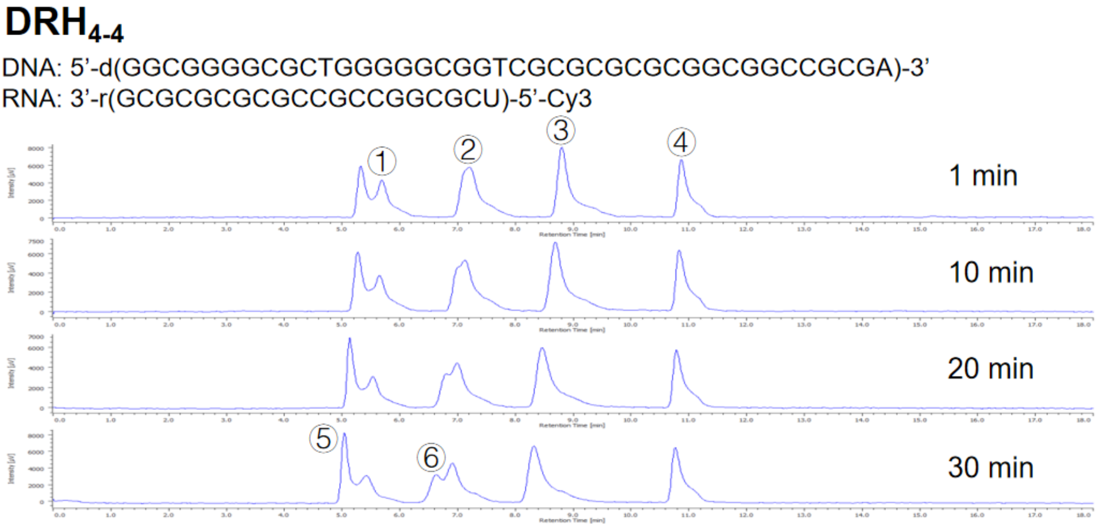

Figure S34. HPLC assay monitors primer extension products in DNA replication process use of DRH$_{4-4}$ and pol δ in 25 mM $MgCl_2$. Reaction times were presented at right column. Starting material and production were labeling as numbers and characterized by MALDI-TOF MS. Peak ①: Cy3-5'-r(UCGCGGCCGCCGCGCGCGCG)-3'/3'-d(AGCGCCGGCGGCGCGCGCGCTGGCGGGGGTCGCGGGGCGG)-5'; Peak ②: Cy3-5'-r(UCGCGGCCGCCGCGCGCGCG)-3'; Peak ③: Cy3-5'-r(UCGCGGCCGCCGCGCGCGCG)-3'/3'-r(GCGCGCGCGCCGCCGGCGCU)-5'; Peak ④: Cy3-5'-r(UCGCGGCCGCCGCGCGCGCG)-3'/3'-r(GCGCGCGCGCCGCCGGCGCU)-5'. Peak ⑤: Cy3-5'-r(UCGCGGCCGCCGCGCGCGCG)d(ACCGCCCCCAGCGCCCCGCC)-3'/3'-d(AGCGCCGGCGGCGCGCGCGCTGGCGGGGGTCGCGGGGCGG)-5'; Peak ⑥: Cy3-5'-r(UCGCGGCCGCCGCGCGCGCG)d(ACCGCCCCCAGCGCCCCGCC)-3'. Red represent extended sequences from RNA primer in DNA replicatio n reaction.

DRH$_{5-5}$

DNA: 5'-d(GGCGGGGCGCTGGGGGCGGTCG^{m}CG^{m}CG^{m}CG^{m}CGGCGGCCGCGA)-3'
RNA: 3'-r(GCGCGCGCGCCGCCGGCGCU)-5'-Cy3

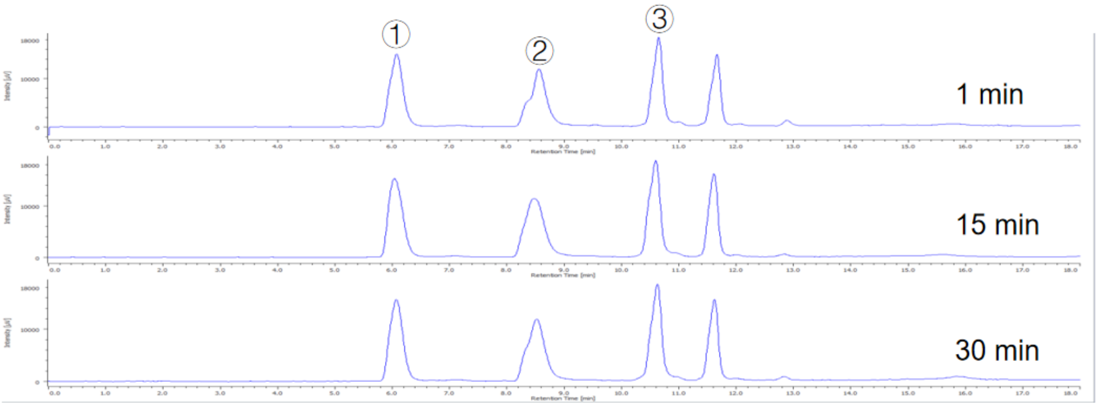

Figure S35. HPLC assay monitors primer extension products in DNA replication process inuse of DRH$_{5-5}$ and pol δ with ZBP1. Reaction times were presented at right column. Starting material were labeling as numbers and characterized by MALDI-TOF MS. Peak ①: Cy3-5'-r(UCGCGGCCGCCGCGCGCGCG)-3'/3'-d(AGCGCCGGCGG^{m}CG^{m}CG^{m}CG^{m}CGCTGGCGGGGGTCGCGGGGCGG)-5'; Peak ②: Cy3-5'-r(UCGCGGCCGCCGCGCGCGCG)-3'; Peak ③: Cy3-5'-r(UCGCGGCCGCCGCGCGCGCG)-3'/3'-r(GCGCGCGCGCCGCCGGCGCU)-5'.

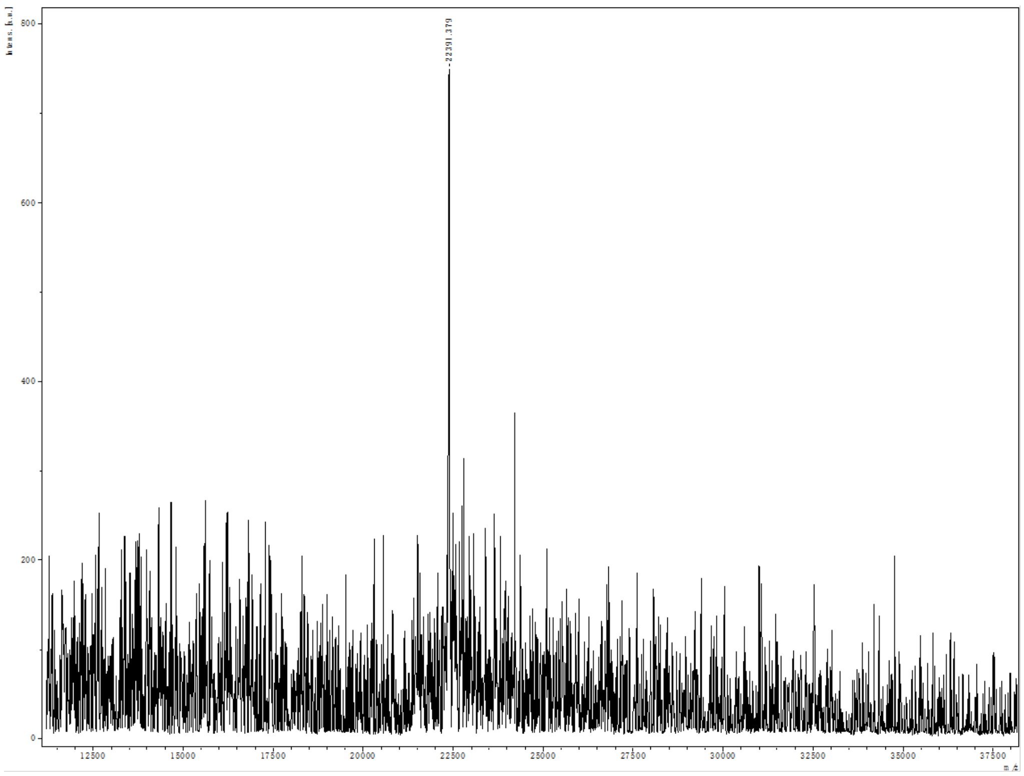

Figure S36. MALDI-TOF MS of Peak ①, Cy3-5'-r(UGUCUCGCGCGCGCGCGCGCGCGCGCG)-3'/3'-d(ACAGAG^{m}**C**G^{m}**C**G^{m}**C**G^{m}**C**G^{m}**C**G^{m}**C**G^{m}**C**G^{m}**C**G^{m}**C**G^{m}**C**GCTTAAGCTAAGTCTAG)-5' from **Figure S31.** Calcd. [M-H]$^{-}$: 22384.116; Found 22391.379.

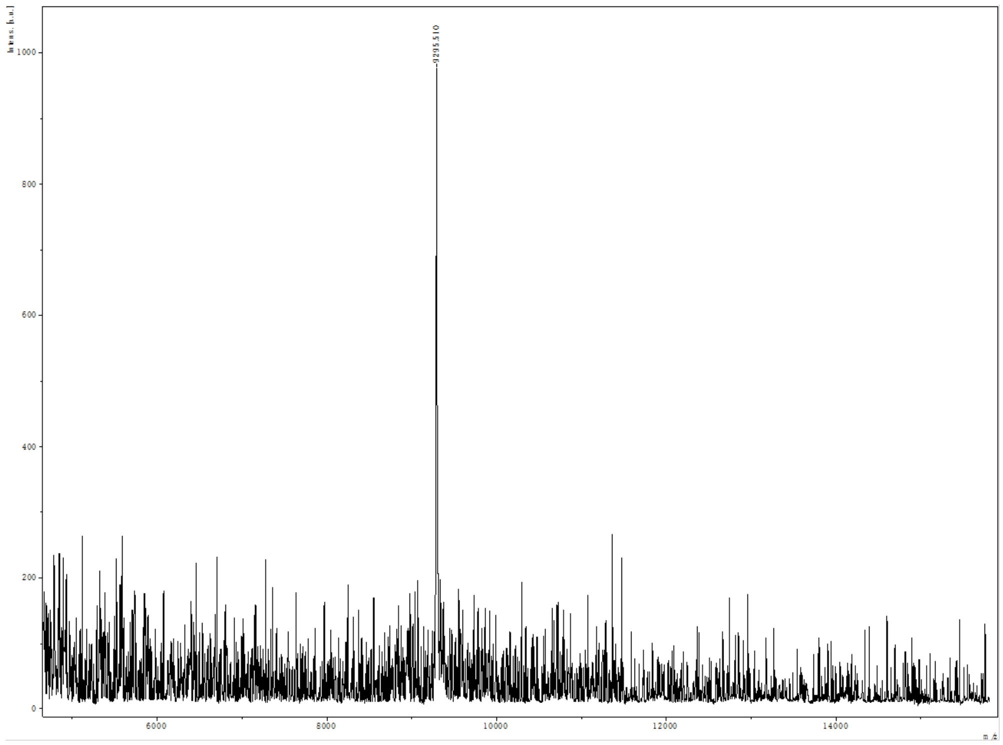

Figure S37. MALDI-TOF MS of Peak ②, Cy3-5'-r(UGUCUCGCGCGCGCGCGCGCGCGCGCG)-3' from **Figure S31.** Calcd. [M-H]$^{-}$: 9293.035; Found 9295.510.

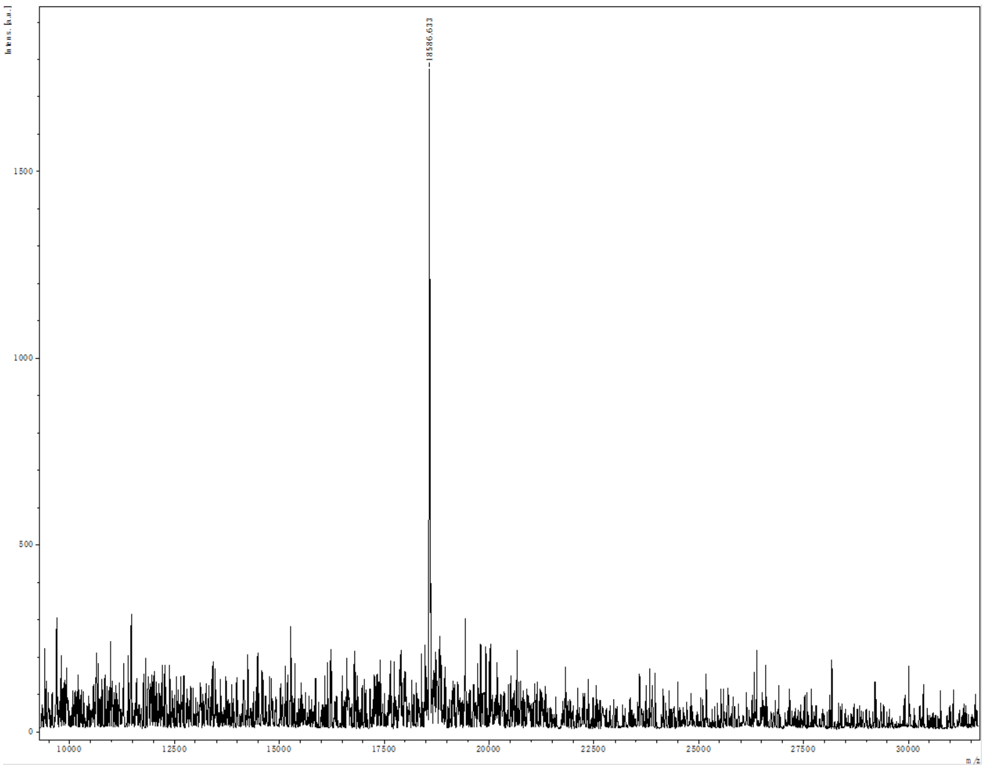

Figure S38. MALDI-TOF MS of Peak ③, Cy3-5’-r(UGUCUCGCGCGCGCGCGCGCGCGCGCG)-3’/3’-r(GCGCGCGCGCGCGCGCGCGCGCUCUGU)-5’ from **Figure S31.** Calcd. [M-H]$^-$: 18592.028; Found 18586.633.

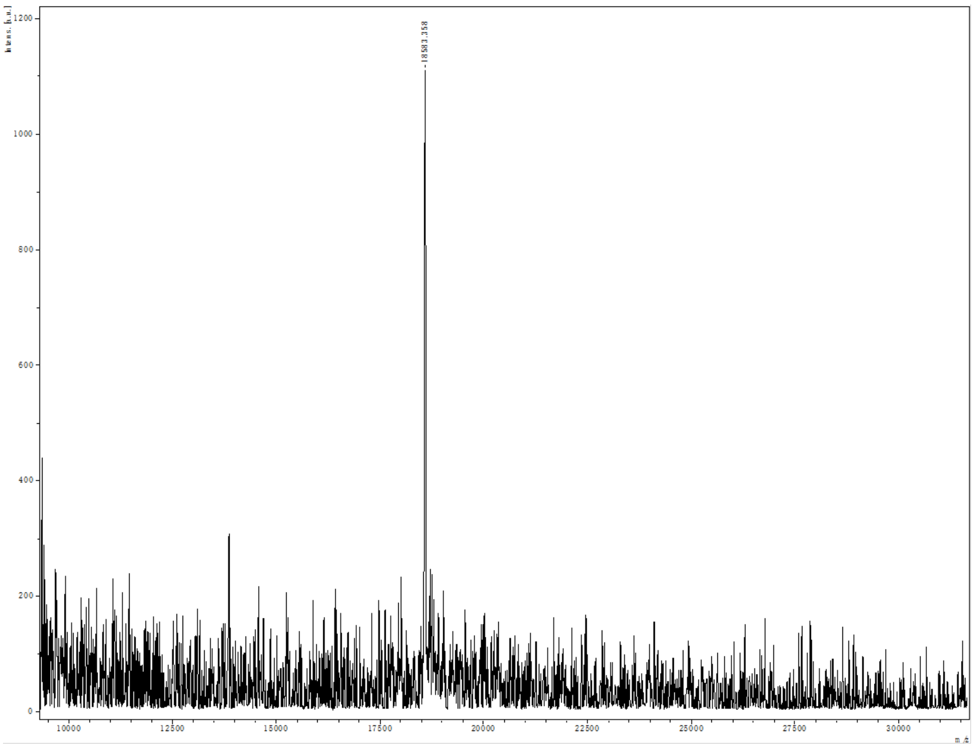

Figure S39. MALDI-TOF MS of Peak ④, Cy3-5’-r(UGUCUCGCGCGCGCGCGCGCGCGCGCG)-3’/3’-r(GCGCGCGCGCGCGCGCGCGCGCUCUGU)-5’ from **Figure S31.** Calcd. [M-H]$^-$: 18592.028; Found 18583.358.

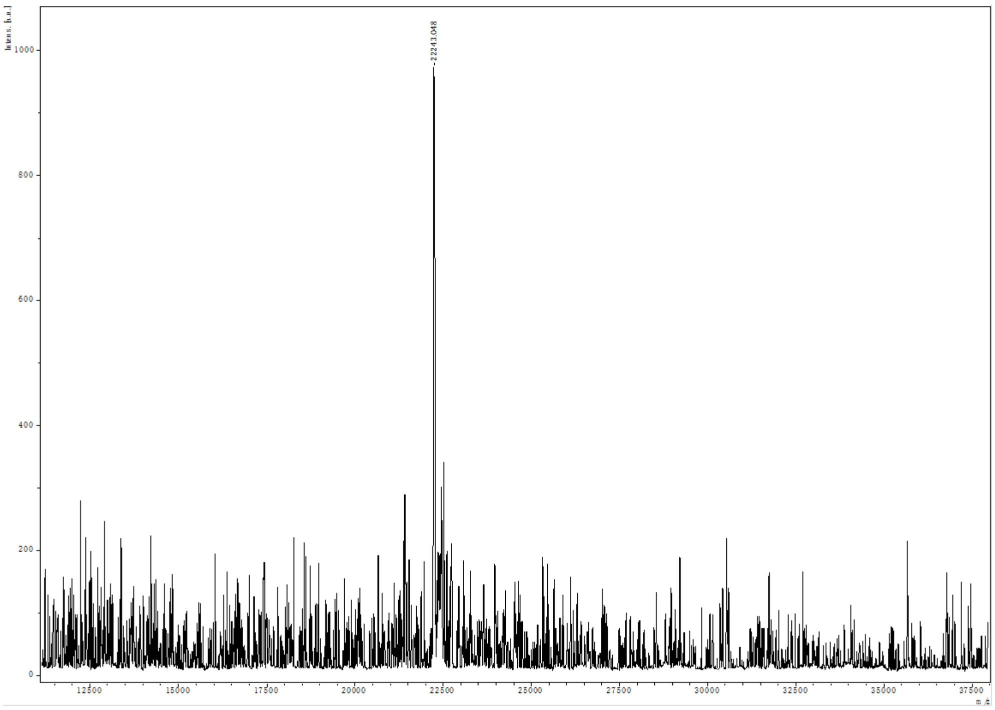

Figure S40. MALDI-TOF MS of Peak ①, Cy3-5’-r(UGUCUCGCGCGCGCGCGCGCGCGCGCG)-3’/3’-d(ACAGAGCGCGCGCGCGCGCGCGCGCTTAAGCTAAGTCTAG)-5’ from **Figure S32.** Calcd. [M-H]$^-$: 22246.696; Found 22243.048.

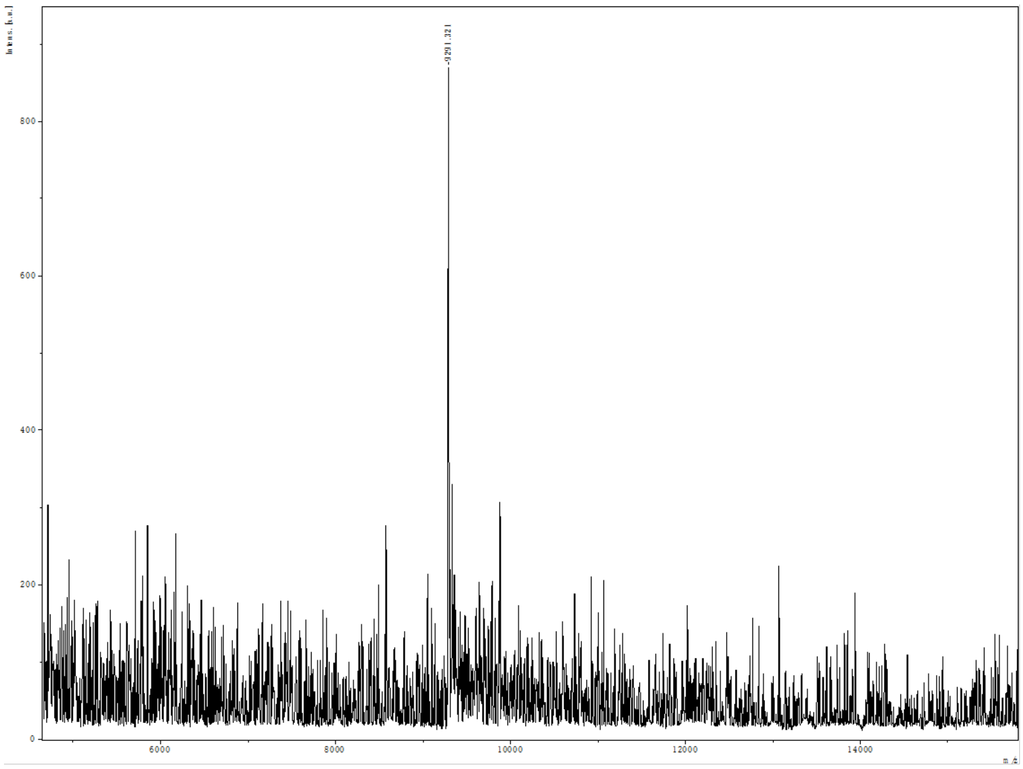

Figure S41. MALDI-TOF MS of Peak ②, Cy3-5’-r(UGUCUCGCGCGCGCGCGCGCGCGCGCG)-3’ from **Figure S32.** Calcd. [M-H]$^-$: 9293.035; Found 9291.321.

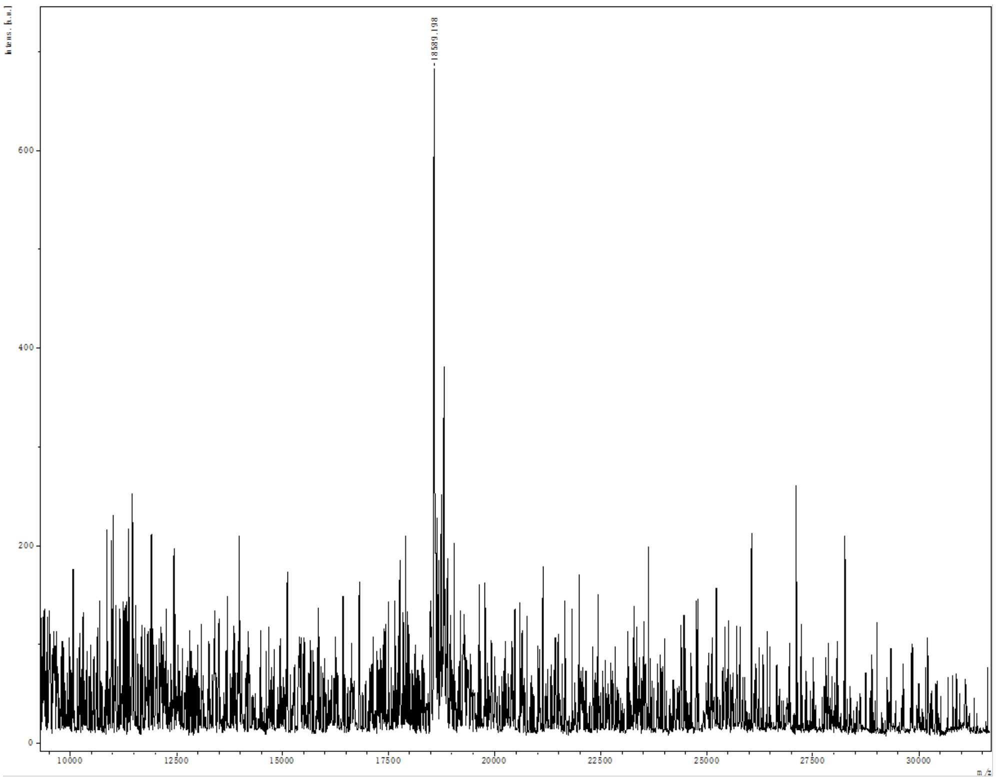

Figure S42. MALDI-TOF MS of Peak ③, Cy3-5’-r(UGUCUCGCGCGCGCGCGCGCG CGCG)-3’/3’-r(GCGCGCGCGCGCGCGCGCGCGCUCUGU)-5’ from **Figure S32.** Calcd. [M-H]$^-$: 18592.028; Found 18589.198.

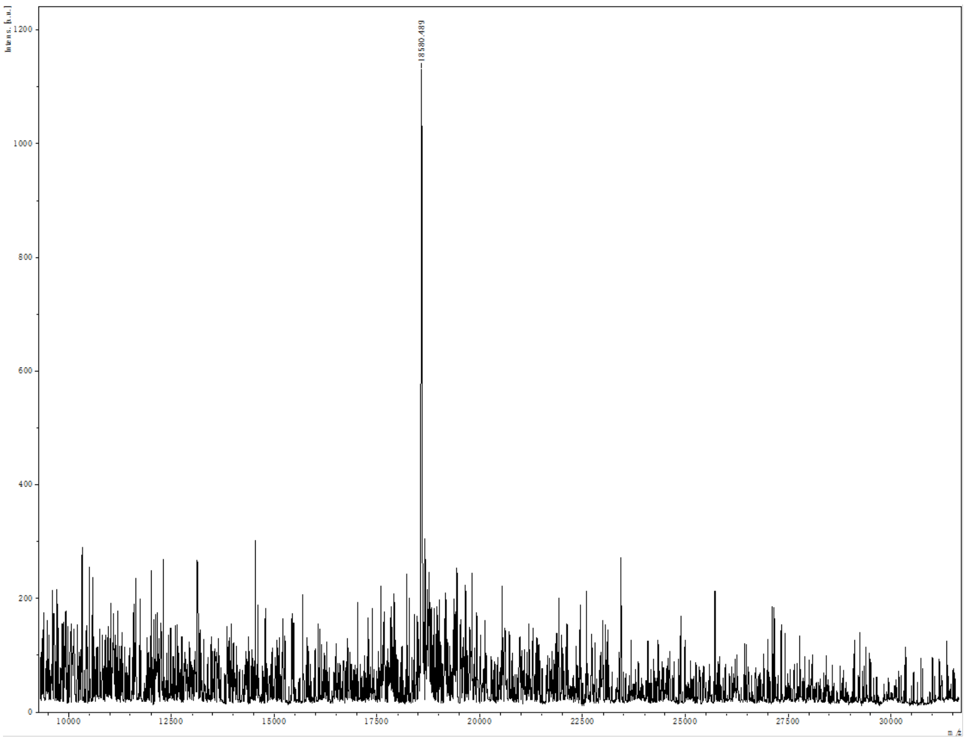

Figure S43. MALDI-TOF MS of Peak ④, Cy3-5’-r(UGUCUCGCGCGCGCGCGCGCG CGCG)-3’/3’-r(GCGCGCGCGCGCGCGCGCGCGCUCUGU)-5’ from **Figure S32.** Calcd. [M-H]$^-$: 18592.028; Found 18580.489.

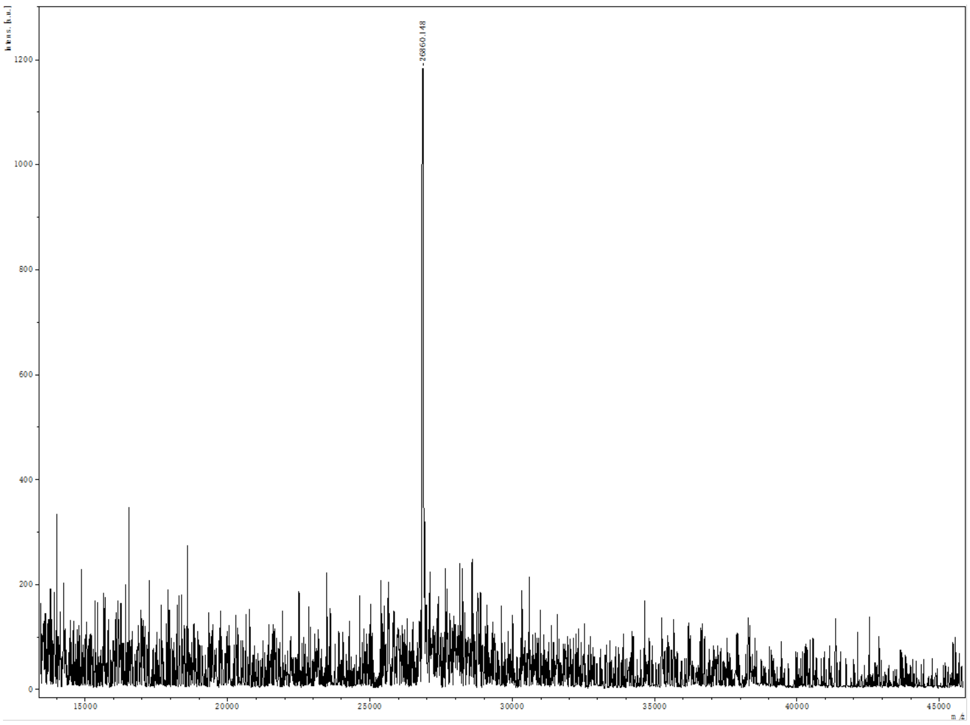

Figure S44. MALDI-TOF MS of Peak ⑤, Cy3-5’-r(UGUCUCGCGCGCGCGCGCGCGCGCGCG)d(AATTCGATTCAGATC)-3’/3’-d(ACAGAGCGCGCGCGCGCGCGCGCGCGCTTAAGCTAAGTCTAG)-5’ from **Figure S32.** Calcd. [M-H]$^{-}$: 26865.723; Found 26860.148.

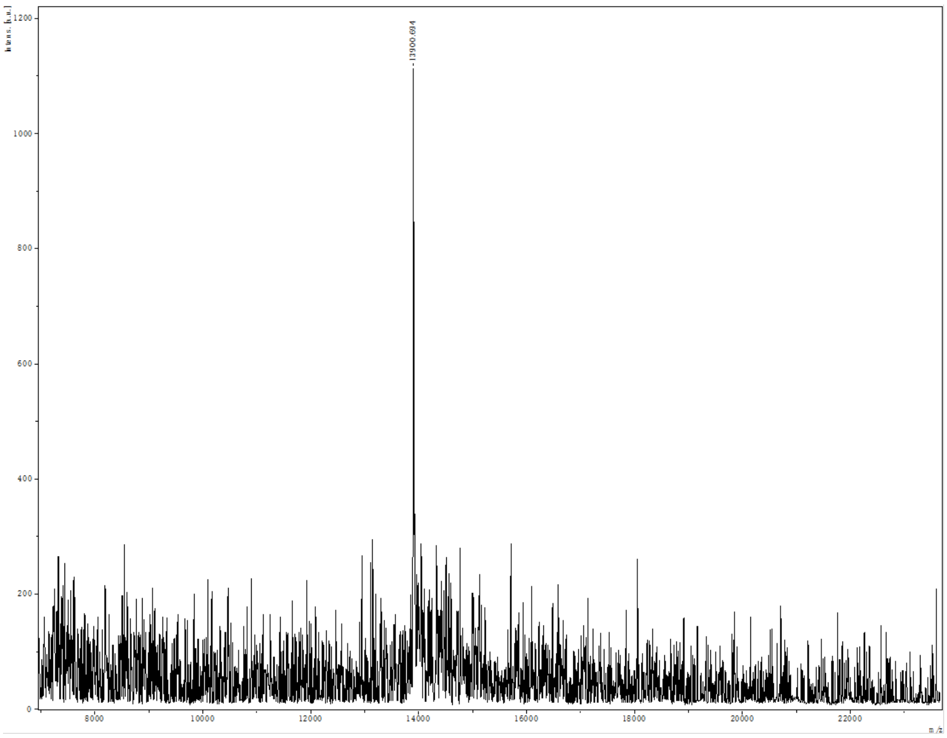

Figure S45. MALDI-TOF MS of Peak ⑥, Cy3-5’-r(UGUCUCGCGCGCGCGCGCGCGCGCGCG)d(AATTCGATTCAGATC)-3’ from **Figure S32.** Calcd. [M-H]$^{-}$: 13905.911; Found 13900.694.

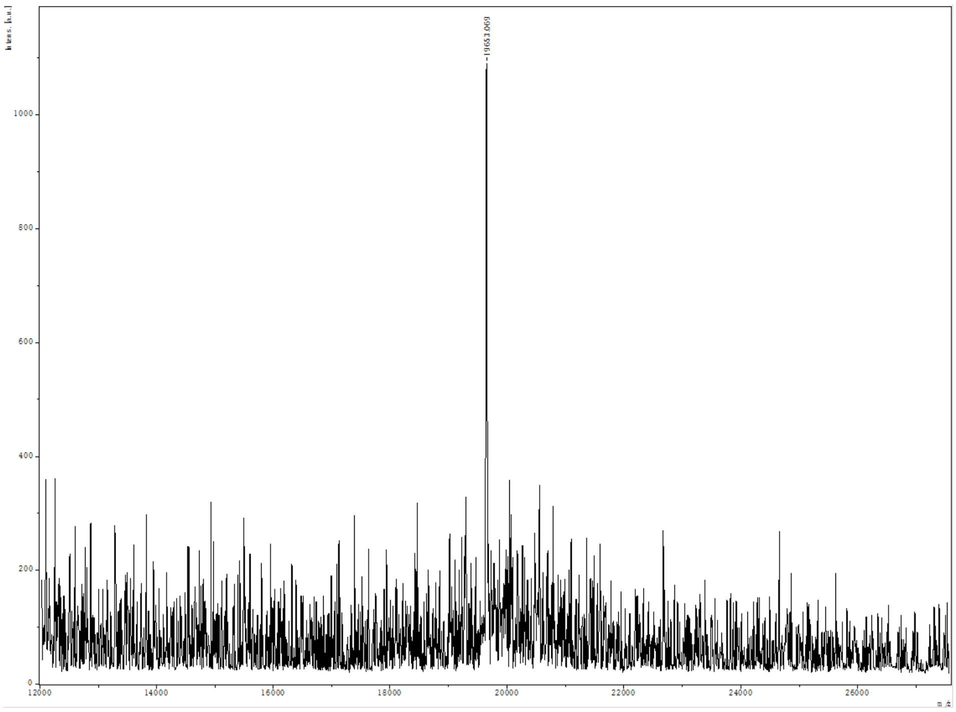

Figure S46. MALDI-TOF MS of Peak ①, Cy3-5'-r(UCGCGGCCGCCGCGCGCGCG)-3'/3'-d(AG^{m}**C**G^{m}**C**^{m}**C**GG^{m}**C**GG^{m}**C**G^{m}**C**G^{m}**C**G^{m}**C**GCTGGCGGGGGTCGCGGGGCGG)-5' from **Figure S33.** Calcd. [M-H]$^{-}$: 19656.827; Found 19653.069.

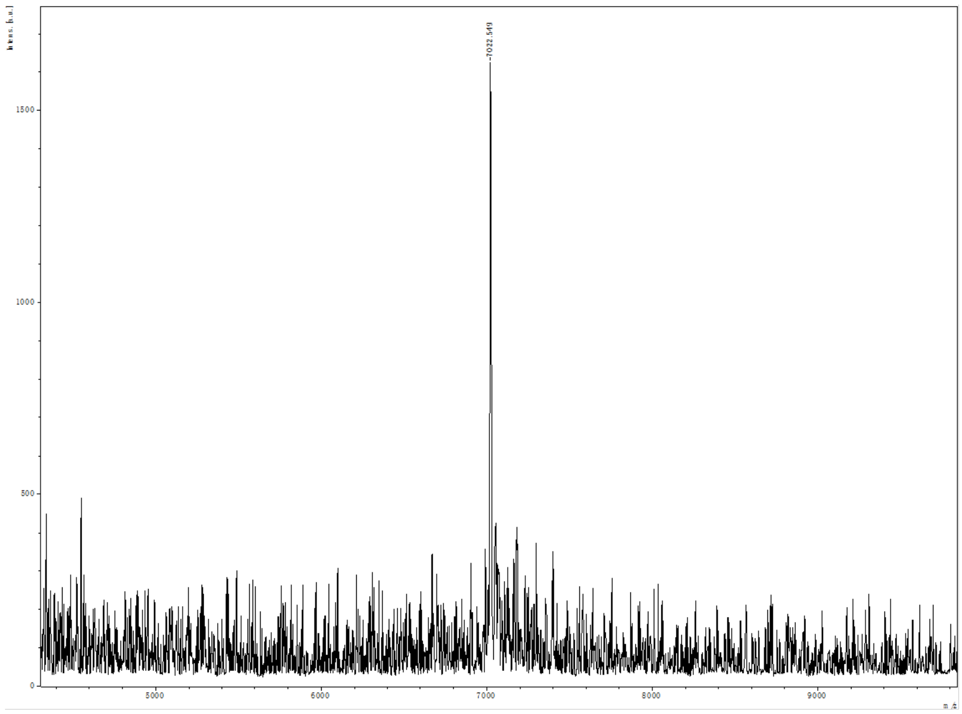

Figure S47. MALDI-TOF MS of Peak ②, Cy3-5'-r(UCGCGGCCGCCGCGCGCGCG)-3' from **Figure S33.** Calcd. [M-H]$^{-}$: 7025.226; Found 7022.549.

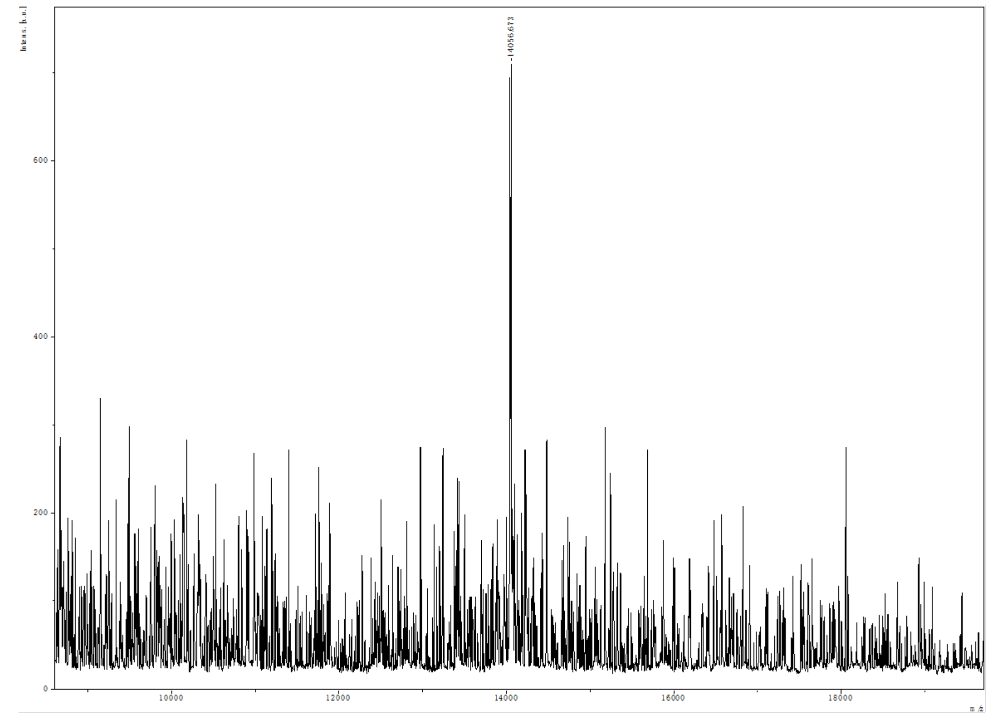

Figure S48. MALDI-TOF MS of Peak ③, Cy3-5’-r(UCGCGGCCGCCGCGCGCGCG)-3’/3’-r(GCGCGCGCGCCGCCGGCGCU)-5’ from **Figure S33.** Calcd. [M-H]$^-$: 14050.452; Found 14056.673.

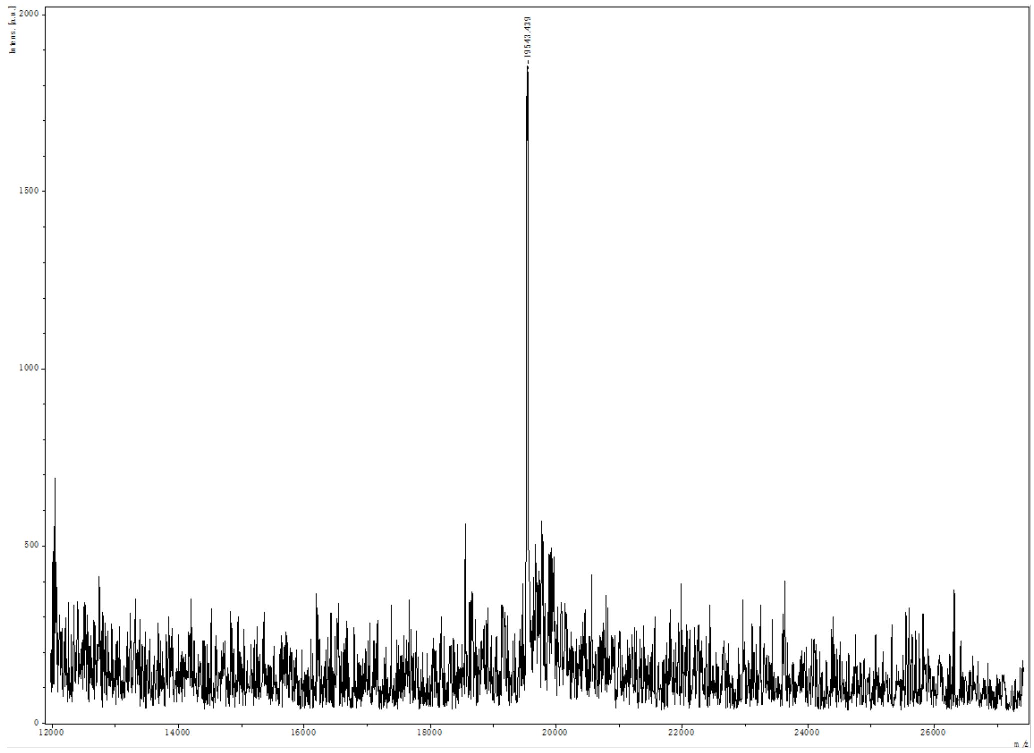

Figure S49. MALDI-TOF MS of Peak ①, Cy3-5’-r(UCGCGGCCGCCGCGCGCGCG)-3’/3’-d(AGCGCCGGCGGCGCGCGCGCTGGCGGGGTCGCGGGGCGG)-5’ from **Figure S34.** Calcd. [M-H]$^-$: 19547.025; Found 19543.439.

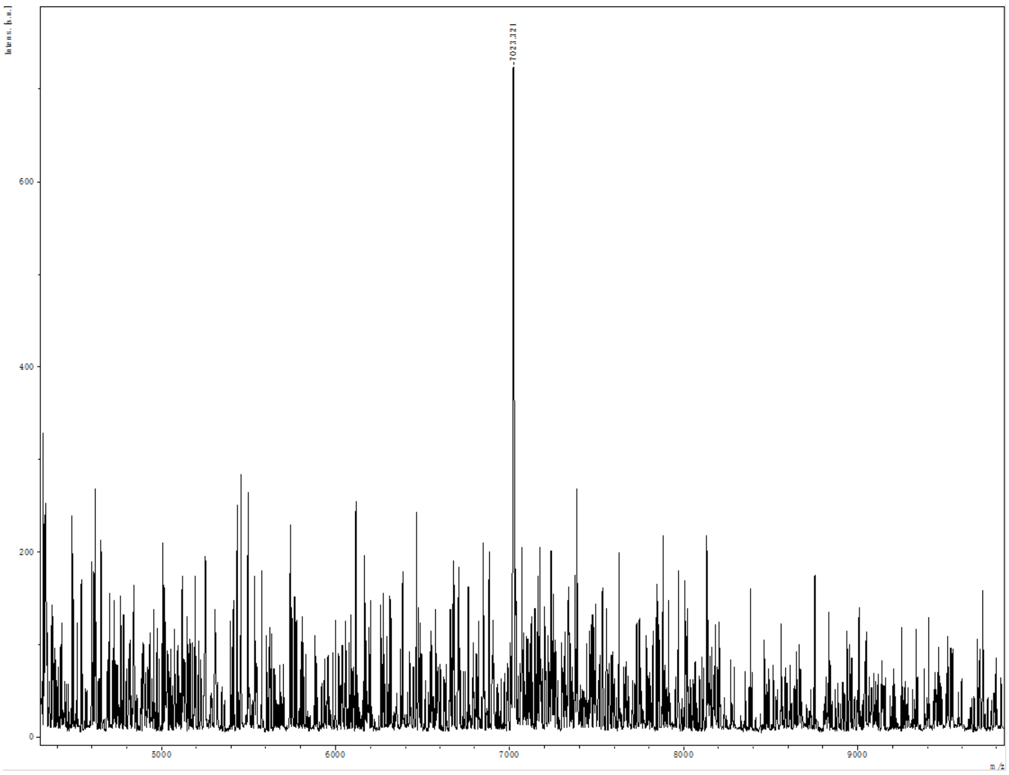

Figure S50. MALDI-TOF MS of Peak ②, Cy3-5'-r(UCGCGGCCGCCGCGCGCG)-3' from **Figure S34.** Calcd. [M-H]$^-$: 7025.226; Found 7023.321.

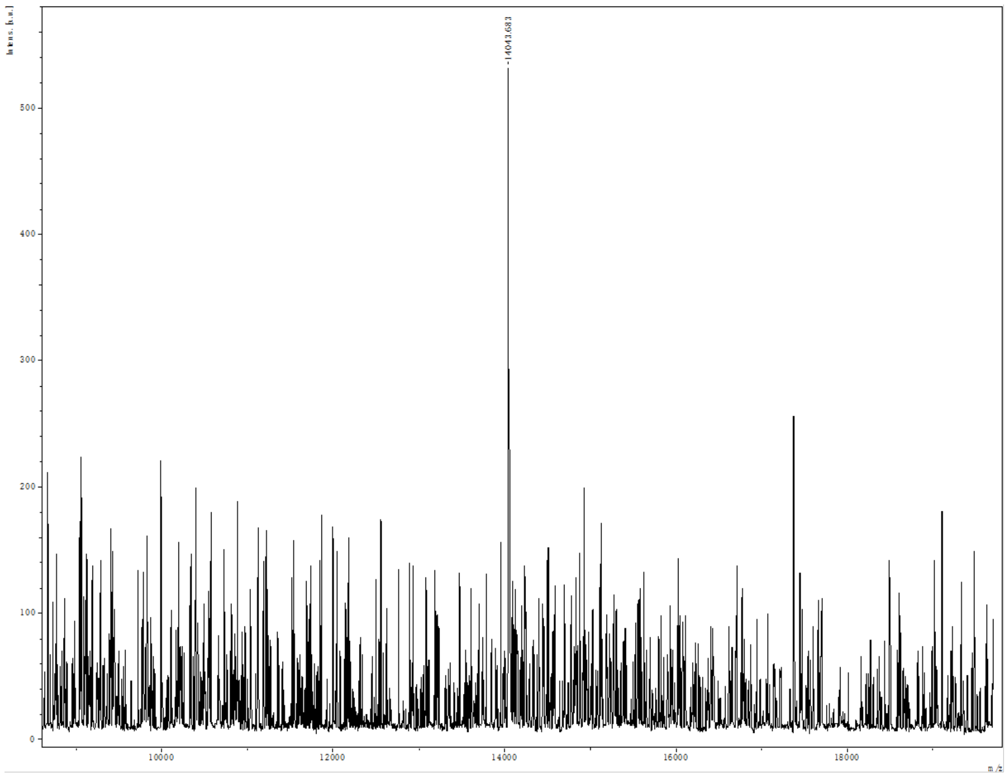

Figure S51. MALDI-TOF MS of Peak ③, Cy3-5'-r(UCGCGGCCGCCGCGCGCG)-3'/3'-r(GCGCGCGCGCCGCCGGCGCU)-5' from **Figure S34.** Calcd. [M-H]$^-$: 14050.452; Found 14043.683.

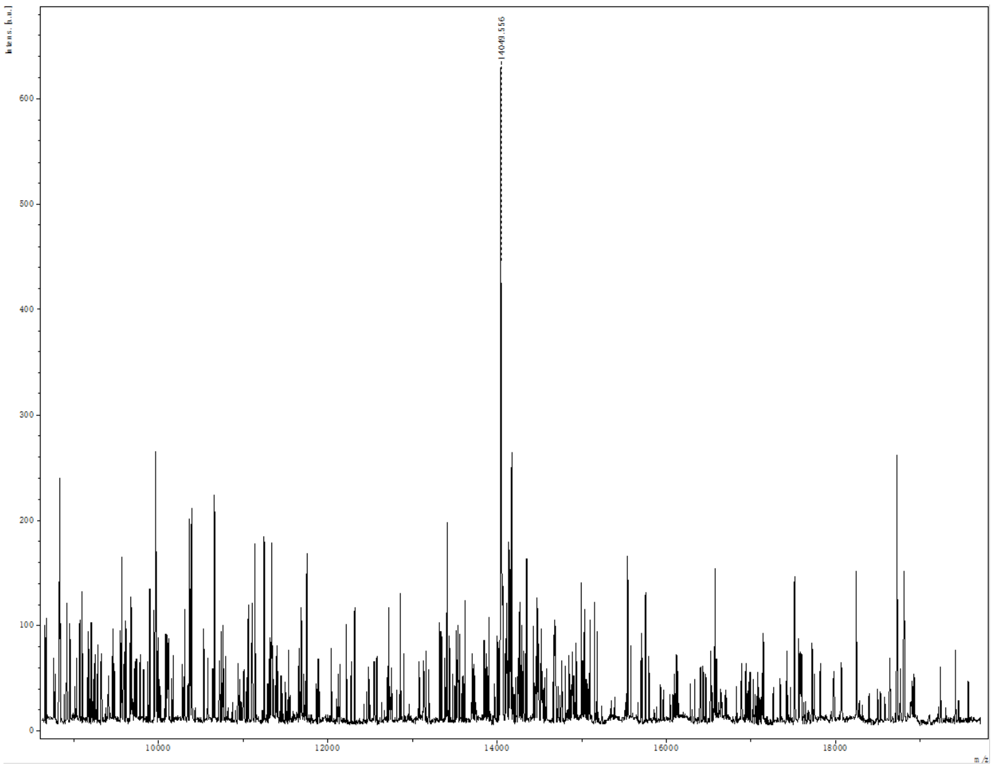

Figure S52. MALDI-TOF MS of Peak ④, Cy3-5'-r(UCGCGGCCGCCGCGCGCGCG)-3'/3'-r(GCGCGCGCGCCGCCGGCGCU)-5' from **Figure S34.** Calcd. [M-H]⁻: 14050.452; Found 14049.556.

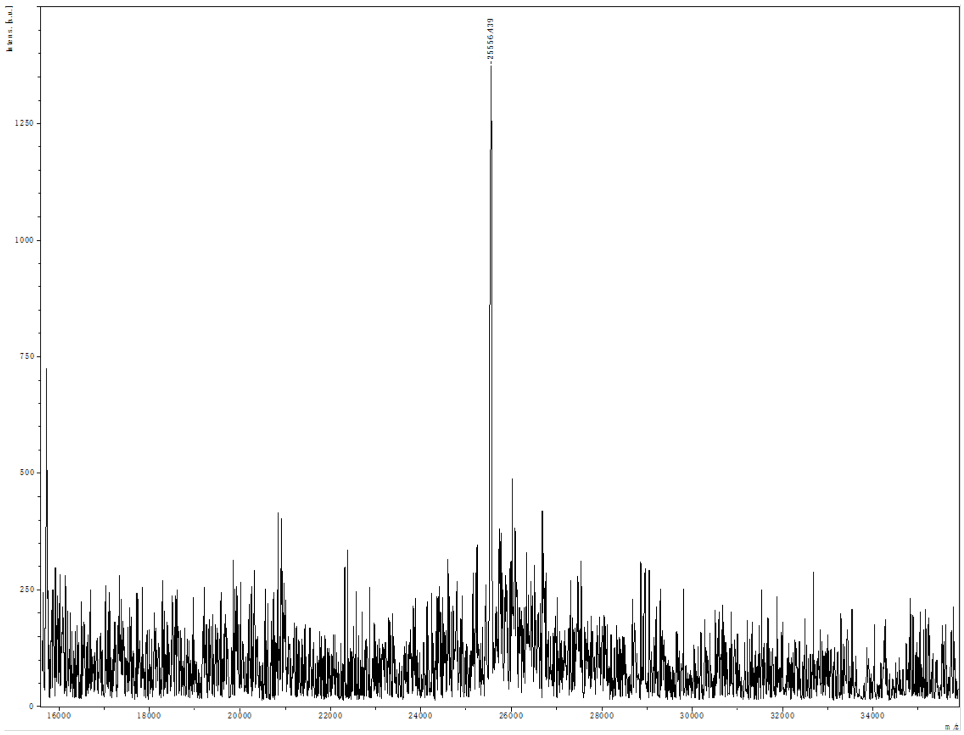

Figure S53. MALDI-TOF MS of Peak ⑤, Cy3-5'-r(UCGCGGCCGCCGCGCGCGCG)d(ACCGCCCCCAGCGCCCCGCC)-3'/3'-d(AGCGCCGGCGGCGCGCGCGCTGGCGGGGGTCGCGGGGCGG)-5' from **Figure S34.** Calcd. [M-H]⁻: 25559.334; Found 25556.439.

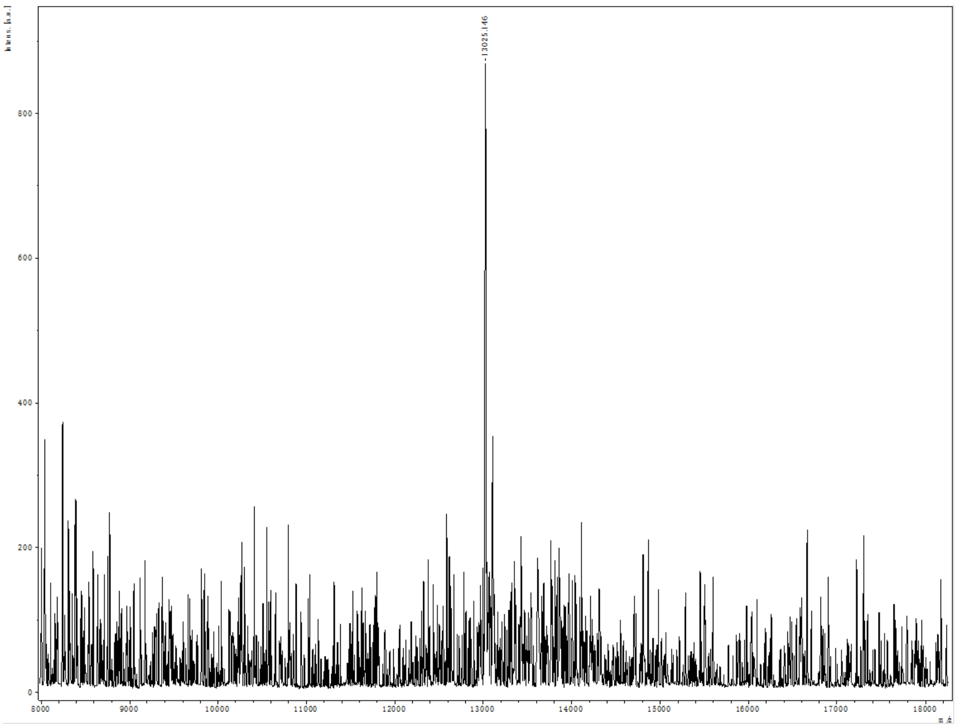

Figure S54. MALDI-TOF MS of Peak ⑥, Cy3-5’-r(UCGCGGCCGCCGCGCGCGCG)d(ACCGCCCCCAGCGCCCCGCC)-3’ from **Figure S34.** Calcd. [M-H]$^{-}$: 13029.668; Found 13025.146.

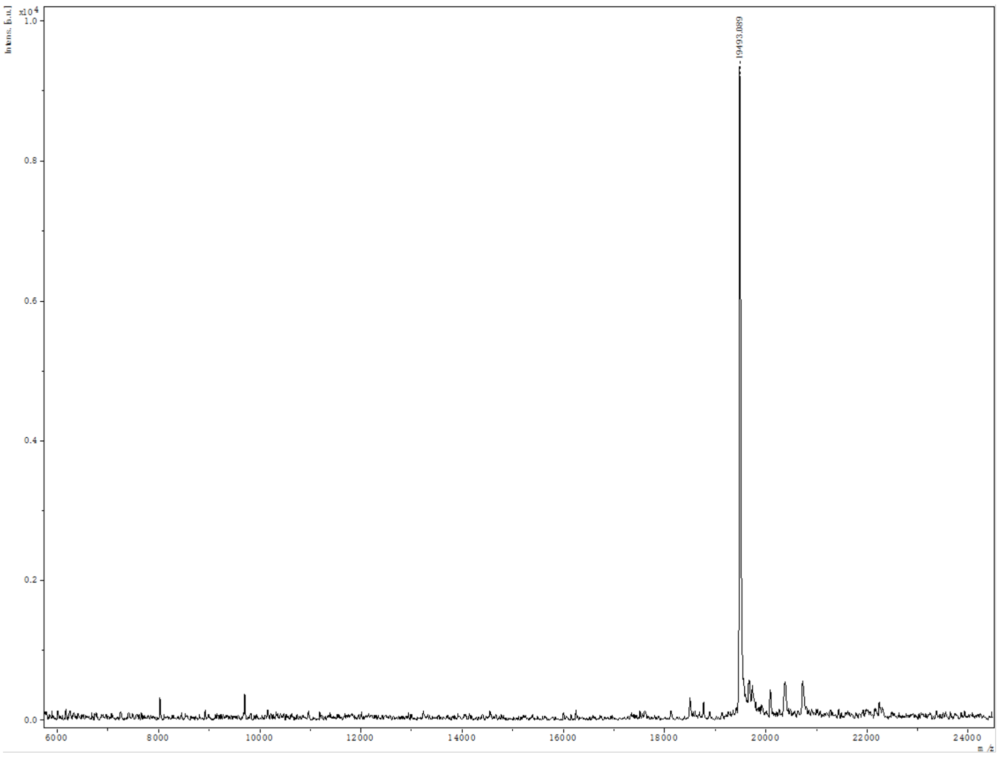

Figure S55. MALDI-TOF MS of Peak ①, Cy3-5’-r(UCGCGGCCGCCGCGCGCGCG)-3’/3’-d(AGCGCCGGCGG^{m}**C**G^{m}**C**G^{m}**C**G^{m}**C**GCTGGCGGGGTCGCGGGGCGG)-5’ from **Figure S35.** Calcd. [M-H]$^{-}$: 19496.628; Found 19493.089.

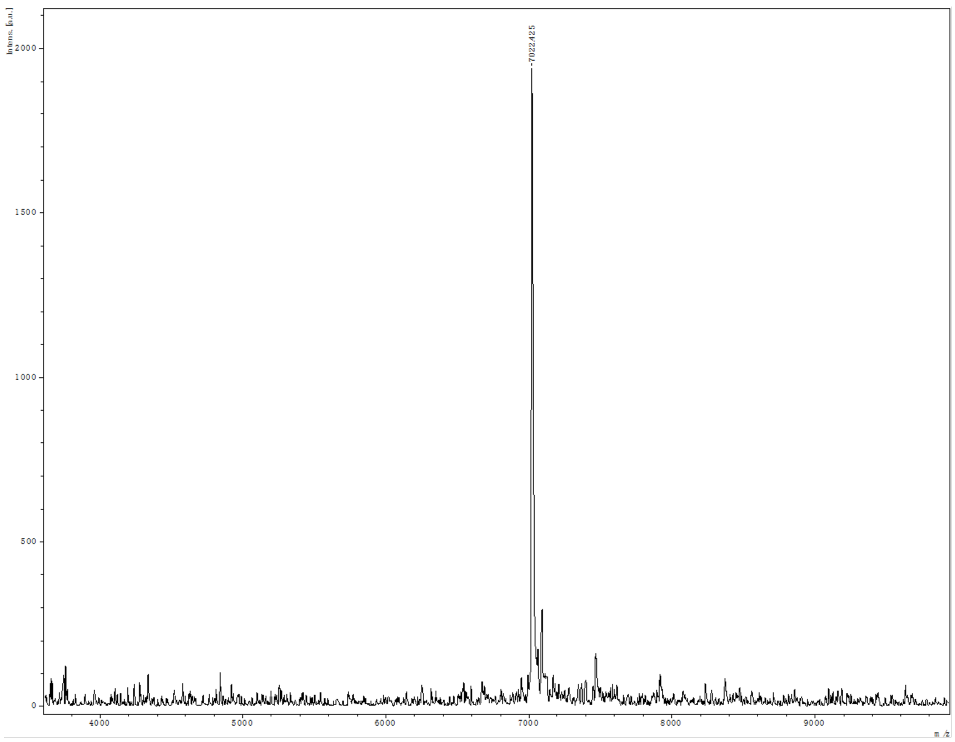

Figure S56. MALDI-TOF MS of Peak ②, Cy3-5'-r(UCGCGGCCGCCGCGCGCGCG)-3' from **Figure S35.** Calcd. $[M-H]^-$: 7025.226; Found 7022.425.

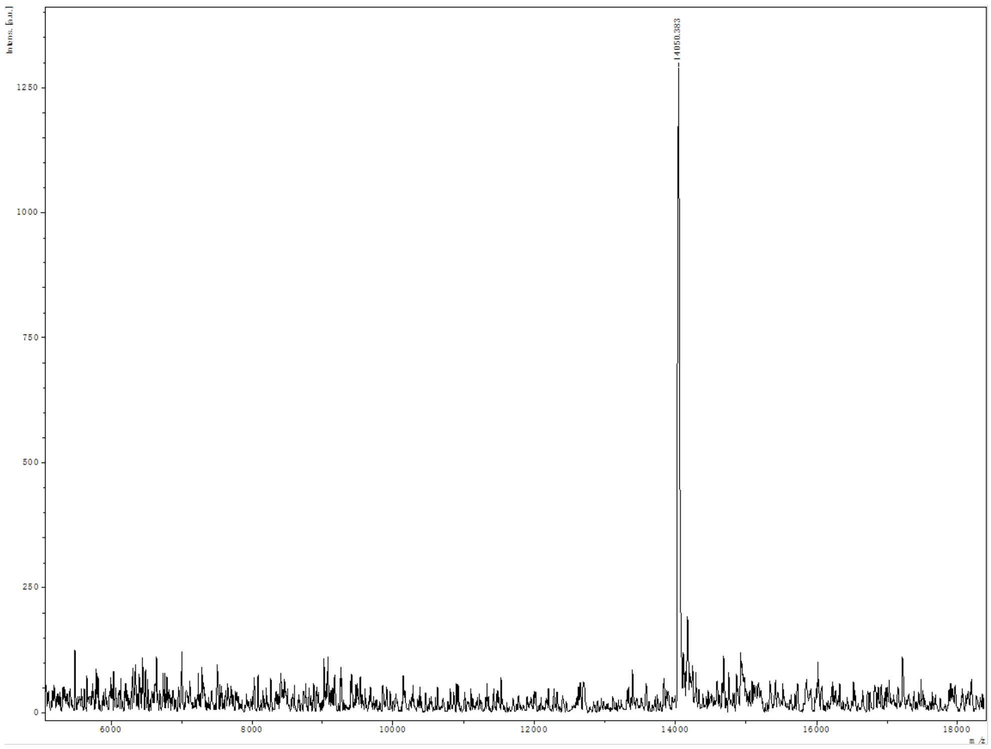

Figure S57. MALDI-TOF MS of Peak ③, Cy3-5'-r(UCGCGGCCGCCGCGCGCGCG)-3'/3'-r(GCGCGCGCGCCGCCGGCGCU)-5' from **Figure S35.** Calcd. $[M-H]^-$: 14050.452; Found 14050.383.

Chemicals. All reagents were commercial and from Aldrich, TCI (Tokyo Chemical Industry) or Wako (Wako Pure Chemical Industries) and used in the absence of extra purification unless especial emphasis. All experiments containing air and/or moisture sensitive reagents were performed within an Ar environment. Thin layer chromatography (TLC) was performed using TLC Silica gel 60 F254 (Merck). Compounds were visualized using a UV lamp (254 nm) or staining with a potassium permanganate solution. Silica gel (Wakogel® C-300, 200–325 mesh) was used for column chromatography. The middle pressure liquid chromatography (MPLC) system was conducted from EPCLC-AI-580S (Yamazen Corporation, Japan) equipped with silica gel column (Hi-Flash Column, Yamazen Corporation). ^{1}H NMR (400 MHz), ^{19}F NMR (100 MHz) and ^{31}P NMR (162 MHz) spectra were recorded on a BRUKER (AV-400M) magnetic resonance spectrometer. DMSO-d_6 and $CDCl_3$ were employed as the solvents. Coupling constants (J) values are indicated in Hz and corrected to within 0.5 Hz. Signal patterns are indicated as s, singlet; d, doublet; t, triplet; q, quartet; m, multiplet. High-resolution mass spectra (HRMS) were recorded by electrospray ionization (ESI) on a electrospray ionization (ESI) on an Exactive Orbitrap mass spectrometer instrument (Thermo Scientific). MALDI-TOF mass spectra were produced on an autoflexⅢ TOF mass spectrometer (Bruker Daltonics, Billerica, MA, USA).

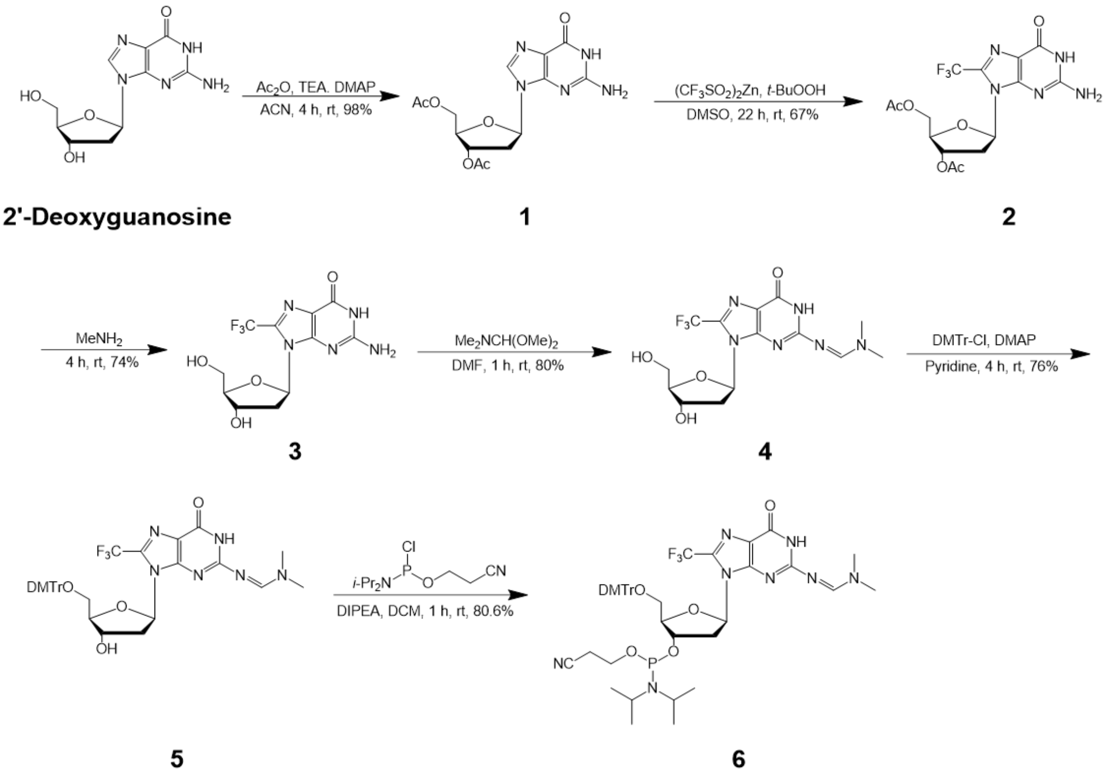

Scheme S1. Synthetic scheme of 8-trifluoromethyl-2'-deoxyguanosine phosphoramidite

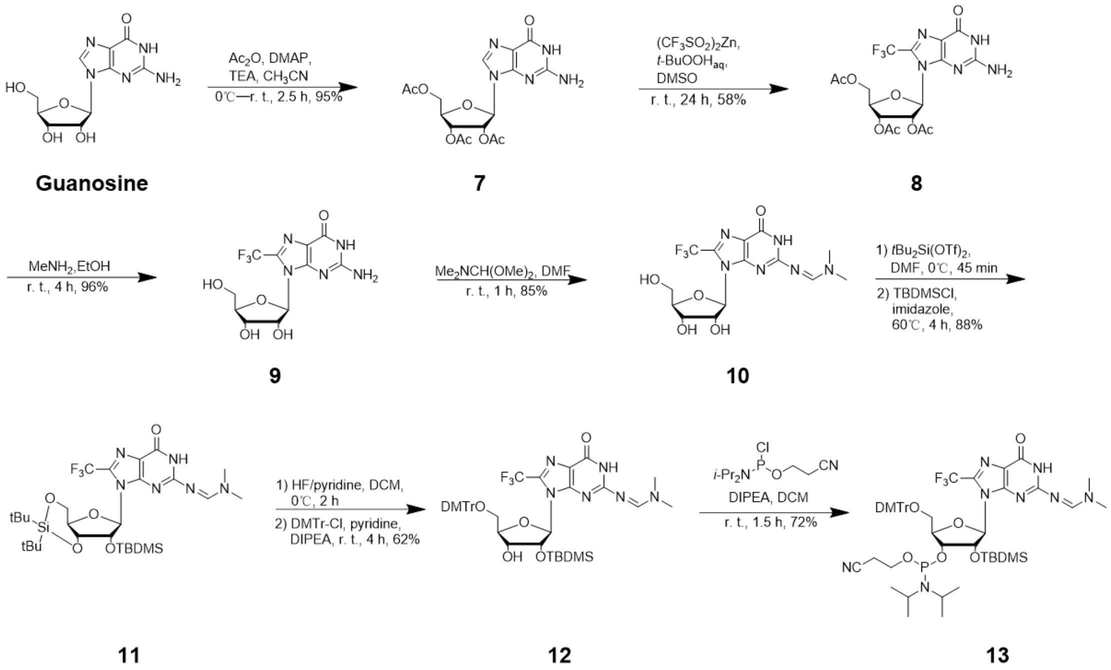

Scheme S2. Synthetic scheme of 8-trifluoromethylguanosine phosphoramidite.

3',5'-Di-*O*-acetyl-2'-deoxyguanosine (1). 2'-Deoxyguanosine (1.5 g, 5.61 mmol) was co-evaporated with 5 mL anhydrous acetonitrile for three times and dissolved in 20 mL anhydrous acetonitrile. Next, triethylamine (6.3 mL, 44.9 mmol), 4-dimethylaminopyridine (68 mg, 0.561 mmol, 0.1 equiv.) was added and the acetic a nhydride (1.24 mL, 13 mmol) also added in dropwise at 0 °C. The reaction was did at 0 °C for 1 hour and additional three hours at room temperature. Methanol in 4 mL was added to quench the reaction. The mixture was concentrated to one third volume and followed by additional 100 mL cooling ethyl ether. The precipitation was collected after filtration and dissolved in 20 mL acetone with stirring at 50 °C for t wo hours. The product was given through filtration as a white solid (1.94 g, 98%). ^{1}H NMR (400 MHz, DMSO-d$_6$) δ 7.93 (s, 1H), 6.52 (s, 1H), 6.15 (dd, J = 4.2 Hz, 1H), 5.31 (dd, J = 3.6 Hz, 1H), 4.30-4.17 (m, 3H), 2.96-2.89 (m, 1H), 2.49-2.05 (m, 1H), 2.10-2.05 (m, 12H); HRMS (ESI) for $C_{14}H_{16}N_5O_6$ [M-H]$^-$: Calcd. 350.1179; Fou nd. 350.1099; HRMS (ESI) for $C_{14}H_{17}N_5O_6Cl$ [M+Cl]$^-$: Calcd. 386.1047; Found. 386.0 867; HRMS (ESI) for $C_{15}H_{18}N_5O_8$ [M+HCOO]$^-$: Calcd. 396.0957; Found. 396.1152.

3',5'-Di-*O*-acetyl-8-trifluoromethyl-2'-deoxyguanosine (2).
3',5'-Di-*O*-acetyl-2'-deoxyguanosine (1.22 g, 3.5 mmol) was dissolved in 20 mL dimethyl sulfoxide and followed by additional Zinc(II) trifluoromethanesulfinate (3.5 g, 10.5 mmol). Tert-butyl hydroperoxide (70% aqueous, total 2.27 mL, 17.5 mmol) was added in dropwise with 10 aliqouts in 20 min intervals. The reaction was consisted as 24 hours at room temperature and extracted using 200 mL dichloromethane with 300 mL water and 100 mL brine. The organic layer was collected and dried over using sodium sulfate. The filtrate was collected after filtration and evaporated *in vacuo*. The residue was purified using MPLC

(chloroform:methanol = 10:1) and gave a white solid in 980 mg (67%). ^{1}H NMR (400 MHz, DMSO-d$_6$) δ 11.09 (s, 1H), 6.55 (d, *J* = 4.4 Hz, 1H), 6.21 (dd, *J* = 3.6 Hz, 1H), 5.48-5.44 (m, 1H), 4.27-4.15 (m, 3H), 3.42 (t, *J* = 2.8 Hz, 1H), 2.36 (t, *J* = 2.8 Hz, 1H), 2.09-2.01 (m, 12H); ^{19}F NMR (372 MHz, DMSO-d$_6$) δ -59.977 (s, 3F); HRMS (ESI) for $C_{15}H_{16}F_3N_5O_6$ $[M+Na]^+$: Calcd. 442.1053; Found. 442.1244.

8-Trifluoromethyl-2'-deoxyguanosine (3).
3',5'-Di-*O*-acetyl-8-trifluoromethyl-2'-deoxyguanosine (980 mg, 2.33 mmol) was dissolved in methylamine in ethanol solution (33%, 12.2 mL, 98 mmol) and reacted for 4 hours at room temperature. The mixture was concentrated *in vacuo* and purified through MPLC (dichloromethane:methanol = 4:1) to gave a white solid as 580 mg (74%). ^{1}H NMR (400 MHz, DMSO-d$_6$) δ 11.00 (s, 1H), 6.70 (s, 1H), 6.15 (t, *J* = 6.0 Hz, 1H), 5.29 (d, *J* = 4.4 Hz, 1H), 4.88 (t, *J* = 6.4 Hz, 1H), 4.44 (d, *J* = 2.8 Hz, 1H), 3.85 (dt, *J* = 7.2 Hz, 1H), 3.67-3.50 (m, 2H), 3.16 (dd, *J* = 2.0 Hz, 1H), 2.18 (dd, *J* = 2.0 Hz, 1H); ^{19}F NMR (372 MHz, DMSO-d$_6$) δ -60.012 (s, 3F); HRMS (ESI) for $C_{11}H_{11}F_3N_5O_4$ $[M-H]^-$: Calcd. 334.0841; Found. 334.0716.

***N*^{2}-Dimethylformamidyl-8-trifluoromethyl-2'-deoxyguanosine (4).**
8-Trifluoromethyl-2'-deoxyguanosine (580 mg, 1.73 mmol) was co-evaporated using 5 mL acetonitrile for three times and dissolved in 5 mL anhydrous dimethylformamide. The *N,N*-dimethylformamide dimethyl acetal (1.7 mL, 12.11 mmol) was added and reacted for 1 hour at room temperature. The mixture was concentrated in evaporator and purified via MPLC (dichloromethane:methanol = 5:1) to gave a white solid as 540 mg (80%). ^{1}H NMR (400 MHz, DMSO-d$_6$) δ 11.74 (s, 1H), 8.55 (s, 1H), 6.21 (t, *J* = 1.2 Hz, 1H), 5.38 (d, *J* = 4.2 Hz, 1H), 4.86 (dd, *J* = 2.4 Hz, 1H), 4.50 (dd, *J* = 1.2 Hz, 1H), 3.87 (dd, *J* = 1.6 Hz, 1H), 3.68-3.55 (m, 2H), 3.20-3.08 (m, 5H), 2.23 (dd, *J* = 1.6 Hz, 1H); ^{19}F NMR (372 MHz, DMSO-d$_6$) δ -60.113 (s, 3F); HRMS (ESI) for $C_{14}H_{16}F_3N_6O_4$ $[M-H]^-$: Calcd. 389.1263; Found. 389.1184; HRMS (ESI) for $C_{14}H_{17}F_3N_6O_4Cl$ $[M+Cl]^-$: Calcd. 425.0748; Found. 425.0925; HRMS (ESI) for $C_{15}H_{18}F_3N_6O_6$ $[M+HCOO]^-$: Calcd. 435.0976; Found. 435.1236.

***N*^{2}-Dimethylformamidyl-8-trifluoromethyl-5'-O-(4,4'-dimethoxytrityl)-2'-deoxyguanosine (5).** *N*^{2}-Dimethylformamidyl-8-trifluoromethyl-2'-deoxyguanosine (540 mg, 1.38 mmol) was co-evaporated with anhydrous pyridine in 5 mL for three times and dissolved in 5 mL pyridine. 4-Dimethylaminopyridine (17 mg, 0.138 mmol) and 4,4'-dimethoxytrityl chloride (705 mg, 2.07 mmol) were added. The reaction was did for 4 hours at room temperature and followed by additional 5 mL methanol quenching the reaction. The mixture was extracted in 50 mL dichloromethane and 25 mL sodium bicarbonate. The organic layer was collected and dried over with anhydrous sodium sulfate. The filtrate was obtained after filtration and concentrated in vacuum to gave a solid residue. The crude compound was purified using MPLC (dichloromethane:methanol = 20:1) to gave a white foam in 726 mg (76%). ^{1}H NMR (400 MHz, DMSO-d$_6$) δ 11.72 (s, 1H), 8.32 (s, 1H), 7.42-7.13 (m, 15H), 6.79-6.72 (m, 5H), 6.32 (dd, *J* = 3.6 Hz, 1H), 5.42 (d, *J* = 1.6 Hz, 1H), 4.64 (dd, *J* = 3.6 Hz, 1H), 3.95 (dt, *J* = 3.6 Hz, 1.6H), 3.73-3.69 (m, 6H), 3.26-3.18 (m, 3H), 3.03 (t, *J* = 1.6 Hz,

6H), 2.35 (dt, J = 2.4 Hz, 1H); ^{19}F NMR (372 MHz, DMSO-d$_6$) δ -59.947 (s, 3F); HRMS (ESI) for $C_{35}H_{35}F_3N_6O_6Na$ [M+Na]$^+$: Calcd. 715.2570; Found. 715.2446.

3'-*O*-[(2-Cyanoethoxy)(diisopropylamino)phosphino]-*N*^{2}-dimethylformamidyl-8-trifluoromethyl-5'-*O*-(4,4'-dimethoxytrityl)-2'-deoxyguanosine (6). The compound **9** (720 mg, 1.05 mmol) was co-evaporated with anhydrous acetonitrile in 5 mL for three times and dissolved in 8 mL anhydrous dichloromethane. The *N,N*-diisopropylethylamine (0.76 mL, 4.2 mmol) was added and stirred for 10 min at room temperature. Next, 2-cyanoethyl-*N,N*-diisopropylamidochlorophosphoramidite (0.5 mL, 2.1 mmol) was added in dropwise and reacted for 1 hour at room temperature. The mixture was extracted using 50 mL dichloromethane with 25 mL 5% sodium bicarbonate and 25 mL brine. The organic layer was collected and dried over using sodium sulfate. The filtrate was given through filtration and concentrated *in vacuo*. The crude compound was purified using MPLC (dichloromethane:methanol:triethylamine= 20:1:0.1) and gave a white foam as product (755 mg, 80.6%). ^{1}H NMR (400 MHz, DMSO-d$_6$) δ 11.78 (s, 1H), 8.38 (s, 1H), 7.40-7.11 (m, 12H), 6.80-6.74 (m, 5H), 6.36 (dd, J = 4.8 Hz, 1H), 5.11-4.98 (m, 1H), 4.09-4.01 (m, 7H), 3.73-3.69 (m, 9H), 3.51-2.47 (m, 14H), 1.21-0.93 (m, 40H); ^{19}F NMR (372 MHz, DMSO-d$_6$) δ -59.987, -60.001 (s, 3F); ^{31}P NMR (161 MHz, DMSO-d$_6$) δ 148.3746; HRMS (ESI) for $C_{44}H_{51}F_3N_8O_7P$ [M-H]$^-$: Calcd. 891.3649; Found. 891.3561.

2',3',5'-Tri-*O*-acetylguanosine (7). Guanosine (0.75 g, 2.6 mmol), trimethylamine (2.9 mL, 20.7 mmol) and 4-dimethylaminopyridine (34.5 mg, 0.28 mmol) were dissolved in 10.1 mL dehydrated acetonitrile, followed by that acetic anhydride (0.825 mL, 8.3 mmol) was added dropwise and the mixture reacted for 1.5 hours at 0 °C and additional 1 hour at room temperature. The reaction was stopped by adding methanol (0.86 mL, 21.34 mmol). The volume was evaporated to 1/3 using a vacuum pump and diethyl ether with appropriate volume was added to get precipitation. The product was collected by filtration, washed with cold diethyl ether, and then stirred for 2 hours in acetone (15 mL) at 50 °C. The filtrate produced 0.9 g (95%) of white solid. ^{1}H NMR (400 MHz, DMSO-d$_6$) δ 10.73 (s, 1H), 7.94 (s, 1H), 6.55 (s, 2H), 5.99 (d, J = 8.0 Hz, 1H), 5.80 (t, J = 8.0 Hz, 1H), 5.50 (dd, J = 4.0 Hz, 1H), 4.41-4.25 (m, 3H), 2.12-2.05 (m, 9H); HRMS (ESI) for $C_{22}H_{35}N_6O_8$ [M+TEA+H]$^+$: Calcd. 511.2507; Found. 511.2494.

2',3',5'-Tri-*O*-acetyl-8-trifluoromethylguanosine (8). 2',3',5'-Tri-*O*-acetyl-guanosine (0.9 g, 1.9 mmol) and Zinc trifluoromethanesulfinate (1.1 g, 6.1 mmol) were mixed with dimethylsulfoxide (23 mL) and dramatically stirred. After the solution is transparent (around 18 min), tert-butyl hydroperoxide (70% aqueous, 1.28 mL, 9.6 mmol) in 10 aliquots (128 μL each) in 22 min. intervals. The mixture gradually turn pale yellow following addition of tert-butyl hydroperoxide and continue reacted for 24 hours at room temperature. The mixture was transferred into 150 mL water and extracted with dichloromethane (3 × 40 mL). The separated organic layers were washed with water (3 × approx. 25 mL), brine (approx. 25 mL) and dried over with sodium sulfate. The obtained sample was filtered, washed with dichloromethane and the filtrate was dried in vacuum. The oily sample was further purified using MPLC using the combination of methanol and dichloromethane (5%, v/v) to give white

product (0.8 g, 58%). ^{1}H NMR (400 MHz, DMSO-d$_6$) δ 12.20 (s, 1H), 6.29 (s, 1H), 5.95 (t, J = 6.4 Hz, 2H), 4.55-4.52 (m, 1H), 4.46-4.38 (m, 2H), 2.15-2.04 (m, 9H); ^{19}F NMR (372 MHz, DMSO-d6) δ -61.1843 (s, 3F); HRMS (ESI) for C17H15O8N5F3Na [M+Na]$^+$: Calcd. 500.1065; Found. 500.0981.

8-Trifluoromethylguanosine (9). 2',3',5'-Tri-*O*-acetyl-8-trifluoromethylguanosine (0.8 g, 1.67 mmol) was mixed in 100 mL glass flask. Methylamine (33% in ethanol, 8.88 mL, 85.1 mmol) was added and given a reaction mixture, followed by that continue to reacted for 4.5 hours at room temperature. The solution was removed in vacuum and the sample was purified by MPLC in the mixture of methanol and dichloromethane (10%, v/v). The product was given as a yellow foam (2.0 g, 96%). ^{1}H NMR (400 MHz, DMSO-d$_6$) δ 11.05 (s, 1H), 6.72 (s, 2H), 5.64 (d, *J* = 6.0 Hz, 1H), 5.50 (d, *J* = 4.4 Hz, 1H), 5.14-4.92 (m, 3H), 4.17 (dd, *J* = 4.8 Hz, 1H), 3.91 (dd, *J* = 3.2 Hz, 1H), 3.71-3.52 (m, 2H); ^{19}F NMR (372 MHz, DMSO-d$_6$) δ -59.8351 (s, 3F); HRMS (ESI) for $C_{11}H_{11}O_5N_5F_3$ [M-H]$^-$: Calcd. 350.0688; Found. 350.0695.

***N*^{2}-dimethylformamidyl-8-trifluoromethylguanosine (10).** 8-Trifluoromethylguanosine (0.5 g, 1.42 mmol) and *N,N*-dimethylformamide dimethyl acetal (1.36 mL, 10.1 mmol) were dissolved in dehydrated dimethylformamide (10 mL). The mixture was stirred for 1 hour at room temperature and dried in vacuum. The remained sample was purified by MPLC in the mixture of methanol and dichloromethane (16%, v/v), the product was given as white solid (0.48 g, 85%). ^{1}H NMR (400 MHz, DMSO-d$_6$) δ 11.76 (s, 1H), 8.54 (s, 1H), 5.68 (d, *J* = 6.0 Hz, 1H), 5.42 (d, *J* = 6.4 Hz, 1H), 5.24 (d, *J* = 4.8 Hz, 1H), 5.05 (*J* = 6.0 Hz, 1H), 4.89 (*J* = 4.6 Hz, 1H), 4.27 (dd, *J* = 5.2 Hz, 1H), 3.94 (dd, *J* = 3.6 Hz, 1H), 3.72-3.56 (m, 2H), 3.18 (s, 3H), 3.08 (s, 3H); HRMS (ESI) for $C_{14}H_{16}O_5N_6F_3$ [M-H]$^-$: Calcd. 405.1231; Found. 405.1225.

***N*^{2}-dimethylformamidyl-8-trifluoromethyl-2'-*O*-(tert-butyldimethylsilyl)-3',5'-*O*-(di-tert-butylsilylene)guanosine (11).** *N*^{2}-dimethylformamidyl-8-trifluoromethylguanosine (100 mg, 0.24 mmol) was added into dehydrated dimethylformamide (6 mL), and di-tert-butylsilyl bis (trifuoromethanesulfonate) (115 mg, 0.27 mmol) was added dropwise. The reaction mixture incubated for 20 minutes at 0 °C. Imidazole (81.8 mg, 1.2 mmol) was added and continue to reacted for 15 minutes at 0 °C and additional 15 minutes at room temperature. Tert-butyldimethylsilyl chloride (172.8 mg, 1.2 mmol) was added, the mixture reacted for 4 hours at 65 °C. The mixture was evaporated in vacuum and the remained sample was separated by MPLC in the combination of dichloromethane and ethyl acetate (27%, v/v). The product was given as a white powder (130 mg, 88%). ^{1}H NMR (400 MHz, DMSO-d$_6$) δ 9.19 (s, 1H), 8.38 (s, 1H), 8.01 (s, 1H), 5.82 (d, *J* = 1.2 Hz, 1H), 5.09 (dd, *J* = 2.0 Hz, 1H), 4.52 (dd, *J* = 6.0 Hz, 1H), 4.42 (dd, *J* = 4.8 Hz, 1H), 4.06 (dt, *J* = 5.2 Hz, 1H), 3.93 (t, *J* = 6.8 Hz, 1H), 3.18-3.14 (m, 7H), 2.95-2.87 (m, 6H), 1.68 (s, 6H), 1.06-0.86 (m, 45H), 0.08-0.04 (m, 12H); HRMS (ESI) for $C_{28}H_{48}O_5N_6F_3Si_2$ [M+H]$^+$: Calcd. 661.3099; Found. 661.3160.

***N*^{2}-dimethylformamidyl-8-trifluoromethyl-5'-*O*-(4,4'-dimethoxytrityl)-2'-*O*-tert-butyldi methylsilylguanosine (12).** Compound **5** (165 mg, 0.25 mmol) was mixed in 1.12 mL dichloromethane, and 27.3 μL hydrofluoric acid-pyridine solution (70% hydrofluoric acid, 30% pyridine) in 0.17 mL pyridine was added and reacted at 0 °C for 2 hours. The mixture was

extracted via dichloromethane and aqueous solution, the organic layer evaporated in vacuum for giving crude compound 130 mg. In the absence of further purification, the collected 130 mg residue and 4,4'-dimethoxytrityl chloride (120.8 mg, 0.36 mmol) were dissolved in 5 mL dehydrated pyridine and stirred for additional 4 hours at room temperature. The solvent was removed in vacuum and purified via MPLC with the mixture of dichloromethane and ethyl acetate (27%, v/v). The product was provided as a white solid (127 mg, 62%).^{1}H NMR (400 MHz, DMSO-d_6) δ 9.05 (s, 1H), 8.61 (dt, *J* = 12 Hz, 1H), 8.20 (s, 1H), 7.41-7.14 (m, 8H), 6.77-6.73 (m, 4H), 5.81 (d, *J* = 4.0 Hz, 1H), 5.14 (dd, *J* = 4.0 Hz, 1H), 4.54 (dd, *J* = 6.4 Hz, 1H), 4.02 (dd, *J* = 12.4 Hz, 1H), 3.76 (s, 6H), 3.44-3.40 (m, 2H), 2.99 (s, 3H), 2.63 (s, 3H), 2.04 (s, 1H), 0.86 (s, 10H), 0.118 (s, 3H), 0.008 (s, 3H); HRMS (ESI) for $C_{41}H_{49}O_7N_6F_3SiNa$ $[M+Na]^+$: Calcd.845.3384; Found. 845.3332.

3'-*O*-[(2-Cyanoethoxy)(diisopropylamino)phosphino]-*N*^{2}-dimethylformamidyl-8-trifluoromethyl-5'-*O*-(4,4'-dimethoxytrityl)-2'-*O*-tert-butyldimethylsilylguanosine (13). Compound **6** (250 mg, 0.61 mmol) co-evaporated with 4 mL dehydrated acetonitrile in three times and dissolved with 5 mL dehydrated dichloromethane. Diisopropylethylamine (0.39 mL, 2.3 mmol) and 1-methylimidazole (0.05 mL, 0.56 mmol) were added. After 7 minutes, 2-cyanoethyl-*N,N*-diisopropylamidochlorophosphoramidite (0.38 mL, 1.82 mmol) was added, the reaction mixture was did for 1.2 hours at room temperature. The mixture was extracted with dichloromethane and aqueous solution, and concentrated *in vacuo*, the given compound was separated by MPLC in the combination of ethyl acetate and dichloromethane (28%, v/v). The compound was provided as a white powder (0.4 g, 72%). ^{1}H NMR (400 MHz, DMSO-d_6) δ 8.76 (s, 1H), 8.03 (s, 1H), 7.92 (s, 1H), 7.48-7.17 (m, 12H), 6.78-6.72 (m, 5H), 5.91-5.88 (m, 1H), 5.30 (m, 1H), 4.58-4.31 (m, 2H), 4.11 (s, 1H), 3.76-3.47 (m, 16H), 2.98-2.56 (m, 5H), 2.31 (s, 1H), 2.10-2.02 (m, 2H), 1.29-0.73 (m, 30H), 0.08-0.00 (m, 11H); ^{31}P NMR (161 MHz, DMSO-d_6) δ 150.86, 148.17; HRMS (ESI) for $C_{56}H_{81}O_8N_9F_3$ $[M+TEA+H]^+$: Calcd. 1124.5782; Found. 1124.5729.

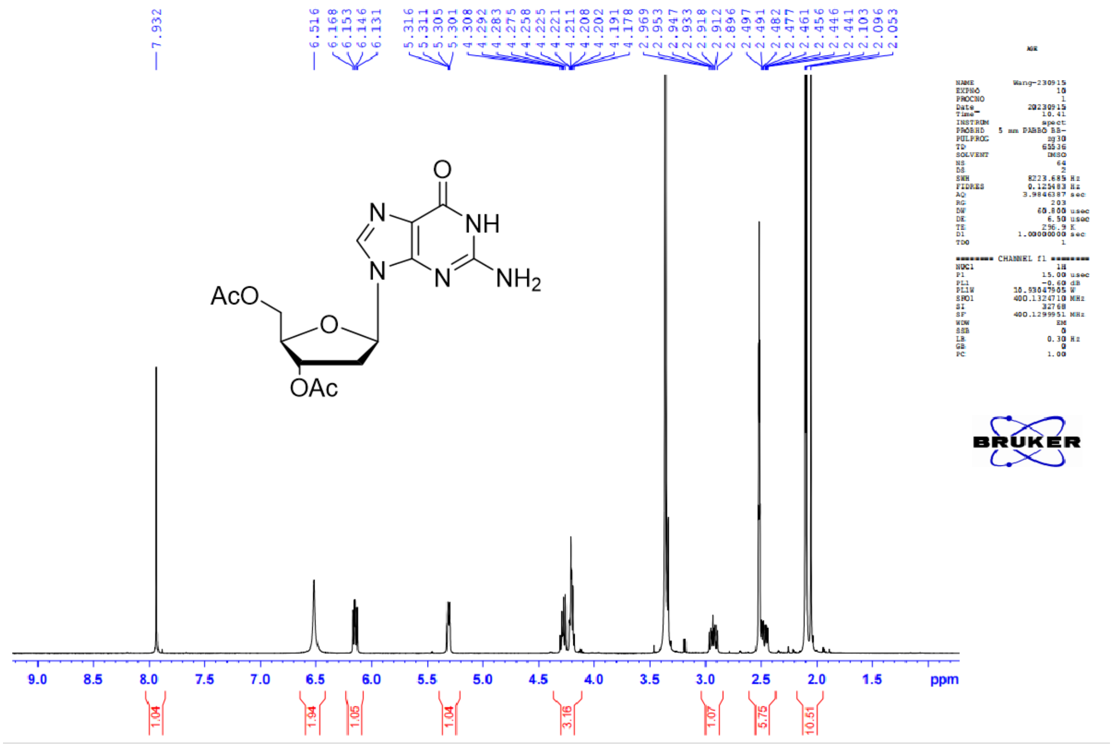

Data S1. ^{1}H NMR spectrum of 3',5'-di-*O*-acetyl-2'-deoxyguanosine (**1**).

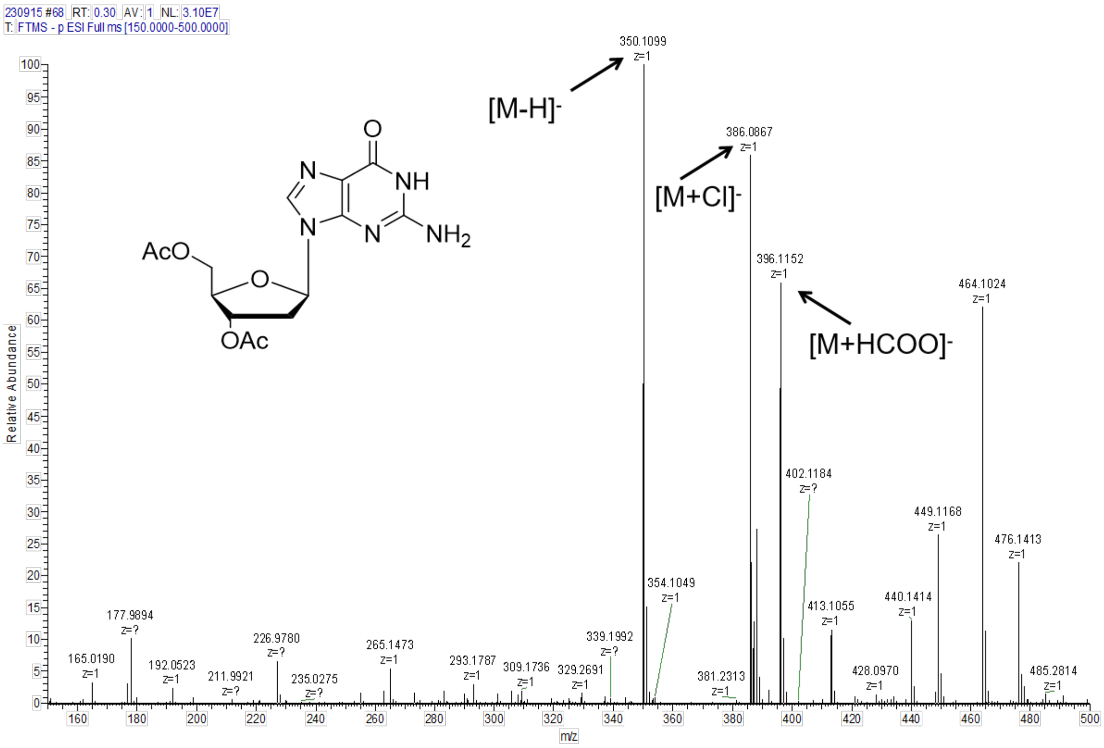

Data S2. HRMS spectrum of 3',5'-di-*O*-acetyl-2'-deoxyguanosine (**1**).

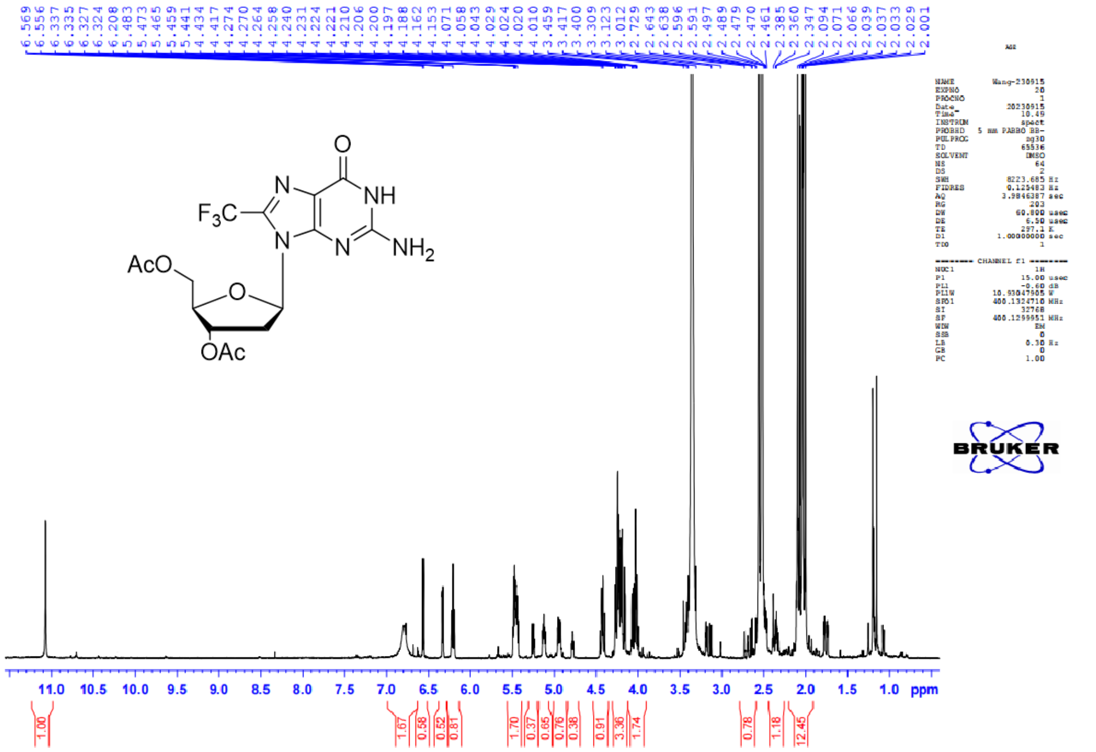

Data S3. ^{1}H NMR spectrum of 3',5'-di-*O*-acetyl-8-trifluoromethyl-2'-deoxyguanosine (**2**).

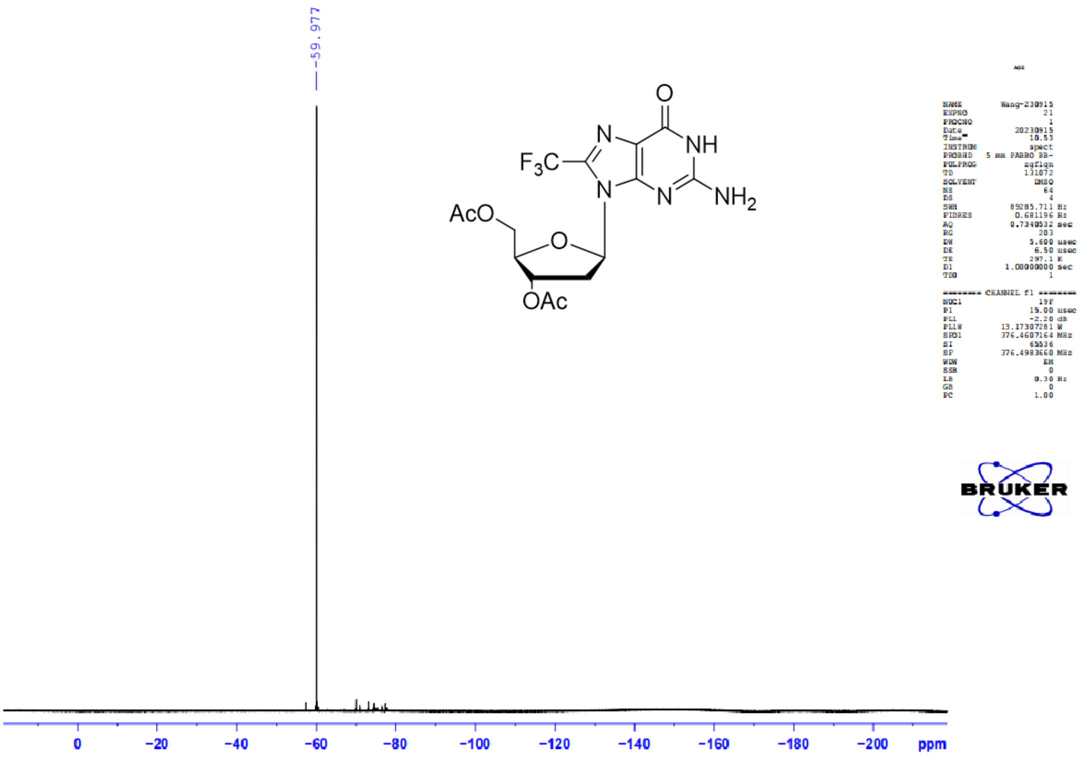

Data S4. ^{19}F NMR spectrum of 3',5'-di-*O*-acetyl-8-trifluoromethyl-2'-deoxyguanosine (**2**).

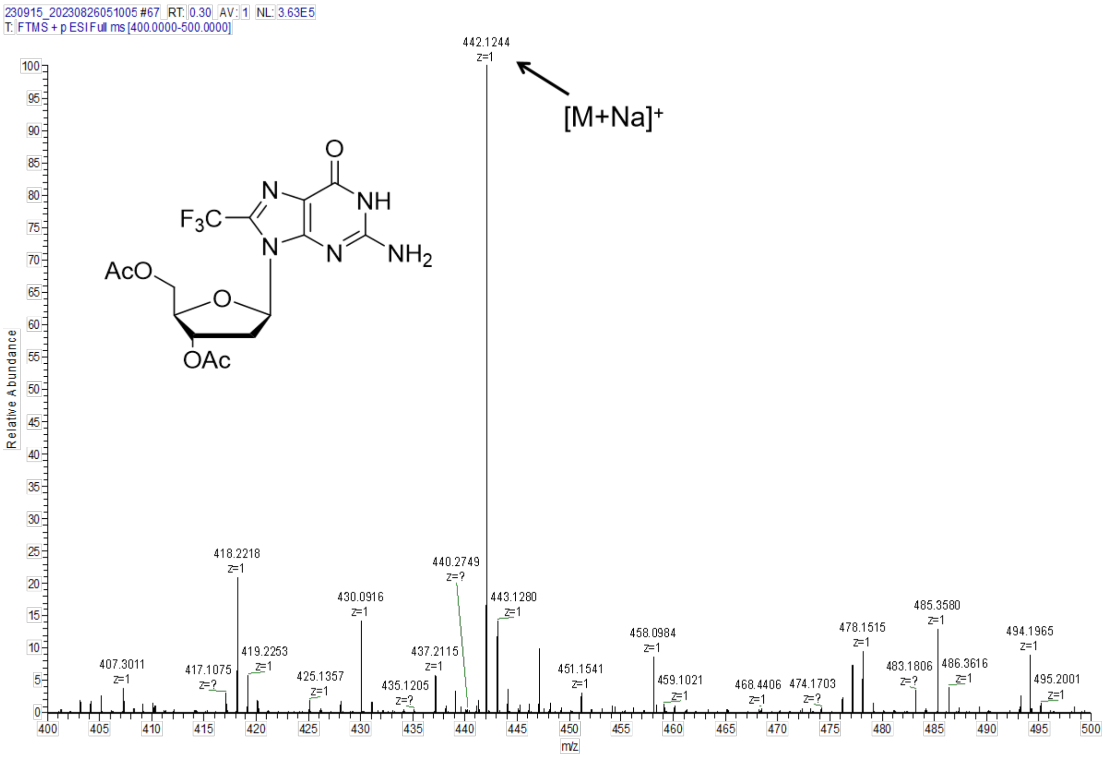

Data S5. HRMS spectrum of 3',5'-di-*O*-acetyl-8-trifluoromethyl-2'-deoxyguanosine (**2**).

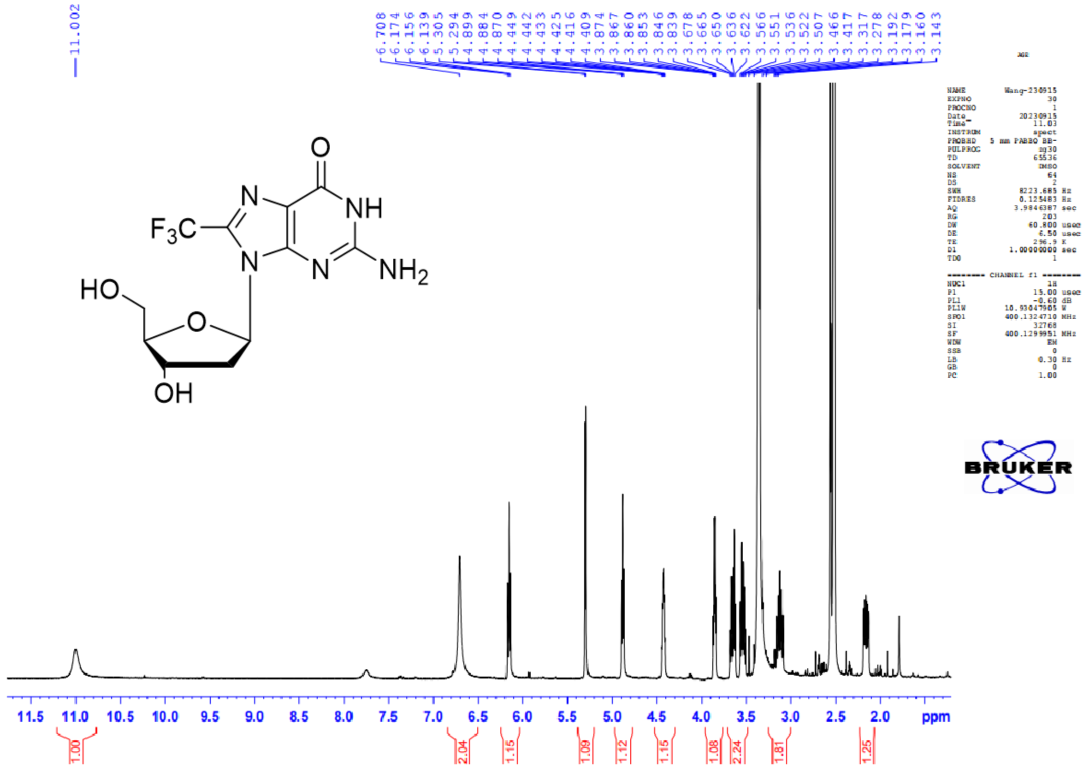

Data S6. ^{1}H NMR spectrum of 8-trifluoromethyl-2'-deoxyguanosine (**3**).

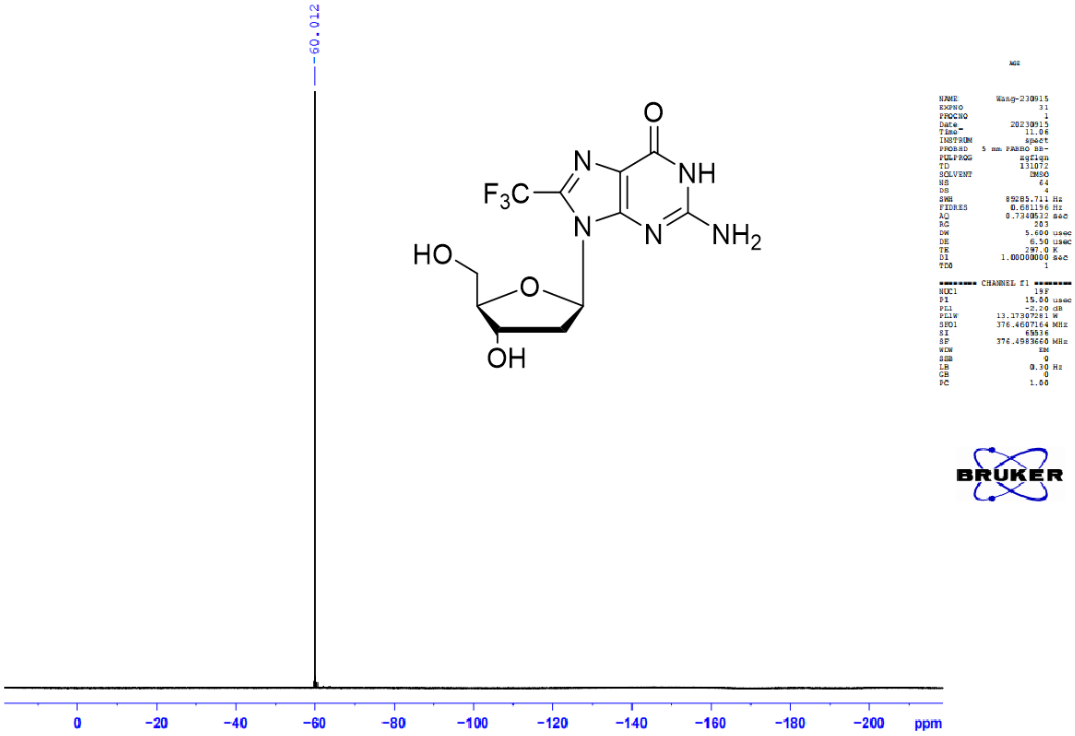

Data S7. ^{19}F NMR spectrum of 8-trifluoromethyl-2'-deoxyguanosine (**3**).

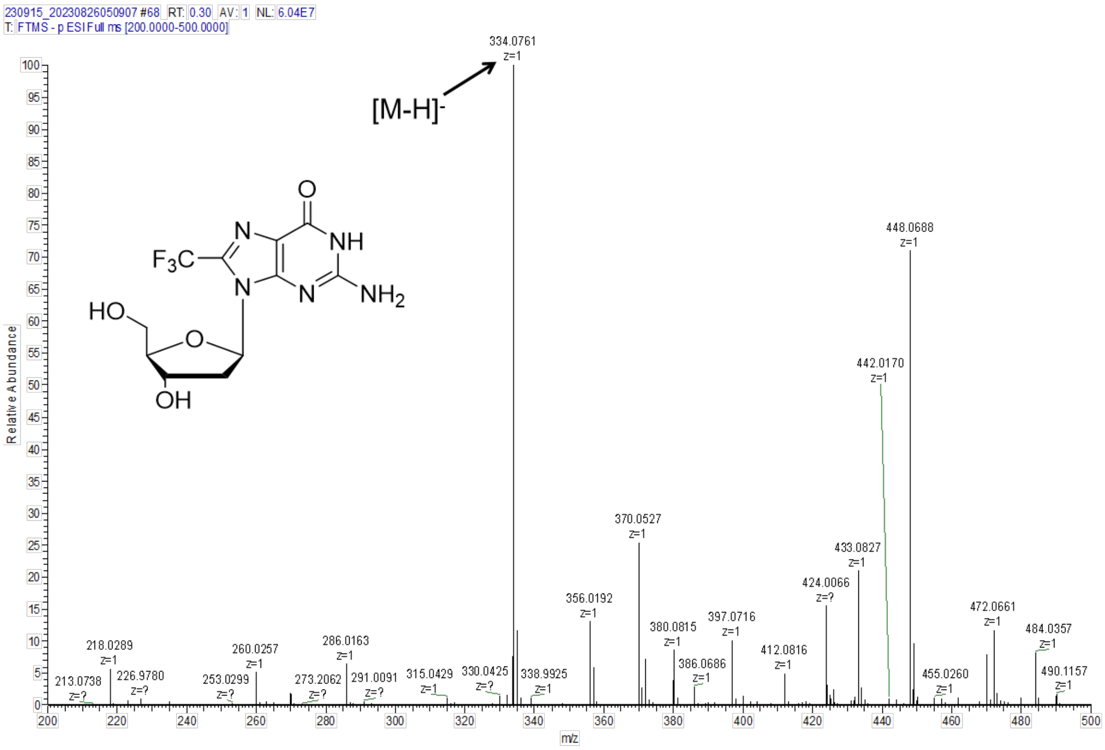

Data S8. HRMS spectrum of 8-trifluoromethyl-2'-deoxyguanosine (**3**).

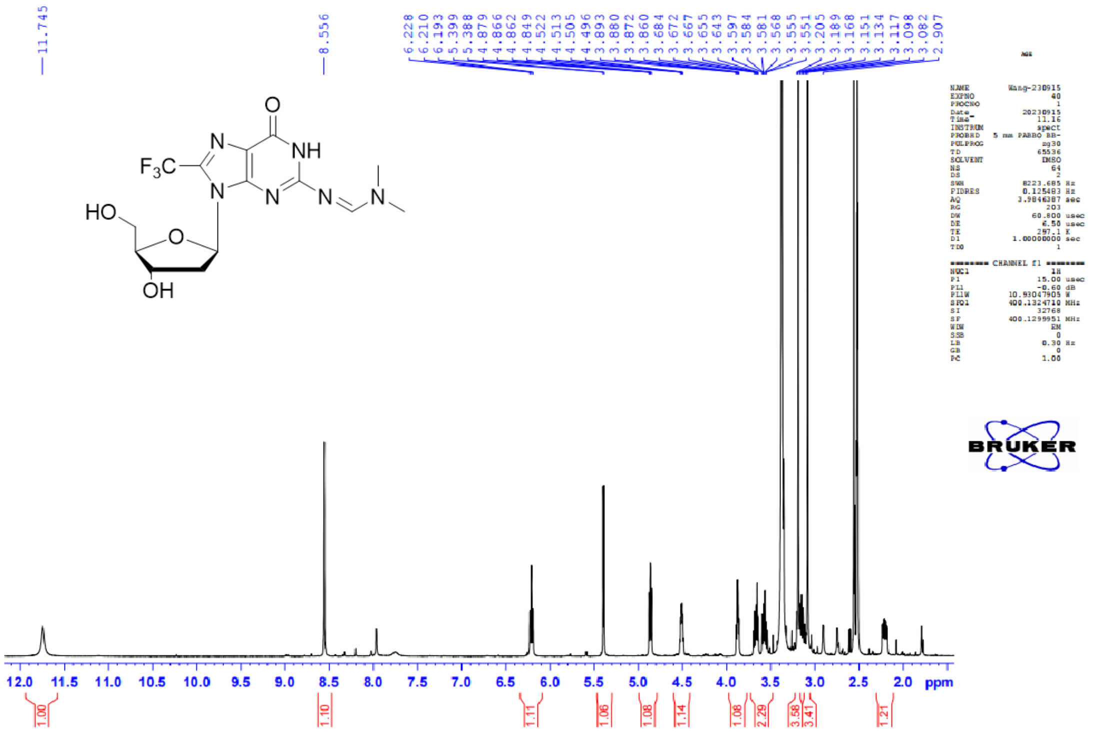

Data S9. ^{1}H NMR spectrum of N^2-dimethylformamidyl-8-trifluoromethyl-2'-deoxyguanosine (**4**).

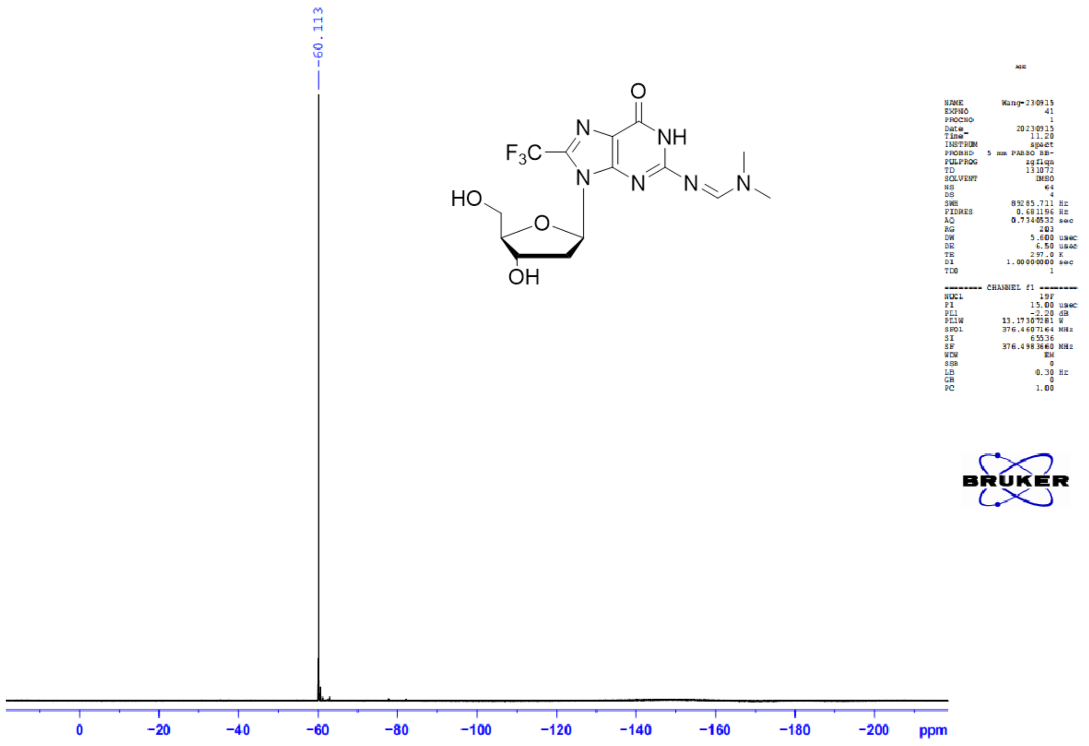

Data S10. ^{19}F NMR spectrum of N^2-dimethylformamidyl-8-trifluoromethyl-2'-deoxyguanosine (**4**).

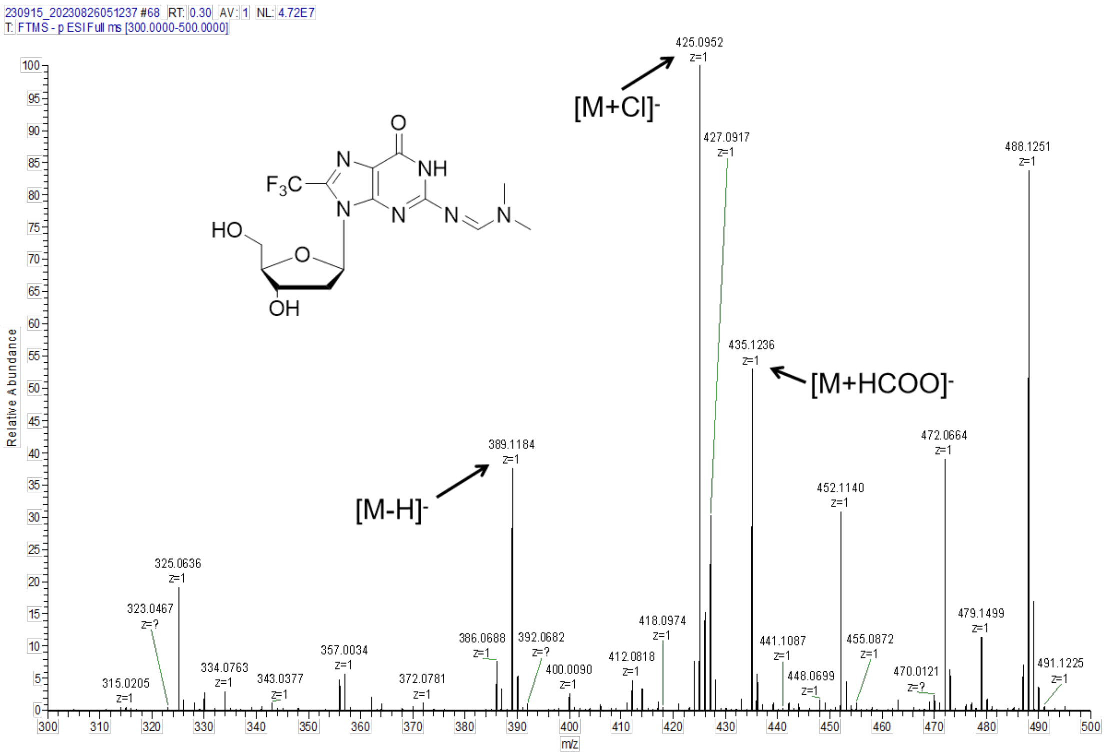

Data S11. HRMS spectrum of N^2-dimethylformamidyl-8-trifluoromethyl-2'-deoxyguanosine (**4**).

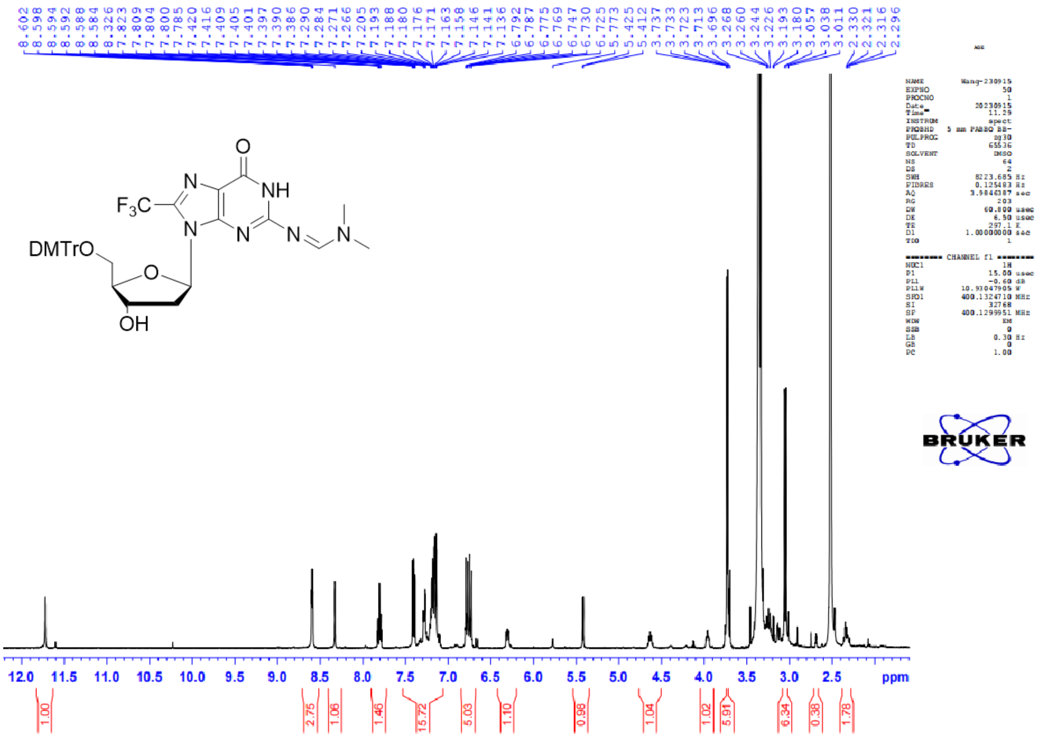

Data S12. ^{1}H NMR spectrum of N^2-dimethylformamidyl-8-trifluoromethyl-5'-O-(4,4'-dimethoxytrityl)-2'-deoxyguanosine (**5**).

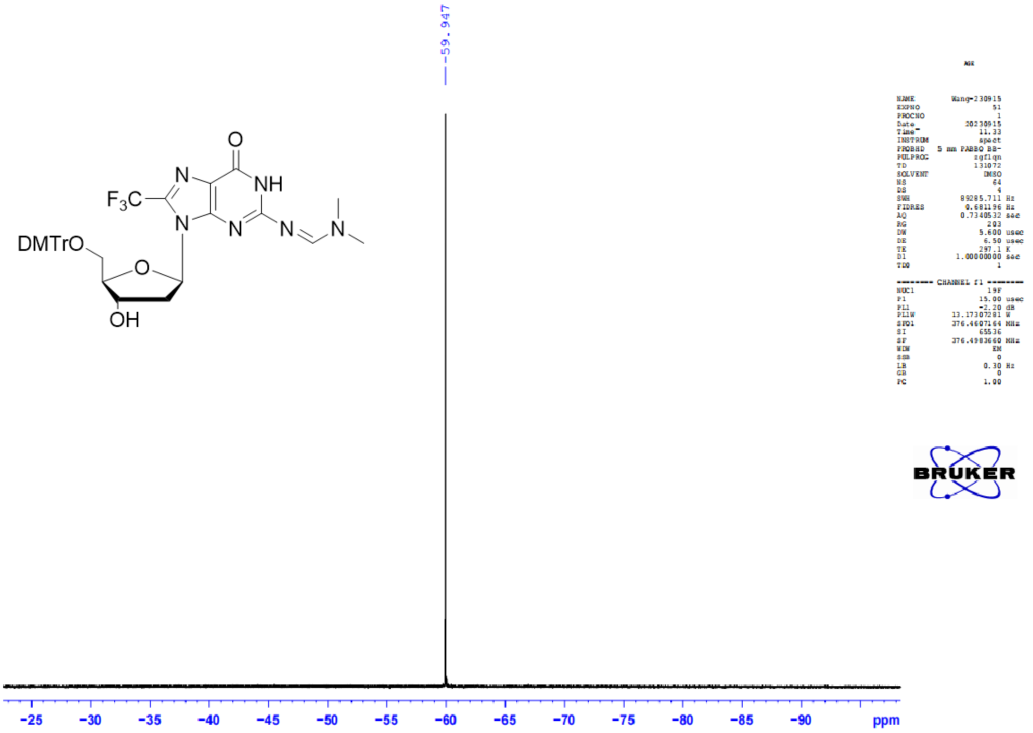

Data S13. ^{19}F NMR spectrum of *N*[2]-dimethylformamidyl-8-trifluoromethyl-5'-O-(4,4'-dimethoxytrityl)-2'-deoxyguanosine (**5**).

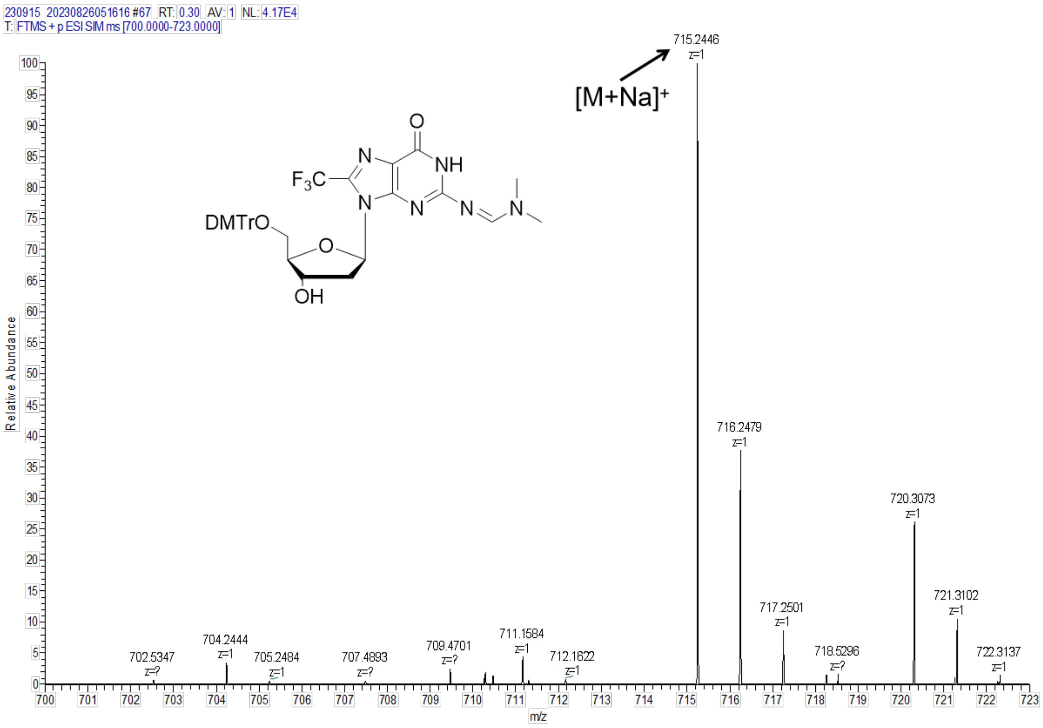

Data S14. HRMS spectrum of *N*[2]-dimethylformamidyl-8-trifluoromethyl-5'-O-(4,4'-dimethoxytrityl)-2'-deoxyguanosine (**5**).

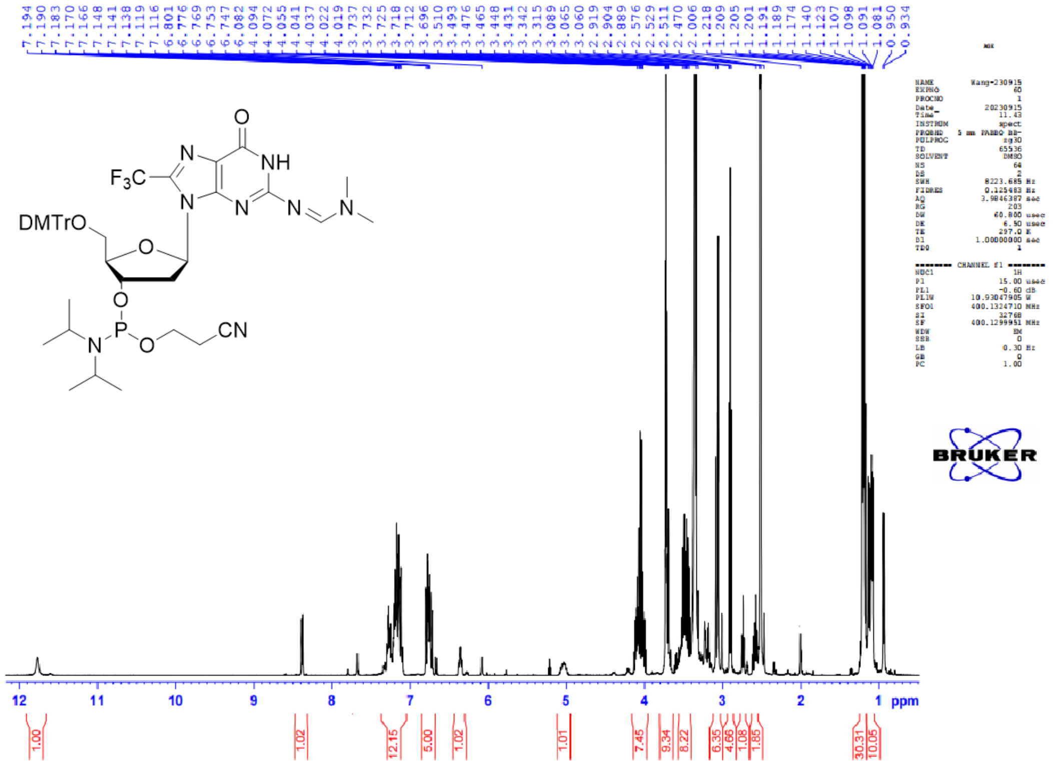

Data S15. ^{1}H NMR spectrum of 3'-*O*-[(2-Cyanoethoxy)(diisopropylamino)phosphino]-*N*^{2}-dimethylformamidyl-8-trifluoromethyl-5'-*O*-(4,4'-dimethoxytrityl)-2'-deoxyguanosine (**6**).

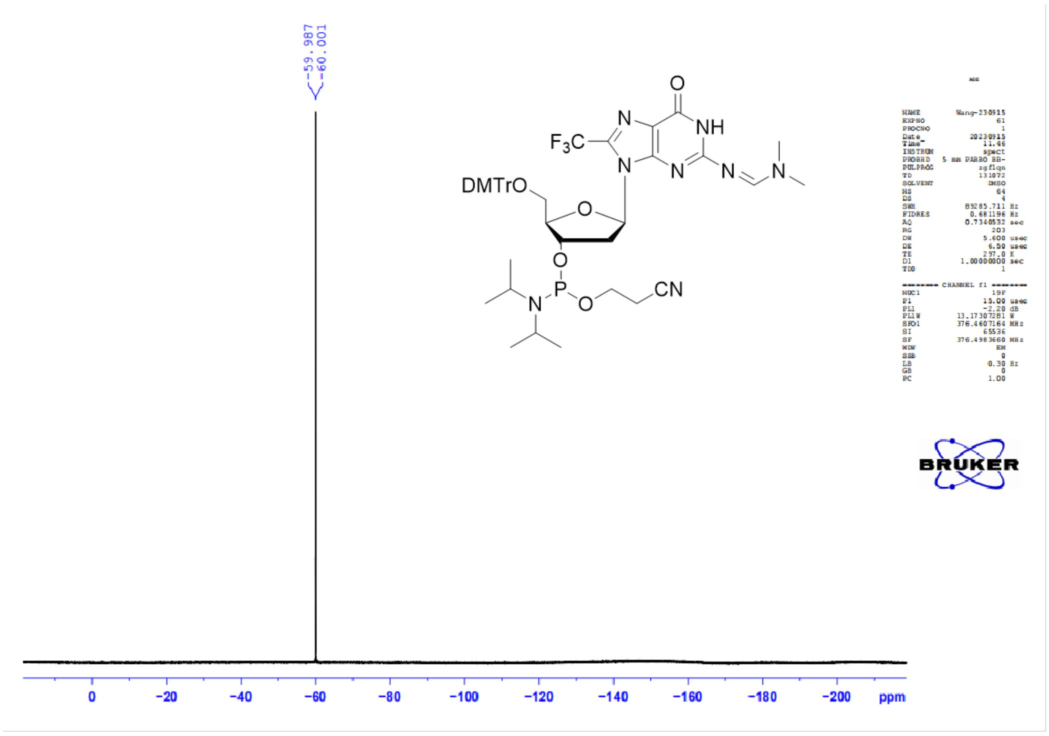

Data S16. ^{19}F NMR spectrum of 3'-*O*-[(2-Cyanoethoxy)(diisopropylamino)phosphino]-*N*^{2}-dimethylformamidyl-8-trifluoromethyl-5'-*O*-(4,4'-dimethoxytrityl)-2'-deoxyguanosine (**6**).

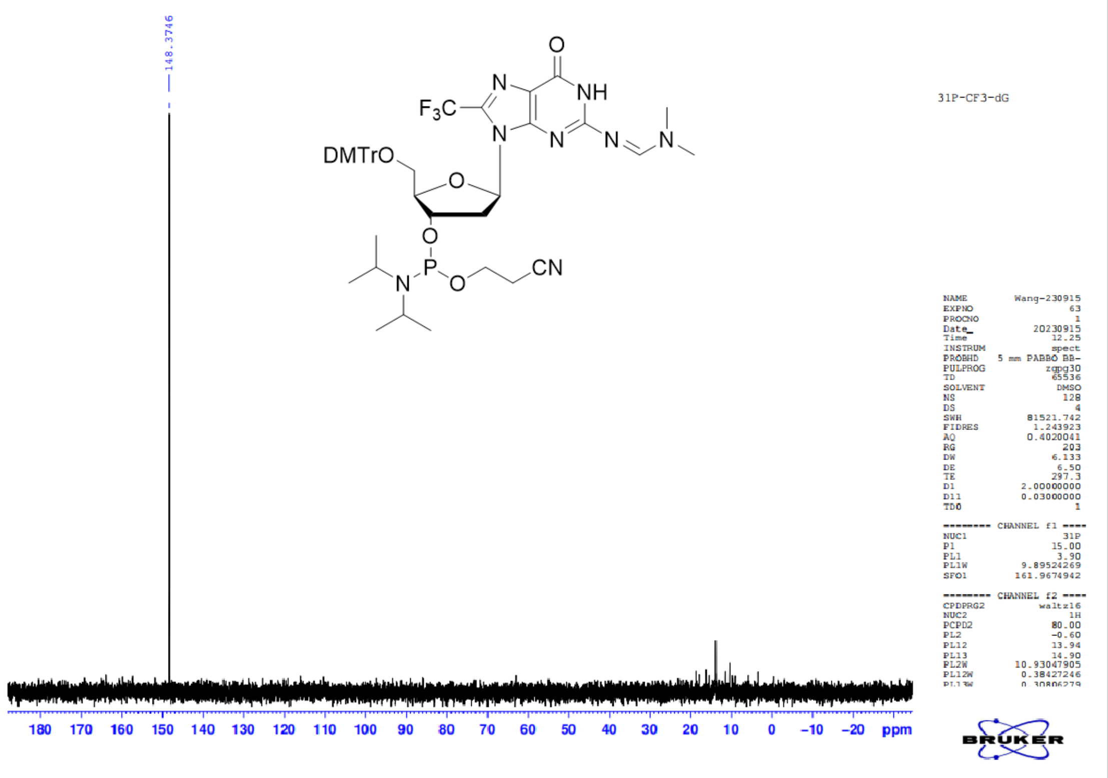

Data S17. ^{31}P NMR spectrum of 3'-*O*-[(2-Cyanoethoxy)(diisopropylamino)phosphino]-*N*[2]-dimethylformamidyl-8-trifluoromethyl-5'-*O*-(4,4'-dimethoxytrityl)-2'-deoxyguanosine (**6**).

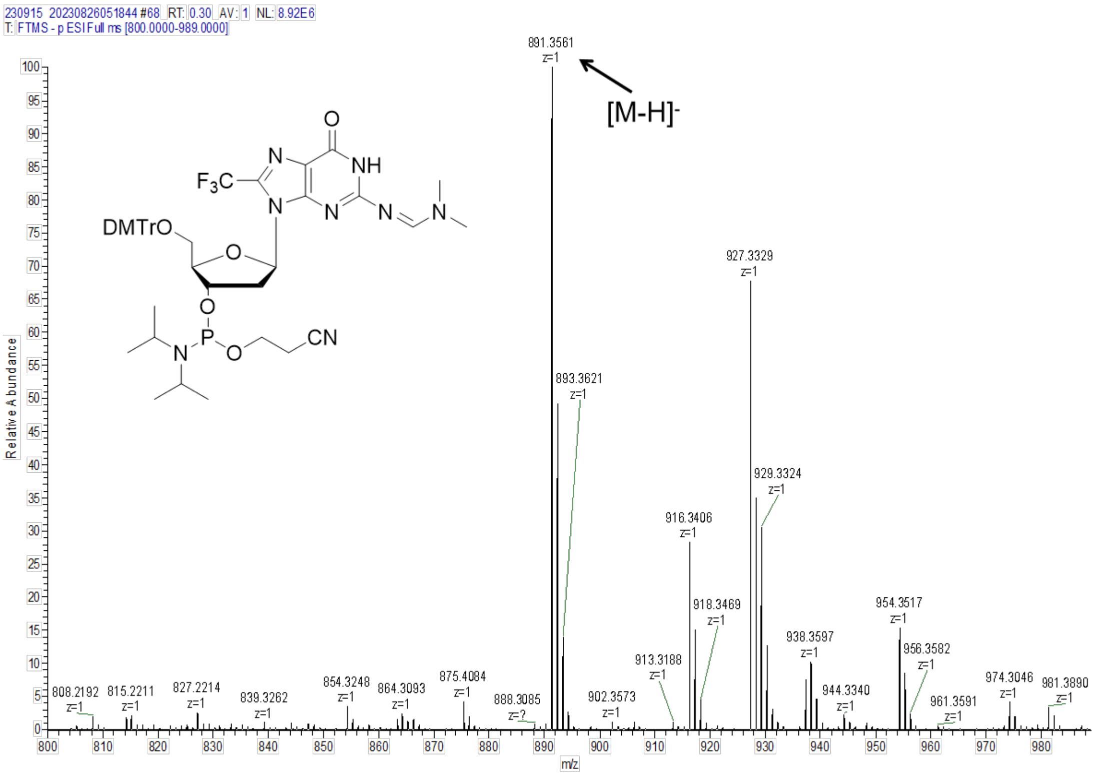

Data S18. HRMS spectrum of 3'-*O*-[(2-Cyanoethoxy)(diisopropylamino)phosphino]-*N*[2]-dimethylformamidyl-8-trifluoromethyl-5'-*O*-(4,4'-dimethoxytrityl)-2'-deoxyguanosine (**7**).

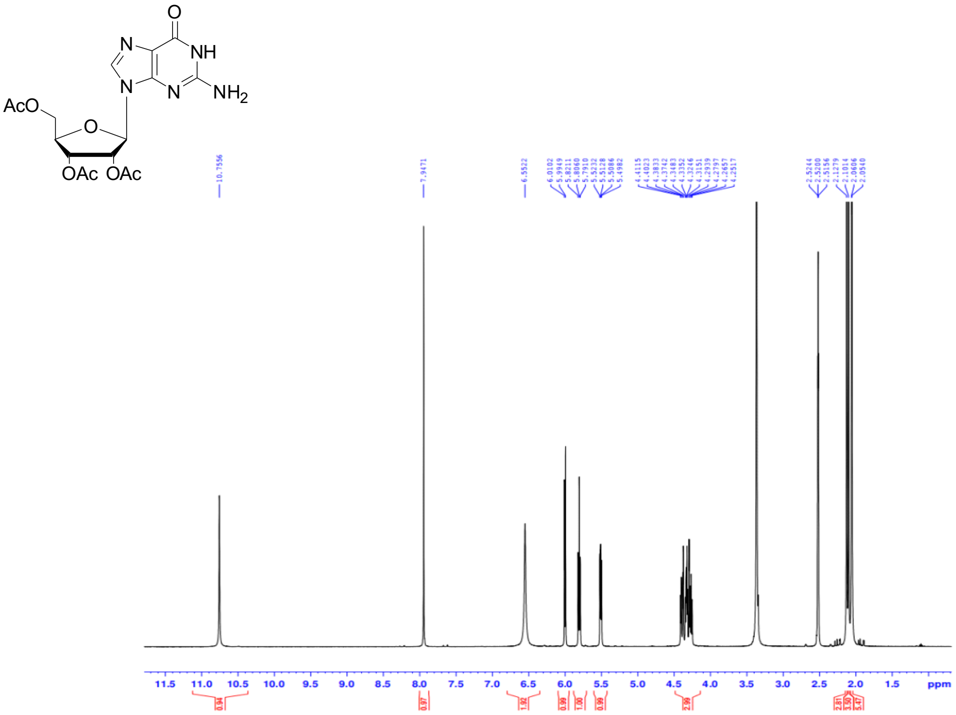

Data S19. ^{1}H NMR spectrum of 2',3',5'-tri-*O*-acetylguanosine (**7**).

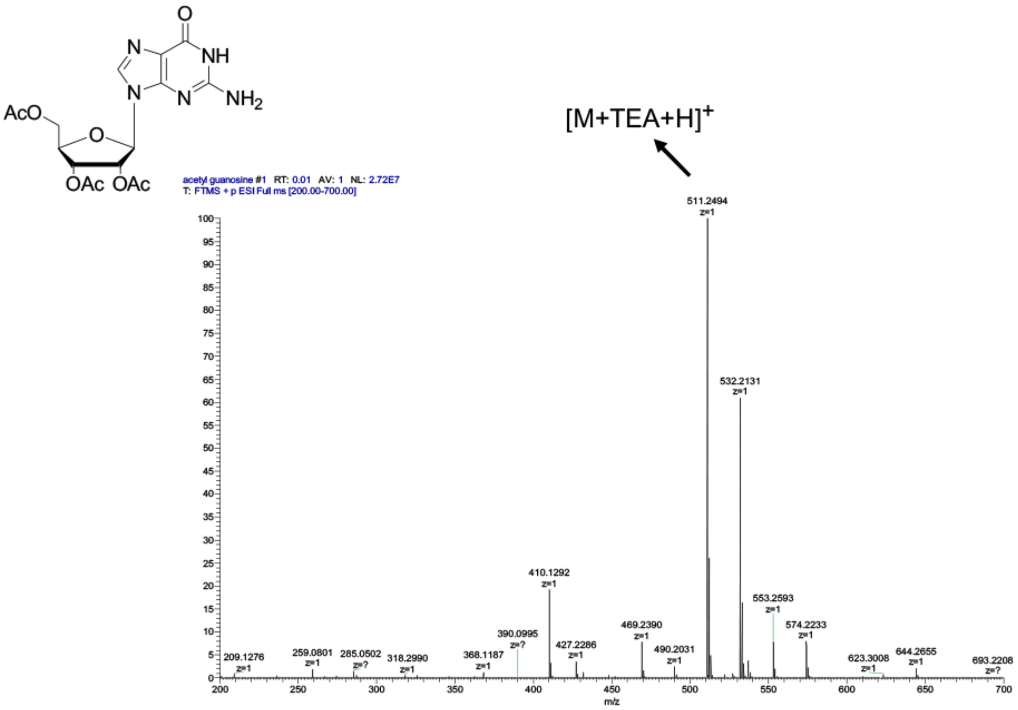

Data S20. HRMS spectrum of 2',3',5'-tri-*O*-acetylguanosine (**7**).

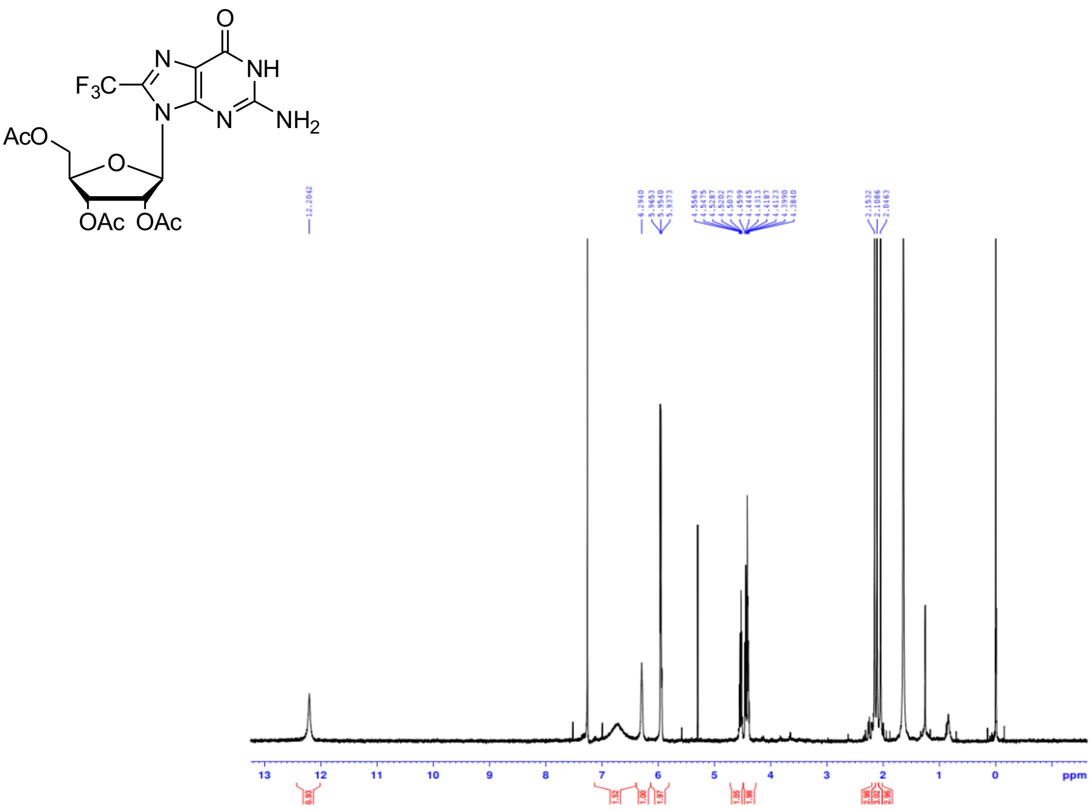

Data S21. ^{1}H NMR spectrum of 2',3',5'-tri-*O*-acetyl-8-trifluoromethylguanosine (**8**).

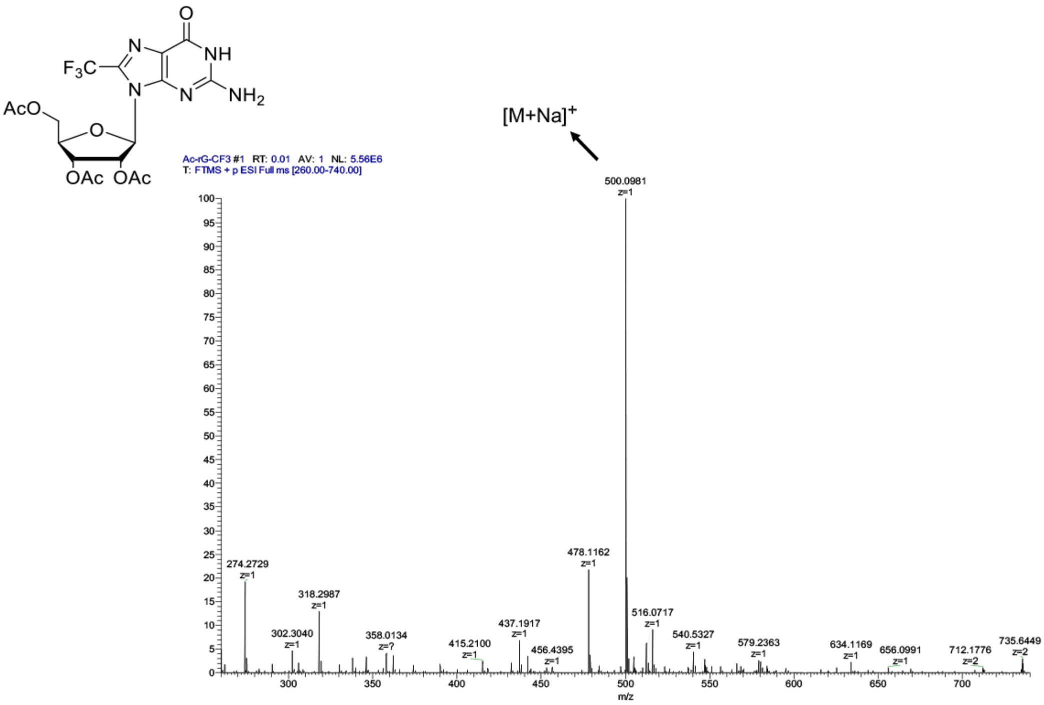

Data S22. HRMS spectrum of 2',3',5'-tri-*O*-acetyl-8-trifluoromethylguanosine (**8**).

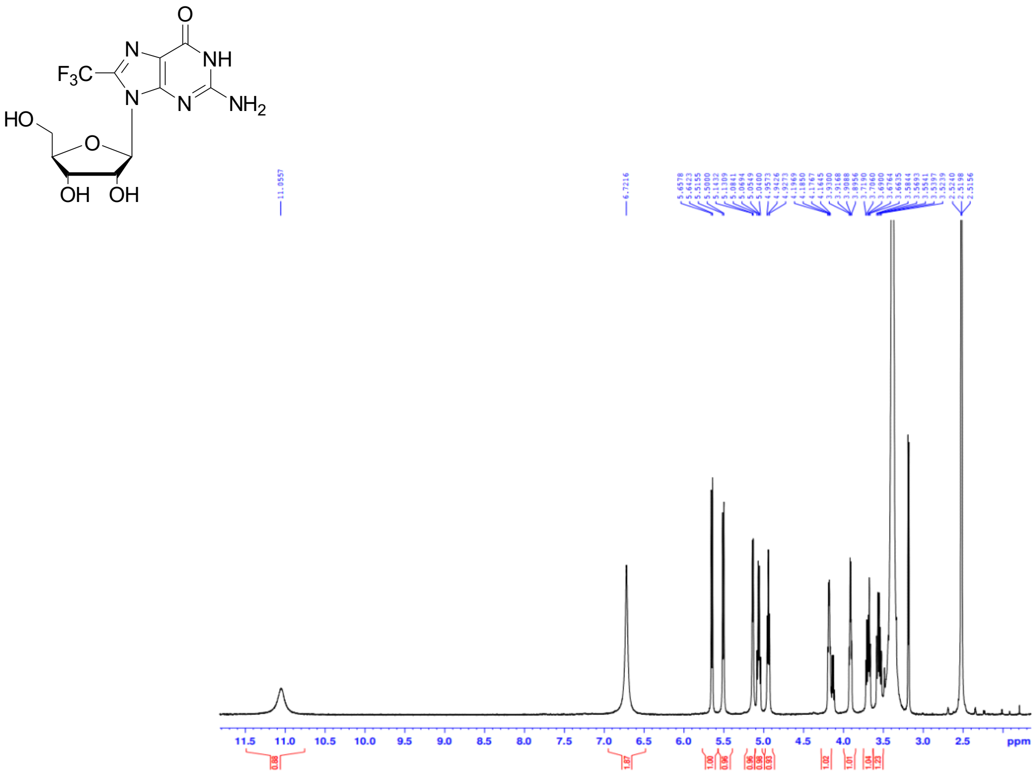

Data S23. ^{1}H NMR spectrum of 8-trifluoromethylguanosine (**9**).

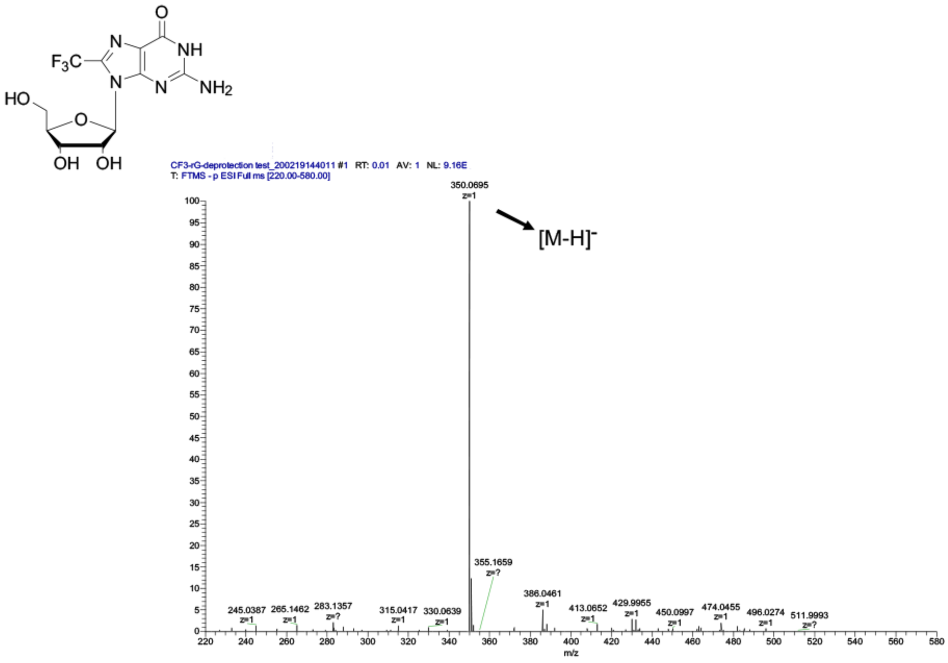

Data S24. HRMS spectrum of 8-trifluoromethylguanosine (**9**).

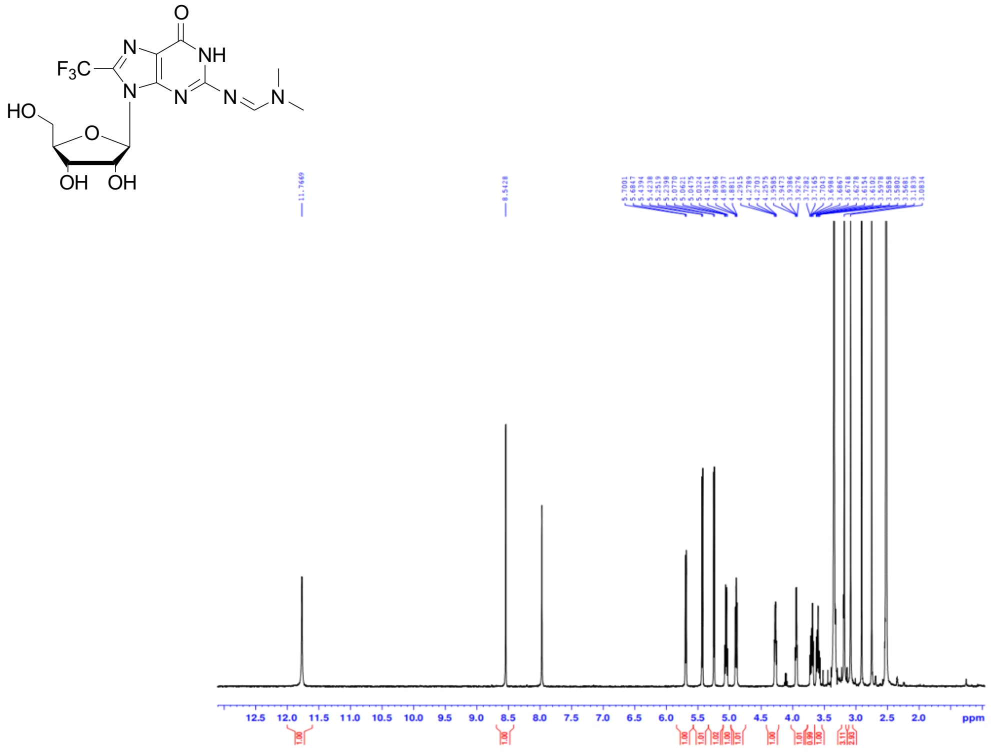

Data S25. ^{1}H NMR spectrum of *N2*-dimethylformamidyl-8-trifluoromethylguanosine (**10**).

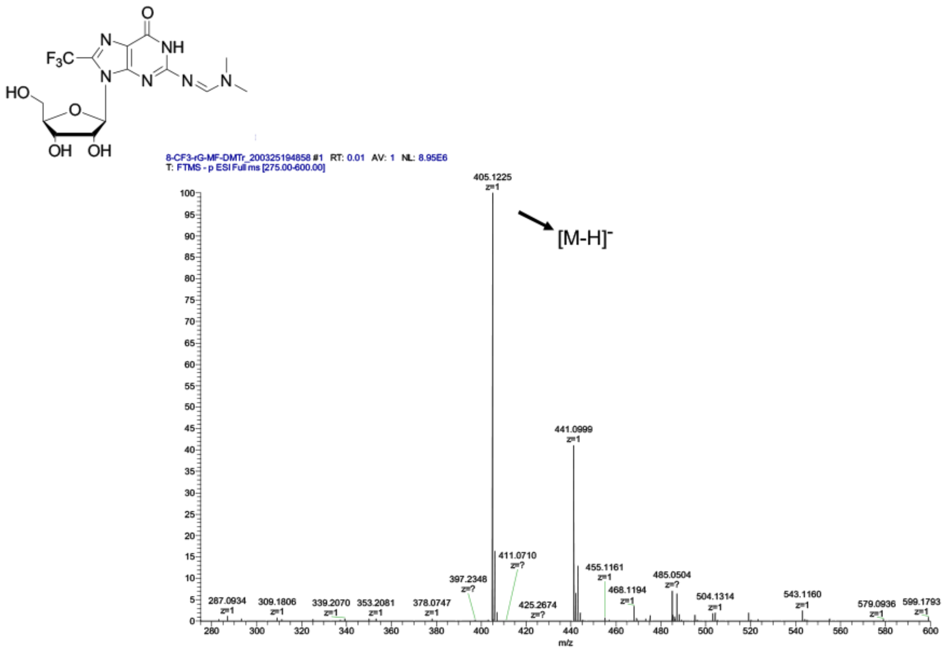

Data S26. HRMS spectrum of *N2*-dimethylformamidyl-8-trifluoromethylguanosine (**10**).

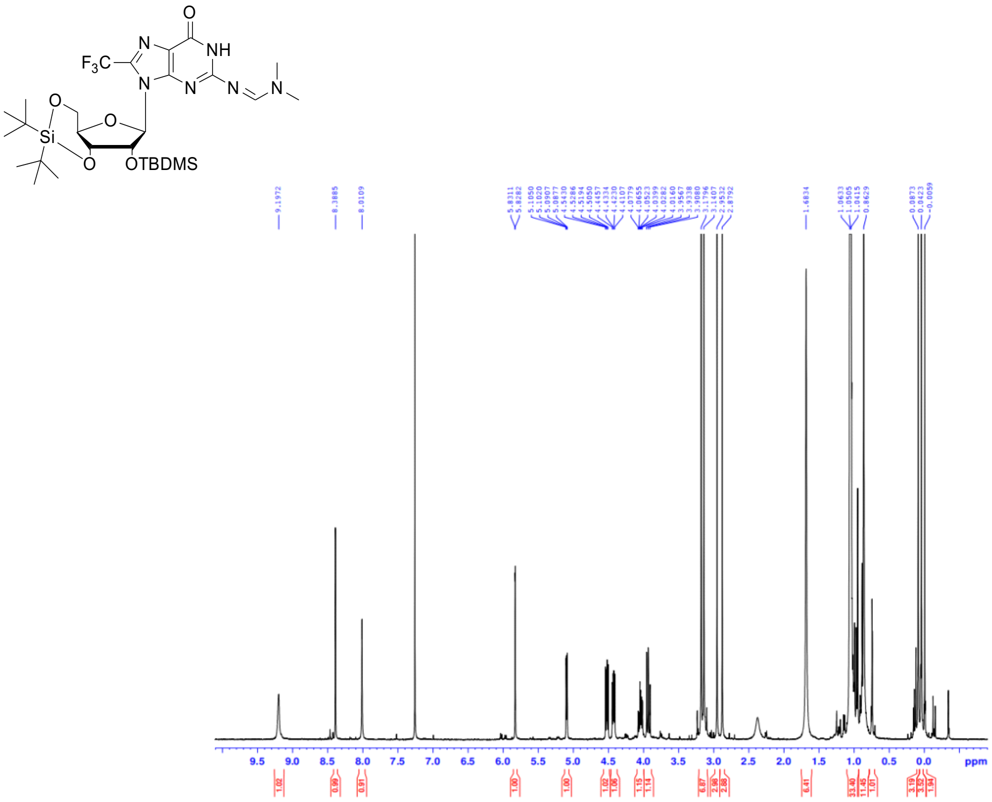

Data S27. ^{1}H NMR spectrum of *N2*-dimethylformamidyl-8-trifluoromethyl-2'-*O*-(tert-butyldimethylsilyl)-3',5'-*O*-(di-tert-butylsilylene)guanosine (**11**).

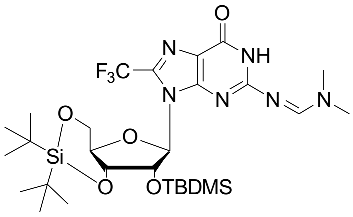

Data S28. HRMS spectrum of *N2*-dimethylformamidyl-8-trifluoromethyl-2'-*O*-(tert-butyldimethylsilyl)-3',5'-*O*-(di-tert-butylsilylene)guanosine (**11**).

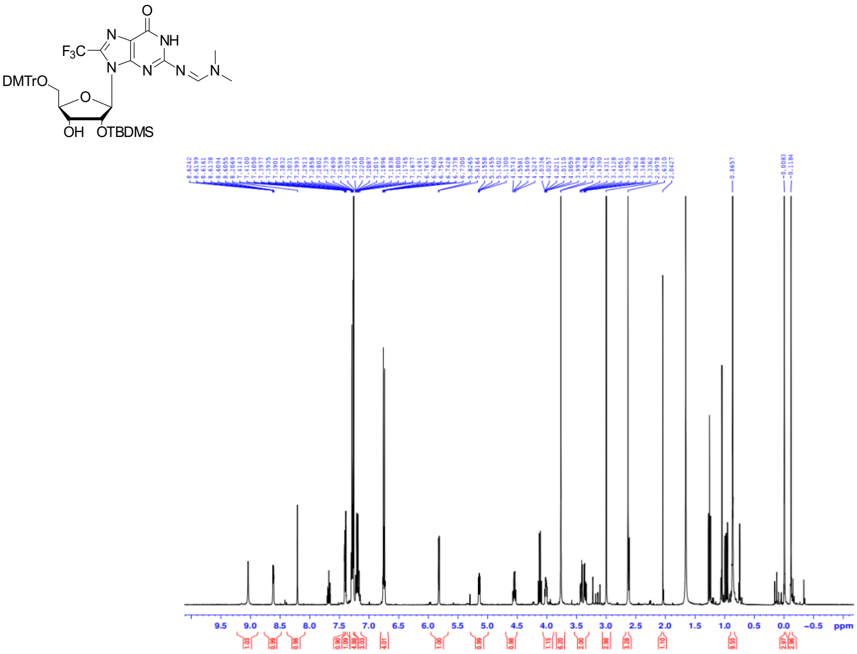

Data S29. ^{1}H NMR spectrum of *N2*-dimethylformamidyl-8-trifluoromethyl-5'-*O*-(4,4'-dimethoxytrityl)-2'-*O*-tert-butyl dimethylsilylguanosine (**12**).

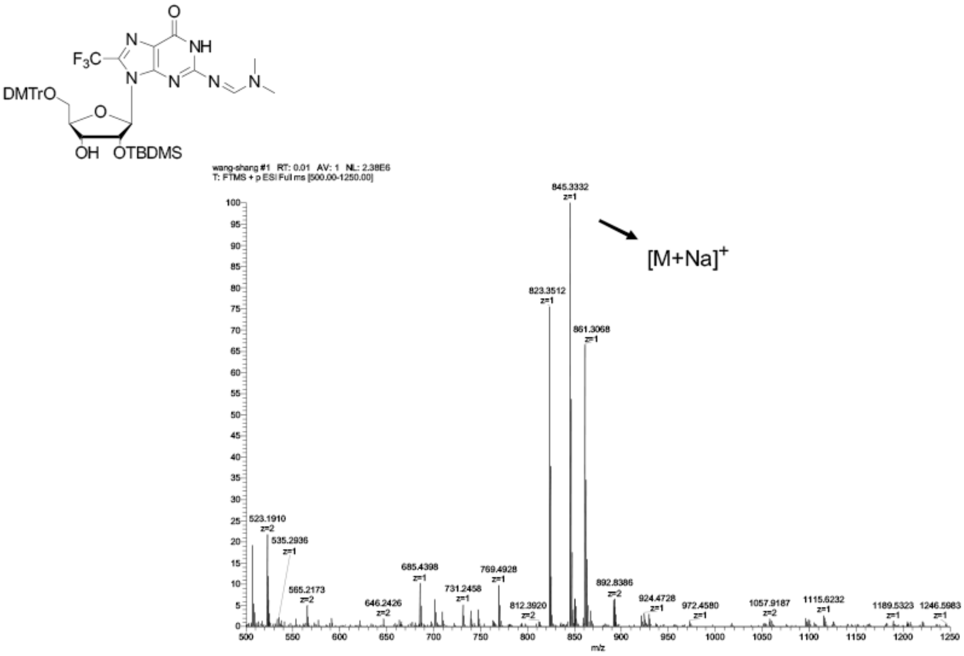

Data S30. HRMS spectrum of *N2*-dimethylformamidyl-8-trifluoromethyl-5'-*O*-(4,4'-dimethoxytrityl)-2'-*O*-tert-butyl dimethylsilylguanosine (**12**).

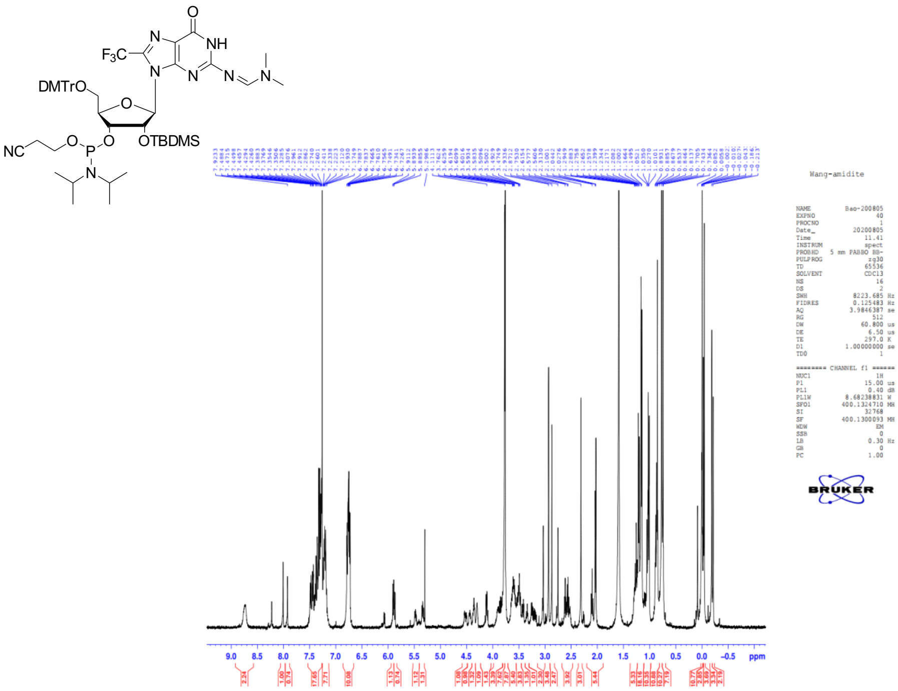

Data S31. ^{1}H NMR spectrum of 3'-*O*-[(2-Cyanoethoxy)(diisopropylamino)phosphino]-*N2*-dimethylformamidyl-8-trifluoromethyl-5'-*O*-(4,4'-dimethoxytrityl)-2'-*O*-tert-butyldimethylsilylguanosine (**13**).

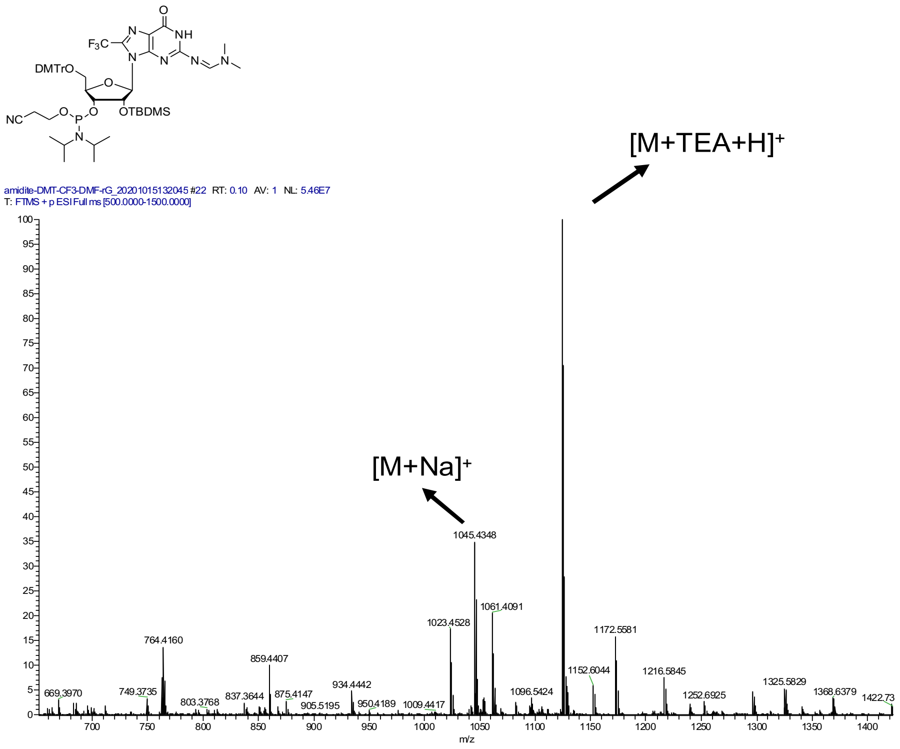

Data S32. HRMS spectrum of 3'-*O*-[(2-Cyanoethoxy)(diisopropylamino)phosphino]-*N2*-dimethylformamidyl-8-trifluoromethyl-5'-*O*-(4,4'-dimethoxytrityl)-2'-*O*-tert-butyldimethylsilylguanosine (**13**).

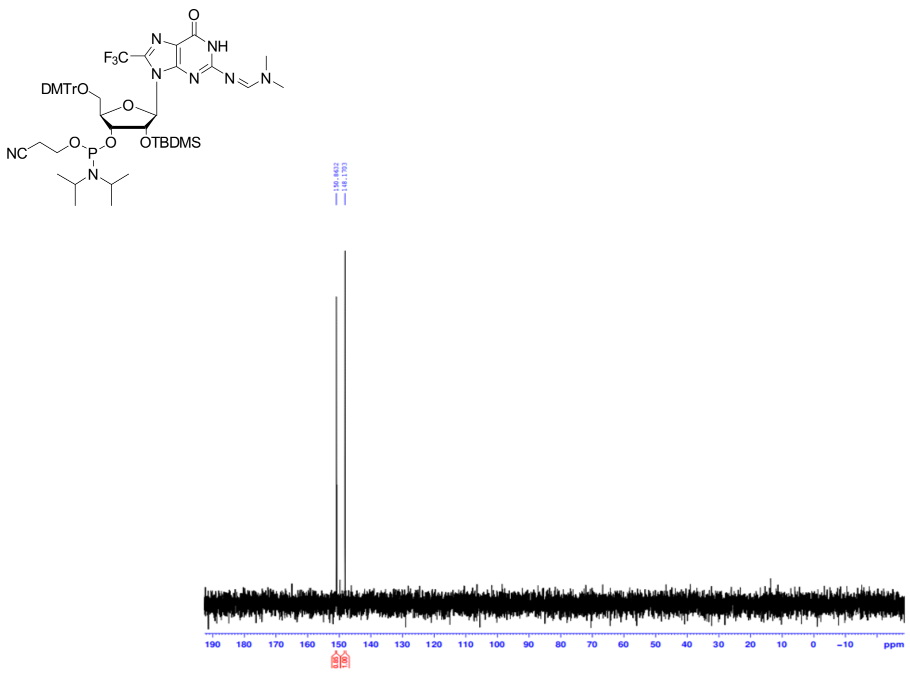

Data S33. ^{31}P NMR spectrum of 3'-*O*-[(2-Cyanoethoxy)(diisopropylamino)phosphino]-*N2*-dimethylformamidyl-8-trifluoromethyl-5'-*O*-(4,4'-dimethoxytrityl)-2'-*O*-tert-butyldimethylsilylguanosine (1**3**).

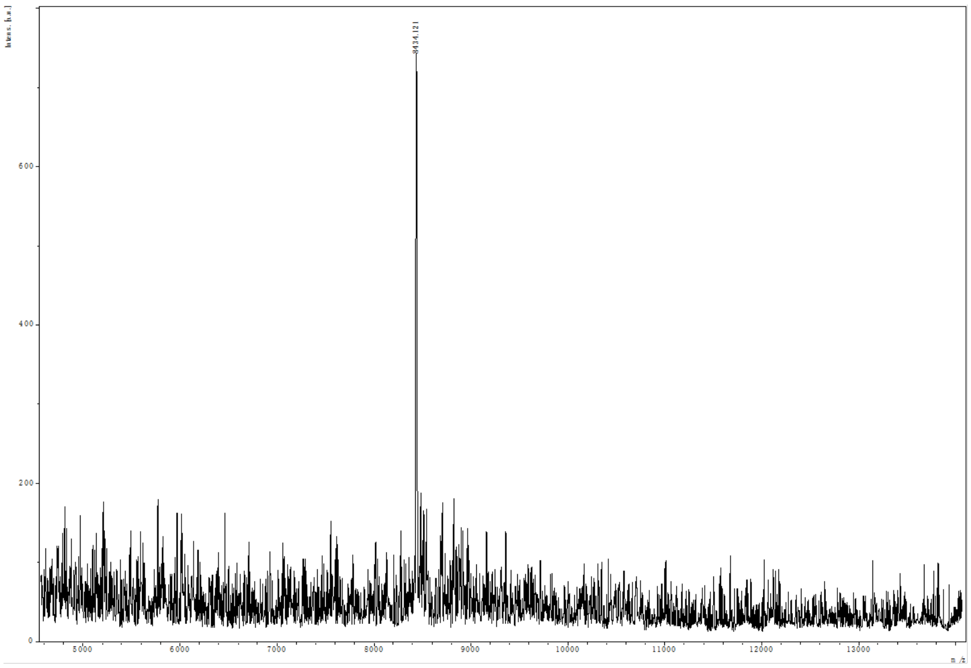

Data S34. MALDI-TOF MS of 5’-d(CG^{m}CG^{m}CG^{m}CG^{m}CG^{m}CG^{m}CG^{m}CG^{m}CG^{m}CG^{m}CGAGACA)-3’. Calcd. [M-H]$^{-}$: 8435.698; Found 8434.141.

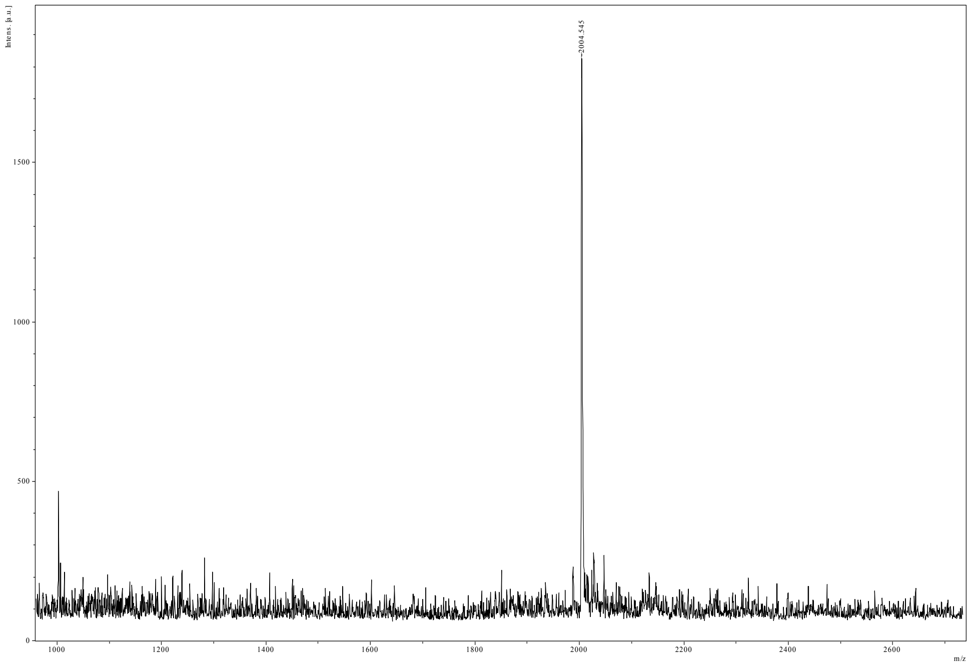

Data S35. MALDI-TOF MS of Cy3-5’-r(C^{F}GC^{F}G)-3’. Calcd. [M-H]$^{-}$: 2004.863; Found 2004.545.

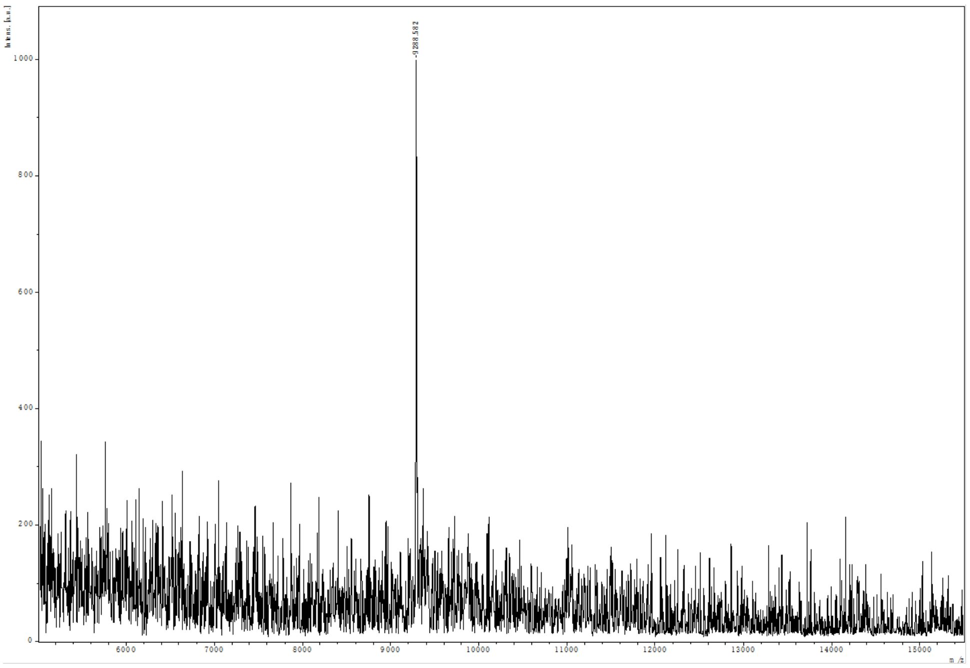

Data S36. MALDI-TOF MS of Cy3-5'-r(UGUCUCGCGCGCGCGCGCGCGCGCGCG)-3'. Calcd. $[M\text{-}H]^-$: 9289.145; Found 9288.582.

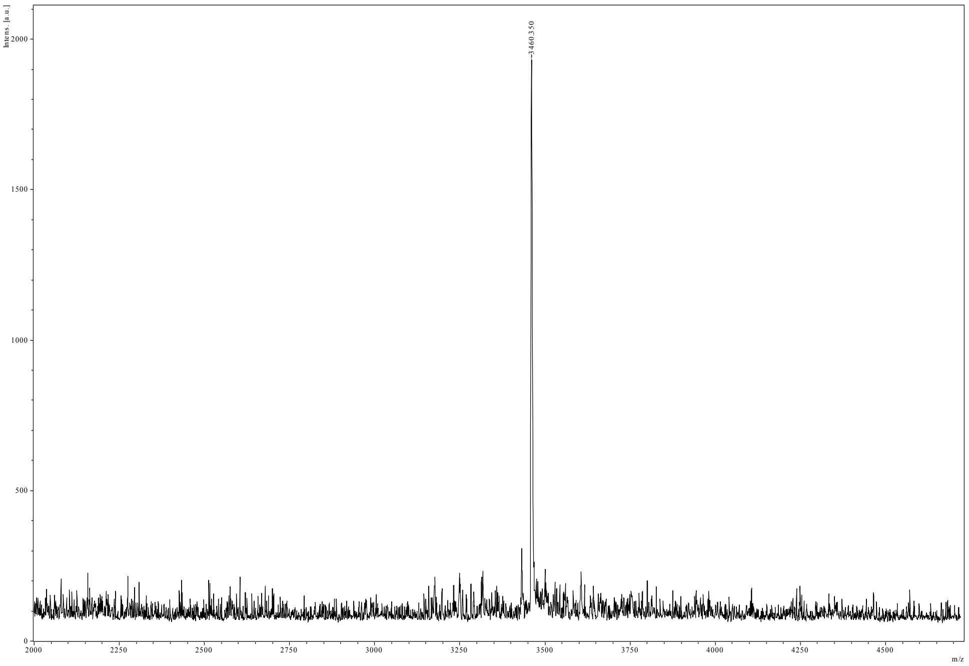

Data S37. MALDI-TOF MS of 5'-d($C^FGA^FGCC^FG^FGCT$)-3'. Calcd. $[M\text{-}H]^-$: 3461.248; Found 3460.350.

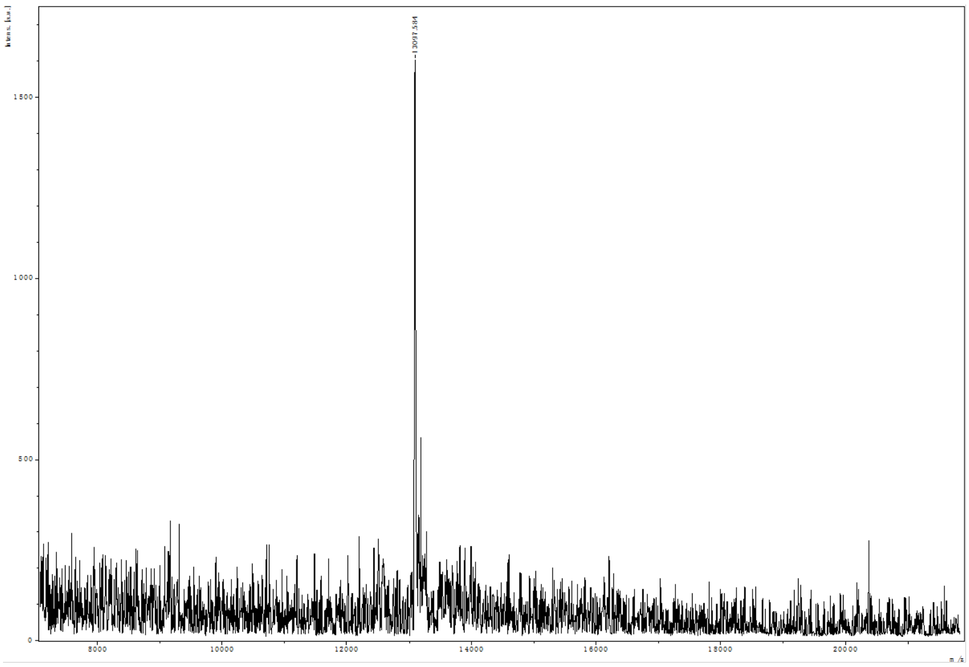

Data S38. MALDI-TOF MS of 5’-d(GATCTGAATCGAATTCG^{m}CG^{m}CG^{m}CG^{m}CG^{m}CG^{m}CG^{m}CG^{m}CG^{m}CG^{m}CGAGACA)-3’. Calcd. [M-H]$^{-}$: 13099.639; Found 13097.584.

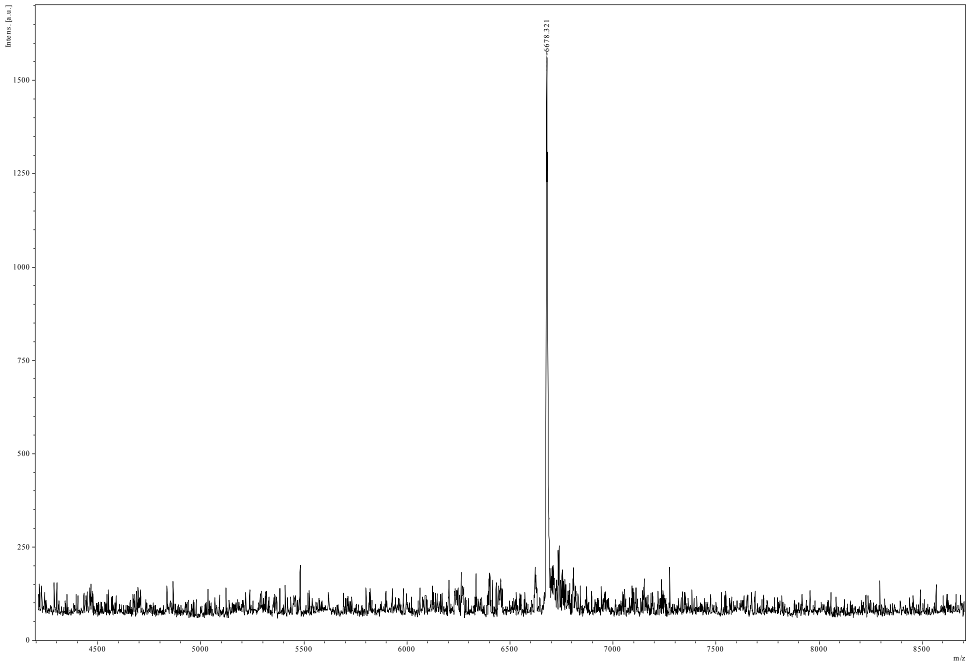

Data S39. MALDI-TOF MS of 5’-d(CCAGACATATC^{F}GA^{F}GCC^{F}G^{F}GCT)-3’. Calcd. [M-H]$^{-}$: 6678.908; Found 6678.321.

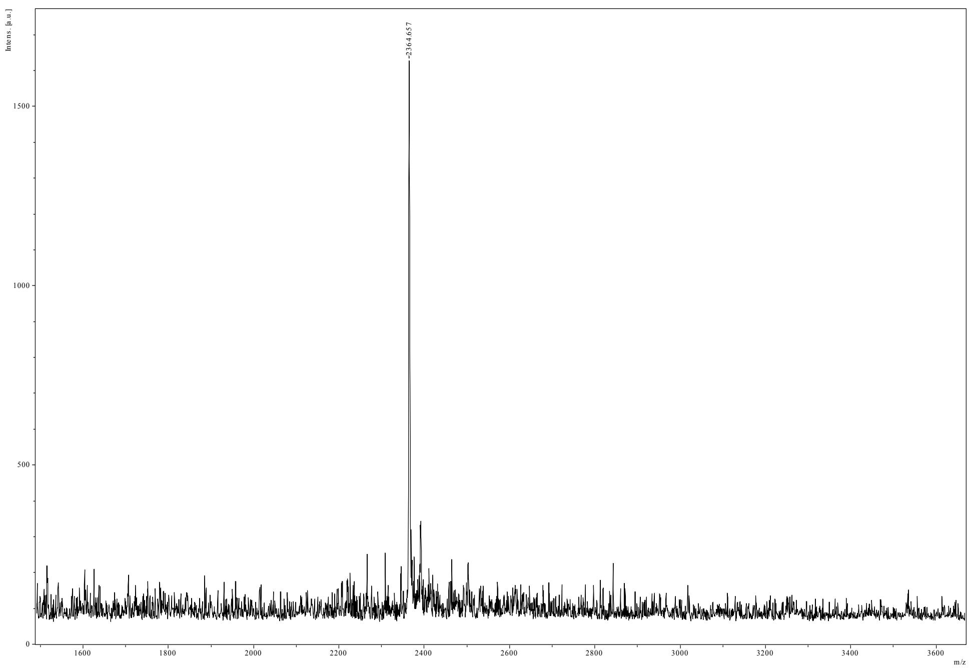

Data S40. MALDI-TOF MS of 5’-d(^{m}CG^{m}C^{F}GTG^{m}CG)-3’. Calcd. [M-H]$^{-}$: 2364.630; Found 2364.657.

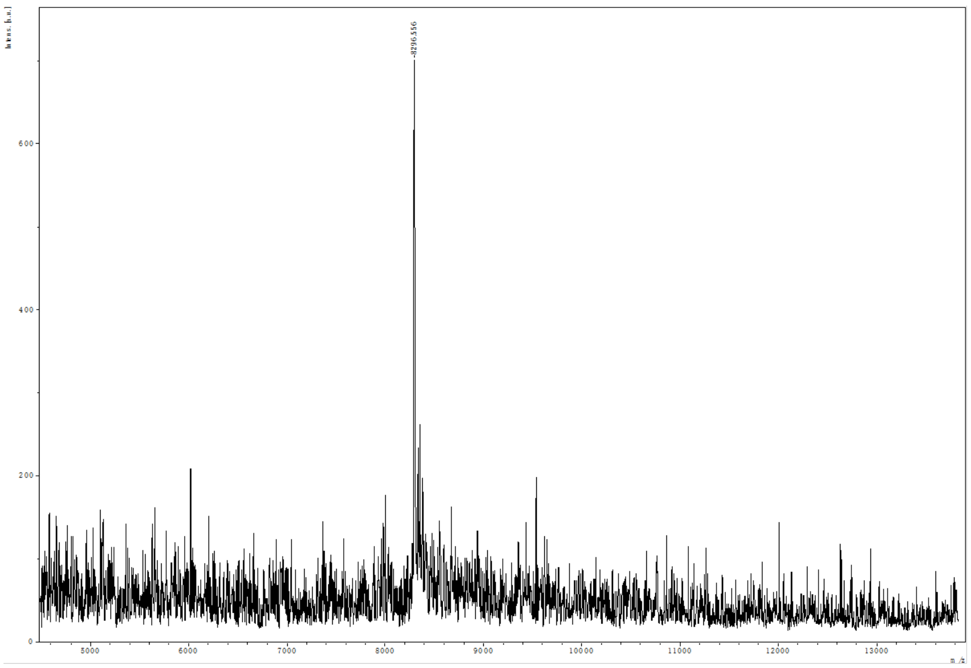

Data S41. MALDI-TOF MS of 5’-d(CGCGCGCGCGCGCGCGCGCGCGAGACA)-3’. Calcd. [M-H]$^{-}$: 8298.681; Found 8296.556.

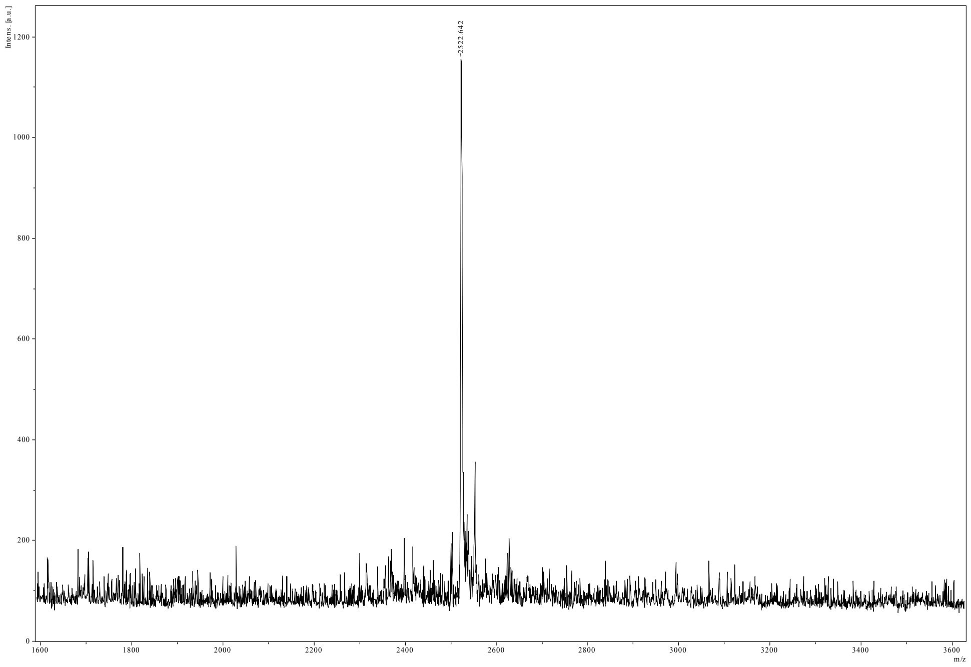

Data S42. MALDI-TOF MS of 5’-r(CGCACGCG)-3’. Calcd. [M-H]$^-$: 2523.683; Found 2522.642.

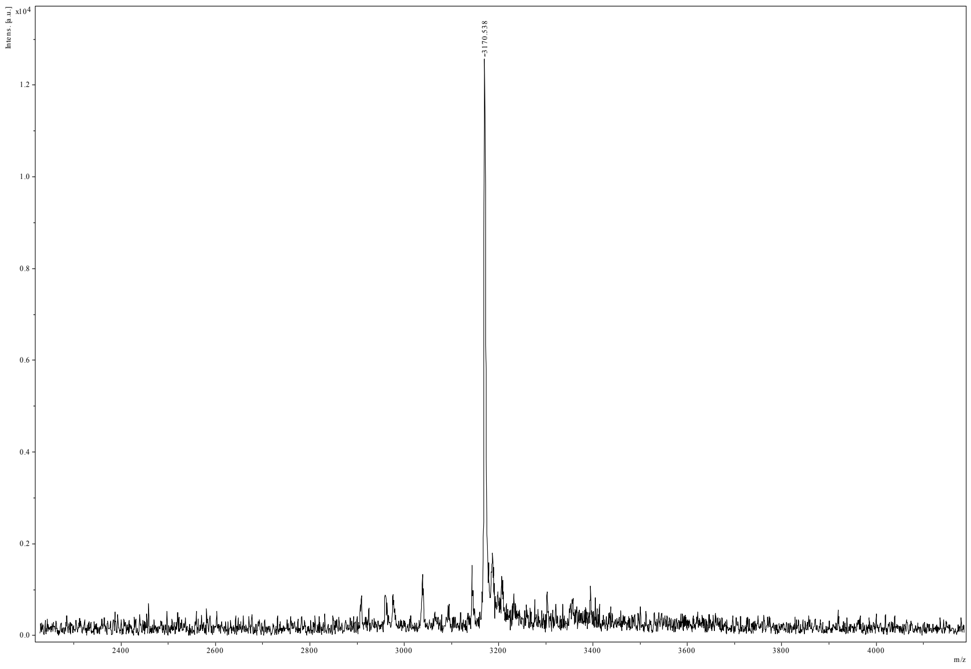

Data S43. MALDI-TOF MS of Cy3-5’-r(CGCGUGCG)-3’. Calcd. [M-H]$^-$: 3170.625; Found 3170.538.

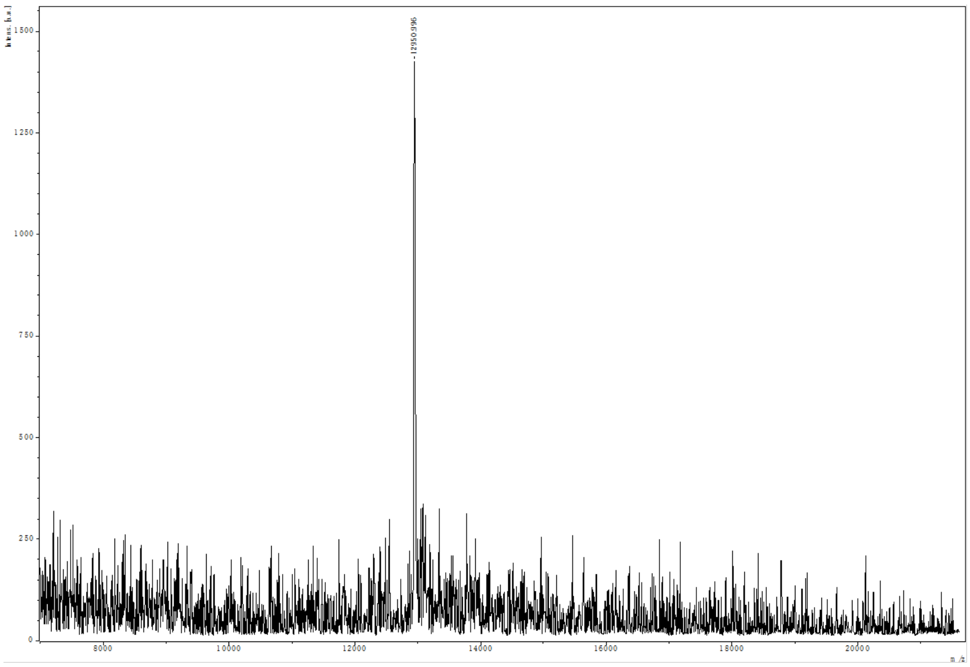

Data S44. MALDI-TOF MS of 5’-d(GATCTGAATCGAATTCGCGCGCGCGCGCGCGCGCGAGACA)-3’. Calcd. [M-H]$^{-}$: 12952.664; Found 12950.996.

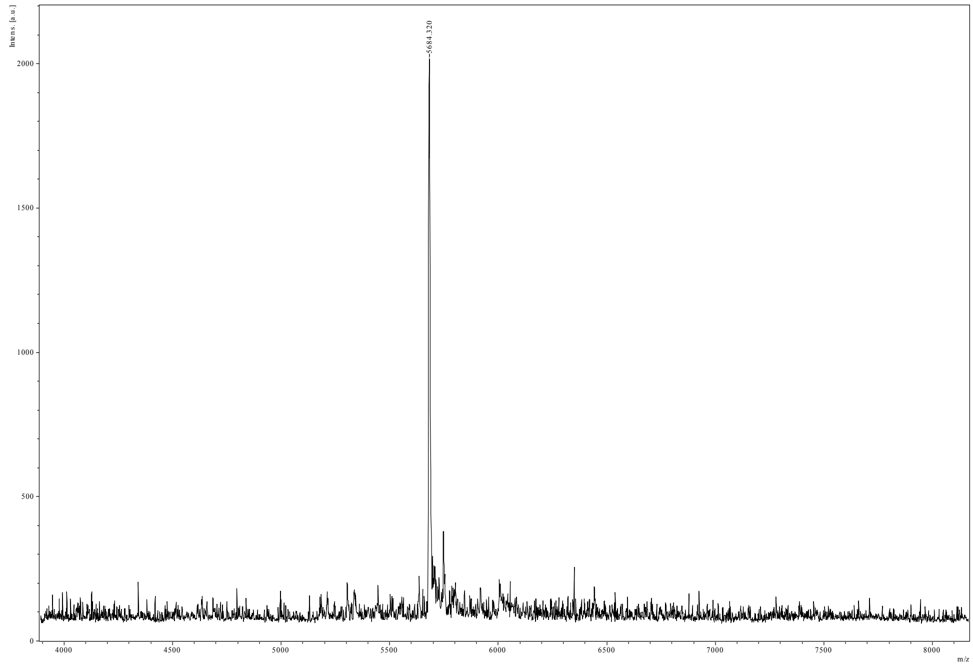

Data S45. MALDI-TOF MS of 5’-d(CCAGACATAT^{m}CG^{m}C^{F}GTG^{m}CG)-3’. Calcd. [M-H]$^{-}$: 5682.664; Found 5684.320.

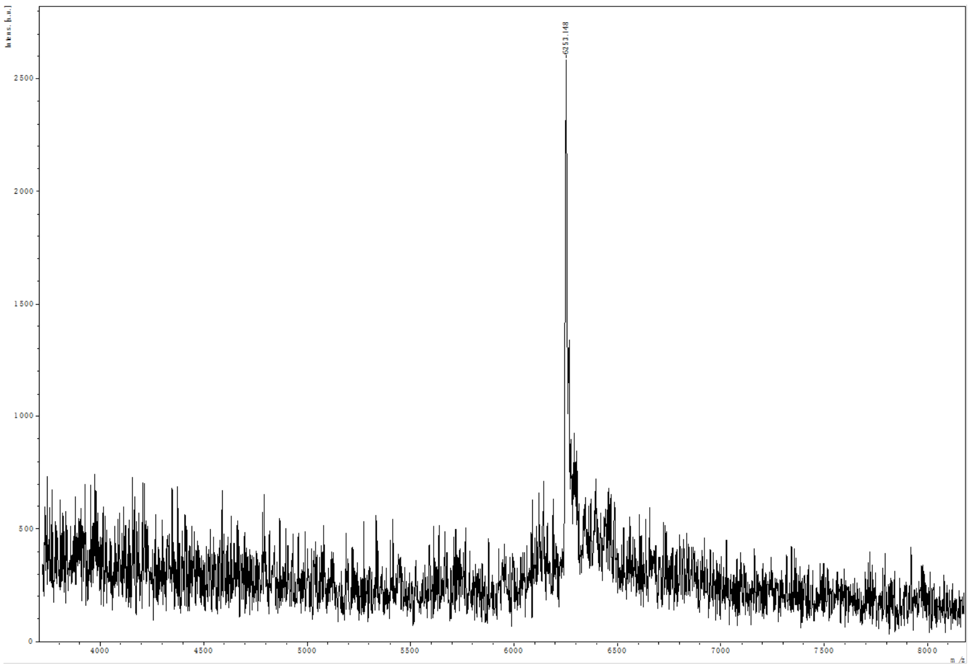

Data S46. MALDI-TOF MS of 5’-d(CG^{m}CG^{m}CG^{m}CG^{m}CGG^{m}CGG^{m}C^{m}CG^{m}CGA)-3’. Calcd. [M-H]$^{-}$: 6255.142; Found 6253.148.

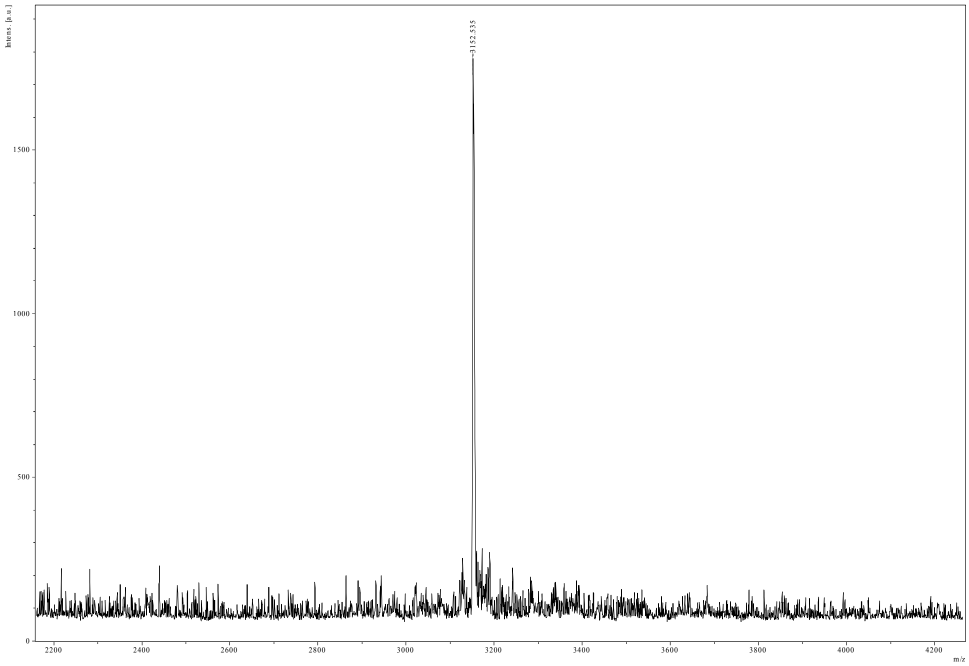

Data S47. MALDI-TOF MS of Cy3-5’-r(CGCACGCG)-3’. Calcd. [M-H]$^{-}$: 3153.669; Found 3152.535.

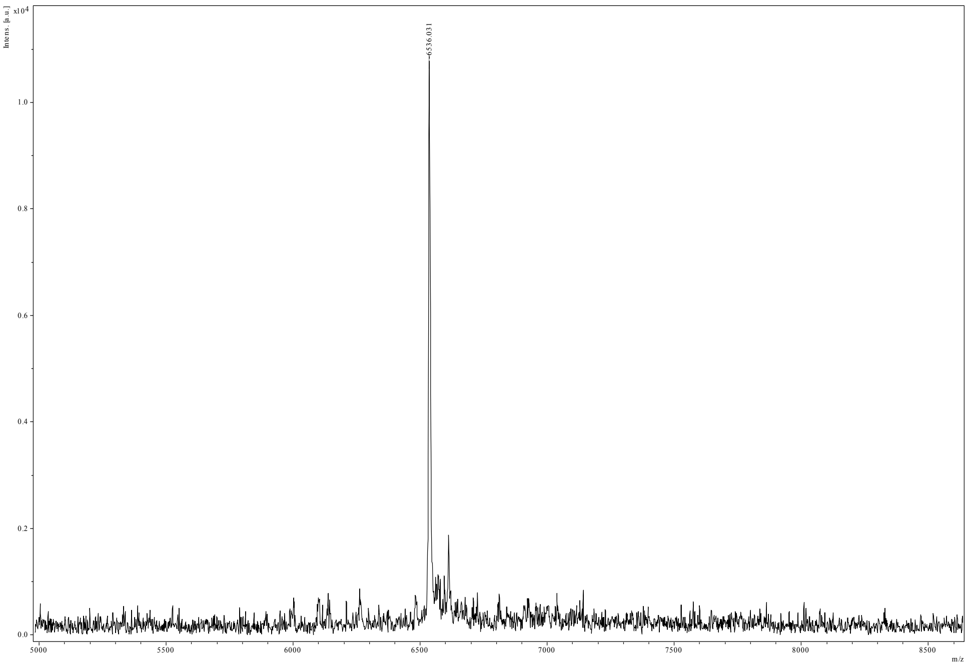

Data S48. MALDI-TOF MS of 5’-d(CGC^{m}G^{m}CG^{m}CG^{m}CGG^{m}CGGCCGCGA)-3’. Calcd. [M-H]$^{-}$: 6535.952; Found 6536.031.

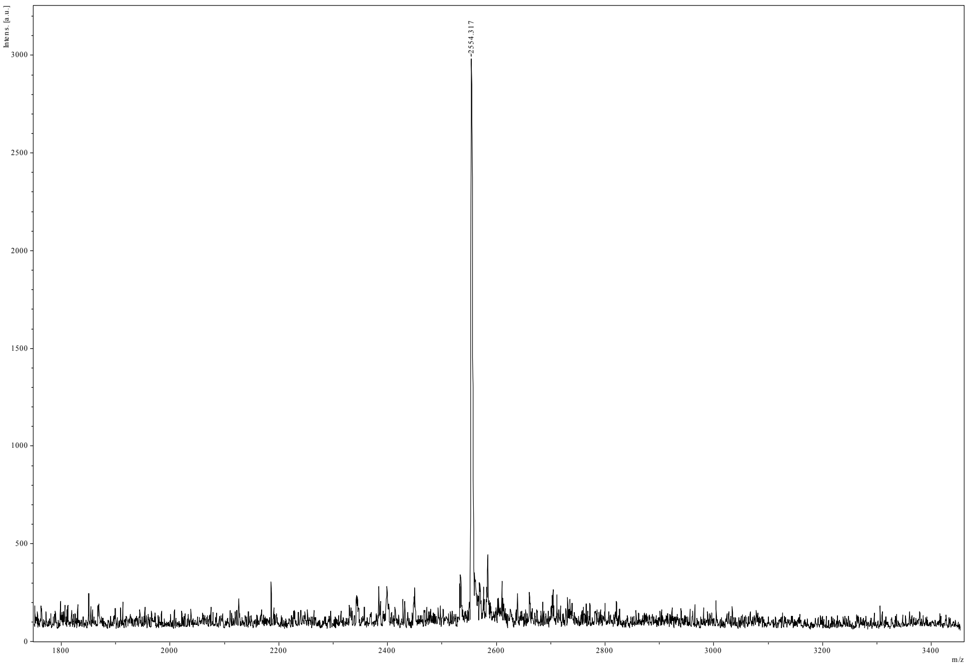

Data S49. MALDI-TOF MS of 5’-d(CGCGTGCG)-3’. Calcd. [M-H]$^{-}$: 2554.608; Found 2554.317.

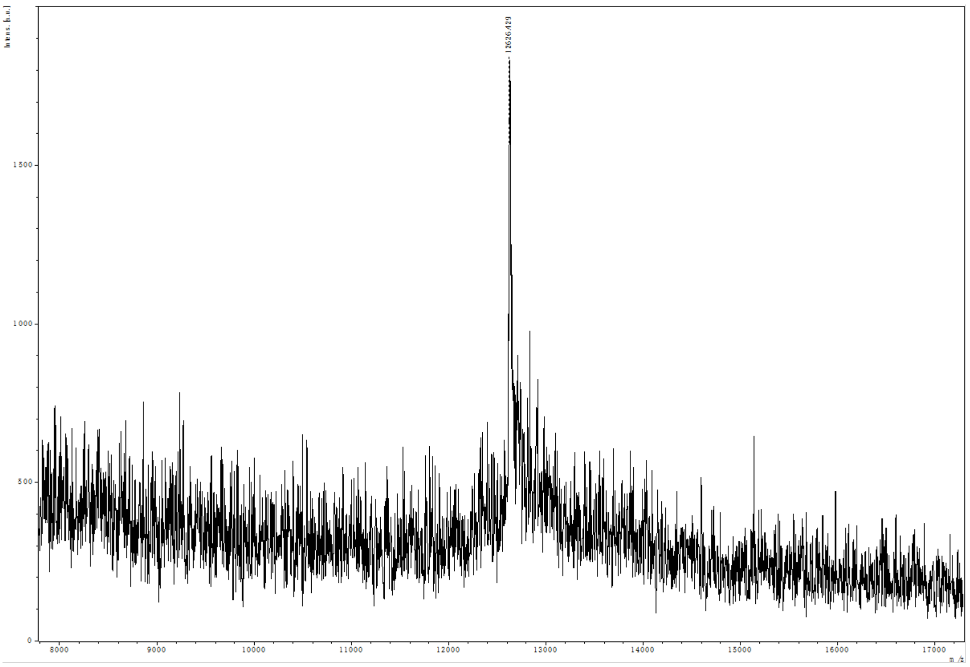

Data S50. MALDI-TOF MS of 5’-d(GGCGGGGCGCTGGGGGCGGTCG^{m}CG^{m}CG^{m}CG^{m}CGG^{m}CGG^{m}C^{m}CG^{m}CGA)-3’. Calcd. [M-H]$^{-}$: 12630.114; Found 12626.429.

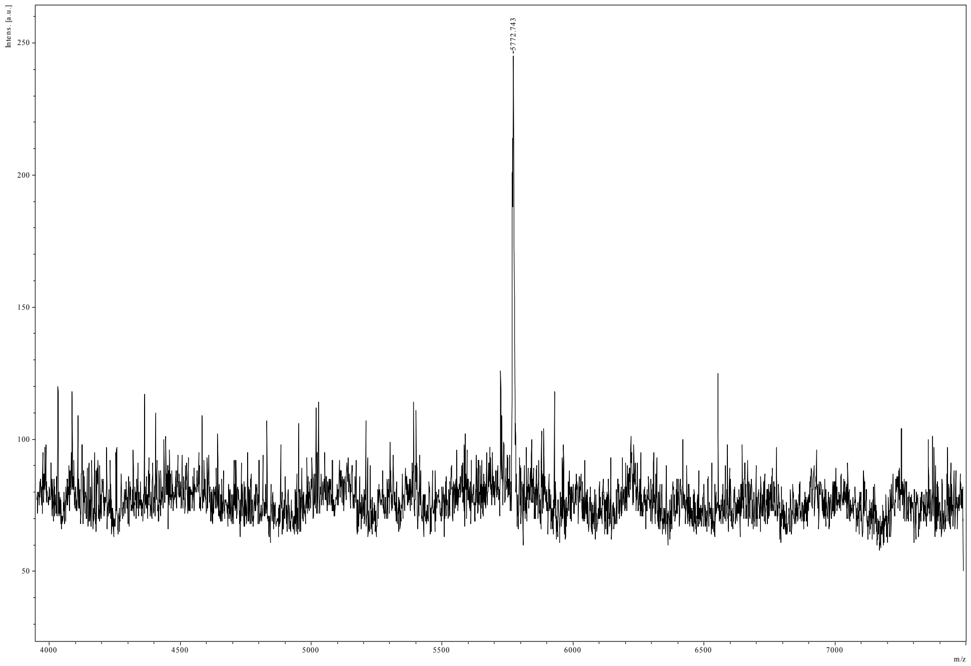

Data S51. MALDI-TOF MS of 5’-d(CCAGACATATCGCGTGCG)-3’. Calcd. [M-H]$^{-}$: 5772.617; Found 5772.743.

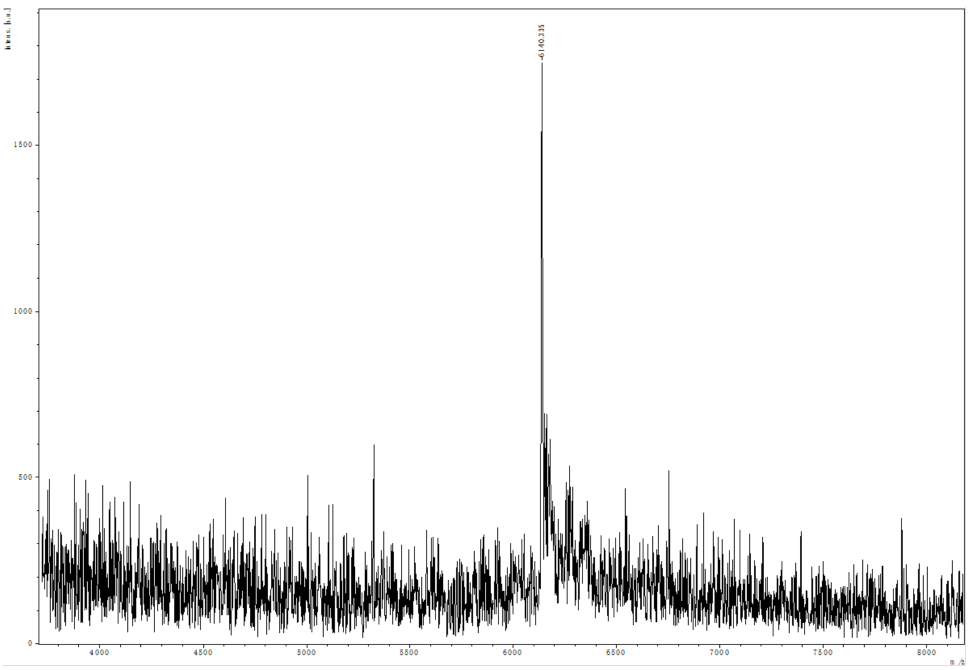

Data S52. MALDI-TOF MS of 5’-d(CGCGCGCGCGGCGGCCGCGA)-3’. Calcd. $[M-H]^-$: 6142.514; Found 6140.335.

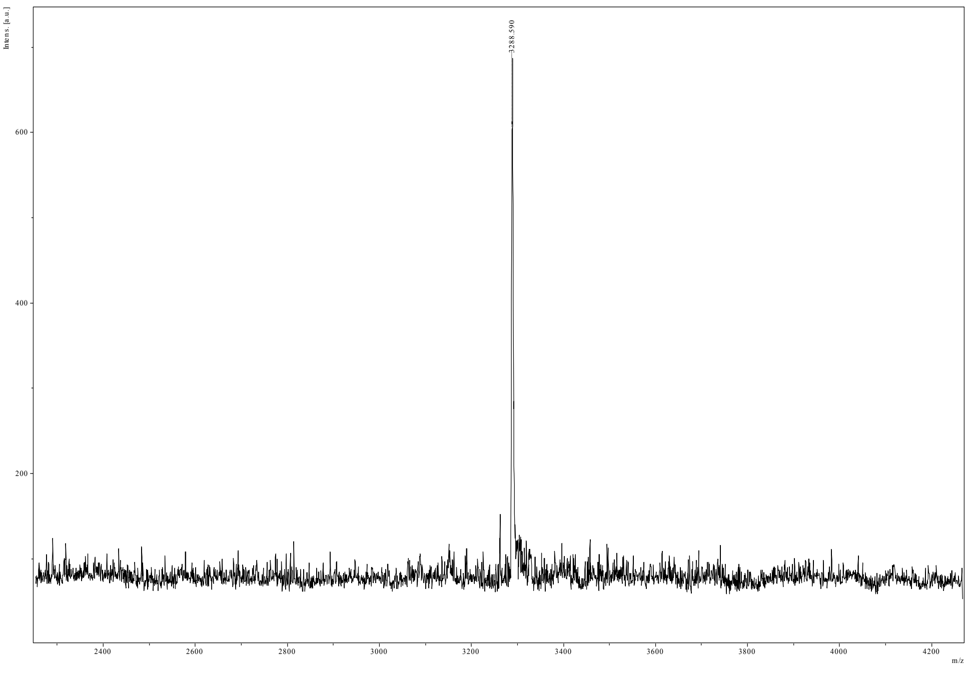

Data S53. MALDI-TOF MS of Cy3-5’-r(C^FGCAC^FGCG)-3’. Calcd. $[M-H]^-$: 3289.079; Found 3288.590.

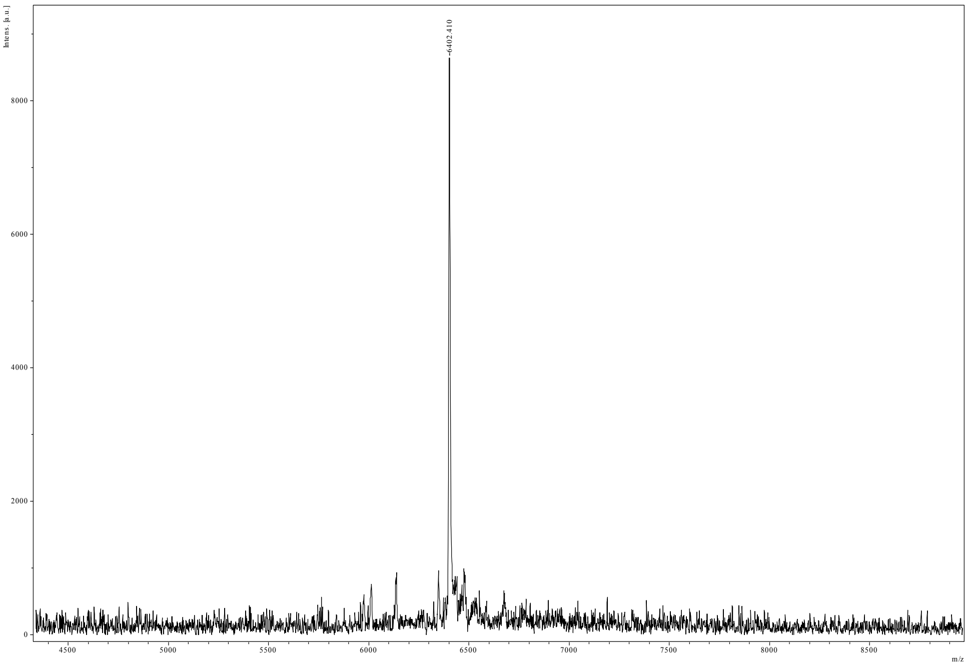

Data S54. MALDI-TOF MS of 5’-r(UCGCGGCCGCCGCGCGCGCG)-3’. Calcd. [M-H]$^-$: 6402.981; Found 6402.410.

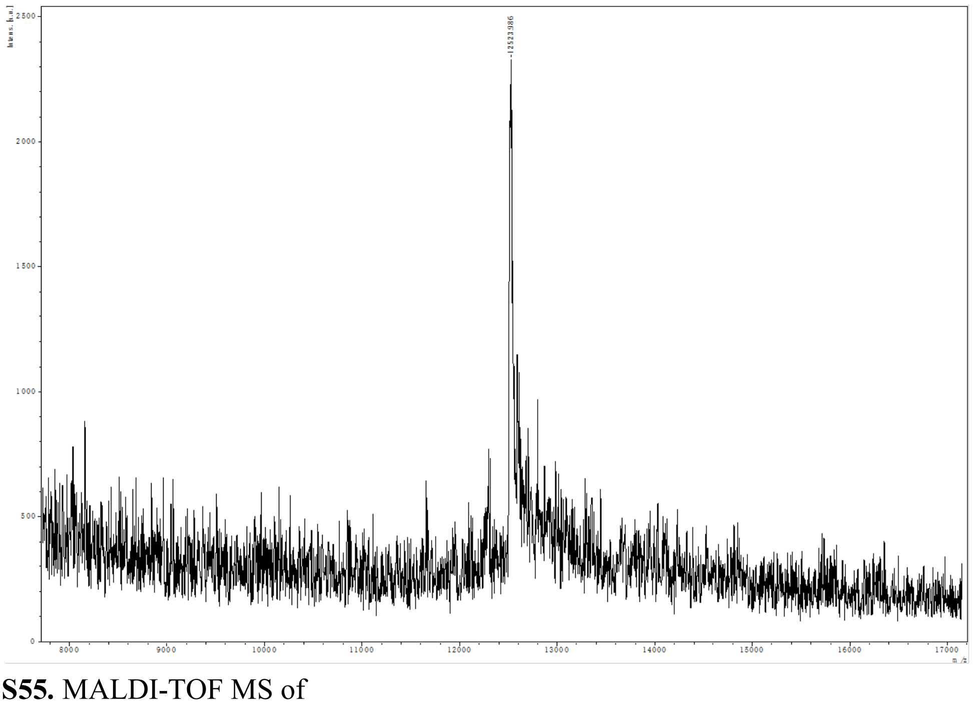

Data S55. MALDI-TOF MS of 5’-d(GGCGGGGCGCTGGGGGCGGTCGCGCGCGGCGGCCGCGA)-3’. Calcd. [M-H]$^-$: 12526.701; Found 12523.986.

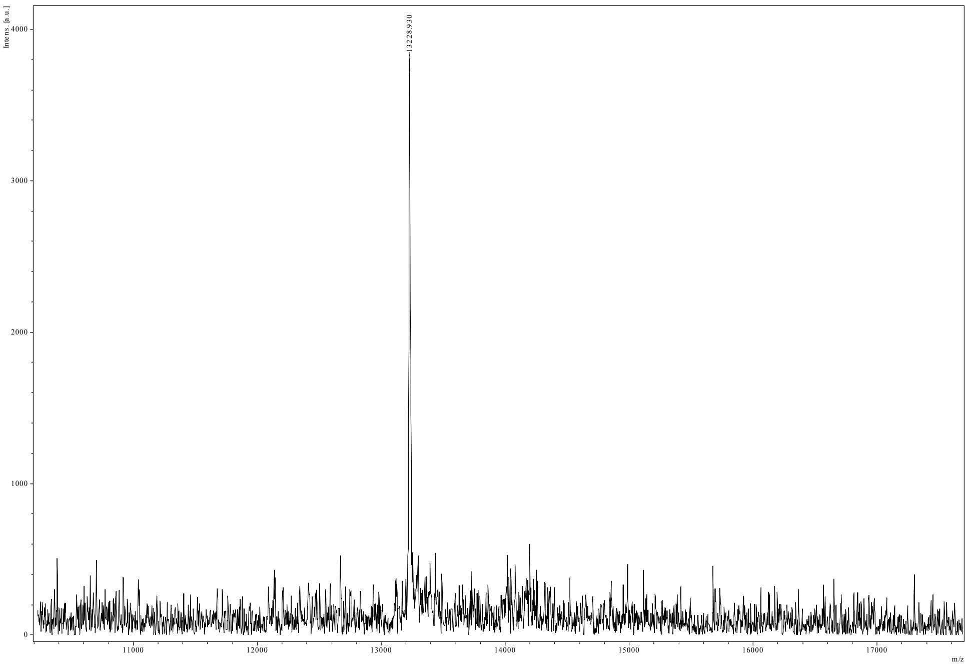

Data S56. MALDI-TOF MS of 5'-d(GGCGGGGCGCTGGGGGCGGTCG^{m}CG^{m}CG^{m}CG^{m}CGG^{m}CGGCCGCGA)-3'. Calcd. [M-H]$^{-}$: 13229.092; Found 13228.930.

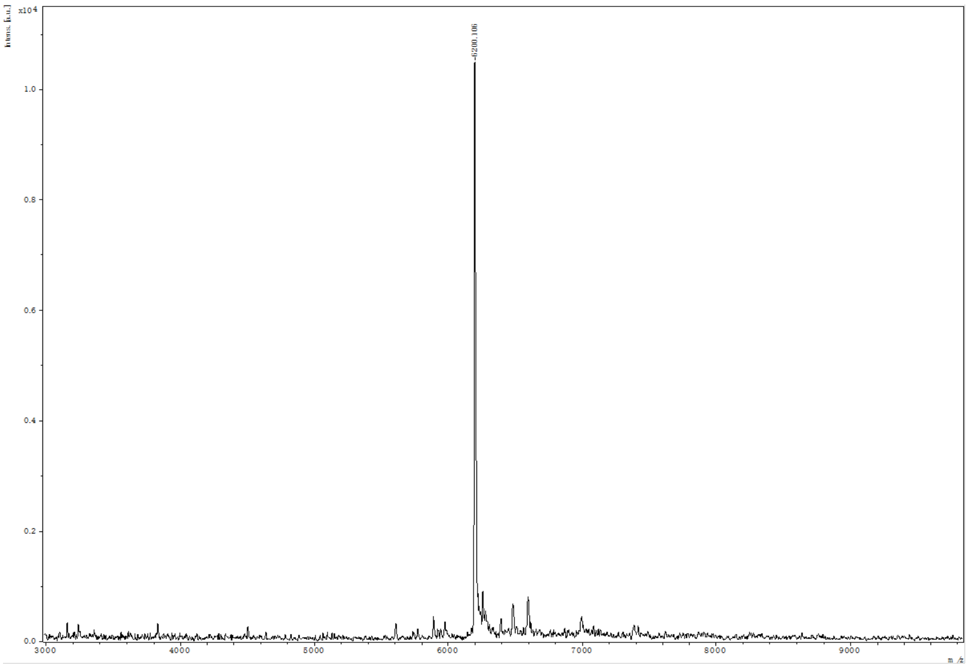

Data S57. MALDI-TOF MS of 5'-d(CG^{m}CG^{m}CG^{m}CG^{m}CGGCGGCCGCGA)-3'. Calcd. [M-H]$^{-}$: 6201.257; Found 6200.106.

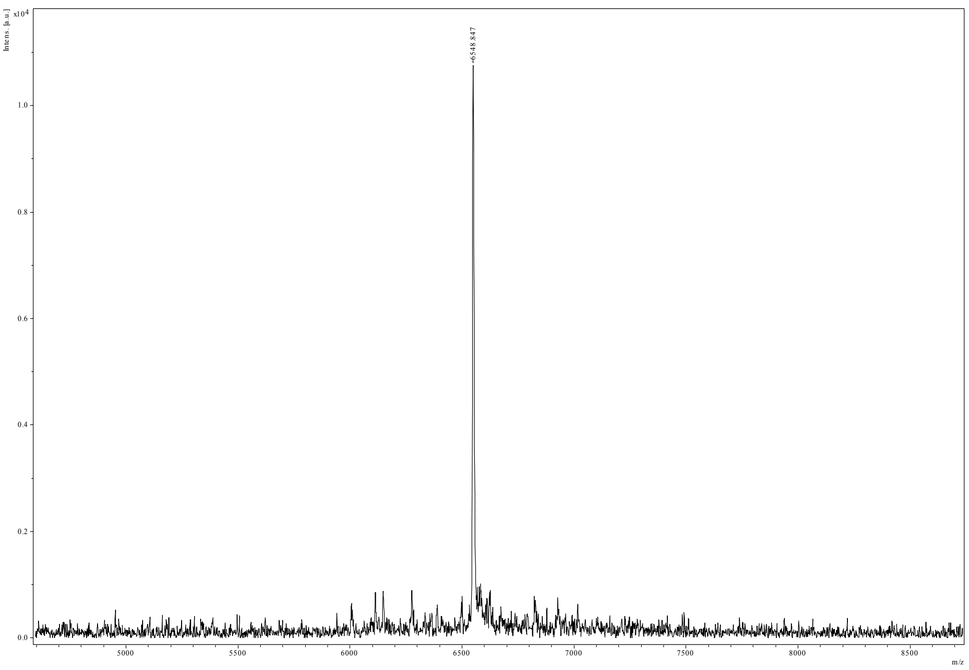

Data S58. MALDI-TOF MS of 5’-d(CG^{m}CG^{m}CG^{m}CG^{m}CGG^{m}CGG^{m}CCGCGA)-3’. Calcd. [M-H]$^{-}$: 6549.903; Found 6548.847.

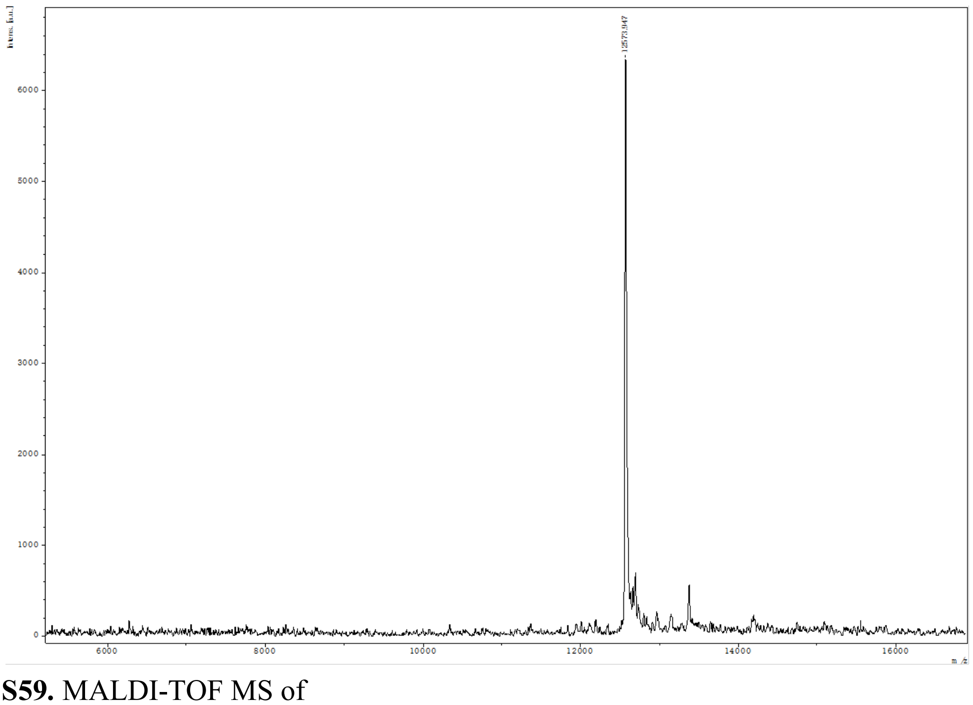

Data S59. MALDI-TOF MS of 5’-d(GGCGGGGCGCTGGGGGCGGTCG^{m}CG^{m}CG^{m}CG^{m}CGGCGGCCGCGA)-3’. Calcd. [M-H]$^{-}$: 12579.227; Found 12573.947.

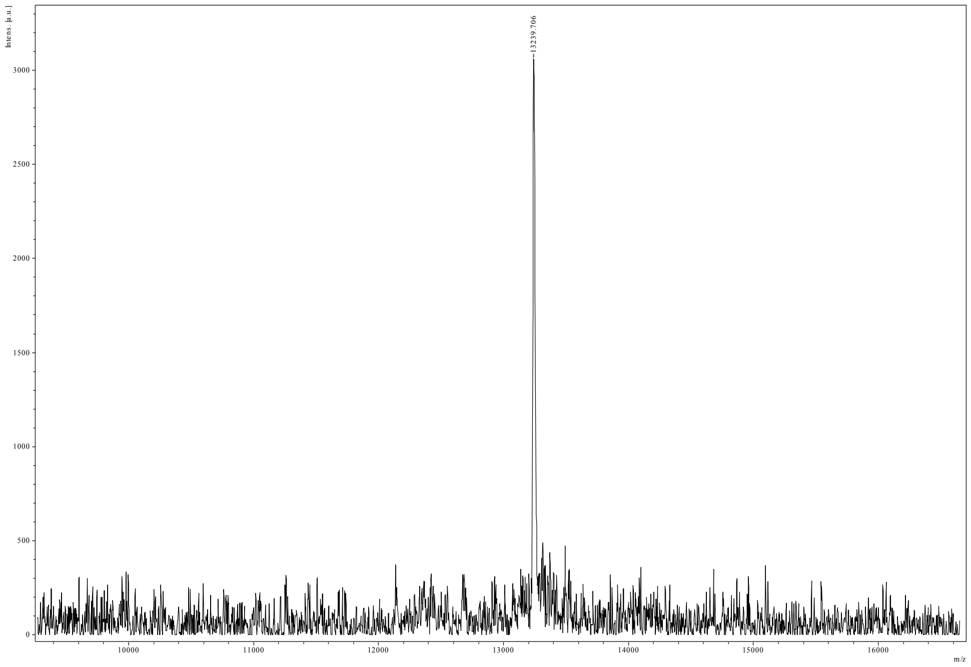

Data S60. MALDI-TOF MS of 5’-d(GGCGGGGCGCTGGGGGCGGTCG^{m}CG^{m}CG^{m}CG^{m}CGG^{m}CGG^{m}CCGCGA)-3’. Calcd. [M-H]$^{-}$: 13243.916; Found 13239.706.

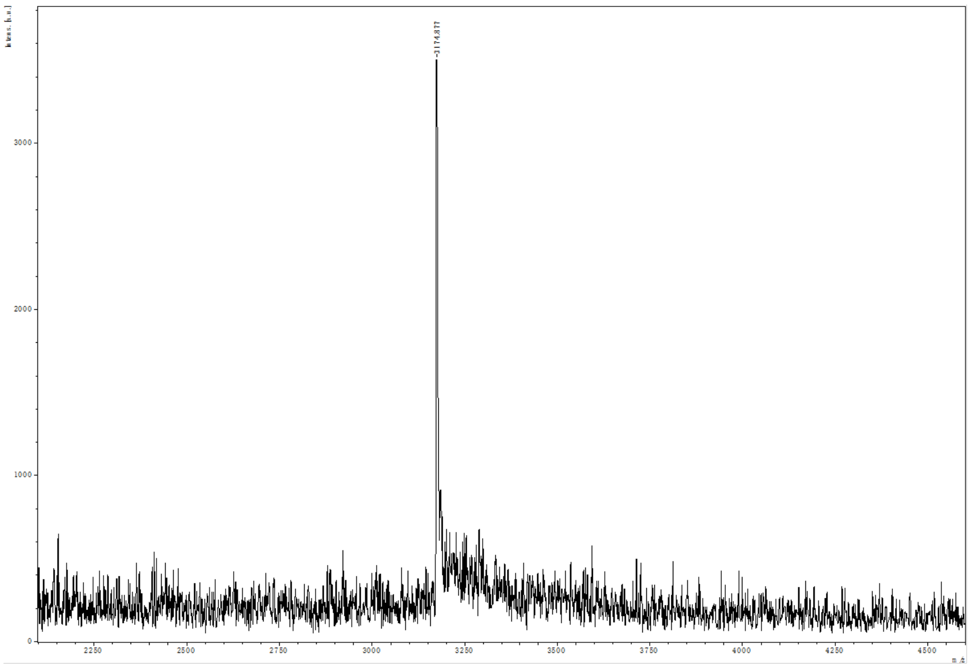

Data S61. MALDI-TOF MS of 5’-r(AGCUCGCGCG)-3’. Calcd. [M-H]$^{-}$: 3175.923; Found 3174.877.

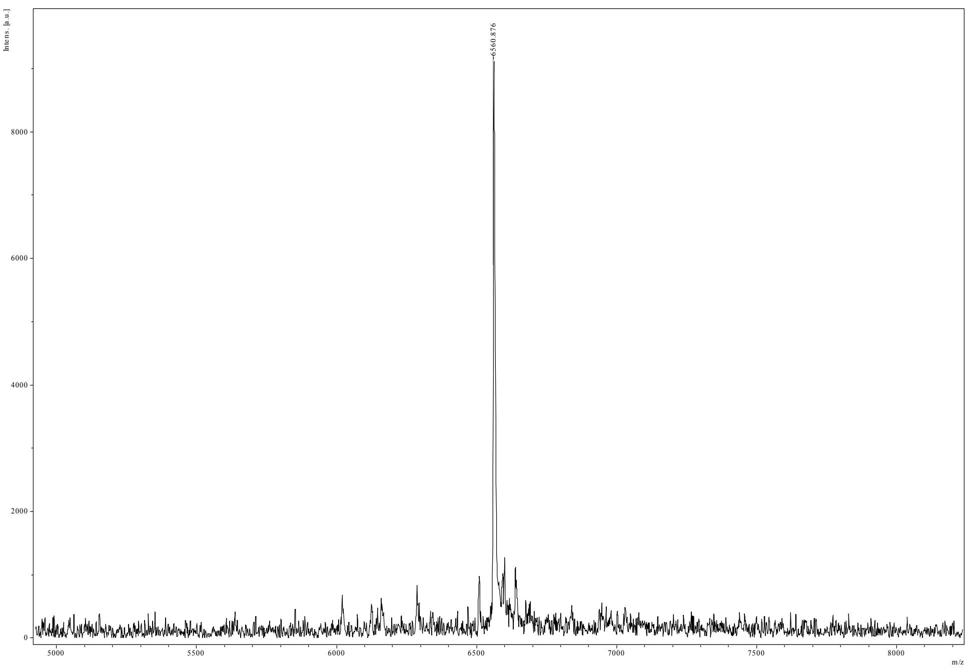

Data S62. MALDI-TOF MS of 5’-d(CG^{m}CG^{m}CG^{m}CG^{m}CGG^{m}CGG^{m}C^{m}CGCGA)-3’. Calcd. [M-H]$^{-}$: 6563.914; Found 6560.876.

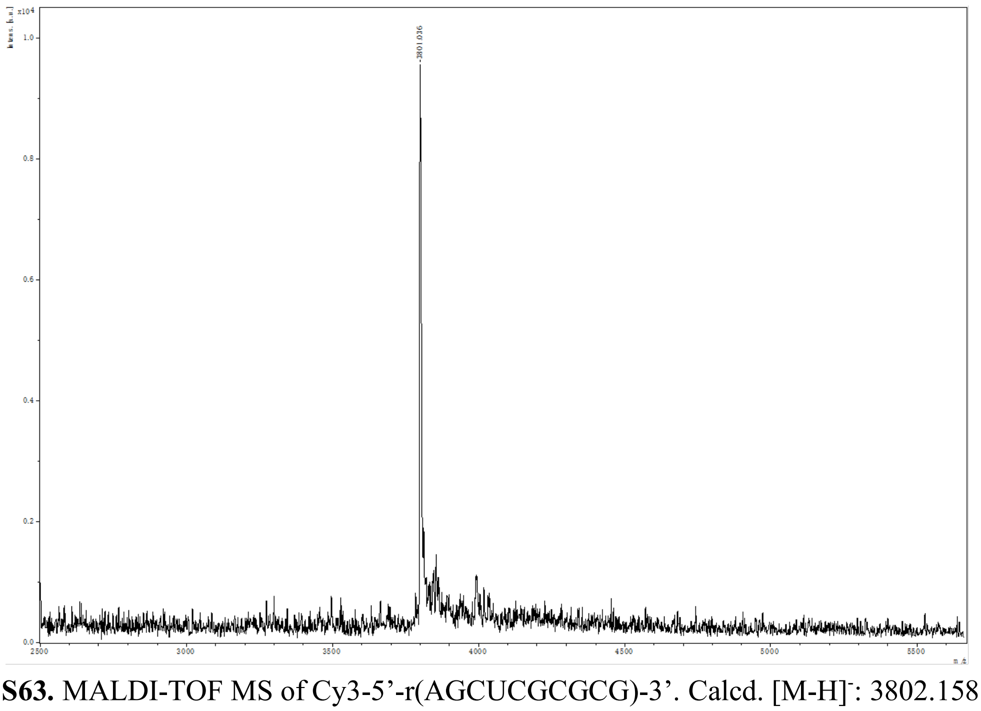

Data S63. MALDI-TOF MS of Cy3-5’-r(AGCUCGCGCG)-3’. Calcd. [M-H]$^{-}$: 3802.158; Found 3801.036.

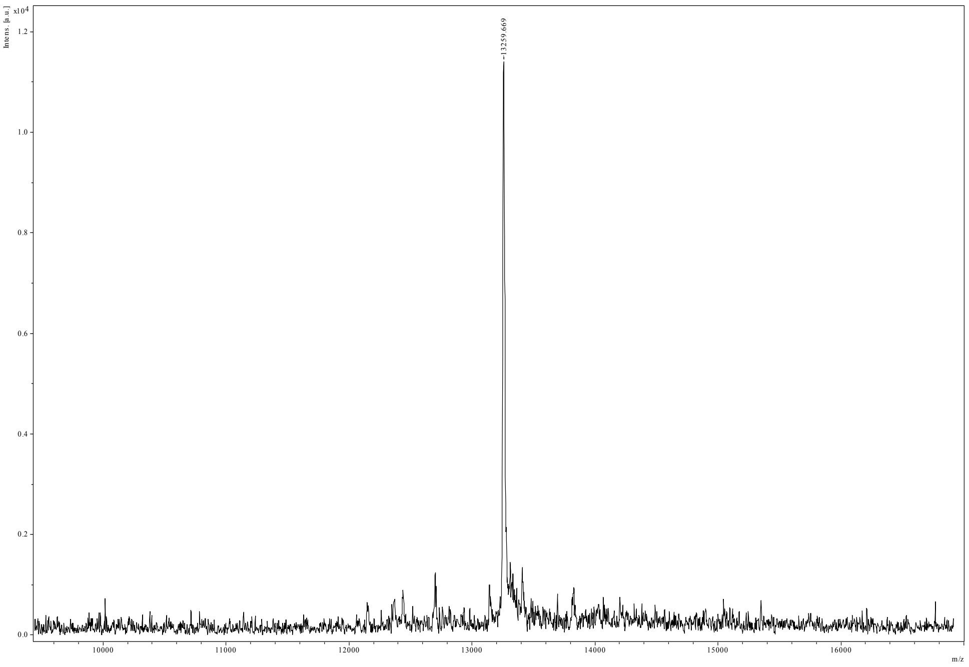

Data S64. MALDI-TOF MS of 5'-d(GGCGGGGCGCTGGGGGCGGTCG^{m}CG^{m}CG^{m}CG^{m}CGG^{m}CGG^{m}C^{m}CGCGA)-3'. Calcd. [M-H]$^{-}$: 13257.951; Found 13259.669.

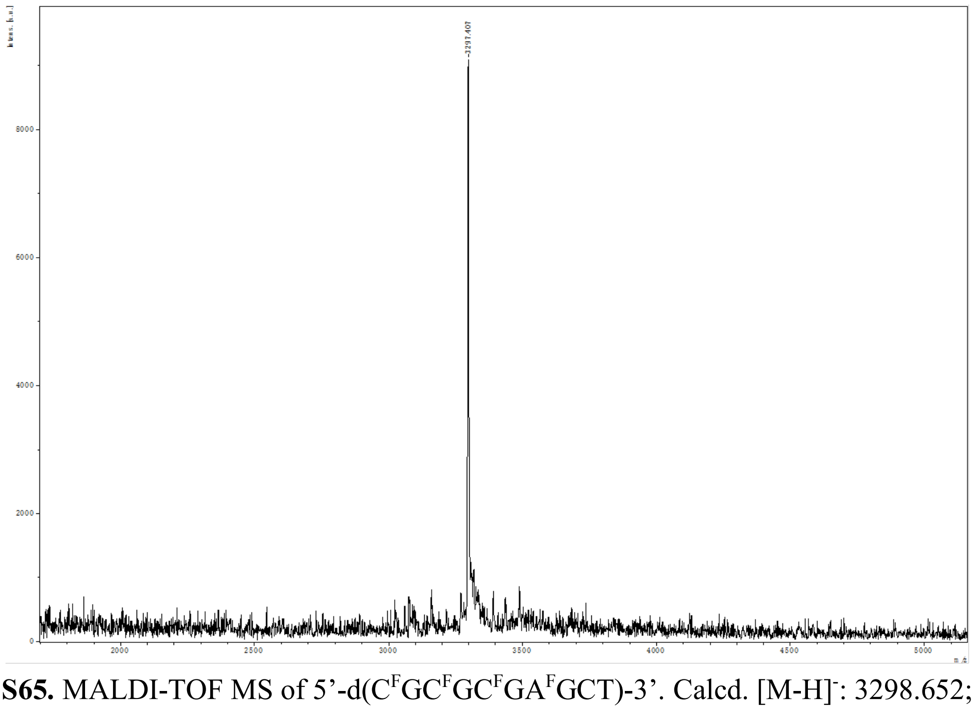

Data S65. MALDI-TOF MS of 5'-d(C^{F}GC^{F}GC^{F}GA^{F}GCT)-3'. Calcd. [M-H]$^{-}$: 3298.652; Found 3297.407.

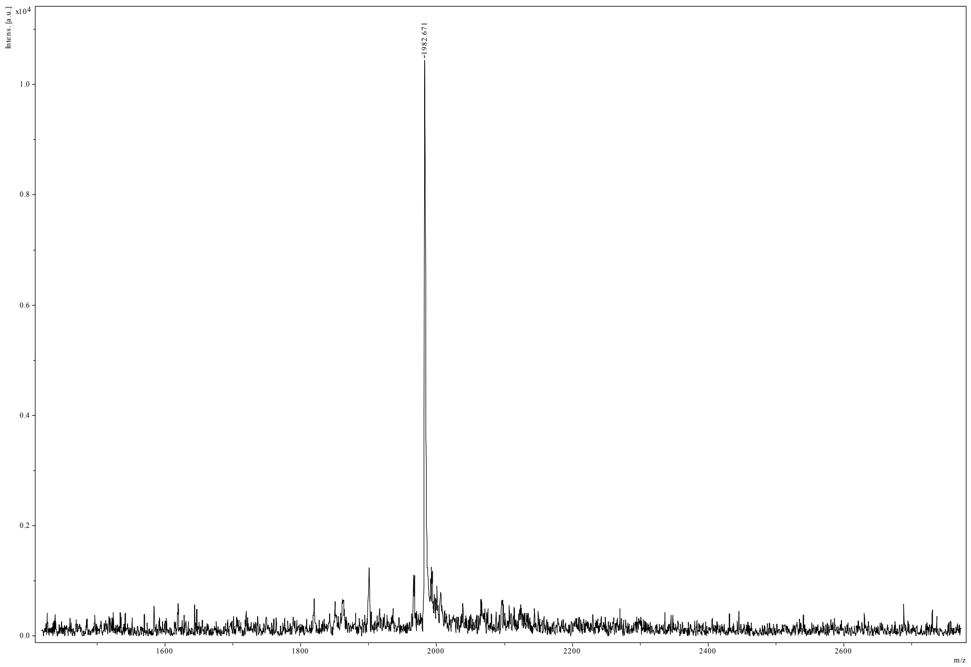

Data S66. MALDI-TOF MS of 5’-d(^{m}CA^{m}C^{F}G^{m}CG)-3’. Calcd. [M-H]$^{-}$: 1983.247; Found 1982.671.

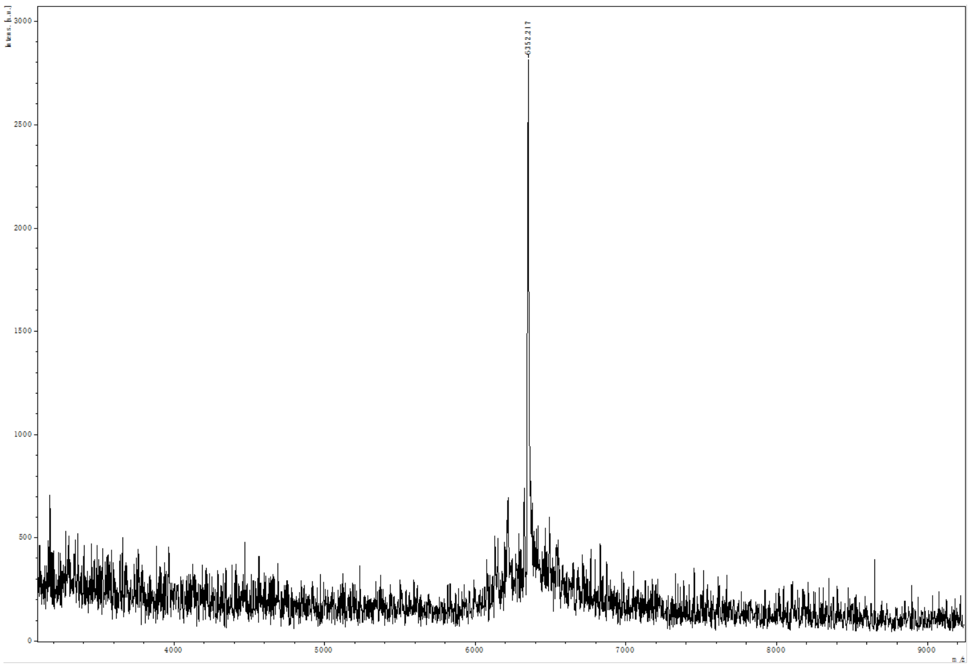

Data S67. MALDI-TOF MS of 5’-d(CCAGACATATC^{F}GC^{F}GC^{F}GA^{F}GCT)-3’. Calcd. [M-H]$^{-}$: 6354.226; Found 6352.217.

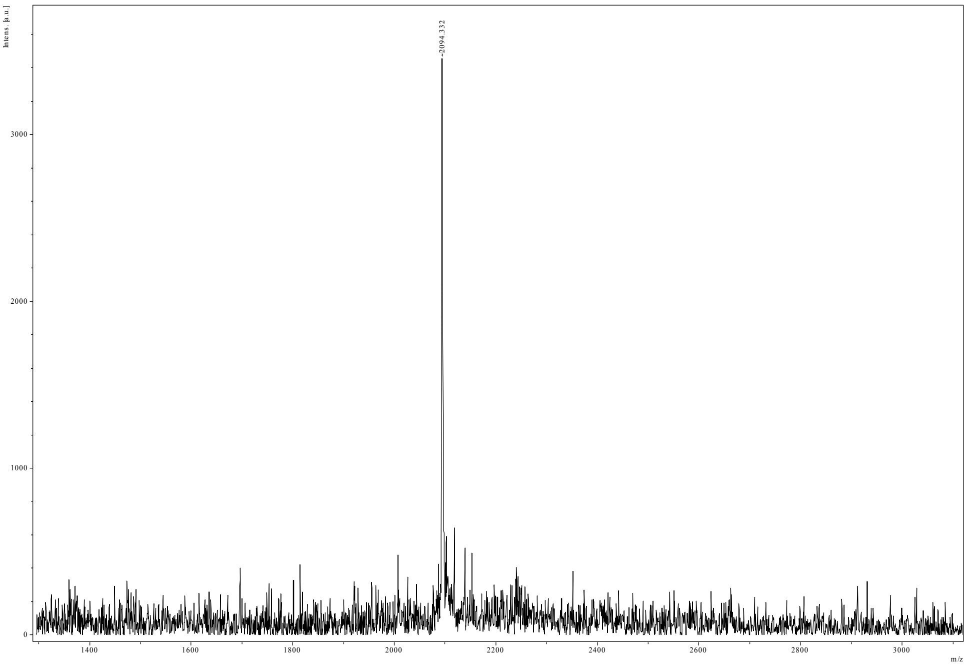

Data S68. MALDI-TOF MS of 5'-r(C^{F}GC^{F}GU^{F}G)-3'. Calcd. [M-H]$^{-}$: 2094.217; Found 2094.332.

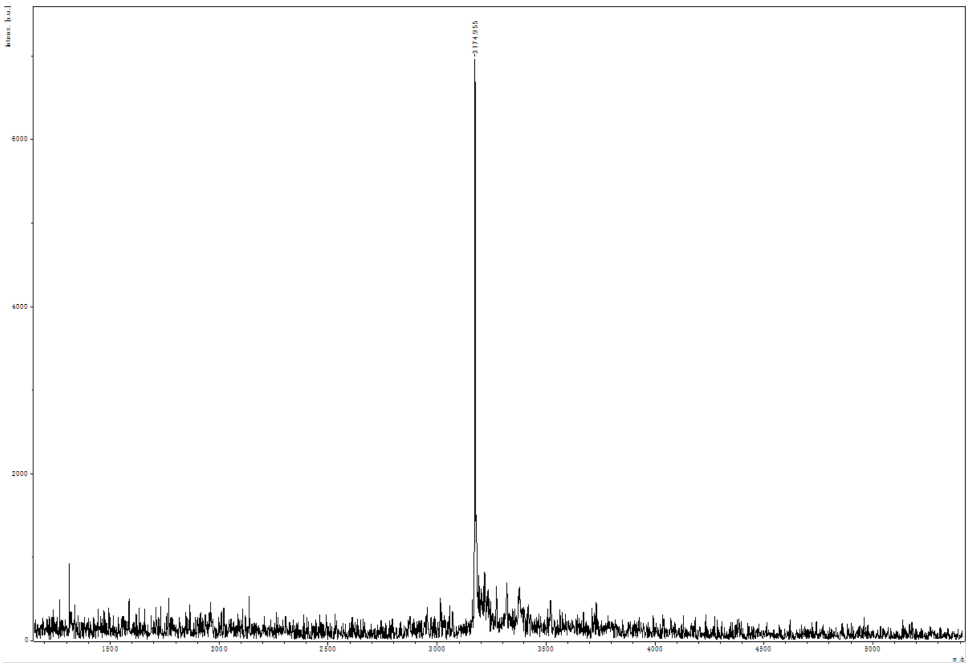

Data S69. MALDI-TOF MS of 5'-r(AGCCGGCUCG)-3'. Calcd. [M-H]$^{-}$: 3175.820; Found 3174.955.

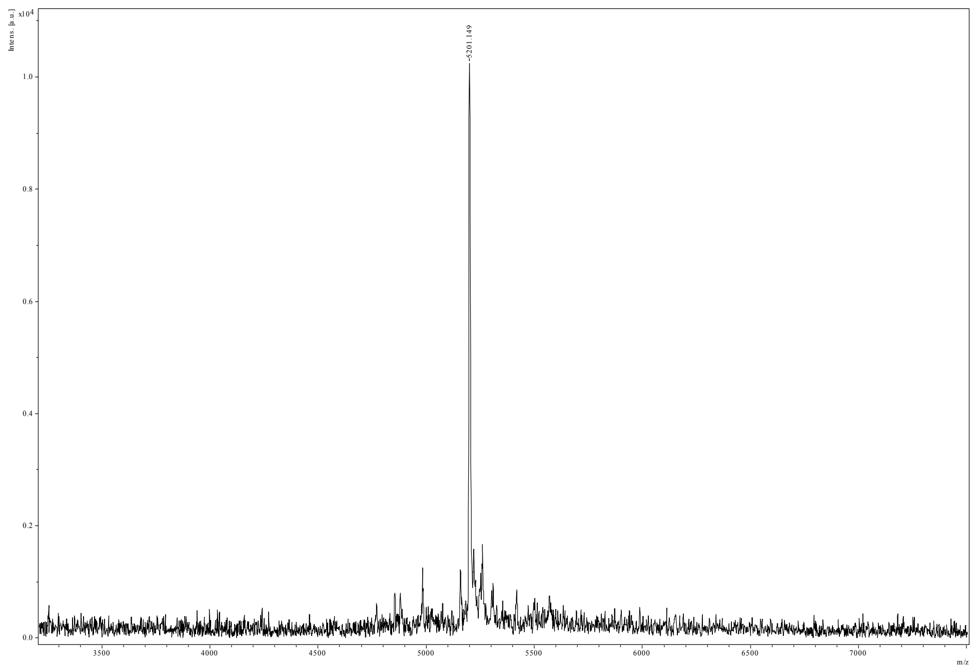

Data S70. MALDI-TOF MS of 5’-d(CCAGACATAT^{m}CA^{m}C^{F}G^{m}CG)-3’. Calcd. [M-H]$^{-}$: 5201.254; Found 5201.149.

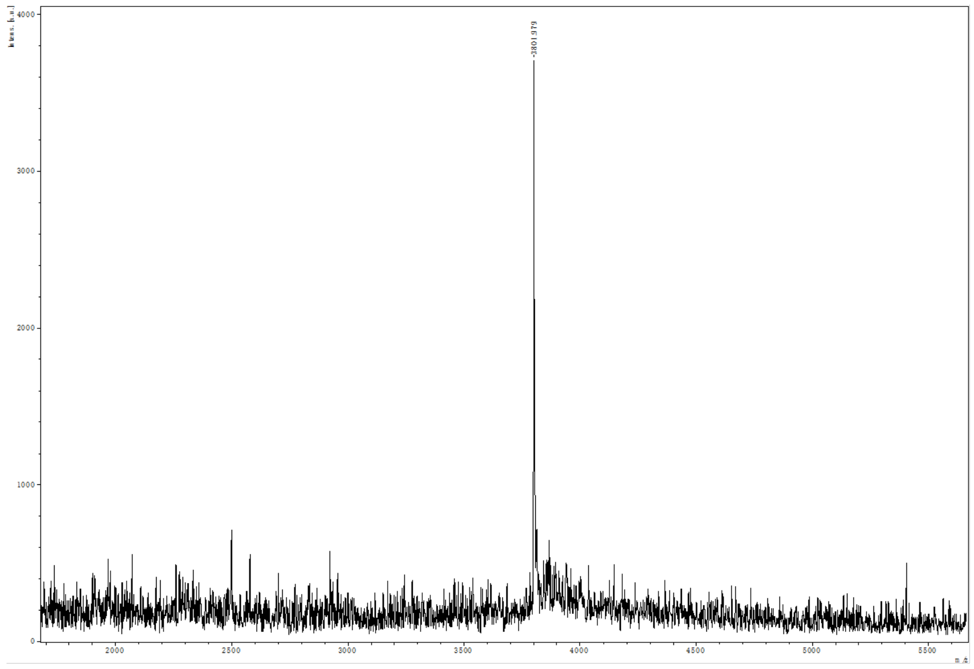

Data S71. MALDI-TOF MS of Cy3-5’-r(AGCCGGCUCG)-3’. Calcd. [M-H]$^{-}$: 3801.513; Found 3801.979.

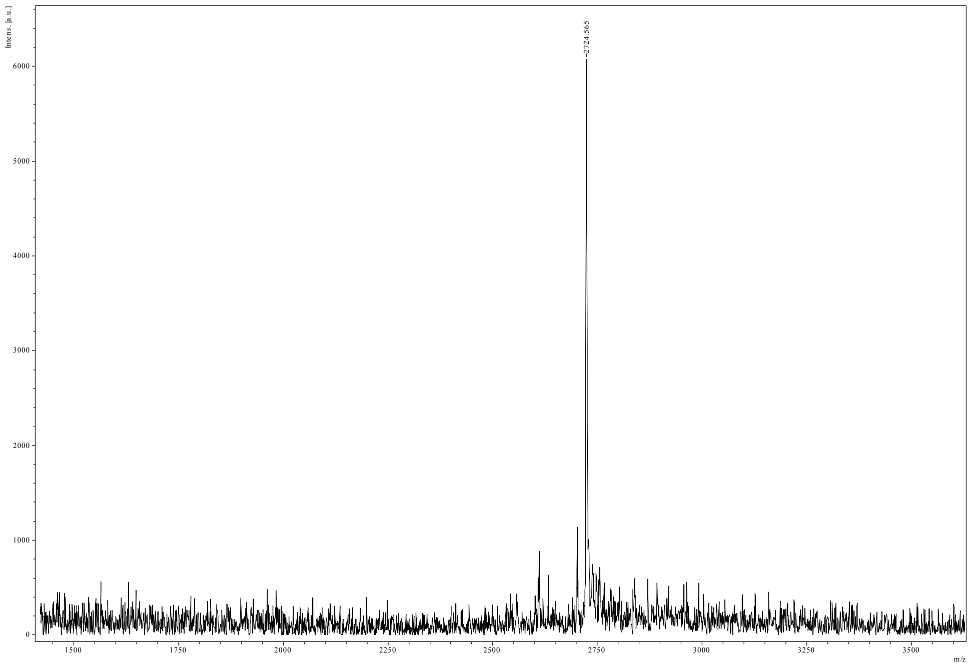

Data S72. MALDI-TOF MS of Cy3-5'-r($C^{F}GC^{F}GU^{F}G$)-3'. Calcd. [M-H]$^{-}$: 2724.284; Found 2724.565.

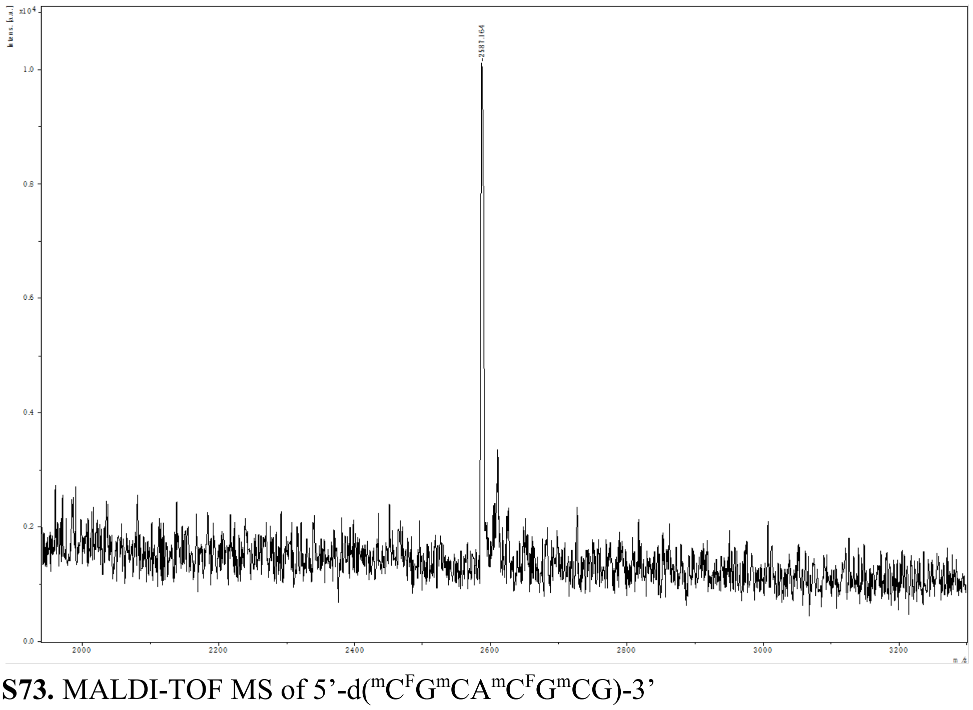

Data S73. MALDI-TOF MS of 5'-d($^{m}C^{F}G^{m}CA^{m}C^{F}G^{m}CG$)-3'
. Calcd. [M-H]$^{-}$: 2587.662; Found 2587.164.

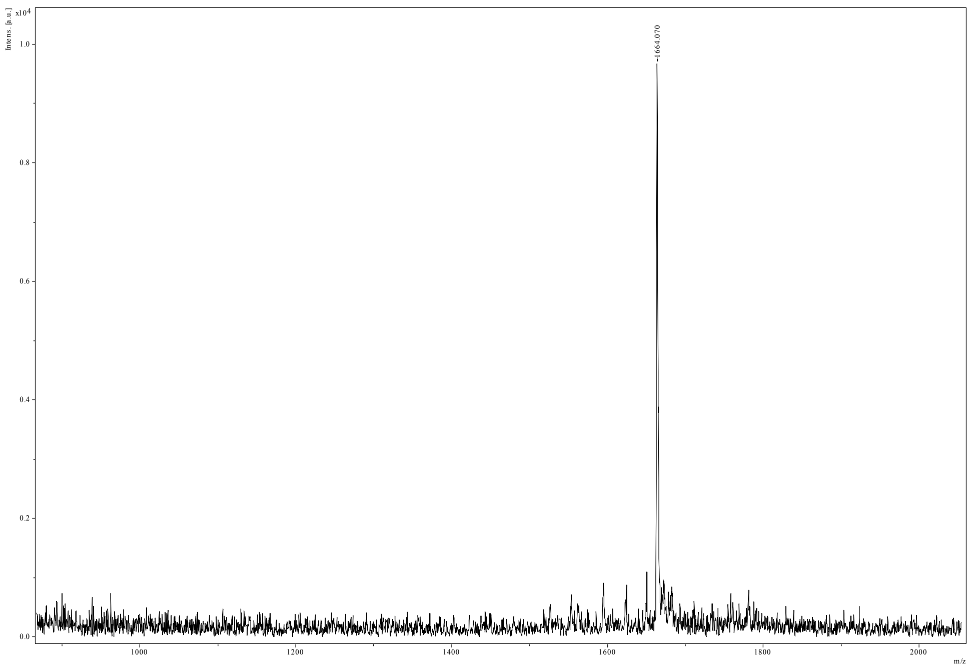

Data S74. MALDI-TOF MS of 5’-d(A^{m}C^{F}G^{m}CG)-3’. Calcd. [M-H]$^{-}$: 1664.034; Found 1664.070.

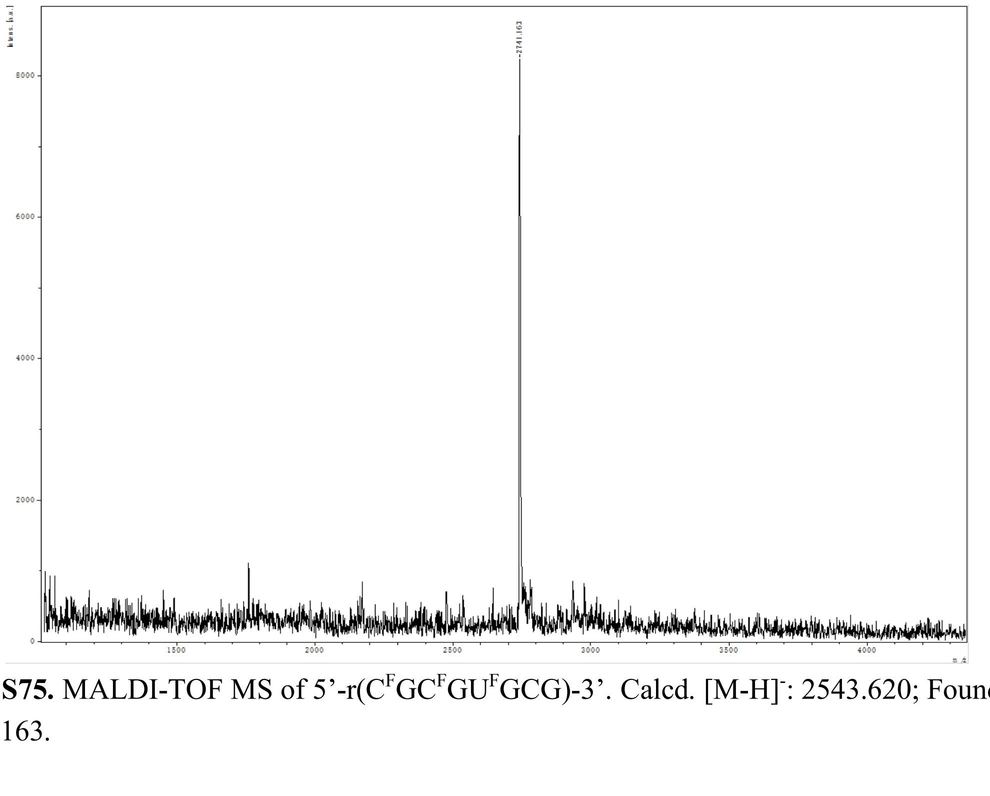

Data S75. MALDI-TOF MS of 5’-r(C^{F}GC^{F}GU^{F}GCG)-3’. Calcd. [M-H]$^{-}$: 2543.620; Found 2741.163.

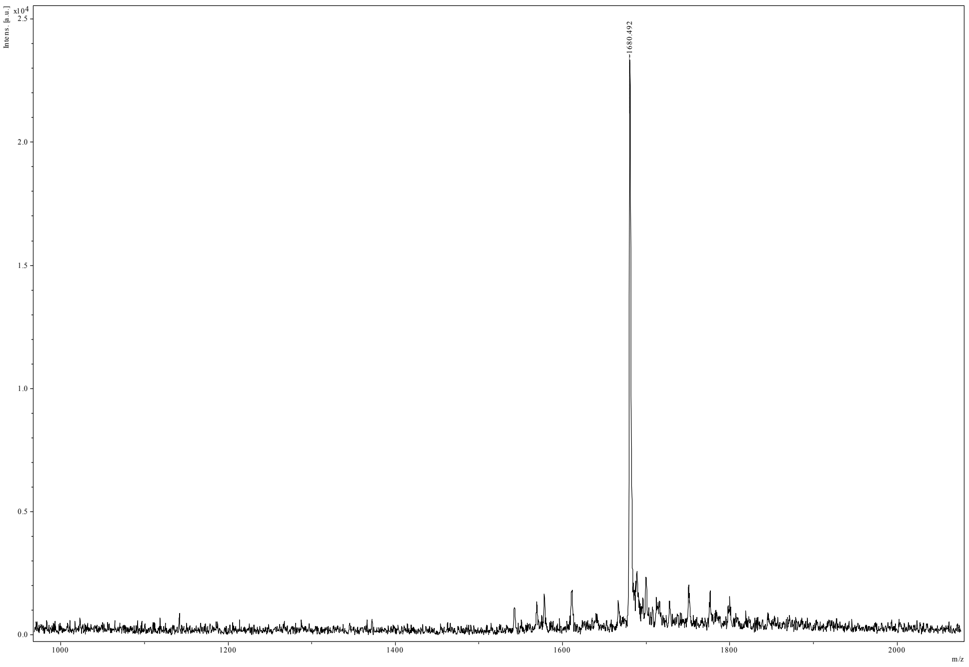

Data S76. MALDI-TOF MS of 5’-r(C^{F}GC^{F}GU)-3’. Calcd. [M-H]$^{-}$: 1681.076; Found 1680.492.

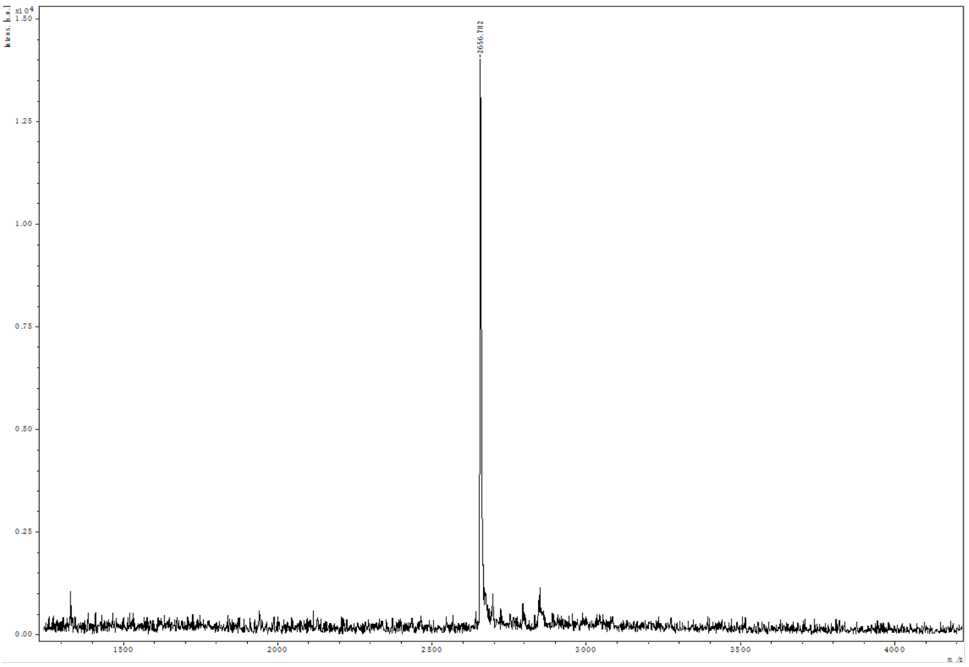

Data S77. MALDI-TOF MS of 5’-r(C^{F}GCAC^{F}GCG)-3’. Calcd. [M-H]$^{-}$: 2657.095; Found 2656.782.

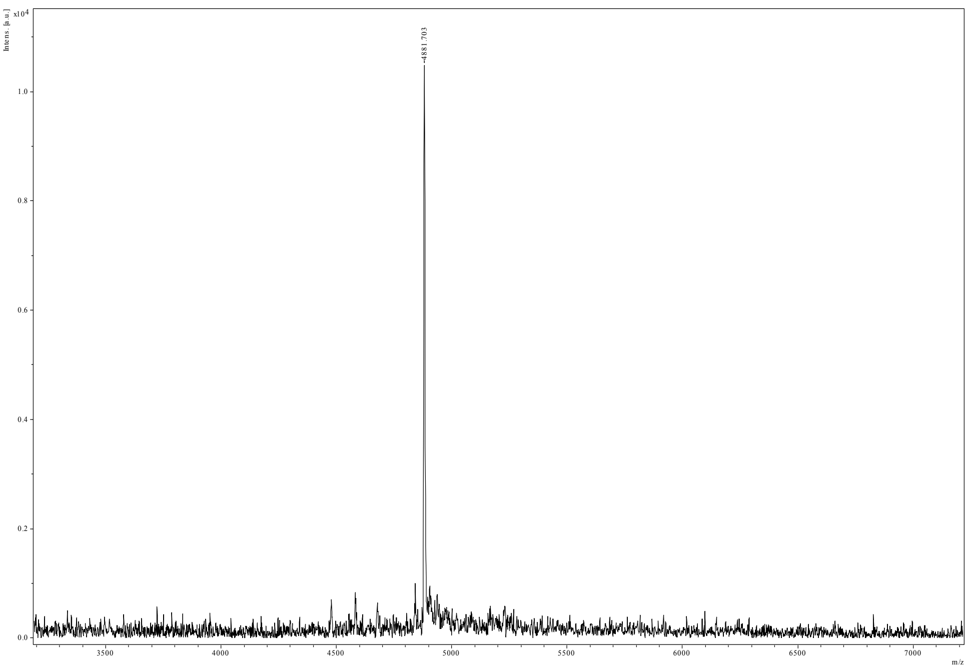

Data S78. MALDI-TOF MS of 5’-d(CCAGACATATA^{m}C^{F}G^{m}CG)-3’. Calcd. [M-H]$^{-}$: 4882.042; Found 4881.703.

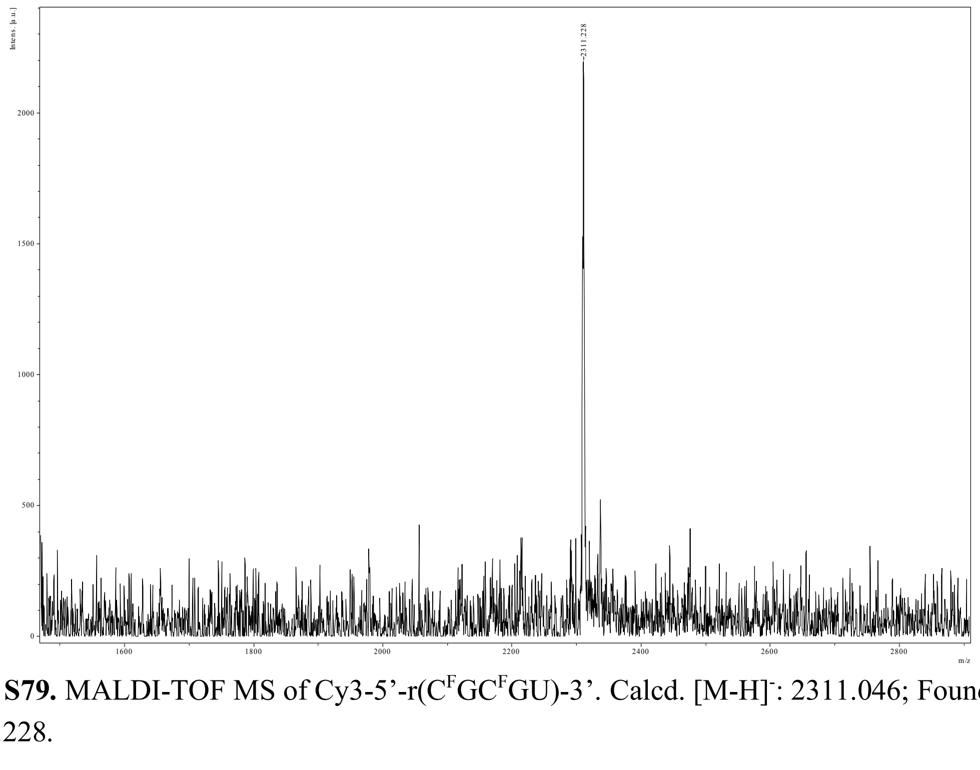

Data S79. MALDI-TOF MS of Cy3-5’-r(C^{F}GC^{F}GU)-3’. Calcd. [M-H]$^{-}$: 2311.046; Found 2311.228.

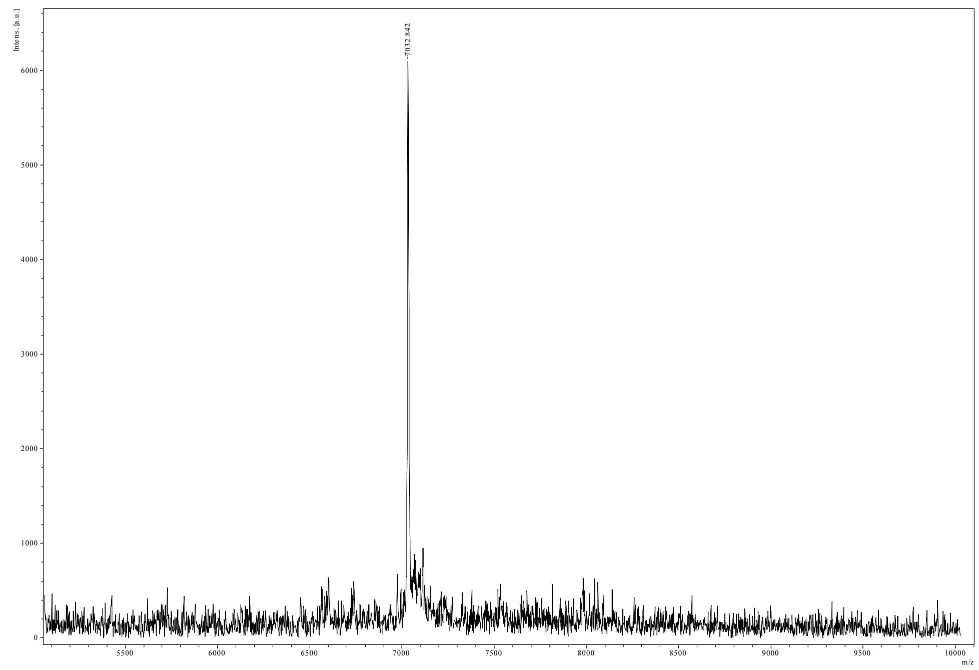

Data S80. MALDI-TOF MS of Cy3-5'-r(UCGCGGCCGCCGCGCGCGCG)-3'. Calcd. $[M-H]^-$: 7032.961; Found 7032.842.

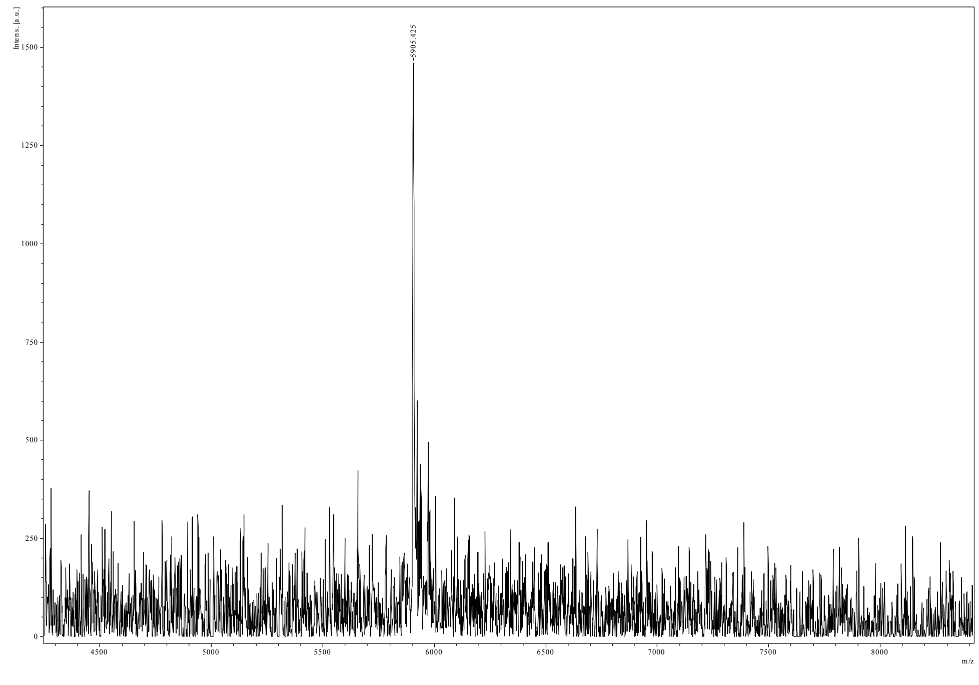

Data S81. MALDI-TOF MS of 5'-d(CCAGACATAT^{m}C^{F}G^{m}CA^{m}C^{F}G^{m}CG)-3'. Calcd. $[M-H]^-$: 5906.518; Found 5905.425.

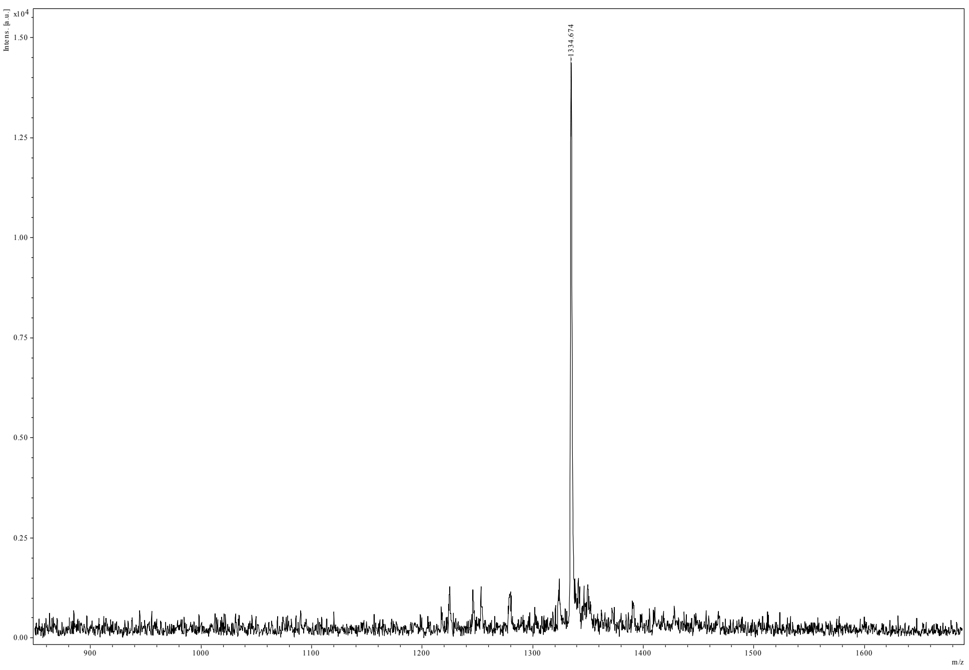

Data S82. MALDI-TOF MS of 5’-d(C^FG^mCG)-3’. Calcd. $[M-H]^-$: 1334.854; Found 1334.674.

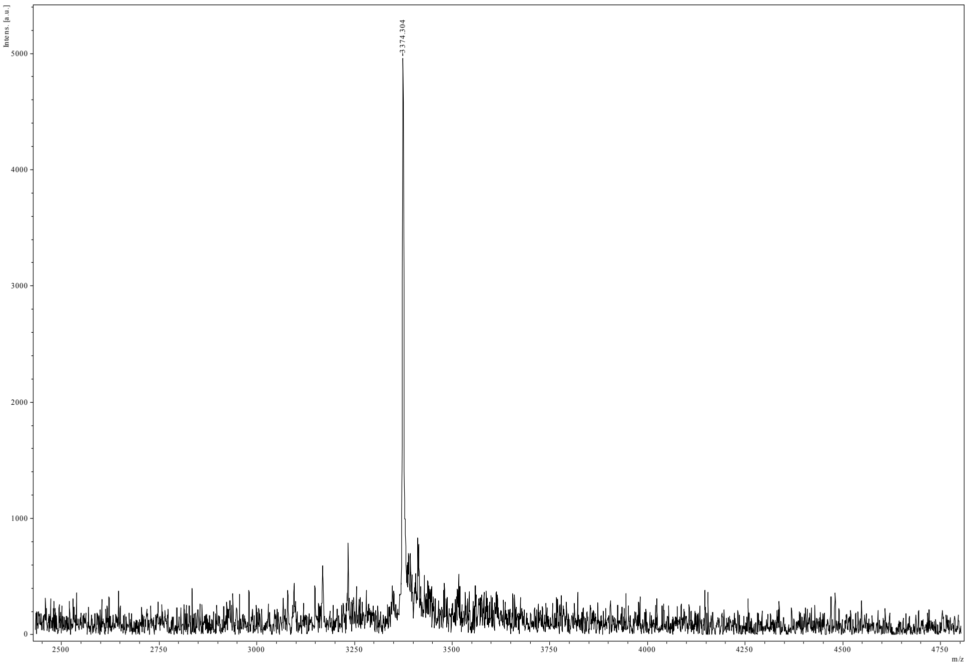

Data S83. MALDI-TOF MS of 5’-r($C^FGC^FGU^FGCG$)-3’. Calcd. $[M-H]^-$: 3374.676; Found 3374.304.

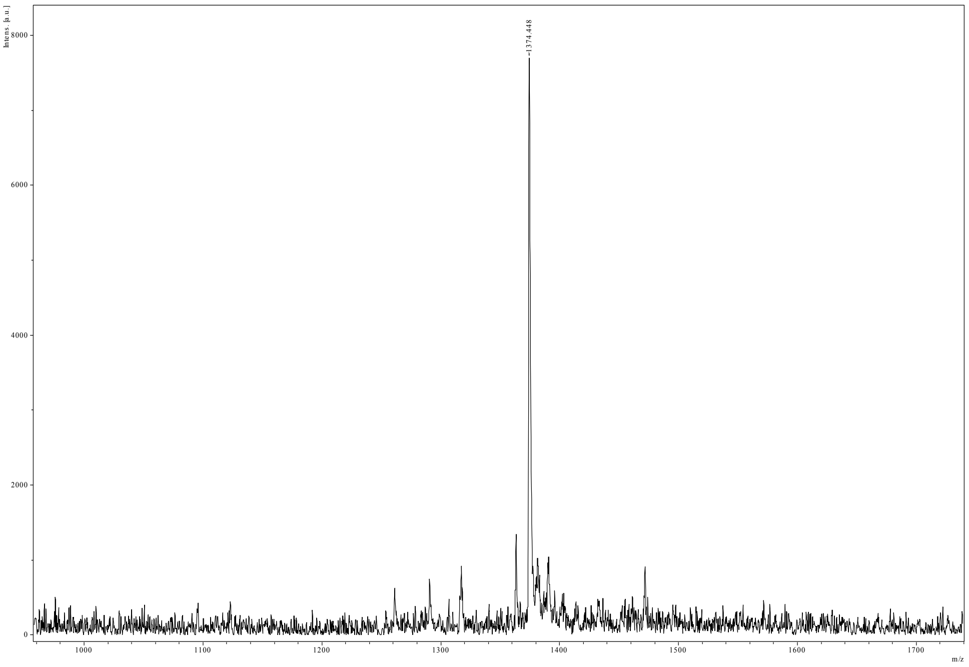

Data S84. MALDI-TOF MS of 5'-r(C^{F}GC^{F}G)-3'. Calcd. [M-H]$^-$: 1374.806; Found 1374.448.

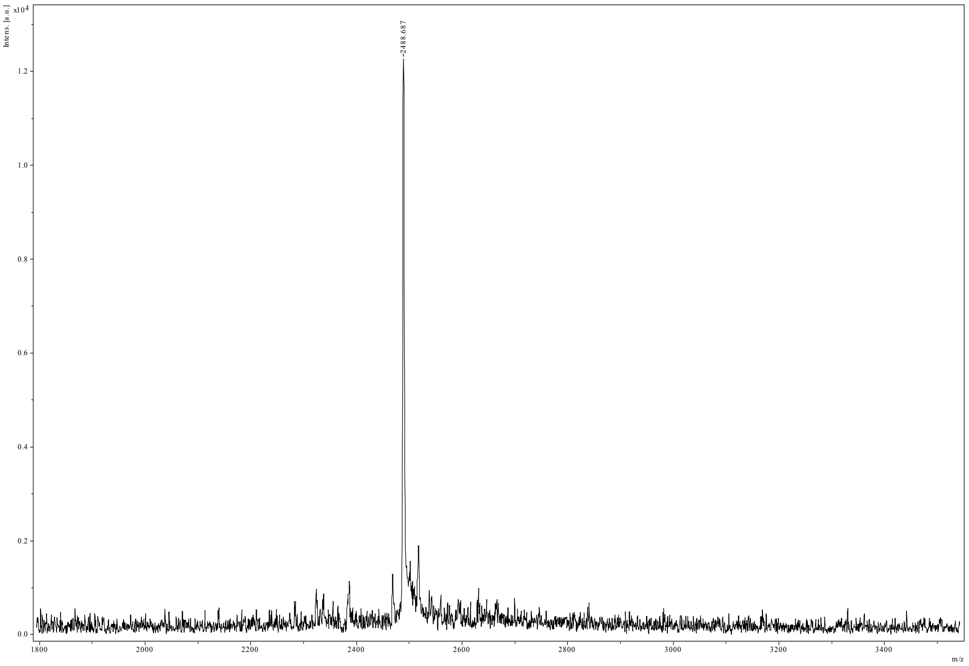

Data S85. MALDI-TOF MS of 5'-d(CGCACGCG)-3'. Calcd. [M-H]$^-$: 2488.505; Found 2488.687.

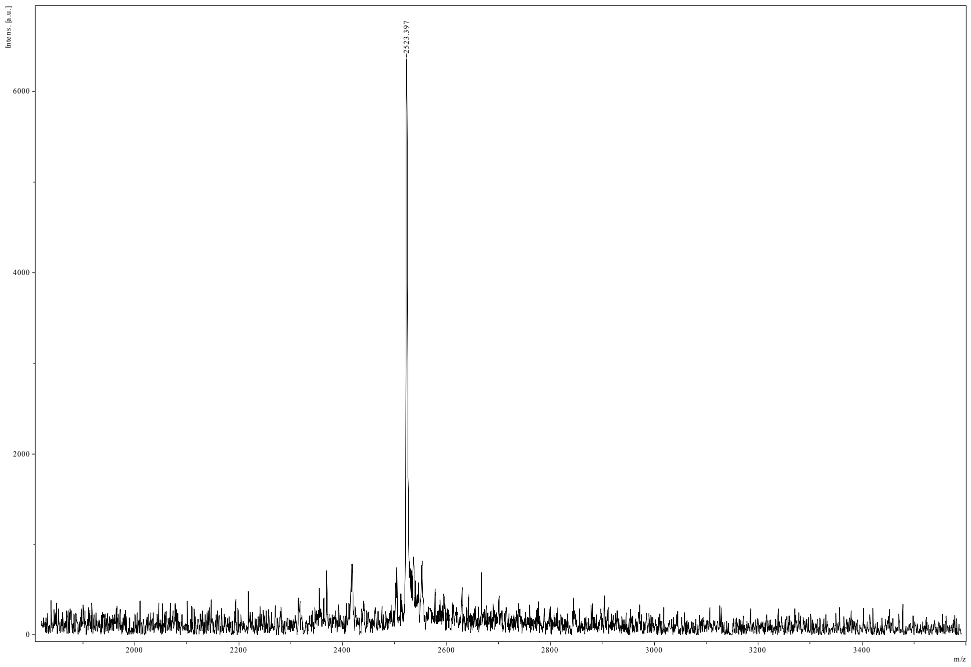

Data S86. MALDI-TOF MS of 5’-r(CGCGUGCG)-3’. Calcd. [M-H]$^-$: 2523.614; Found 2523.397.

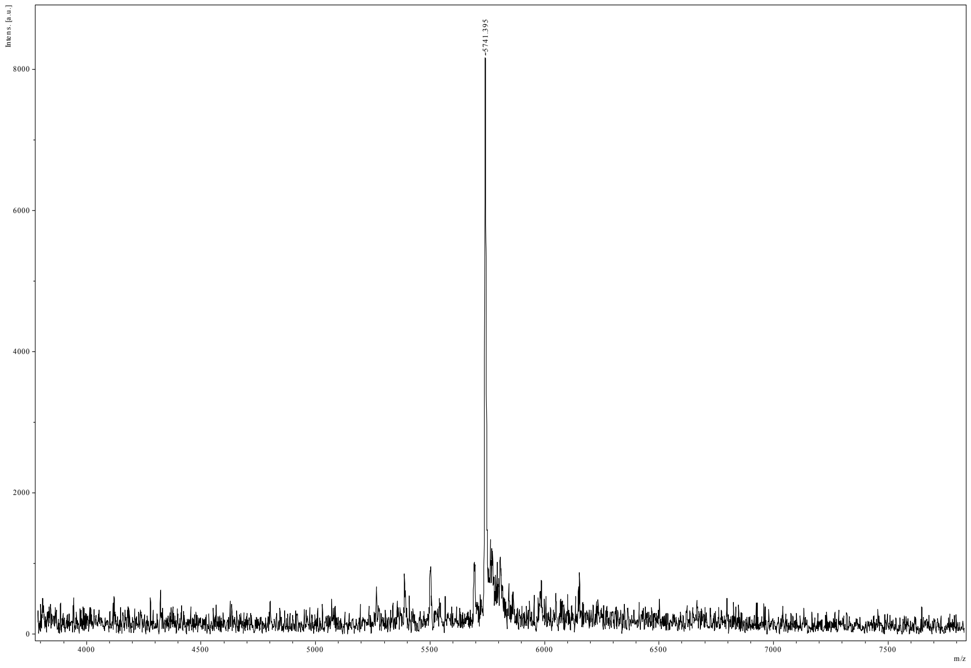

Data S87. MALDI-TOF MS of 5’-d(CCGAGCATATCGCACGCG)-3’. Calcd. [M-H]$^-$: 5741.516; Found 5741.395.

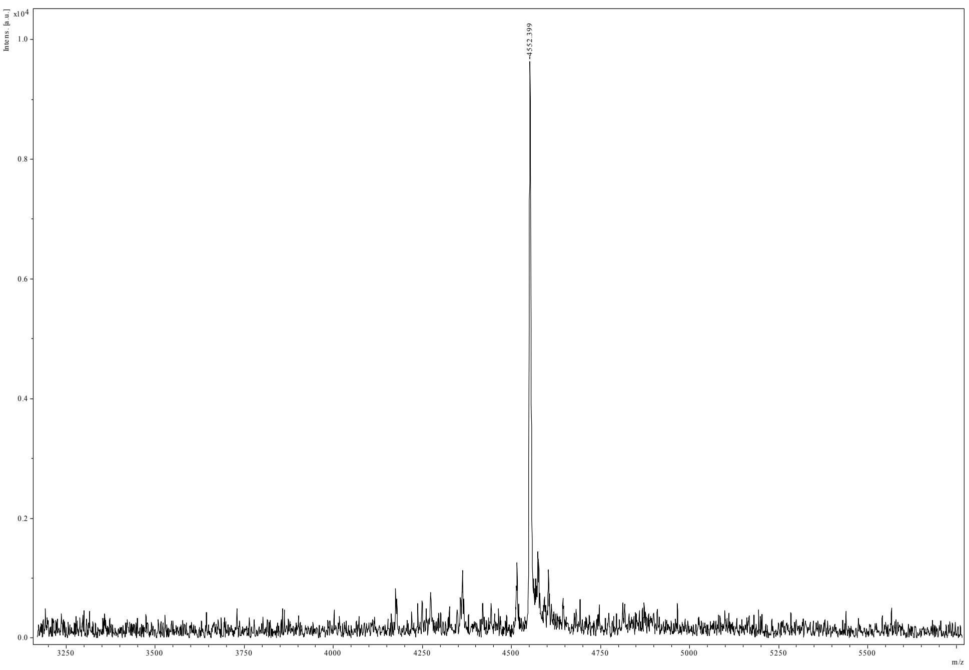

Data S88. MALDI-TOF MS of 5'-d(CCAGACATAT^{m}C^{F}G^{m}CG)-3'. Calcd. [M-H]$^{-}$: 4552.834; Found 4552.399.

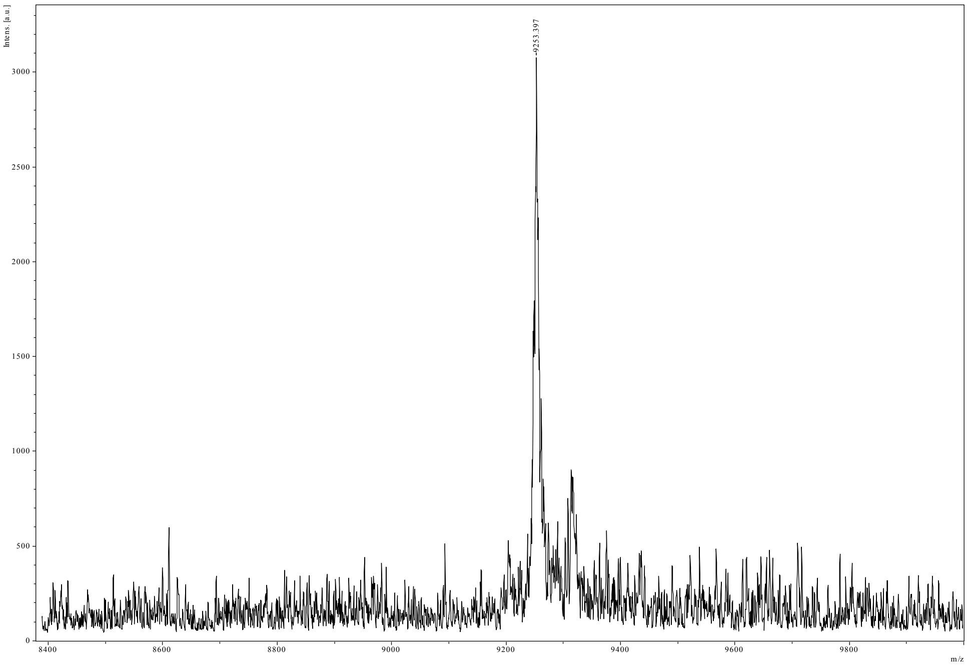

Data S89. MALDI-TOF MS of Cy3-5'-r(UGUCUCGCGCGCGCGCGCGCGCGUU)-3'. Calcd. [M-H]$^{-}$: 9253.453; Found 9253.397.
